# Isolation, Structure Elucidation, and Absolute Configuration of Germacrane Isomers from *Carpesium divaricatum*

**DOI:** 10.1038/s41598-018-30782-2

**Published:** 2018-08-20

**Authors:** Tao Zhang, Jia-Huan Chen, Jin-Guang Si, Gang Ding, Qiu-Bo Zhang, Hong-Wu Zhang, Hong-Mei Jia, Zhong-Mei Zou

**Affiliations:** 1 Institute of Medicinal Plant Development, Chinese Academy of Medical Sciences and Peking Union Medical College, Beijing, 100193 P. R. China; 2 School of Traditional Chinese Medicine, Shenyang Pharmaceutical University, Shenyang, 110016 P. R. China

## Abstract

Five sets of germacrane isomers (**1**/**8/17**, **2**/**7/10/11/13/16/18**, **3**/**4/5/14/20**, **6**/**12/15**, and **9**/**19**) with different skeletal types, including seven new ones (**1**–**3**, **8**–**9**, and **15**–**16**) were isolated from the whole plant of *Carpesium divaricatum*. Among them, there are six pairs of stereoisomers (**1**/**8**, **2**/**13**, **4**/**14**, **6**/**12**, **7**/**11** and **10/11**). The planar structures and relative configurations of the new compounds were elucidated by detailed spectroscopic analysis. The absolute configurations of **4**, **10**, **11**, and **17** were established by circular dichroism (CD) spectra and X-ray crystallographic analyses, and the stereochemistry of the new compounds **1**–**3**, **8**–**9**, and **15**–**16** were determined by similar CD spectra with **4**, **10**, **11**, and **17**, respectively. The confusion in the literature about subtypes I and II of germacranolides was clarified in this paper. The NMR data of **10**–**11**, and the absolute configurations of the known compounds **4**–**6**, **13**–**14**, and **17**–**20** were reported for the first time. Compounds **13**, **17**, and **18** showed cytotoxicity against human cervical (HeLa), colon (LoVo) and stomach cancer (BGC-823) cell lines with IC_50_ values in the range 4.72–13.68 *μ*M compared with the control *cis*-platin (7.90–15.34 *μ*M).

## Introduction

The genus *Carpesium* (Asteraceae) includes 25 species worldwide, most of which are distributed across Asia and Europe, particularly in southwest China^1,2^. The plant *Carpesium divaricatum*, as a Chinese folk medicine, has been used for the treatment of fevers, colds, bruises, and inflammatory diseases^3–7^. Previous investigations indicated that a series of diverse compounds were isolated, including sesquiterpenoid lactones, acyclic diterpenes, and thymol derivatives, with the sesquiterpenoid lactones being the major constituents^5–11^.

Germacranolides are one of the main sesquiterpene lactones, reported with broad bioactivities including cytotoxicity, anti-inflammation and anti-malaria^2,4,10–13^. So far, 54 germacranolides from the *Carpesium* plants have been reported^2,12,14^. The parent nucleus of the germacranolides contains a 5-membered *γ*-lactone ring fused to a circular 10-membered carbocycle, with different post-modifications to produce complex and diverse structural features. In addition, these germacranolides contain as many as nine stereogenic centers, creating the problem of stereo configuration. The relative configurations of 2, 5-hemiacetal-linked germacranolides are often deduced by NOESY analysis^7–9,11,15–20^, but their absolute configurations have rarely been reported^12,14^. The undefined absolute configuration and the incorrect depiction of the epoxy bonds cause significant confusion within the structures of this kind of compounds^7–9,11,15–20^.

**Figure 1:**
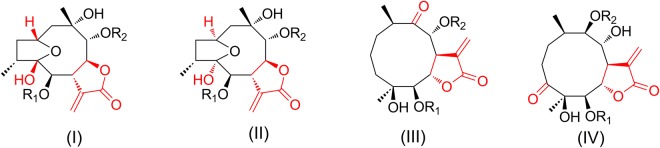
Four subtypes of germacranolides.

In our continuing effort to search for bioactive constituents from *C. divaricatum*, germacrane isomers attracted our attention due to their structural diversity. Five sets of germacrane isomers (**1**/**8/17**, **2**/**7/10/11/13/16/18**, **3**/**4/5/14/20**, **6**/**12/15**, and **9**/**19**), including seven new ones (**1**–**3**, **8**–**9**, and **15**–**16**) were indentified in the current investigation. Notably, these germacranolides include six pairs of stereoisomers (**1**/**8**, **2**/**13**, **4**/**14**, **6**/**12**, **7**/**11** and **10/11**). Structurally, these germacrane isomers belong to different skeletal types. According to the configuration of 2,5-hemiacetal group, linkage site of the ketone group and the position of 5-membered *γ*-lactone ring, the germacranolides could be further divided into several subtypes (Fig. 1). Compounds **1**–**7** together with the reported compounds ineupatolide B–C^21^ are attributable to subtype I (named 2*β*,5*β*-epoxygermacranolide), which contains a 5-membered *α*-methylene-*γ*-lactone ring linkage at C-7 and C-8, and the 2*β*,5*β*-hemiacetal group on the macro-ring system. Compounds **8**–**14** and the known compounds divaricin A–C^8^ are assigned as subtype II (named 2*α*,5*α*-epoxygermacranolide), having a 7,8-*α*-methylene-*γ*-lactone ring and the 2*α*,5*α*-hemiacetal group. The known compounds incaspitolide A–C^22,23^ and eight germacranolides we previously reported from *C. divaricatum*^12^ are classified as subtype III (named 9-oxo-germacranolide) with structural features of one 6,7-*α*-methylene-*γ*-lactone ring and the 9-ketone group. Compounds **15**–**20** represent subtype IV (named 3-oxo-germacranolide), possessing a 6,7-*α*-methylene-*γ*-lactone ring and the 9-ketone group. Both subtypes I and II are 2,5-hemiacetal-linked germacranolides, but their configurations at the bridgehead carbons between 5-membered ring and 9-membered ring are opposite. Subtypes III and IV have similar skeletons, but linkage sites of the ketone group are different (9-ketone group in subtype III and 3-ketone group in subtype IV). Twenty analogues here we further isolated from same species represent other three subtypes of germacranolides (subtypes I–II and IV). NOESY spectrum, circular dichroism (CD) method and X-ray data analysis were used to confirm their relative and absolute configurations. In this paper, the isolation, the structural elucidation, the absolute configuration and bioactive evaluation of these compounds were present. The confusion in the literature about subtypes I and II of germacranolides is also discussed.

## Results and Discussion

### Structural Elucidation of Compounds from Subtype I

**Figure 2:**
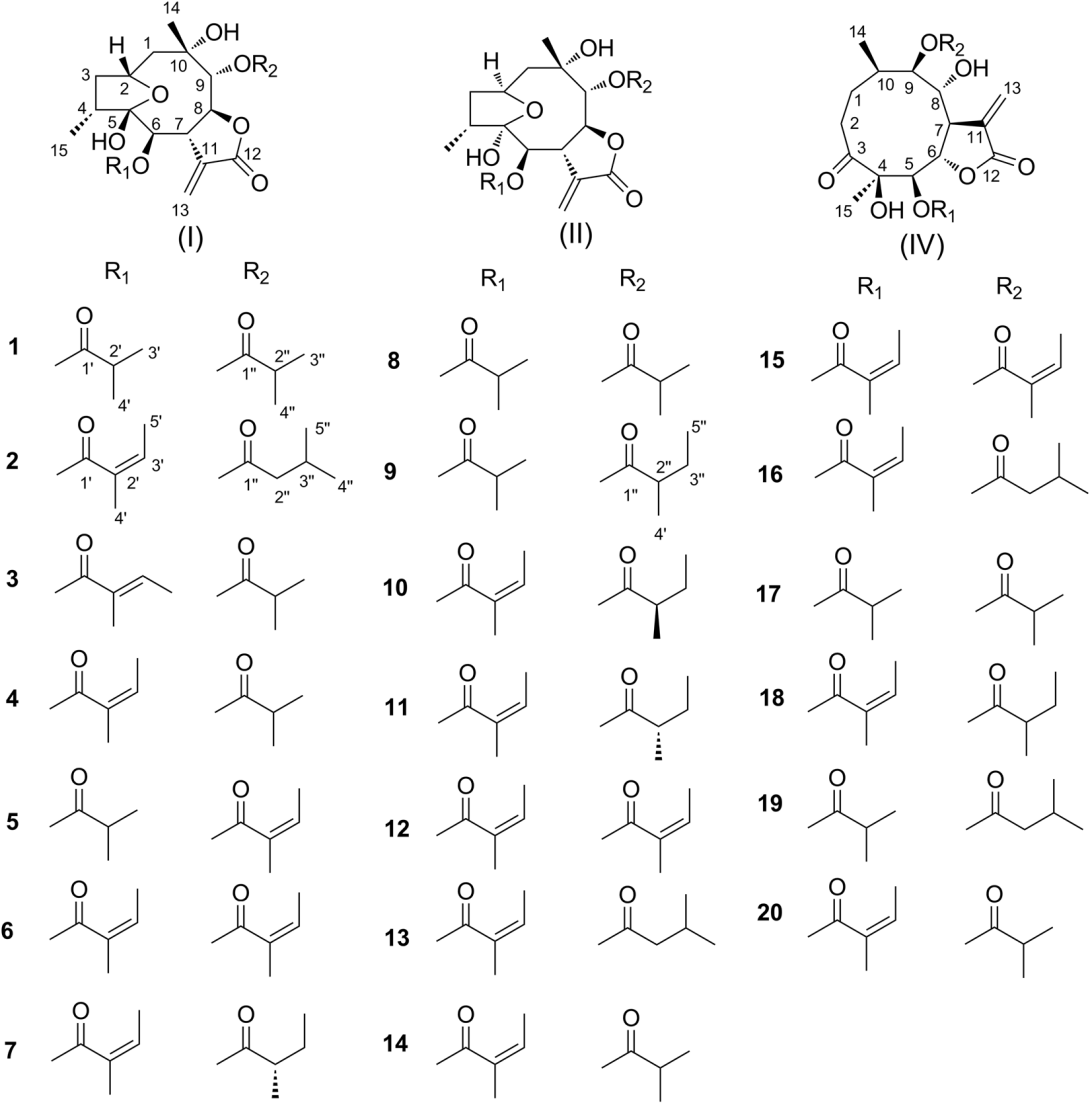
Chemical structures of compounds **1**–**20**.

**Figure 3:**
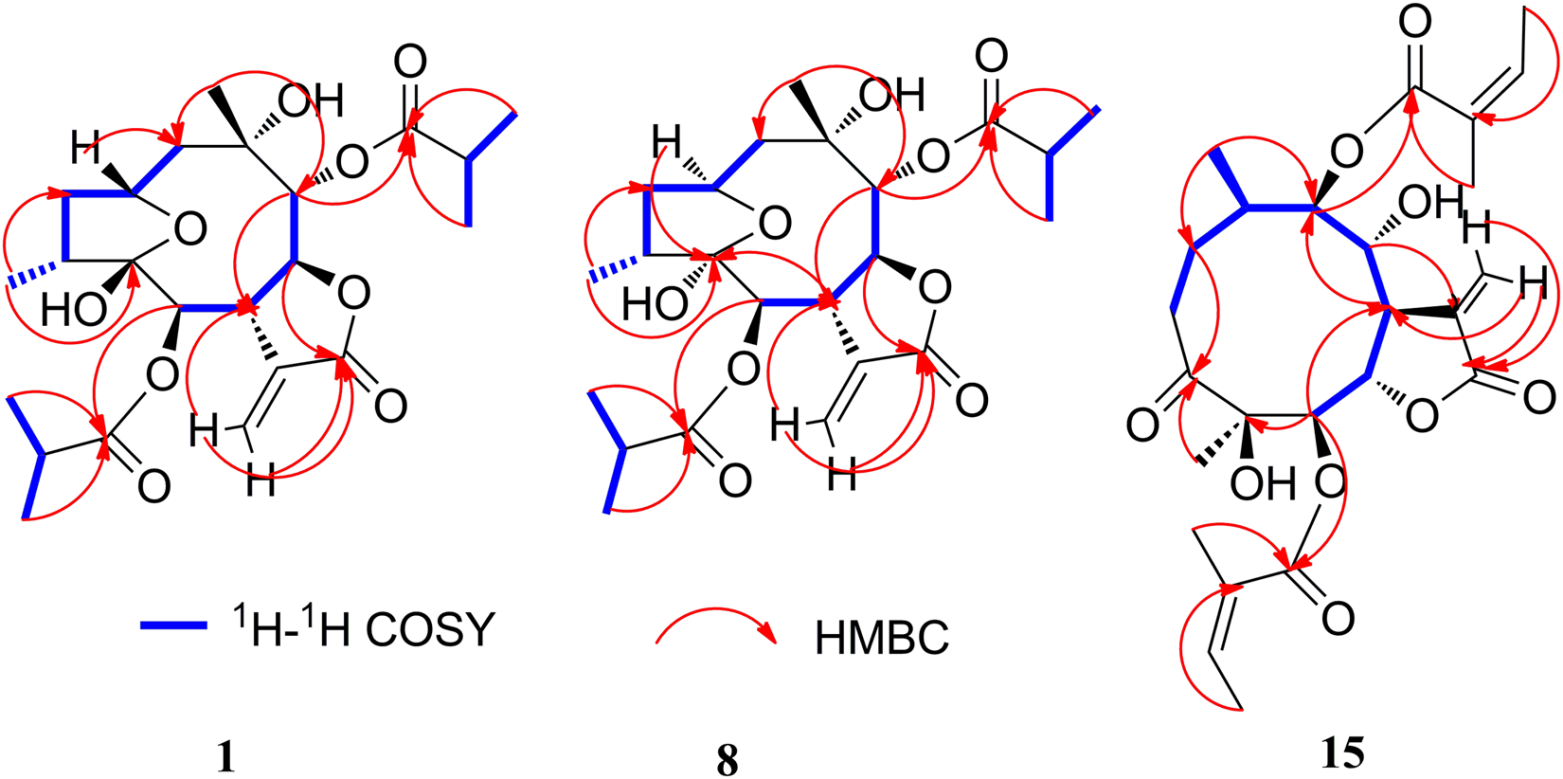
Key ^1^H-^1^H COSY and HMBC correlations of compounds **1**, **8**, and **15**.

Compound **1** (Fig. 2) was obtained as white needles. The molecular formula was assigned as C_23_H_34_O_9_ on the basis of the positive-ion HRESIMS ion at *m/z* 477.2104 [M + Na]^+^, together with its ^1^H and ^13^C NMR data (Tables 1 and 2). The IR spectrum showed the presence of hydroxyl (3493 cm^−1^) and carbonyl (1767 and 1737 cm^−1^) functional groups. The ^1^H NMR spectrum of **1** displayed the presence of two isobutyryloxy groups, which was further confirmed by the HRESIMS data of **1** with fragment ions at *m/z* 367.1758 [M + 1-HOiBu]^+^ and 261.1127 [M + 1-HOiBu-HOiBu-H_2_O]^+^. The ^1^H and ^13^C NMR spectra of **1** also showed an *α*-methylene-*γ*-lactone moiety at *δ*_H_ 5.68 (1 H, dd, *J* = 3.0, 1.2 Hz, Ha-13) and 6.13 (1 H, dd, *J* = 3.6, 1.2 Hz, Hb-13), *δ*_C_ 134.8 (C-11), 124.2 (C-13) and 170.2 (C-12); two lactone carbonyl carbons at *δ*_C_ 176.6 (C-1′) and 178.0 (C-1″); a dioxygenated carbon at *δ*_C_ 105.6 (C-5); one oxygenated tertiary carbon at 72.0 (C-10); five methines including four oxygenated ones at *δ*_H_ 4.30 (1 H, m, H-2), 4.87 (1 H, d, *J* = 7.2 Hz, H-6), 3.90 (1 H, m, H-7), 4.70 (1 H, dd, *J* = 6.0, 6.0 Hz, H-8) and 5.07 (1 H, d, *J* = 4.8 Hz, H-9), *δ*_C_ 70.9 (C-2), 74.4 (C-6), 45.9 (C-7), 78.1 (C-8), and 80.3 (C-9); and two methyl groups at *δ*_H_ 1.33 (3 H, s, CH_3_-14), 0.99 (3 H, d, *J* = 7.2 Hz, CH_3_-15). These observations and analyses of ^1^H-^1^H COSY, HSQC, and HMBC spectra (Fig. 3) suggested that the structure of **1** was similar to that of 2*β*,5-epoxy-5,10-dihydroxy-6*α*-angeloyloxy-9*β*-isobutyryloxy-germacran-8*α*,12-olide (**4**)^9^, except for an isobutyryloxy group in **1** compared to the angeloyloxy group at C-6 in **4**.

**Table 1:** ^1^H NMR Spectroscopic Data for Compounds **1**–**16** (*δ* in ppm, *J* in Hz).

No.	1^a^	2^a^	3^b^	4^a^	5^b^	6^a^	7^a^	8^a^
1a	1.90 dd (15.6, 12.0)	1.92 m	1.98 m	1.98 m	1.96 o^c^	1.95 o	1.98 m	2.04 dd (15.6,12.0)
1b	1.83 dd (15.6, 4.2)	1.84 dd (15.0, 4.2)	1.87 m	1.89 dd (15.6, 4.2)	1.89 m	1.86 o	1.90 dd (15.6, 4.2)	1.61 dd (15.6, 4.2)
2	4.30 m	4.30 m	4.33 m	4.36 m	4.35 m	4.32 m	4.36 m	4.55 m
3a	2.56 m	2.57 m	2.69 m	2.72 m	2.62 o	2.59 m	2.63 m	1.92 m
3b	1.40 m	1.40 m	1.40 m	1.46 m	1.45 m	1.41 m	1.47 m	1.75 dd (12.0, 6.6)
4	2.30 m	2.31 m	2.32 m	2.36 m	2.36 m	2.32 m	2.37 m	2.69 m
6	4.87 d (7.2)	4.99 d (7.8)	4.98 d (7.0)	5.04 d (7.2)	4.94 d (7.0)	5.01 d (6.6)	5.04 d (7.2)	5.02 d (10.8)
7	3.90 m	3.92 m	3.94 m	3.97 m	4.03 m	4.00 m	3.97 m	3.30 dd (10.8,1.2)
8	4.70 dd (6.0, 6.0)	4.74 dd (5.4, 5.4)	4.75 dd (6.5, 5.0)	4.80 dd (6.0, 5.4)	4.80 dd (6.0, 5.5)	4.79 dd (5.4, 5.4)	4.80 dd (6.0, 5.4)	5.24 dd (10.2,1.2)
9	5.07 d (6.0)	5.12 d (5.4)	5.14 d (5.0)	5.18 d (5.4)	5.23 d (5.5)	5.22 d (5.4)	5.18 d (5.4)	4.59 d (10.2)
13a	6.13 dd (3.6, 1.2)	6.06 dd (3.0, 0.6)	6.04 dd (3.5, 1.0)	6.11 d (3.0)	6.19 d (3.0)	6.07 dd (3.6, 1.2)	6.11 dd (3.0, 0.6)	6.17 br s
13b	5.68 dd (3.6, 1.2)	5.60 dd (3.0, 0.6)	5.62 dd (3.5, 1.0)	5.65 d (3.0)	5.75 d (3.0)	5.60 dd (3.6, 1.2)	5.65 dd (3.0, 0.6)	5.73 d (1.2)
14	1.33 s	1.40 s	1.36 s	1.40 s	1.41 s	1.36 s	1.40 s	1.19 s
15	0.99 d (7.2)	0.99 d (7.2)	0.99 d (7.5)	1.04 d (7.2)	1.06 d (7.0)	1.00 d (7.2)	1.04 d (7.2)	1.14 d (6.6)
2′	2.54 m				2.61 o			2.53 m
3′	1.15 d (7.2)	6.21 qq (7.2, 1.8)	7.02 q (8.5)	6.25 qq (7.8, 1.8)	1.20 d (7.0)	6.21 qq (7.2, 1.8)	6.25 qq (7.2, 1.8)	1.10 d (7.2)
4′	1.07 d (7.2)	1.90 dq (1.8, 1.2)	1.82 br s	1.96 dq (1.8, 1.2)	1.13 d (7.0)	1.91 dq (1.8, 1.2)	1.96 dq (1.8, 1.2)	1.13 d (7.2)
5′		1.96 dq (7.2, 1.2)	1.84 d (8.5)	2.01 dq (7.8, 1.2)		1.97 dq (7.2, 1.2)	2.01 dq (7.2, 1.2)	
2″	2.56 m	2.27 o, 2.27 o	2.59 m	2.62 m			2.53 m	2.64 m
3″	1.15 d (7.2)	2.08 m	1.18 d (7.5)	1.21 d (7.2)	6.08 qq (7.0, 1.5)	6.04 qq (7.2, 1.8)	1.79 m,1.46 m	1.16 d (7.2)
4″	1.14 d (7.2)	0.94 d (7.2)	1.17 d (7.5)	1.20 d (7.2)	1.97 d (1.5)	1.93 dq (1.8, 1.2)	1.20 d (7.2)	1.20 d (7.2)
5″		0.93 d (7.2)			1.92 dq (7.0, 1.0)	1.86 dq (7.2, 1.2)	0.96 t (7.8)	
**No**.	**9** ^**b**^	**10** ^**a**^	**11** ^**a**^	**12** ^**b**^	**13** ^**a**^	**14** ^**a**^	**15** ^**b**^	**16** ^**b**^
1a	2.07 dd (15.5, 12.5)	2.05 dd (15.6, 12.6)	2.06 dd (15.6, 12.6)	2.13 dd (15.5, 12.0)	2.05 dd (15.6, 12.0)	2.05 dd (15.6, 12.0)	1.87 m	1.87 m
1b	1.63 dd (15.5, 4.0)	1.62 dd (15.6, 4.2)	1.62 dd (15.6, 4.2)	1.67 dd (15.5, 4.0)	1.61 dd (15.6, 4.2)	1.61 dd (15.6, 4.2)	1.74 m	1.65 m
2	4.57 m	4.56 m	4.57 m	4.62 m	4.56 m	4.56 m	3.87 m, 2.24 o	3.88 m, 2.23 o
3a	1.94 m	1.94 m	1.95 m	1.97 o	1.93 m	1.93 m		
3b	1.78 m	1.77 dd (12.0, 6.6)	1.78 dd (12.0, 6.6)	1.83 dd (12.0, 7.0)	1.77 dd (12.0, 7.2)	1.77 dd (12.0,7.2)		
4	2.72 m	2.72 m	2.72 m	2.77 m		2.72 m		
5							5.51 dd (10.0, 2.0)	5.52 dd (9.5, 2.0)
6	5.04 d (11.5)	5.17 d (10.8)	5.17 d (10.8)	5.22 d (11.0)	5.16 d (10.8)	5.17 d (10.8)	4.73 dd (10.0, 6.5)	4.73 dd (9.5, 6.5)
7	3.34 dd (11.5,1.5)	3.32 dd (10.8, 1.2)	3.33 dd (10.8, 1.2)	3.41 br d (11.0)	3.36 dd (10.8,1.2)	3.33 dd (10.2, 1.2)	3.08 m	3.06 m
8	5.26 br d (10.0)	5.28 dd (10.2, 1.2)	5.27 dd (10.2, 1.2)	5.33 br d (10.0)	5.26 dd (10.2,1.2)	5.27 br d (9.6)	4.48 d (10.5)	4.44 d (10.5)
9	4.61 d (10.0)	4.60 d (10.2)	4.60 d (10.2)	4.73 d (10.0)	4.59 d (10.2)	4.60 d (9.6)	5.27 d (10.5)	5.18 d (10.5)
10							2.24 o	2.23 o
13	6.19 br s, 5.76 br s	6.11 d (1.2), 5.67 d (1.2)	6.11 d (1.2), 5.67 d (1.2)	6.17 br s, 5.73 br s	6.11 br s, 5.67 d (1.2)	6.11 br s, 5.67 br s	6.34 d (3.0), 5.71 d (3.0)	6.34 d (3.0), 5.69 d (3.0)
14	1.22 s	1.21 s	1.21 s	1.26 s	1.20 s	1.20 s	0.99 d (6.5)	0.99 d (6.5)
15	1.17 d (6.5)	1.13 d (6.6)	1.13 d (6.6)	1.18 d (6.5)	1.13 d (6.6)	1.13 d (6.6)	1.25 s	1.26 s
2′	2.56 m							
3′	1.13 d (7.0)	6.10 qq (6.6, 1.8)	6.10 qq (7.2, 1.8)	6.17 o	6.10 qq (5.4,1.8)	6.10 qq (6.0, 1.2)	6.18 o	6.20 qq (7.0, 1.5)
4′	1.16 d (7.0)	1.89 dq (1.8, 1.2)	1.89 dq (1.8, 1.2)	1.97 s	1.89 dq (1.8,1.2)	1.90 dq (1.2, 1.2)	2.00 s	1.99 q (1.5)
5′		1.90 dq (6.6, 1.2)	1.91 dq (7.2, 1.2)	1.95 dq (5.5, 1.5)	1.90 dq (5.4,1.2)	1.89 dq (6.0, 1.2)	1.96 br d (10.5)	2.02 dq (7.0, 1.5)
2″	2.47 m	2.46 m	2.47 m		2.28 o, 2.28 o	2.64 m		2.32 o, 2.32 o
3″	1.76 m,1.48 m	1.70 m,1.45 m	1.77 m,1.47 m	6.17 o	2.10 m	1.16 d (7.2)	6.18 o	2.13 o
4″	1.17 d (7.0)	1.19 d (6.6)	1.16 d (7.2)	1.97 s	0.97 d (7.8)	1.20 d (7.2)	2.00 s	1.01 d (5.5)
5″	0.97 t (7.5)	0.90 t (7.8)	0.96 t (7.8)	1.95 dq (5.5, 1.5)	0.96 d (7.8)		1.96 d (10.5)	1.01 d (5.5)

^a^Measured at 600 MHz in methanol-*d*_4_; ^b^Measured at 500 MHz in methanol-*d*_4_; ^c^Overlapped with other signals.

**Table 2:** ^13^C NMR Spectroscopic Data for Compounds **1**–**16** (*δ* in ppm).

No.	1^a^	2^a^	3^b^	4^a^	5^b^	6^a^	7^a^	8^a^	9^b^	10^a^	11^a^	12^b^	13^a^	14^a^	15^b^	16^b^
1	48.8	48.8	48.9	48.8	49.1	49.2	48.9	43.9	43.9	43.9	43.9	43.8	43.9	43.9	25.7	25.4
2	70.9	70.8	70.9	70.8	71.0	70.8	70.9	73.7	73.7	73.8	73.7	73.7	73.7	73.7	33.0	33.2
3	40.4	40.5	40.5	40.5	40.5	40.5	40.6	37.1	37.1	37.3	37.3	37.1	37.3	37.3	217.9	217.8
4	43.4	43.3	43.4	43.3	43.5	43.4	43.4	35.9	36.0	35.9	35.9	35.8	35.9	35.9	80.4	80.4
5	105.6	105.7	105.7	105.7	105.7	105.7	105.7	105.6	105.6	105.8	105.8	105.6	105.8	105.8	78.1	78.1
6	74.4	74.3	74.7	74.4	74.5	74.4	74.4	75.6	75.6	75.8	75.8	75.7	75.8	75.8	80.0	80.0
7	45.9	45.8	45.8	45.8	45.9	45.8	45.8	44.9	44.9	45.1	45.1	45.0	45.0	45.1	41.7	41.6
8	78.1	78.1	78.1	78.1	78.2	78.2	78.2	78.1	78.0	78.2	78.1	78.2	78.3	78.2	70.5	70.3
9	80.3	80.4	80.3	80.3	80.5	80.5	80.4	78.0	77.9	78.0	77.9	77.8	78.0	78.0	78.5	78.7
10	72.0	72.0	72.1	72.1	72.1	72.1	72.2	71.2	71.3	71.1	71.3	71.1	71.1	71.2	30.1	29.9
11	134.8	135.0	134.9	135.0	135.0	135.0	135.1	134.4	134.4	134.6	134.7	134.5	134.6	134.6	132.8	132.8
12	170.2	170.3	170.3	170.3	170.3	170.3	170.2	169.9	169.8	169.9	169.9	169.9	170.0	170.0	169.7	169.7
13	124.2	124.3	124.3	124.3	124.3	124.3	124.3	125.6	125.4	125.5	125.4	125.4	125.5	125.6	124.0	123.9
14	23.3	23.3	23.3	23.3	23.5	23.4	23.4	29.2	29.3	29.4	29.3	29.2	29.2	29.2	20.0	20.0
15	12.5	12.3	12.1	12.3	12.6	12.3	12.4	13.6	13.6	13.6	13.6	13.5	13.6	13.6	23.4	23.4
1′	176.6	166.7	167.0	166.7	176.6	166.7	166.8	176.1	176.1	166.3	166.3	166.2	166.4	166.4	167.1	167.1
2′	33.9	127.1	128.0	127.1	33.9	127.1	127.1	33.8	33.8	127.1	127.1	127.0	127.1	127.1	127.5	127.5
3′	18.0	140.4	139.2	140.3	18.1	140.4	140.4	18.0	18.0	139.4	139.4	139.3	139.4	139.4	138.4	138.4
4′	17.7	19.1	13.2	19.1	17.6	19.2	19.1	17.8	17.8	19.2	19.3	19.1	19.2	19.3	19.3	19.3
5′		14.7	10.6	14.7		14.7	14.7			14.6	14.6	14.5	14.6	14.6	14.6	14.6
1″	178.0	173.9	178.1	178.0	168.8	168.8	177.6	177.0	176.6	176.7	176.7	167.6	173.1	177.1	167.7	173.2
2″	33.5	42.2	33.5	33.5	127.8	127.8	41.0	33.8	41.1	41.4	41.1	127.7	42.6	33.8	127.8	43.2
3″	18.0	25.2	18.1	18.0	137.0	136.9	26.2	18.0	26.3	26.2	26.3	137.3	25.1	18.0	137.7	25.4
4″	17.9	21.4	18.0	17.9	19.3	19.1	15.9	17.8	15.7	16.0	15.7	19.1	21.4	17.8	19.5	21.4
5″		21.4			14.5	14.4	10.8		10.6	10.7	10.7	14.4	21.4		14.7	21.5

^a^Measured at 150 MHz in methanol-*d*_4_; ^b^Measured at 125 MHz in methanol-*d*_4_.

**Figure 4:**
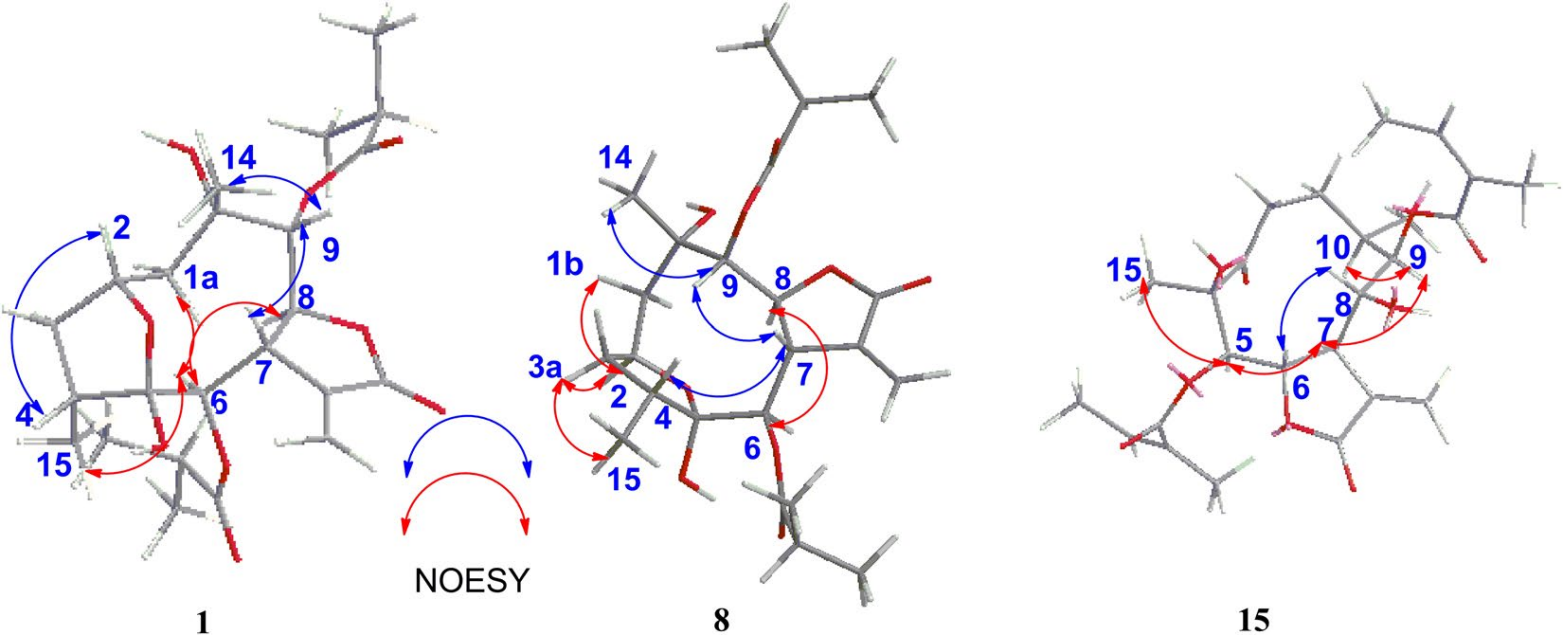
Key NOESY correlations of compounds **1**, **8**, and **15**.

The relative configuration of **1** was determined by analysis of the NOESY data (Fig. 4). The NOE correlations of H_3_-15/H-6 and H-6/H-8 indicated they were cofacial and were arbitrarily assigned as *α*-orientations, whereas the correlations of H-7/H-9 and H-9/H_3_-14 showed their *β*-orientations. The NOE correlations of H-2/H-4 and H-6/H_3_-15, and the lack of correlation of H-4 with H-7, suggested that H-2, and 5-OH were *β*-oriented. Thus, the relative configuration of **1** was established.

Compound **2** possessed molecular formula of C_25_H_36_O_9_ based on the HRESIMS ion at *m/z* 503.2261 [M + Na]^+^. The ^1^H and ^13^C NMR data of **2** were similar to those of **1**, except that the isobutyryloxy groups at C-6 and C-9 in **1** were replaced by an angeloyloxy group at C-6 and a 3-methylbutyryloxy group at C-9 in **2**. The ^1^H-^1^H COSY, HSQC, and HMBC spectra supported the structure of **2** as shown. The NOE correlations of H-2/H-4, H-6/H_3_-15, H-6/H-8, H-7/H-9 and H-9/H_3_-14 in **2** indicated that **2** had the same relative configuration as **1**.

Compounds **3**–**4** shared the same molecular formula C_24_H_34_O_9_ from their HRESIMS at *m/z* 489.2112 [M + Na]^+^ and *m/z* 489.2100 [M + Na]^+^. The ^1^H and ^13^C NMR data of **3** showed a great similarity with those of **4**, except for the ester residues at C-6. The angeloyloxy group at C-8 in **4** was placed by a tigloyloxy group in **3**^24^. The ^1^H-^1^H COSY, HSQC and HMBC spectra of **3** confirmed this observation, leading to the assignment of its planar structure. The relative configuration of **3** was deduced to be the same as **4**, on the basis of similar ROESY data.

Compounds **4–7** were identified as 2*β*,5-epoxy-5,10-dihydroxy-6*α*-angeloyloxy-9*β*-isobutyryloxy-germacran-8*α*,12-olide (**4**)^9^, ineupatolide A (**5**)^21^, 2*β*,5-epoxy- 5,10-dihydroxy-6*α*,9*β*-diangeloyloxy-germacran-8*α*,12-olide (**6**)^15–20^ and ineupatolide (**7**)^8,14,15^ by comparison of their MS, ^1^H NMR, and ^13^C NMR spectroscopic data, as well as optical rotation data with reported data. The conclusions were also confirmed by the ^1^H-^1^H COSY, HSQC, HMBC, and NOESY spectra. However, only compound **7** has been reported the absolute configuration before.

**Figure 5:**
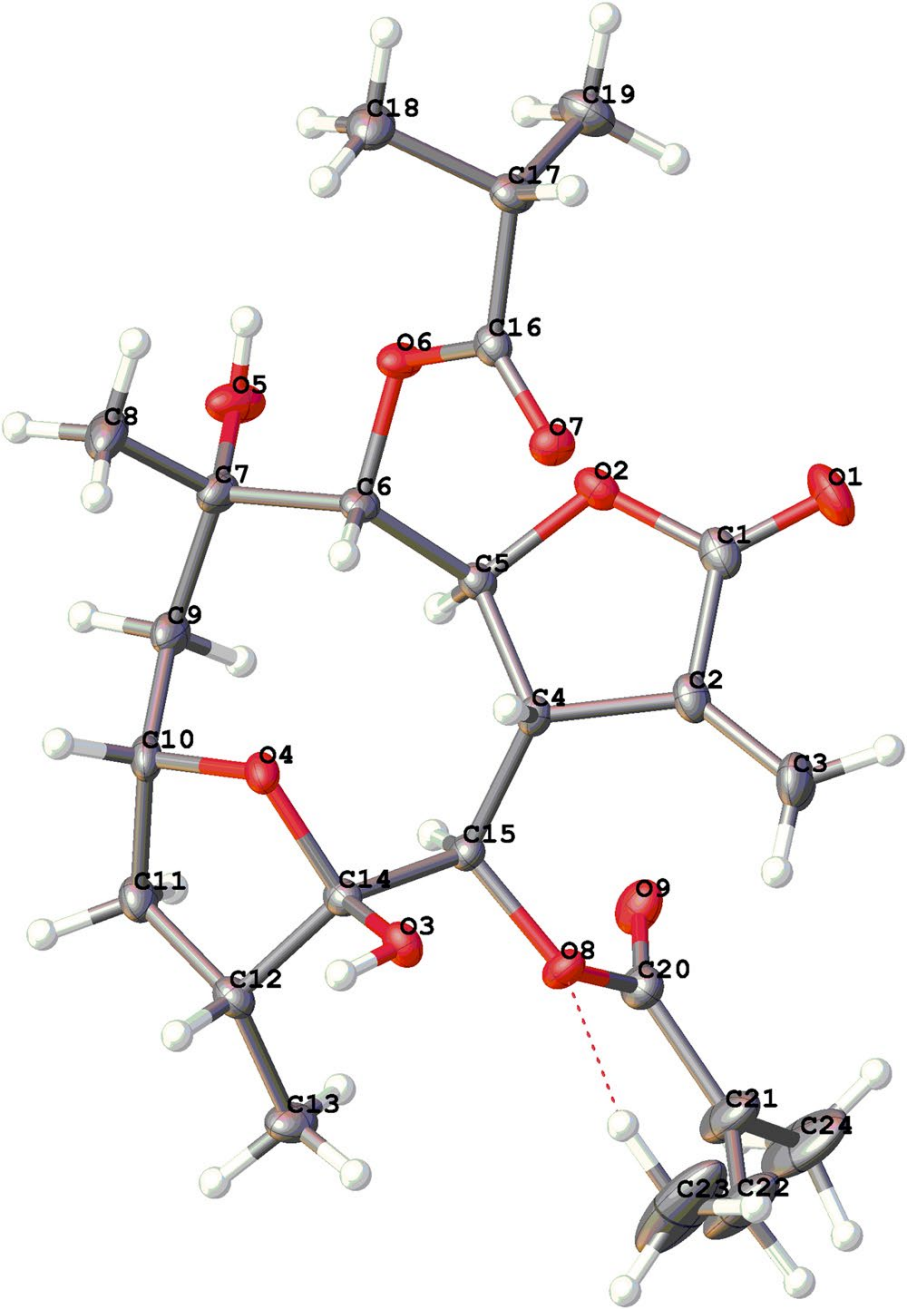
X-ray ORTEP drawing of **4**.

Fortunately, a single crystal of **4** was obtained from MeOH. The X-ray crystallographic analysis [flack parameter:−0.10(12)] unambiguously established the absolute configuration of **4** as 2*R*, 4*R*, 5*S*, 6*R*, 7*S*, 8*S*, 9*R*, and 10*S* (Fig. 5). Based on the established absolute configuration of **4**, we deduced the absolute configurations of **1–3** and **4–6** from similar CD data (supplementary information Fig. C1). Due to the fact that there are some differences in the CD spectra of **1** and **5**, the absolute configurations of **1** and **5** were further confirmed by using quantum chemical electronic circular dichroism (ECD) calculations. It was clear that the calculated ECD spectra of (2*R*, 4*R*, 5*S*, 6*R*, 7*S*, 8*S*, 9*R*, 10*S*)-**1** and **5** were matched very well with the experimental ECD spectra of **1** and **5** (supplementary information C2). Thus, the structures of compounds **1–6** were defined as shown, named (2*R*, 5*S*)-cardivarolide A (**1**), (2*R*, 5*S*)-cardivarolide B (**2**), (2*R*, 5*S*)-ciscardivarolide C (**3**), (2*R*, 5*S*)-cardivarolide C (**4**), ineupatolide A (**5**), and (2*R*, 5*S*)-cardivarolide D (**6**), respectively.

This type of germacranolides has significant confusion because almost all of the compounds have been determined by comparison of their spectroscopic data with those of ineupatolide (**7**)^15^. In addition to the previous incorrect assignment of the absolute configuration of ineupatolide (**7**)^15,21^, the depiction of the epoxy bonds, in which the bonds of 2,5-epoxy group were depicted with bold or dashed lines, is also incorrect^15,16^. Thus, it is suggested that the structures of germacranolides of this type (subtype I) should be depicted as shown.

### Structural Elucidation of Compounds from Subtype II

Compound **8** had the same molecular formula (C_23_H_34_O_9_, *m*/*z* 477.2101 [M + Na]^+^ in HRESIMS) and the same planar structure as **1** according to the ^1^H-^1^H COSY, HSQC, and HMBC spectra (Fig. 3). Comparing the ^1^HNMR data of **8** with **1**, the main difference was downfield signals of H-7 (*δ* 3.30 in **8** and 3.90 in **1**), H-9 (*δ* 4.59 in **8** and 5.07 in **1**), and H-8 (*δ* 5.24 in **8** and 4.70 in **1**) in **8** (see Table 1). These results suggested that **8** and **1** had different configurations^8^. The relative configuration of **8** was elucidated by NOESY analysis (Fig. 4). The NOE correlations of H-4/H-7, H_3_-15 (4-CH_3_) /H-3a, H-3a/H-2 and H-2/H-1b, indicated that H-2, H_3_-15, and 5-OH had *α*-orientations^8^. The *β-*orientations of H-7, H-9 and H_3_-14, and *α*-orientations of H-6 and H-8 could be deduced from the correlations of H-4/H-7, H-7/H-9, H-9/H_3_-14 and H-8/H-6. Compared with **1**, compound **8** had the opposite configurations of H-2 and 5-OH linkage to the bridgehead carbons between 5-membered ring and 9-membered ring. Therefore, H-4 in space was much closer to H-7 but H_3_-15 was far away from H-6 in **8**, compared to those of **1** (supplementary information Fig. C3). Thus, NOE correlations of H_3_-15/H-6 and H-4/H-6 were not observed in **8**. On the basis of these data, the relative configuration of **8** was established.

The molecular formula of compound **9** was assigned as C_24_H_36_O_9_ by the positive-ion HRESIMS ion at *m/z* 491.2271 [M + Na]^+^, the same as that of **20**. The ^1^H and ^13^C NMR data of **9** were similar to those of **8**, except for the ester residues at C-9. The 2-methylbutyryloxy group at C-9 appeared in **9** instead of the isobutyryloxy group in **8**. The ^1^H-^1^H COSY, HSQC, and HMBC spectra supported the structure of **9** as shown. The NOE correlations of H-4/H-7, H-8/H-6, H-7/H-9 and H-9/H_3_-14 in **8** and **9** indicated that **9** had the same relative configuration as **8**.

Compounds **10**–**14** were identified as (2*S*, 4*R*, 5*R*, 6*R*, 7*S*, 8*S*, 9*R*, 10*S*, 2″*R*) -2,5-epoxy-5,10-dihydroxy-6-angeloyloxy-9-2-methylbutyryloxy-germacran-8,12-olide (**10**)^14,25,26^, (2*S*, 4*R*, 5*R*, 6*R*, 7*S*, 8*S*, 9*R*, 10*S*, 2″*S*)-2,5-epoxy-5,10-dihydroxy-6-angeloyloxy-9-2-methylbutyryloxy-germacran-8,12-olide (**11**)^14,25,26^, (2*S*, 4*R*, 5*R*, 6*R*, 7*S*, 8*S*, 9*R*, 10*S*)-2,5-epoxy-5,10-dihydroxy-6-angeloyloxy-9-angeloyloxy-germacran-8,12-olide (**12**)^8,14,25,26^, 2*α*,5-epoxy-5,10-dihydroxy-6*α*-angeloyloxy-9*β*-3-methylbutyryloxy-germacran-8,12-olide (**13**)^18–20,25,26^, and 2*α*,5-epoxy-5,10-dihydroxy-6*α*-angeloyloxy-9*β*-isobutyryloxy-germacran-8,12-olide (**14**)^9,19,20,26^ by comparison of their MS, ^1^H NMR, and ^13^CNMR spectral with reported data. The conclusions were also confirmed by the ^1^H-^1^H COSY, HSQC, HMBC, and NOESY spectra. However, the absolute configurations of **13**–**14** have not been concluded in the literature.

**Figure 6:**
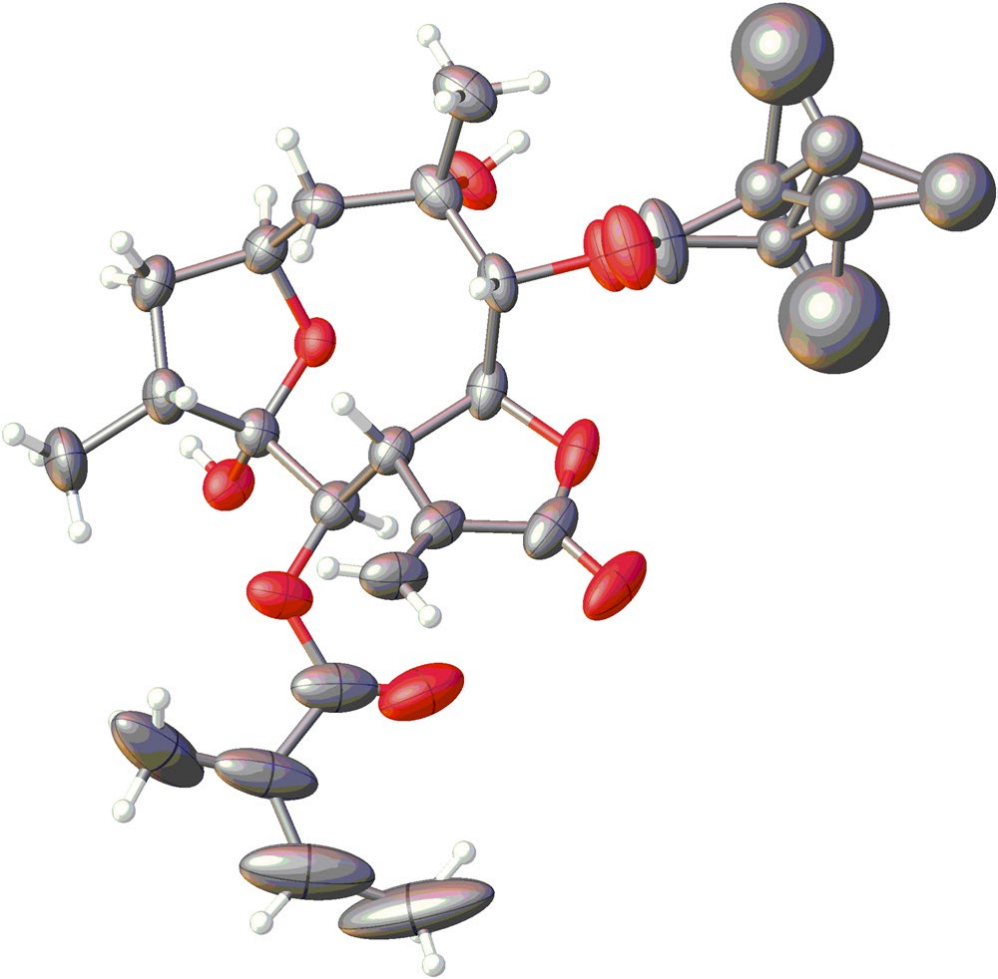
X-ray ORTEP drawing of the mixture crystals of **10** and **11**.

Herein, we further confirmed the absolute configurations of **10** and **11** by X-ray crystallographic analysis. Although a suitable single crystal of compound **10** or **11** was not obtained through many attempts with different solvents, mixed trigonal crystals of **10** and **11** (1:1) were obtained from MeOH. Both compounds have the same nuclear structures, and the only difference is that the absolute configuration of 2-methylbutyryloxy group at C-9 is 2″*R* or 2″*S*. Because the minor difference does not affect their crystal structures, the X-ray diffraction experiment of the mixture crystal supported the absolute configuration of the nuclear structure. The X-ray crystallographic analysis [flack parameter = −0.0(2)] unambiguously established the absolute configurations of **10** and **11** as 2*S*, 4*R*, 5*R*, 6*R*, 7*S*, 8*S*, 9*R*, and 10*S* (Fig. 6).

Considering similar CD data of **8–11** and **13–14** resulted in the conclusion of the absolute configurations of **8–9** and **13–14** as 2*S*, 4*R*, 5*R*, 6*R*, 7*S*, 8*S*, 9*R*, and 10*S* (supplementary information Fig. C4). Thus, the structures of new compounds **8–9** and known compounds **10–14** were defined as shown and named (2*S*, 5*R*)-isocardivarolide A (**8**), (2*S*, 5*R*)-isocardivarolide E (**9**), (2*S*, 5*R*, 2″*R*)-ineupatolide (**10**), (2*S*, 5*R*, 2″*S*)-ineupatolide (**11**), ent-divaricin B (**12**), (2*S*, 5*R*)-isocardivarolide B (**13**), and (2*S*,5*R*)-isocardivarolide C (**14**), respectively.

In addition, compounds **10** and **11** were usually reported as a mixture from *C. triste*^25,26^. Although both of them were separated successfully in the literature^14^, the NMR data have not been reported. Herein, the NMR data of **10** and **11** were reported for the first time. The MS, ^1^H NMR, and ^13^CNMR spectroscopic data of compound **12** were consistent or superposable with those of divaricin B^8,19,20,26^. These data indicated that both of them shared the same relative configurations. However, all isolated compounds **1–11** and **13–14** had negative optical rotation, but divaricin B has the opposite optical rotation (${[{\rm{\alpha }}]}_{{\rm{D}}}^{20}$ − 15.6 of **12** and ${[{\rm{\alpha }}]}_{{\rm{D}}}^{20}$ + 18 of divaricin B^19^), which suggested that divaricin B and compound **12** could be enantiomers and have the opposite absolute configuration. Herein, in order to distinguish two compounds, compound **12** was named ent-divaricin B. The MS, NMR, CD and optical rotation data of **12** was also reported in the paper.

Similarly, there is confusion in the literature about this class of compounds (subtype II) due to the incorrect depiction of the epoxy bonds. Thus, the structures of germacranolides of this type (subtype II) were depicted correctly in this paper.

### Structural Elucidation of Compounds from Subtype IV

The molecular formula of compound **15** was assigned as C_25_H_34_O_9_ by positive-ion HRESIMS ion at *m/z* 501.2101 [M + Na]^+^, the same as those of **6** and **12**. However, the ^1^H and ^13^C NMR data implied that the structure of **15** was similar to that of **17**^22,25^ (supplementary information S24), except that two isobutyryloxy groups of **17** were replaced by two angeloyloxy groups in **15**, which was further confirmed by the ^1^H-^1^H COSY, HSQC, and HMBC spectra (Fig. 3). The relative configuration of **15** was determined by analysis of ROESY data. The key NOE correlations of H-8/H-6, H-7/H-5, H-5/H_3_-15, H-7/H-9, and H-9/H-10 indicated that **15** had the same relative configuration as **17** (Fig. 4).

Compound **16** was the isomers of **2**, **7**, **10**, **11**, **13**, **18** and **19**, based on its HRESIMS (*m/z* 503.2263 [M + Na]^+^, C_25_H_36_O_9_Na). The ^1^H and ^13^C NMR data were similar to those of **17**, except for an angeloyloxy group at C-6 and the 3-methylbutyryloxy groups at c-9 in **16** instead of two isobutyryloxy groups in **17**. The conclusion was confirmed by analysis of relevant ^1^H-^1^H COSY, HSQC and HMBC data. The relative configuration of **16** was determined to be the same as that of **15** by comparison of their ROESY data.

Compounds **17**–**20** were identified as incaspitolide D (**17**)^23,27^, 4*β*,8*α*-dihydroxy-5*β*-angeloyloxy-9*β*-2-methylbutyryloxy-3-oxo-germacran-6*α*,12-olide (**18**)^17^, 4*β*,8*α*-dihydroxy-5*β*-isobutyryloxy-9*β*-3-methylbutyryloxy-3-oxo-germacran-6*α*,12-olide (**19**)^22^, and 4*β*,8*α*-dihydroxy-5*β*-angeloyloxy-9*β*-isobutyryloxy-3-oxo-germacran-6*α*,12-olide (**20**)^28^, by comparison of their MS, ^1^H NMR, and ^13^CNMR spectroscopic data, as well as optical rotation data with reported data. However, their absolute configurations have not been reported.

**Figure 7:**
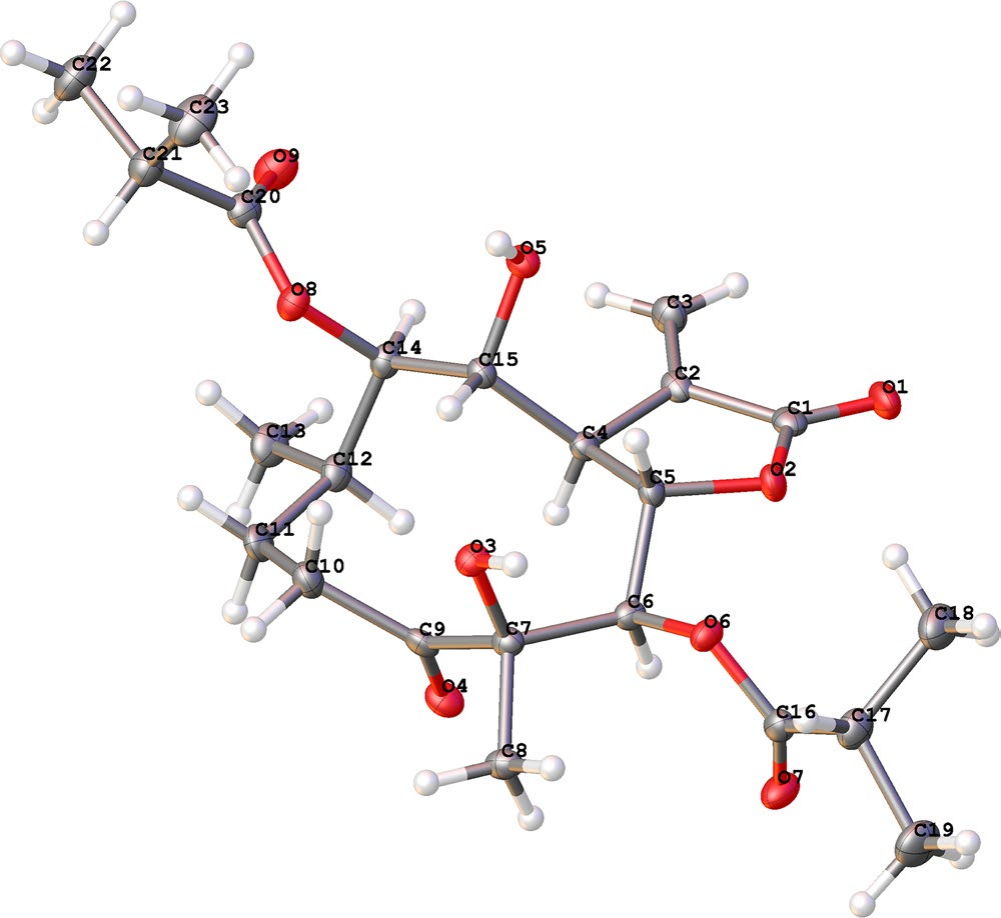
X-ray ORTEP drawing of **17**.

Therefore, the absolute configuration of **17** was determined to be 4*R*, 5*R*, 6*S*, 7*R*, 8*R*, 9*R*, and 10*R* by X-ray crystallographic analysis [flack parameter: −0.00(11)] (Fig. 7). Similar CD data of **15**–**17** and **18**–**20** assigned the absolute configurations of **15**–**16** and **18**–**20** as 4*R*, 5*R*, 6*S*, 7*R*, 8*R*, 9*R*, and 10*R* (supplementary information Fig. C5). Thus, the structures of new compounds **15–16** (named cardivarolide F and cardivarolide G, respectively) and the known compounds **18–20** were elucidated as shown.

All compounds were evaluated for their cytotoxic activity against human cervical (HeLa), colon (LoVo), stomach (BGC-823), and breast cancer (MCF-7) cell lines. Compounds **13**, **17**, and **18** exhibited cytotoxicity against HeLa (IC_50_ values of 7.60, 5.76, and 4.72* μ*M), LoVo (IC_50_ values of 7.81, 8.00, and 7.31* μ*M), and BGC-823 (IC_50_ values of 12.68, 13.68, and 11.67 *μ*M) cell lines, and the IC_50_ values were lower than that of the positive control *cis*-platin (IC_50_ values of 7.90, 13.03, and 15.34 *μ*M, respectively).

In conclusion, five sets of germacrane isomers (**1**/**8/17**, **2**/**7/10/11/13/16/18**, **3**/**4/5/14/20**, **6**/**12/15**, and **9**/**19**) representing three subtypes of germacranolides (subtypes I–II and IV) were isolated from the whole plant of *C. divaricatum*. The isolated germacrane isomers, including seven new ones (**1**–**3**, **8**–**9**, and **15**–**16**), contained a 5-membered γ-lactone ring fused to a circular 10-membered carbocycle. Subtypes I and II have the same planar structure, but the absolute configurations at C-2 and C-5 are different (2*R*, 5*S* in subtype I and 2*S*, 5*R* in subtype II). We obtained six pairs of stereoisomers (**1**/**8**, **2**/**13**, **4**/**14**, **6**/**12**, **7**/**11** and **10/11**) from the same plant. The isolation of these stereoisomers is a huge challenge because they are highly oxygenated and have similar structures.

The absolute configurations of compounds **4**, **10**, **11**, and **17** were unambiguously established by X-ray crystallographic analyses. The other compounds with the same skeleton were determined by comparison of NOESY and CD data with those of **4**, **10**, **11**, and **17**. Our findings have clarified the confusion in the literature about subtypes I and II of germacranolides. Compounds **13**, **17**, and **18** showed significant cytotoxicity against three human tumor cell lines. These findings are an important addition to the present knowledge on the structurally diverse and biologically important germacranolide family.

## Methods

### General Experimental Procedures

Optical rotations were measured on a Perkin-Elmer 241 polarimeter (Perkin-Elmer, Waltham, MA, USA) and UV spectra were recorded on Shimadzu UV-2501 PC (Shimadzu, Kyoto, Japan). IR data were recorded using a Shimadzu FTIR-8400S spectrophotometer (Shimadzu, Kyoto, Japan). ^1^H and ^13^C-NMR data were acquired with Bruker 600 and Bruker 500 instruments (Bruker, Rheinstetten, Germany) using the solvent signals (CD_3_OD: *δ*_H_ 3.31/*δ*_C_ 49.0 ppm;) as references. HRESIMS data were acquired using Q-TOF analyzer in SYNAPT HDMS system (Waters, Milford, MA, USA). CD spectra were recorded on a JASCO J-815 Spectropolarimeter (Jasco, Tokyo, Japan). X-ray diffraction data were collected on the Agilent GEMINI^TM^E instrument (CrysAlisPro software, Version 1.171.35.11; Agilent, Santa Clara, CA, USA). HPLC was performed using Waters 2535 system (Waters, Milford, MA, USA) with the following components: preparative column, a Daisogel-C_18_-100A (10 *μ*m, 30 × 250 mm, ChuangXinTongHeng Sci.&Tech., Beijing, China) and a YMC-Pack ODS-A column (5 *μ*m, 10 × 250 mm, YMC, Kyoto, Japan); and detector, Waters 2489 UV. Sephadex LH-20 (40–70 *μ*m, Pharmacia Biotech AB, Uppsala, Sweden), silica gel (60–100, 100–200, and 200–300 mesh) and silica gel GF254 sheets (0.20–0.25 mm) (Qingdao Marine Chemical Plant, Qingdao, China) were used for column chromatography and TLC, respectively. TLC spots were visualized under UV light and by dipping into 5% H_2_SO_4_ in EtOH followed by heating.

### Plant Material

The whole plants of *C. divaricatum* were collected from EnShi, Hubei province of China, in August of 2013. They were identified by Prof. Ben-Gang Zhang of Institute of Medicinal Plant Development. A voucher specimen (No. 20130828) was deposited in the National Compound Library of Traditional Chinese Medicines, Institute of Medicinal Plant Development, Chinese Academy of Medical Sciences and Peking Union Medical College (CAMS & PUMC), China.

### Extraction and Isolation

The air-dried plants (9 kg) were extracted three times (7 days each time) with EtOH–H_2_O (95:5) at room temperature. The combined extract was concentrated under reduced pressure to furnish a dark brown residue (570 g), which was suspended in H_2_O and partitioned in turn with petroleum ether (bp 60–90 °C), EtOAc, and *n*-BuOH. The EtOAc extract (207 g) was separated chromatographically on silica gel column (60–100 mesh, 16 × 20 cm) with a gradient mixture of CH_2_Cl_2_–MeOH (100:1, 60:1, 30:1, 15:1, and 6:1) as eluent. Five fractions were collected according to TLC analysis. Fraction A (CH_2_Cl_2_–MeOH, 100:1, 140 g) was separated by silica gel column chromatography (CC) (100–200 mesh, 16 × 20 cm) with petroleum ether–Aceton (50:1, 25:1, 20:1, 15:1, 12:1, 10:1, 7:1, 5:1, 3:1, and 1:1) as eluent to give fractions A_1_–A_11_. Fraction A_10_ (petroleum ether–Aceton, 3:1, 40 g) was separated by Sephadex LH-20 CC (5 × 200 cm, MeOH) to give Fr.A_10_S_1_–Fr.A_10_S_3._ Fraction A_10_S_2_ (20 g) was then subjected to MCI gel CC (6 × 50 cm) with a gradient mixture of MeOH–H_2_O (60:40, 80:20, and 100:0, 4000 mL each) to give three fractions (Fr.A_10_S_2_M_1_–Fr.A_10_S_2_M_3_).

Fraction A_10_S_2_M_2_ (MeOH–H_2_O, 80:20, 3 g) was purified using preparative HPLC (Daisogel–C_18_–100 A, 10μm; 250 × 30 mm; 20 mL/min, 60% MeOH in H_2_O) to yield **1** (30 mg). Fraction A_10_S_2_M_2_ (13 g) was further separated chromatographically on silica gel column (200–300 mesh, 5 × 50 cm) with a gradient mixture of CH_2_Cl_2_– MeOH (150:1, 100:1, 50:1, and 20:1) as eluent, and a total of 86 fractions (Fr.A_10_S_2_M_2_-1–86, 200 mL each) were collected. Fraction A_10_S_2_M_2_-56–60 were recrysted with CH_2_Cl_2_–MeOH (10:1) to yield **17** (200 mg). Fraction A_10_S_2_M_2_-70 (100 mg) was purified using semipreparative HPLC (YMC–Pack ODS–A column; 5μm; 250 × 10 mm; 2 mL/min, 50% MeOH in H_2_O) to yield **13** (20 mg) and **14** (30 mg). Fraction A_10_S_2_M_2_-69 (100 mg) was purified using semipreparative HPLC with MeOH–H_2_O (50:50) to yield **10** (10 mg), **11** (9 mg), and a mixture of **9** and **12** (25 mg). The mixture of **9** and **12** (25 mg) was further purified using semipreparative HPLC (40–80% MeCN in H_2_O for 40 min) to yield **9** (4.5 mg) and **12** (6.2 mg). Fraction A_10_S_2_M_2_-15–19 (140 mg) were purified using semipreparative HPLC (40–60% MeOH in H_2_O for 20 min, and followed by 60–80% for 20 min) to yield **6** (10 mg) and **7** (50 mg). Fraction A_10_S_2_M_2_-20–24 (2 g) were separated by preparative HPLC (65% MeOH in H_2_O) and semipreparative HPLC (60% MeOH in H_2_O for 10 min, and followed by 60–90% for 25 min; 40–85% MeCN in H_2_O for 40 min) to yield **5** (6.8 mg), **3** (4 mg), **15** (5 mg), and **18** (12 mg). Fraction A_10_S_2_M_2_-34–50 (1.5 g) were separated by preparative HPLC (70% MeOH in H_2_O) and semipreparative HPLC (52–75% MeOH in H_2_O for 25 min, and followed by 75–95% for 10 min) to yield **4** (50 mg). Fraction A_10_S_2_M_2_-74–79 (140 mg) were purified using semipreparative HPLC (60–80% MeOH in H_2_O for 25 min, and followed by 80–90% for 20 min; 30–70% MeCN in H_2_O for 40 min) and to yield **8** (4 mg) and **19** (35 mg).

Fraction A_9_ (petroleum ether-Aceton, 5:1, 30 g) was separated by Sephadex LH-20 CC (5 × 200 cm, MeOH) to give Fr.A_9_S_1_–Fr.A_9_S_3._ Fraction A_9_S_2_ (20 g) was then subjected to MCI gel CC (6 × 50 cm) with a gradient mixture of MeOH–H_2_O (60:40, 80:20, and 100:0, 4000 mL each) to give three fractions (Fr.A_9_S_2_M_1_–Fr.A_9_S_2_M_3_). Fraction A_9_S_2_M_2_ (10 g) was further separated chromatographically on silica gel column (100–200 mesh, 5 × 50 cm)with a gradient mixture of petroleum ether–Aceton (10:1, 7:1, 5:1, 3.5:1, 2:1, and 1:1) as eluent, and a total of 200 fractions (Fr.A_9_S_2_M_2_-1–200, 50 mL each) were collected. Fraction A_9_S_2_M_2_-113–123 (1 g) were separated by preparative HPLC (65% MeOH in H_2_O) and semipreparative HPLC (68% MeOH in H_2_O for 50 min; 40–80% MeCN in H_2_O for 40 min) to yield **2** (4.6 mg). Fraction A_9_S_2_M_2_-107–112 (2.5 g) were separated by silica gel column chromatography (CC) (200–300 mesh, 5 × 40 cm) with CH_2_Cl_2_–MeOH (150:1, 75:1, 30:1, and 15:1) as eluent to give Fr. A_9_S_2_M_2_-107-112-A_1_–Fr. A_9_S_2_M_2_-107-112-A_8_. Fraction A_9_S_2_M_2_-107–112–A_3_ (CH_2_Cl_2_–MeOH, 75: 1, 500 mg) was further purified using semipreparative HPLC(65–90% MeOH in H_2_O for 40 min; 40–80% MeCN in H_2_O for 40 min) to yield **16** (10 mg) and **20** (10 mg).

### X-ray Crystal Structure Analysis

X-ray diffraction data were collected on the Agilent GEMINI^TM^E instrument (CrysAlisPro software, Version 1.171.35.11), with enhanced Cu Kα radiation (λ = 1.54184 Å). The structure was solved by direct methods and refined by full-matrix least-squares techniques (SHELXL-97). All non-hydrogen atoms were refined with anisotropic thermal parameters. Hydrogen atoms were located by geometrical calculations and from positions in the electron density maps. Crystallographic data (excluding structure factors) for **4**, **10**, **11**, and **17** in this paper have been deposited with the Cambridge Crystallographic Data Centre (deposition numbers CCDC 1407813, 1407814, and 1407812). Copies of the data can be obtained, free of charge, on application to CCDC, 12 Union Road, Cambridge CB2 1EZ, UK (fax: +44 12 23336033 or e-mail: deposit@ccdc.cam.ac.uk).

A colorless orthorhombic crystal (0.58 × 0.48 × 0.45 mm) of **4** was obtained from MeOH. Crystal data were C_24_H_34_O_9_, *M* = 466.51, *T* = 99.5 K, orthorhombic, space group *P*2_1_2_1_2_1_, a = 9.05085(16) Å, b = 14.4036(3) Å, c = 19.1015(3) Å, *α* = 90.00°, *β* = 90.00°, *γ* = 90.00°, *V* = 2490.16(8) Å^3^, *Z* = 4, *ρ* = 1.244 mg/mm^3^, *μ*(Cu Kα) = 0.790 mm^−1^, measured reflections = 8581, unique reflections = 4706 (R_int_ = 0.0189), largest difference peak/hole = 0.247/ −0.197 e Å^−3^, and flack parameter = −0.10(12). The final R indexes [*I* > 2*σ* (*I*)] were R_1_ = 0.0338, and wR_2_ = 0.0884. The final R indexes (all data) were R_1_ = 0.0345, and wR_2_ = 0.0890. The goodness of fit on F^2^ was 1.055.

After trying several solvent systems, the mixture trigonal crystal (0.50 × 0.20 × 0.20 mm) of **10** and **11** (1:1) was from MeOH. Main parameters: C_25.487355_H_27_O_10.25_, *M* = 497.32, *T* = 103.1 K, trigonal, space group *P*3_2_2_1_, a = 18.3947(4) Å, b = 18.3947(4) Å, c = 28.7027(5) Å, *α* = 90.00°, *β* = 90.00°, *γ* = 120.00°, *V* = 8410.8(3) Å^3^, *Z* = 12, *ρ* = 1.178 mg/mm^3^, *μ*(Cu Kα) = 0.774 mm^−1^, measured reflections = 56790, unique reflections = 10785 (R_int_ = 0.0359), largest difference peak/hole = 0.857/−0.560 e Å^−3^, and flack parameter = −0.0(2). The final R indexes [*I* > 2*σ* (*I*)] were R_1_ = 0.0668, and wR_2_ = 0.1781. The final R indexes (all data) were R_1_ = 0.0737, and wR_2_ = 0.1857. The goodness of fit on F^2^ was 1.027.

A colorless monoclinic crystal (0.55 × 0.40 × 0.36 mm) of **17** was grown from CH_2_Cl_2_-MeOH (20:1). Crystal data: C_23_H_34_O_9_, *M* = 454.50, *T* = 101.0 K, monoclinic, space group *P*2_1_, a = 13.5135(4) Å, b = 9.5039(2) Å, c = 18.8280(6) Å, *α* = 90.00°, *β* = 105.051(3)°, *γ* = 90.00°, *V* = 2335.13(11) Å^3^, *Z* = 4, *ρ* = 1.293 mg/mm^3^, *μ*(Cu Kα) = 0.827 mm^−1^, measured reflections = 16917, unique reflections = 8859 (R_int_ = 0.0244), largest difference peak/hole = 0.759/−0.445 e Å^−3^, and flack parameter = −0.00(11). The final R indexes [*I* > 2*σ* (*I*)] were R_1_ = 0.0390, and wR_2_ = 0.1006. The final R indexes (all data) were R_1_ = 0.0397, and wR_2_ = 0.1012. The goodness of fit on F^2^ was 1.024.

### Cytotoxicity Assays

The assay was run in triplicate. In a 96-well plate, each well was plated with 2 × 10^4^ cells. After cell attachment overnight, the medium was removed, and each well was treated with 100 *μ*L of medium containing 0.1% DMSO or different concentrations of the test compounds and the positive control *cis*-platin. The plate was incubated for 4 days at 37 °C in a humidified, 5% CO_2_ atmosphere. Cytotoxicity was determined using a modified 3-(4,5-dimethylthiazol-2-yl)-2,5-diphenyltetrazolium bromide (MTT) colorimetric assay^29^. After addition of 10 *μ*L MTT solution (5 mg/mL), cells were incubated at 37 °C for 4 h. After adding 150 *μ*L DMSO, cells were shaken to mix thoroughly. The absorbance of each well was measured at 540 nm in a Multiscan photometer. The IC_50_ values were calculated by Origin software and listed in Table 3.

**Table 3:** Cytotoxicity of Compounds **13**, **17** and **18**.

compounds	IC_50_ (*μ*M)			
	HeLa	LoVo	BGC-823	MCF-7
**13**	7.60 ± 0.54	7.81 ± 0.25	12.68 ± 0.42	>50
**17**	5.76 ± 0.74	8.00 ± 0.20	13.68 ± 0.63	>50
**18**	4.72 ± 0.13	7.31 ± 0.21	11.67 ± 0.25	>50
*cis*-platin	7.90 ± 0.23	13.03 ± 1.49	15.34 ± 0.35	16.38 ± 1.41

Values were mean ± SD.

*Cis*-platin, positive control.

Cell lines: HeLa: cervical cancer, LoVo: colon cancer, BGC-823: stomach cancer, and MCF-7: breast cancer.

## Electronic supplementary material

Supplementary Information
