# Supplementary material for: Isolation, Structure Elucidation, and Absolute Configuration of Germacrane Isomers from Carpesium divaricatum
Source: Sci Rep. 2018 Aug 20;8:12418. doi: 10.1038/s41598-018-30782-2 (PMC6102216; doi:10.1038/s41598-018-30782-2)
Supplement: Supplementary file 1 — Supplementary Information [file 41598_2018_30782_MOESM1_ESM.pdf]

Supplementary Information

Isolation, Structure Elucidation, and Absolute Configuration of Germacrane Isomers from *Carpesium divaricatum*

Tao Zhang,[1] Jia-Huan Chen,[1, 2] Jin-Guang Si,[1] Qiu-Bo Zhang,[1] Gang Ding,[1] Hong-Wu Zhang,[1] Hong-Mei Jia,[1] and Zhong-Mei Zou[1*]

[1] Institute of Medicinal Plant Development, Chinese Academy of Medical Sciences and Peking Union Medical College, Beijing 100193, P. R. China.

[2] School of Traditional Chinese Medicine, Shenyang Pharmaceutical University, Shenyang 110016, P. R. China

*Correspondence and requests for materials should be addressed to Zhong-Mei Zou (Email: zmzou@implad.ac.cn; Tel: +86-010-57833290; Fax: +86-010-57833290)

Contents of Supplementary Information

	contents
T1	Characterization of compounds **1–16**
Fig.C1	CD spectra of compounds **1–7**
C2	The relevant data of ECD calculations
Fig.C3	The simulated distances of H-4/H-7 and H3-15/H-6 by MOE software
Fig.C4	CD spectra of compounds **8–14**
Fig.C5	CD spectra of compounds **15–20**
Fig. S1.1	^{1}H NMR spectrum (600 MHz) of (2*R*, 5*S*)-cardivarolide A (**1**) in CD_3OD
Fig. S1.2	^{13}C NMR spectrum (150 MHz) of (2*R*, 5*S*)-cardivarolide A (**1**) in CD_3OD
Fig. S1.3	^{1}H-^{1}H COSY spectrum (600 MHz) of (2*R*, 5*S*)-cardivarolide A (**1**) in CD_3OD
Fig. S1.4	HSQC spectrum (600 MHz) of (2*R*, 5*S*)-cardivarolide A (**1**) in CD_3OD
Fig. S1.5	HMBC spectrum (600 MHz) of (2*R*, 5*S*)-cardivarolide A (**1**) in CD_3OD
Fig. S1.6	NOESY spectrum (600 MHz) of (2*R*, 5*S*)-cardivarolide A (**1**) in CD_3OD
Fig. S1.7	UV spectrum of (2*R*, 5*S*)-cardivarolide A (**1**)
Fig. S1.8	IR spectrum of (2*R*, 5*S*)-cardivarolide A (**1**)
Fig. S1.9	HRESIMS spectrum of (2*R*, 5*S*)-cardivarolide A (**1**)
Fig. S1.10	CD spectrum of (2*R*, 5*S*)-cardivarolide A (**1**)
Fig. S2.1	^{1}H NMR spectrum (600 MHz) of (2*R*, 5*S*)-cardivarolide B (**2**) in CD_3OD
Fig. S2.2	^{13}C NMR spectrum (150 MHz) of (2*R*, 5*S*)-cardivarolide B (**2**) in CD_3OD
Fig. S2.3	^{1}H-^{1}H COSY spectrum (600 MHz) of (2*R*, 5*S*)-cardivarolide B (**2**) in CD_3OD
Fig. S2.4	HSQC spectrum (600 MHz) of (2*R*, 5*S*)-cardivarolide B (**2**) in CD_3OD
Fig. S2.5	HMBC spectrum (600 MHz) of (2*R*, 5*S*)-cardivarolide B (**2**) in CD_3OD
Fig. S2.6	NOESY spectrum (600 MHz) of (2*R*, 5*S*)-cardivarolide B (**2**) in CD_3OD
Fig. S2.7	UV spectrum of (2*R*, 5*S*)-cardivarolide B (**2**)
Fig. S2.8	IR spectrum of (2*R*, 5*S*)-cardivarolide B (**2**)
Fig. S2.9	HRESIMS spectrum of (2*R*, 5*S*)-cardivarolide B (**2**)
Fig. S2.10	CD spectrum of (2*R*, 5*S*)-cardivarolide B (**2**)
Fig. S3.1	^{1}H NMR spectrum (500 MHz) of (2*R*, 5*S*)-ciscardivarolide C (**3**) in CD_3OD
Fig. S3.2	^{13}C NMR spectrum (125 MHz) of (2*R*, 5*S*)-ciscardivarolide C (**3**) in CD_3OD
Fig. S3.3	^{1}H-^{1}H COSY spectrum (500 MHz) of (2*R*, 5*S*)-ciscardivarolide C (**3**) in CD_3OD
Fig. S3.4	HSQC spectrum (500 MHz) of (2*R*, 5*S*)-ciscardivarolide C (**3**) in CD_3OD
Fig. S3.5	HMBC spectrum (500 MHz) of (2*R*, 5*S*)-ciscardivarolide C (**3**) in CD_3OD
Fig. S3.6	ROESY spectrum (500 MHz) of (2*R*, 5*S*)-ciscardivarolide C (**3**) in CD_3OD
Fig. S3.7	UV spectrum of (2*R*, 5*S*)-ciscardivarolide C (**3**)
Fig. S3.8	IR spectrum of (2*R*, 5*S*)-ciscardivarolide C (**3**)
Fig. S3.9	HRESIMS spectrum of (2*R*, 5*S*)-ciscardivarolide C (**3**)
Fig. S3.10	CD spectrum of (2*R*, 5*S*)-ciscardivarolide C (**3**)
Fig. S4.1	^{1}H NMR spectrum (500 MHz) of (2*R*, 5*S*)-cardivarolide C (**4**) in $CDCl_3$
Fig. S4.2	^{13}C NMR spectrum (125 MHz) of (2*R*, 5*S*)-cardivarolide C (**4**) in $CDCl_3$
Fig. S4.3	^{1}H-^{1}H COSY spectrum (500 MHz) of (2*R*, 5*S*)-cardivarolide C (**4**) in $CDCl_3$
Fig. S4.4	HSQC spectrum (500 MHz) of (2*R*, 5*S*)-cardivarolide C (**4**) in $CDCl_3$

Fig. S4.5	HMBC spectrum (500 MHz) of (2*R*, 5*S*)-cardivarolide C (**4**) in $CDCl_3$
Fig. S4.6	ROESY spectrum (500 MHz) of (2*R*, 5*S*)-cardivarolide C (**4**) in $CDCl_3$
Fig. S4.7	^{1}H NMR spectrum (600 MHz) of (2*R*, 5*S*)-cardivarolide C (**4**) in CD_3OD
Fig. S4.8	^{13}C NMR spectrum (600 MHz) of (2*R*, 5S)-cardivarolide C (**4**) in CD_3OD
Fig. S4.9	NOESY spectrum (600 MHz) of (2*R*, 5*S*)-cardivarolide C (**4**) in CD_3OD
Fig. S4.10	UV spectrum of (2*R*, 5*S*)-cardivarolide C (**4**)
Fig. S4.11	IR spectrum of (2*R*, 5*S*)-cardivarolide C (**4**)
Fig. S4.12	HRESIMS spectrum of (2*R*, 5*S*)-cardivarolide C (**4**)
Fig. S4.13	CD spectrum of (2*R*, 5*S*)-cardivarolide C (**4**)
Fig. S5.1	^{1}H NMR spectrum (500 MHz) of ineupatolide A (**5**) in CD_3OD
Fig. S5.2	^{13}C NMR spectrum (125 MHz) of ineupatolide A (**5**) in CD_3OD
Fig. S5.3	^{1}H-^{1}H COSY spectrum (500 MHz) of ineupatolide A (**5**) in CD_3OD
Fig. S5.4	HSQC spectrum (500 MHz) of ineupatolide A (**5**) in CD_3OD
Fig. S5.5	HMBC spectrum (500 MHz) of ineupatolide A (**5**) in CD_3OD
Fig. S5.6	NOESYspectrum (600 MHz) of ineupatolide A (**5**) in CD_3OD
Fig. S5.7	UV spectrum of ineupatolide A (**5**)
Fig. S5.8	IR spectrum of ineupatolide A (**5**)
Fig. S5.9	HRESIMS spectrum of ineupatolide A (**5**)
Fig. S5.10	CD spectrum of ineupatolide A (**5**)
Fig. S6.1	^{1}H NMR spectrum (600 MHz) of (2*R*, 5*S*)-cardivarolide D (**6**) in CD_3OD
Fig. S6.2	^{13}C NMR spectrum (150 MHz) of (2*R*, 5*S*)-cardivarolide D (**6**) in CD_3OD
Fig. S6.3	^{1}H-^{1}H COSY spectrum (600 MHz) of (2*R*, 5*S*)-cardivarolide D (**6**) in CD_3OD
Fig. S6.4	HSQC spectrum (600 MHz) of (2*R*, 5*S*)-cardivarolide D (**6**) in CD_3OD
Fig. S6.5	HMBC spectrum (600 MHz) of (2*R*, 5*S*)-cardivarolide D (**6**) in CD_3OD
Fig. S6.6	NOESY spectrum (600 MHz) of (2*R*, 5*S*)-cardivarolide D (**6**) in CD_3OD
Fig. S6.7	UV spectrum of (2*R*, 5*S*)-cardivarolide D (**6**)
Fig. S6.8	IR spectrum of (2*R*, 5*S*)-cardivarolide D (**6**)
Fig. S6.9	HRESIMS spectrum of (2*R*, 5*S*)-cardivarolide D (**6**)
Fig. S6.10	CD spectrum of (2*R*, 5*S*)-cardivarolide D (**6**)
Fig. S7.1	^{1}H NMR spectrum (600 MHz) of ineupatolide (**6**) in CD_3OD
Fig. S7.2	^{13}C NMR spectrum (150 MHz) of ineupatolide (**6**) in CD_3OD
Fig. S7.3	^{1}H-^{1}H COSY spectrum (500 MHz) of ineupatolide (**6**) in CD_3OD
Fig. S7.4	HSQC spectrum (500 MHz) of ineupatolide (**6**) in CD_3OD
Fig. S7.5	HMBC spectrum (500 MHz) of ineupatolide (**6**) in CD_3OD
Fig. S7.6	NOESYspectrum (600 MHz) of ineupatolide (**6**) in CD_3OD
Fig. S7.7	UV spectrum of ineupatolide (**6**)
Fig. S7.8	IR spectrum of ineupatolide (**6**)
Fig. S7.9	HRESIMS spectrum of ineupatolide (**6**)
Fig. S7.10	CD spectrum of ineupatolide (**6**)
Fig. S8.1	^{1}H NMR spectrum (600 MHz) of (2*S*, 5*R*)-isocardivarolide A (**8**) in CD_3OD
Fig. S8.2	^{13}C NMR spectrum (150 MHz) of (2*S*, 5*R*)-isocardivarolide A (**8**) in CD_3OD
Fig. S8.3	^{1}H-^{1}H COSY spectrum (600 MHz) of (2*S*, 5*R*)-isocardivarolide A (**8**) in CD_3OD
Fig. S8.4	HSQC spectrum (600 MHz) of (2*S*, 5*R*)-isocardivarolide A (**8**) in CD_3OD

Fig. S8.5	HMBC spectrum (600 MHz) of (2*S*, 5*R*)-isocardivarolide A (**8**) in CD_3OD
Fig. S8.6	NOESY spectrum (600 MHz) of (2*S*, 5*R*)-isocardivarolide A (**8**) in CD_3OD
Fig. S8.7	UV spectrum of (2*S*, 5*R*)-isocardivarolide A (**8**)
Fig. S8.8	IR spectrum of (2*S*, 5*R*)-isocardivarolide A (**8**)
Fig. S8.9	HRESIMS spectrum of (2*S*, 5*R*)-isocardivarolide A (**8**)
Fig. S8.10	CD spectrum of (2*S*, 5*R*)-isocardivarolide A (**8**)
Fig. S9.1	^{1}H NMR spectrum (500 MHz) of (2*S*, 5*R*)-isocardivarolide F (**9**) in CD_3OD
Fig. S9.2	^{13}C NMR spectrum (125 MHz) of (2*S*, 5*R*)-isocardivarolide F (**9**) in CD_3OD
Fig. S9.3	^{1}H-^{1}H COSY spectrum (500 MHz) of (2*S*, 5*R*)-isocardivarolide F (**9**) in CD_3OD
Fig. S9.4	HSQC spectrum (500 MHz) of (2*S*, 5*R*)-isocardivarolide F (**9**) in CD_3OD
Fig. S9.5	HMBC spectrum (500 MHz) of (2*S*, 5*R*)-isocardivarolide F (**9**) in CD_3OD
Fig. S9.6	ROESY spectrum (500 MHz) of (2*S*, 5*R*)-isocardivarolide F (**9**) in CD_3OD
Fig. S9.7	UV spectrum of (2*S*, 5*R*)-isocardivarolide F (**9**)
Fig. S9.8	IR spectrum of (2*S*, 5*R*)-isocardivarolide F (**9**)
Fig. S9.9	HRESIMS spectrum of (2*S*, 5*R*)-isocardivarolide F (**9**)
Fig. S9.10	CD spectrum of (2*S*, 5*R*)-isocardivarolide F (**9**)
Fig. S10.1	^{1}H NMR spectrum (600 MHz) of (2*S*, 5*R*, 2″*R*)-ineupatolide (**10**) in CD_3OD
Fig. S10.2	^{13}C NMR spectrum (150 MHz) of (2*S*, 5*R*, 2″*R*)-ineupatolide (**10**) in CD_3OD
Fig. S10.3	^{1}H-^{1}H COSY spectrum (600 MHz) of (2*S*, 5*R*, 2″*R*)-ineupatolide (**10**) in CD_3OD
Fig. S10.4	HSQC spectrum (600 MHz) of (2*S*, 5*R*, 2″*R*)-ineupatolide (**10**) in CD_3OD
Fig. S10.5	HMBC spectrum (600 MHz) of (2*S*, 5*R*, 2″*R*)-ineupatolide (**10**) in CD_3OD
Fig. S10.6	NOESY spectrum (600 MHz) of (2*S*, 5*R*, 2″*R*)-ineupatolide (**10**) in CD_3OD
Fig. S10.7	UV spectrum of (2*S*, 5*R*, 2″*R*)-ineupatolide (**10**)
Fig. S10.8	IR spectrum of (2*S*, 5*R*, 2″*R*)-ineupatolide (**10**)
Fig. S10.9	HRESIMS spectrum of (2*S*, 5*R*, 2″*R*)-ineupatolide (**10**)
Fig. S10.10	CD spectrum of (2*S*, 5*R*, 2″*R*)-ineupatolide (**10**)
Fig. S11.1	^{1}H NMR spectrum (600 MHz) of (2*S*, 5*R*, 2″*S*)-ineupatolide (**11**) in CD_3OD
Fig. S11.2	^{13}C NMR spectrum (150 MHz) of (2*S*, 5*R*, 2″*S*)-ineupatolide (**11**) in CD_3OD
Fig. S11.3	^{1}H-^{1}H COSY spectrum (600 MHz) of (2*S*, 5*R*, 2″*S*)-ineupatolide (**11**) in CD_3OD
Fig. S11.4	HSQC spectrum (600 MHz) of (2*S*, 5*R*, 2″*S*)-ineupatolide (**11**) in CD_3OD
Fig. S11.5	HMBC spectrum (600 MHz) of (2*S*, 5*R*, 2″*S*)-ineupatolide (**11**) in CD_3OD
Fig. S11.6	NOESY spectrum (600 MHz) of (2*S*, 5*R*, 2″*S*)-ineupatolide (**11**) in CD_3OD
Fig. S11.7	UV spectrum of (2*S*, 5*R*, 2″*S*)-ineupatolide (**11**)
Fig. S11.8	IR spectrum of (2*S*, 5*R*, 2″*S*)-ineupatolide (**11**)
Fig. S11.9	HRESIMS spectrum of (2*S*, 5*R*, 2″*S*)-ineupatolide (**11**)
Fig. S11.10	CD spectrum of (2*S*, 5*R*, 2″*S*)-ineupatolide (**11**)
Fig. S12.1	^{1}H NMR spectrum (500 MHz) of divaricin B (**12**) in CD_3OD
Fig. S12.2	^{13}C NMR spectrum (125 MHz) of divaricin B (**12**) in CD_3OD
Fig. S12.3	^{1}H-^{1}H COSY spectrum (500 MHz) of divaricin B (**12**) in CD_3OD
Fig. S12.4	HSQC spectrum (500 MHz) of divaricin B (**12**) in CD_3OD
Fig. S12.5	HMBC spectrum (500 MHz) of divaricin B (**12**) in CD_3OD
Fig. S12.6	ROESY spectrum (500 MHz) of divaricin B (**12**) in CD_3OD
Fig. S12.7	UV spectrum of divaricin B (**12**)

Fig. S12.8	IR spectrum of divaricin B (**12**)
Fig. S12.9	HRESIMS spectrum of divaricin B (**12**)
Fig. S12.10	CD spectrum of divaricin B (**12**)
Fig. S13.1	^{1}H NMR spectrum (600 MHz) of (2*S*, 5*R*)-isocardivarolide B (**13**) in CD_3OD
Fig. S13.2	^{13}C NMR spectrum (150 MHz) of (2*S*, 5*R*)-isocardivarolide B (**13**) in CD_3OD
Fig. S13.3	^{1}H-^{1}H COSY spectrum (600 MHz) of (2*S*, 5*R*)-isocardivarolide B (**13**) in CD_3OD
Fig. S13.4	HSQC spectrum (600 MHz) of (2*S*, 5*R*)-isocardivarolide B (**13**) in CD_3OD
Fig. S13.5	HMBC spectrum (600 MHz) of (2*S*, 5*R*)-isocardivarolide B (**13**) in CD_3OD
Fig. S13.6	NOESY spectrum (600 MHz) of (2*S*, 5*R*)-isocardivarolide B (**13**) in CD_3OD
Fig. S13.7	UV spectrum of (2*S*, 5*R*)-isocardivarolide B (**13**)
Fig. S13.8	IR spectrum of (2*S*, 5*R*)-isocardivarolide B (**13**)
Fig. S13.9	HRESIMS spectrum of (2*S*, 5*R*)-isocardivarolide B (**13**)
Fig. S13.10	CD spectrum of (2*S*, 5*R*)-isocardivarolide B (**13**)
Fig. S14.1	^{1}H NMR spectrum (600 MHz) of (2*S*, 5*R*)-isocardivarolide C (**14**) in CD_3OD
Fig. S14.2	^{13}C NMR spectrum (150 MHz) of (2*S*, 5*R*)-isocardivarolide C (**14**) in CD_3OD
Fig. S14.3	^{1}H-^{1}H COSY spectrum (600 MHz) of (2*S*, 5*R*)-isocardivarolide C (**14**) in CD_3OD
Fig. S14.4	HSQC spectrum (600 MHz) of (2*S*, 5*R*)-isocardivarolide C (**14**) in CD_3OD
Fig. S14.5	HMBC spectrum (600 MHz) of (2*S*, 5*R*)-isocardivarolide C (**14**) in CD_3OD
Fig. S14.6	NOESY spectrum (600 MHz) of (2*S*, 5*R*)-isocardivarolide C (**14**) in CD_3OD
Fig. S14.7	UV spectrum of (2*S*, 5*R*)-isocardivarolide C (**14**)
Fig. S14.8	IR spectrum of (2*S*, 5*R*)-isocardivarolide C (**14**)
Fig. S14.9	HRESIMS spectrum of (2*S*, 5*R*)-isocardivarolide C (**14**)
Fig. S14.10	CD spectrum of (2*S*, 5*R*)-isocardivarolide C (**14**)
Fig. S15.1	^{1}H NMR spectrum (500 MHz) of cardivarolide F (**15**) in CD_3OD
Fig. S15.2	^{13}C NMR spectrum (125 MHz) of cardivarolide F (**15**) in CD_3OD
Fig. S15.3	^{1}H-^{1}H COSY spectrum (500 MHz) of cardivarolide F (**15**) in CD_3OD
Fig. S15.4	HSQC spectrum (500 MHz) of cardivarolide F (**15**) in CD_3OD
Fig. S15.5	HMBC spectrum (500 MHz) of cardivarolide F (**15**) in CD_3OD
Fig. S15.6	ROESY spectrum (500 MHz) of cardivarolide F (**15**) in CD_3OD
Fig. S15.7	UV spectrum of cardivarolide F (**15**)
Fig. S15.8	IR spectrum of cardivarolide F (**15**)
Fig. S15.9	HRESIMS spectrum of cardivarolide F (**15**)
Fig. S15.10	CD spectrum of cardivarolide F (**15**)
Fig. S16.1	^{1}H NMR spectrum (500 MHz) of cardivarolide G (**16**) in CD_3OD
Fig. S16.2	^{13}C NMR spectrum (125 MHz) of cardivarolide G (**16**) in CD_3OD
Fig. S16.3	^{1}H-^{1}H COSY spectrum (500 MHz) of cardivarolide G (**16**) in CD_3OD
Fig. S16.4	HSQC spectrum (500 MHz) of cardivarolide G (**16**) in CD_3OD
Fig. S16.5	HMBC spectrum (500 MHz) of cardivarolide G (**16**) in CD_3OD
Fig. S16.6	ROESY spectrum (500 MHz) of cardivarolide G (**16**) in CD_3OD
Fig. S16.7	UV spectrum of cardivarolide G (**16**)
Fig. S16.8	IR spectrum of cardivarolide G (**16**)
Fig. S16.9	HRESIMS spectrum of cardivarolide G (**16**)
Fig. S16.10	CD spectrum of cardivarolide G (**16**)

Fig. S17	CD spectrum of incaspitolide D (**17**)
Fig. S18	CD spectrum of compound **18**
Fig. S19	CD spectrum of compound **19**
Fig. S20	CD spectrum of compound **20**
S21	X-ray data of (2*R*, 5*S*)-cardivarolide C (**4**)
S22	X-ray data of (2*S*, 5*R*, 2″*R*)-ineupatolide (**10**) and (2*S*, 5*R*, 2″*S*)-ineupatolide (**11**)
S23	X-ray data of incaspitolide D (**17**)
S24	^{1}H and ^{13}C NMR spectroscopic data of **17** in CD_3OD

*T1 Characterization of compounds **1–16***

(2*R*, 5*S*)-cardivarolide A (**1**): white needles (CH_3OH), $[\alpha]_D^{20}$ –39.3 (*c* 0.107, MeOH); mp 181–183 °C; UV (MeOH) λ_{max}(logε): 213 (3.66) nm, IR (KBr) ν_{max}: 3493, 3298, 1767, 1737 cm^{-1}; CD (MeOH) 206 (Δε +0.238), 253 (Δε +0.107), 226 (Δε -0.599) nm; HRESIMS (pos.): *m/z* 477.2104 $[M+Na]^+$ (calcd for $C_{23}H_{34}O_9Na$, 477.2101); ^1H NMR data, see Table 1, ^{13}C NMR data, see Table 2.

(2*R*, 5*S*)-cardivarolide B (**2**): white needles (CH_3OH), $[\alpha]_D^{20}$ –50.0 (*c* 0.120, MeOH); mp 189–191 °C; UV (MeOH) λ_{max}(logε): 200 (4.72) nm, IR (neat) ν_{max}: 3399, 1755, 1703, 1644 cm^{-1}; HRESIMS (pos.): *m/z* 503.2261 $[M+Na]^+$ (calcd for $C_{25}H_{36}O_9Na$, 503.2257); CD (MeOH) 221 (Δε +0.002), 266 (Δε +0.004), 295 (Δε +0.003), 237 (Δε -0.007) , 339 (Δε -0.003) nm; ^1H NMR data, see Table 1, ^{13}C NMR data, see Table 2.

(2*R*, 5*S*)-ciscardivarolide C (**3**): white needles (CH_3OH), $[\alpha]_D^{20}$ –37.0 (*c* 0.10, MeOH); UV (MeOH) λ_{max}(logε): 216 (3.74) nm, IR (neat) ν_{max}: 3455, 2973, 1754, 1650 cm^{-1}; CD (MeOH) 218 (Δε +0.076), 255 (Δε +0.019) nm; HRESIMS (pos.): *m/z* 489.2112 $[M+Na]^+$ (calcd for $C_{24}H_{34}O_9Na$,489.2101); ^1H NMR data, see Table 1, ^{13}C NMR data, see Table 2.

(2*R*, 5*S*)-cardivarolide C (**4**): white needles (CH_3OH), $[\alpha]_D^{20}$ –47.7 (*c* 0.107, MeOH); UV (MeOH) λ_{max}(logε): 217 (3.99) nm, IR (KBr) ν_{max}: 3503, 3390, 1772, 1690, 1654 cm^{-1}; CD (MeOH) 220 (Δε +0.159), 260 (Δε +0.013), 239 (Δε -0.047) nm; HRESIMS (pos.): *m/z* 489.2100 $[M+Na]^+$ (calcd for $C_{24}H_{34}O_9Na$,489.2101); ^1H NMR data, see Table 1, ^{13}C NMR data, see Table 2.

ineupatolide A (**5**): white needles (CH_3OH), $[\alpha]_D^{20}$ –48.8 (*c* 0.080, MeOH); UV

(MeOH) λ_{max}(logε): 215 (4.15) nm, IR (KBr) ν_{max}: 3505, 3388, 1772, 1690, 1654 cm^{-1}; CD (MeOH) 255 (Δε +0.020) nm; HRESIMS (pos.): *m/z* 489.2107 $[M+Na]^+$ (calcd for $C_{24}H_{34}O_9Na$,489.2101); ^1H NMR data, see Table 1, ^{13}C NMR data, see Table 2.

(2*R*, 5*S*)-cardivarolide D (**6**): white needles (CH_3OH), $[\alpha]_D^{20}$ –42.5 (*c* 0.113, MeOH); UV (MeOH) λ_{max}(logε): 200 (4.75) nm, IR (neat) ν_{max}: 3502, 3408, 1750, 1695, 1651 cm^{-1}; CD (MeOH) 227 (Δε +0.022), 267 (Δε +0.001), 248 (Δε -0.001) nm; HRESIMS (pos.): *m/z* 501.2107 $[M+Na]^+$ (calcd for $C_{25}H_{34}O_9Na$, 501.2101); ^1H NMR data, see Table 1, ^{13}C NMR data, see Table 2.

ineupatolide (**7**): white needles (CH_3OH), $[\alpha]_D^{20}$ –42.5 (*c* 0.080, MeOH); UV (MeOH) λ_{max}(logε): 215 (4.19) nm, IR (neat) ν_{max}: 3510, 3416, 1774, 1691 cm^{-1}; CD (MeOH) 220 (Δε +0.307), 266 (Δε +0.048), 243 (Δε -0.031) nm; HRESIMS (pos.): *m/z* 503.2266 $[M+Na]^+$ (calcd for $C_{25}H_{36}O_9Na$, 503.2257); ^1H NMR data, see Table 1, ^{13}C NMR data, see Table 2.

(2*S*, 5*R*)-isocardivarolide A (**8**): white needles (CH_3OH), $[\alpha]_D^{20}$ –7.00 (*c* 0.100, MeOH); mp 175–177 °C; UV (MeOH) λ_{max}(logε): 210 (3.61) nm, IR (neat) ν_{max}: 3392, 1652 cm^{-1}; CD (MeOH) 255 (Δε +0.015), 218 (Δε -0.068) nm; HRESIMS (pos.): *m/z* 477.2101 $[M+Na]^+$ (calcd for $C_{23}H_{34}O_9Na$, 477.2101); ^1H NMR data, see Table 1, ^{13}C NMR data, see Table 2.

(2*S*, 5*R*)-isocardivarolide F (**9**): white needles (CH_3OH), $[\alpha]_D^{20}$ –6.00 (*c* 0.150, MeOH); mp 178–180 °C; UV (MeOH) λ_{max}(logε): 200 (4.65) nm, IR (neat) ν_{max}: 3409, 1762, 1723, 1648 cm^{-1}; CD (MeOH) 254 (Δε +0.285), 216 (Δε -0.761) nm; HRESIMS (pos.): *m/z* 491.2271 $[M+Na]^+$ (calcd for $C_{24}H_{36}O_9Na$, 491.2257); ^1H

NMR data, see Table 1, ^{13}C NMR data, see Table 2.

(2*S*, 5*R*, 2″*R*)-ineupatolide (**10**): white needles (CH_3OH), $[\alpha]_D^{20}$ –10.0 (*c* 0.080, MeOH); UV (MeOH) λ_{max}(logε): 218 (4.03) nm, IR (neat) ν_{max}: 3449, 1759, 1720, 1642 cm^{-1}; CD (MeOH) 259 (Δε +0.044), 211 (Δε -0.756), 311 (Δε -0.009) nm; HRESIMS (pos.): *m/z* 481.2454 $[M+H]^+$ (calcd for $C_{25}H_{37}O_9$, 481.2438); ^{1}H NMR data, see Table 1, ^{13}C NMR data, see Table 2.

(2*S*, 5*R*, 2″*S*)-ineupatolide (**11**): white needles (CH_3OH), $[\alpha]_D^{20}$ –1.25 (*c* 0.080, MeOH); UV (MeOH) λ_{max}(logε): 213 (3.71) nm, IR (neat) ν_{max}: 3543, 3463, 1758 1722, 1649 cm^{-1}; CD (MeOH) 260 (Δε +0.014) nm; HRESIMS (pos.): *m/z* 481.2454 $[M+H]^+$ (calcd for $C_{25}H_{37}O_9$, 481.2438); ^{1}H NMR data, see Table 1, ^{13}C NMR data, see Table 2.

ent-divaricin B (**12**): white needles (CH_3OH), $[\alpha]_D^{20}$ –15.6 (*c* 0.090, MeOH); UV (MeOH) λ_{max}(logε): 217 (4.41) nm, IR (neat) ν_{max}: 3449, 1758, 1722, 1652 cm^{-1}; CD (MeOH) 260 (Δε +0.081), 231 (Δε +0.2) nm; HRESIMS (pos.): *m/z* 501.2109 $[M+Na]^+$ (calcd for $C_{25}H_{34}O_9Na$, 501.2101); ^{1}H NMR data, see Table 1, ^{13}C NMR data, see Table 2.

(2*S*, 5*R*)-isocardivarolide B (**13**): white needles (CH_3OH), $[\alpha]_D^{20}$ –11.0 (*c* 0.073, MeOH); UV (MeOH) λ_{max}(logε): 213 (4.00) nm, IR (KBr) ν_{max}: 3482, 1763, 1724, 1643 cm^{-1}; CD (MeOH) 258 (Δε +0.018) nm; HRESIMS (pos.): *m/z* 481.2457 $[M+H]^+$ (calcd for $C_{25}H_{37}O_9$, 481.2438); ^{1}H NMR data, see Table 1, ^{13}C NMR data, see Table 2.

(2*S*, 5*R*)-isocardivarolide C (**14**): white needles (CH_3OH), $[\alpha]_D^{20}$ –3.75 (*c* 0.080,

MeOH); UV (MeOH) λ_{max}(logε): 213 (3.79) nm, IR (KBr) ν_{max}: 3482, 1769, 1723, 1647 cm^{-1}; CD (MeOH) 255 (Δε +0.028) nm; HRESIMS (pos.): *m/z* 467.2298 $[M+H]^+$ (calcd for $C_{24}H_{35}O_9$, 467.2281); ^1H NMR data, see Table 1, ^{13}C NMR data, see Table 2.

Cardivarolide F (**15**): white needles (CH_3OH), $[\alpha]_D^{20}$ –84.7 (*c* 0.085, MeOH); mp 228–230 °C; UV (MeOH) λ_{max}(logε): 199 (4.88) nm, IR (neat) ν_{max}: 3443, 1757, 1718, 1690 cm^{-1}; CD (MeOH) 216 (Δε -0.025), 307 (Δε -0.005) nm; HRESIMS (pos.): *m/z* 501.2101 $[M+Na]^+$ (calcd for $C_{25}H_{34}O_9Na$, 501.2101); ^1H NMR data, see Table 1, ^{13}C NMR data, see Table 2.

Cardivarolide G (**16**): white needles (CH_3OH), $[\alpha]_D^{20}$ –90.0 (*c* 0.10, MeOH); mp 216–218 °C; UV (MeOH) λ_{max}(logε): 213 (3.87) nm, IR (KBr) ν_{max}: 3427, 1740, 1661 cm^{-1}; CD (MeOH) 212 (Δε -0.793), 308 (Δε -0.135) nm; HRESIMS (pos.): *m/z* 503.2257 $[M+Na]^+$ (calcd for $C_{25}H_{38}O_9Na$, 503.2263); ^1H NMR data, see Table 1, ^{13}C NMR data, see Table 2.

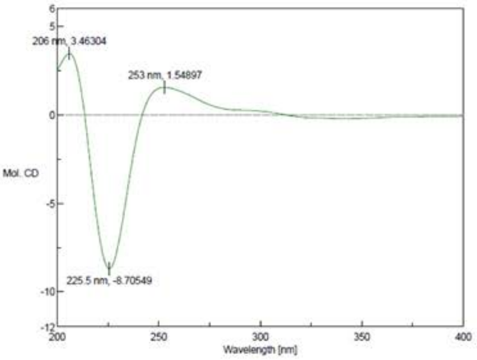

1

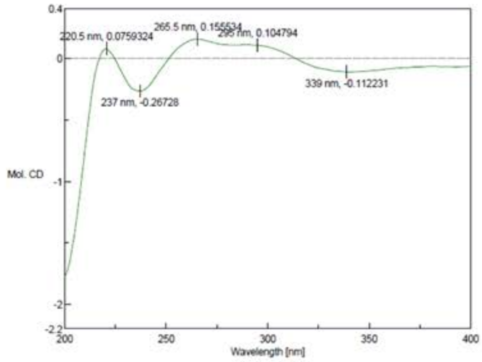

2

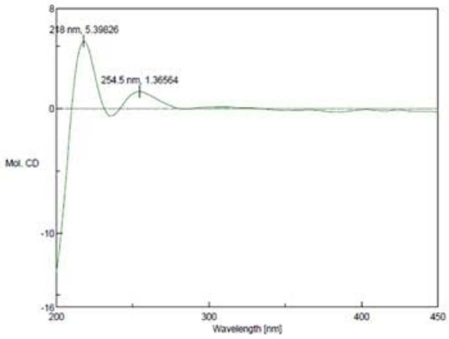

3

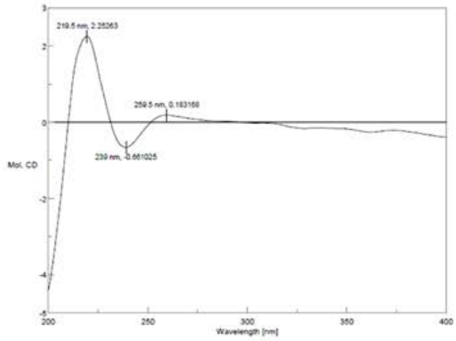

4

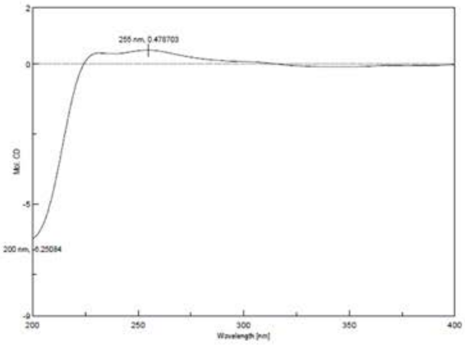

5

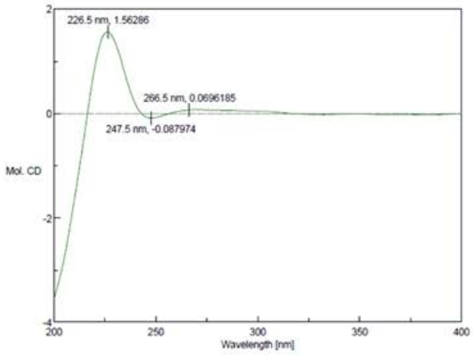

6

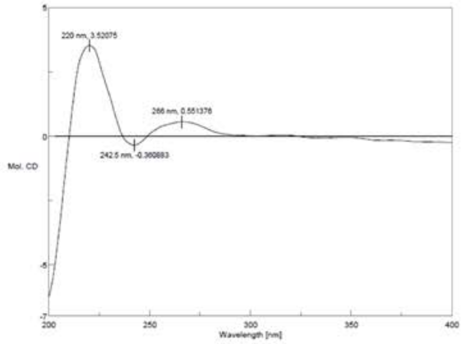

7

Fig. C1 CD spectra of compounds **1**-**7**

C2: The relevant data of ECD calculations

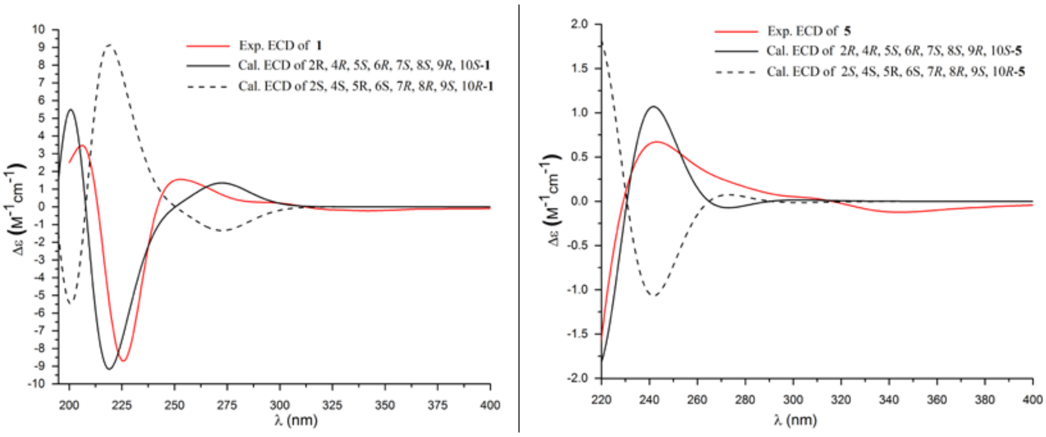

Fig. C2 The calculated ECD and experimental ECD spectra of **1** and **5**

Computational details

In general, conformational analyses were carried out via random searching in the Sybyl-X 2.0 using the MMFF94S force field with an energy cutoff of 2.5 kcal/mol.[1] Subsequently, the conformerswere re-optimized using DFT at the b3lyp/6-31+g(d) level in gasphase by the GAUSSIAN 09 program.[2] The energies, oscillator strengths, and rotational strengths (velocity) of the first 60 electronic excitations were calculated using the TDDFT methodology at the b3lyp/6-311++g(d,p) level in vacuum. The ECD spectra were simulated by the overlapping Gaussian function (half the bandwidth at 1/e peak height, σ = 0.3 for 1 and hjb-24, and 0.35 for hjb-10).[3] To get the final spectra, the simulated spectra ofthe conformers were average according to the Boltzmann distribution theory and their relative Gibbs free energy (ΔG).Theoretical ECD spectrum of the corresponding enantiomerwere obtained by directly inverse of the ECD spectrum of the above-mentioned compounds, respectively. By comparing the experiment spectrum with the calculated ECD spectra, the eight chiral centers of **1** and **5** were determined to be 2*R*, 4*R*, 5*S*, 6*R*, 7*S*, 8*S*, 9*R*, and 10*S*.

Reference:

(1) Sybyl Software, version X 2.0; Tripos Associates Inc.: St. Louis, MO, 2013.

(2) Frisch, M. J.; Trucks, G. W.; Schlegel, H. B.; Scuseria, G. E.; Robb, M. A.; Cheeseman, J. R.; Scalmani, G.; Barone, V.; Mennucci, B.; Petersson, G. A.; Nakatsuji, H.; Caricato, M.; Li, X.; Hratchian, H. P.; Izmaylov, A. F.; Bloino, J.; Zheng, G.; Sonnenberg, J. L.; Hada, M.; Ehara, M.; Toyota, K.; Fukuda, R.; Hasegawa, J.; Ishida, M.; Nakajima, T.; Honda, Y.; Kitao, O.; Nakai, H.; Vreven, T.; Montgomery, Jr., J. A.; Peralta, J. E.; Ogliaro, F.; Bearpark, M.; Heyd, J. J.; Brothers, E.; Kudin, K. N.; Staroverov, V. N.; Kobayashi, R.; Normand, J.; Raghavachari, K.; Rendell, A.; Burant, J. C.; Iyengar, S. S.; Tomasi, J.; Cossi, M.; Rega, N.; Millam, J. M.; Klene, M.; Knox, J. E.; Cross, J. B.; Bakken, V.; Adamo, C.; Jaramillo, J.; Gomperts, R.; Stratmann, R. E.; Yazyev, O.; Austin, A. J.; Cammi, R.; Pomelli, C.; Ochterski, J. W.; Martin, R. L.; Morokuma, K.; Zakrzewski, V. G.; Voth, G. A.; Salvador, P.; Dannenberg, J. J.; Dapprich, S.; Daniels, A. D.; Farkas, Ö.; Foresman, J. B.; Ortiz, J. V.; Cioslowski, J.; Fox, D. J.Gaussian 09, Rev. C 01; Gaussian, Inc., Wallingford CT, 2009.

(3) Stephens, P. J.; Harada, N. ECD cotton effect approximated by the Gaussian curve and other methods. *Chirality***2010**, *22*, 229–233.

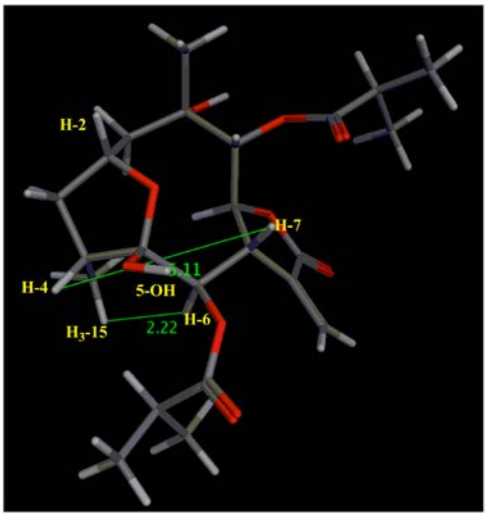

1

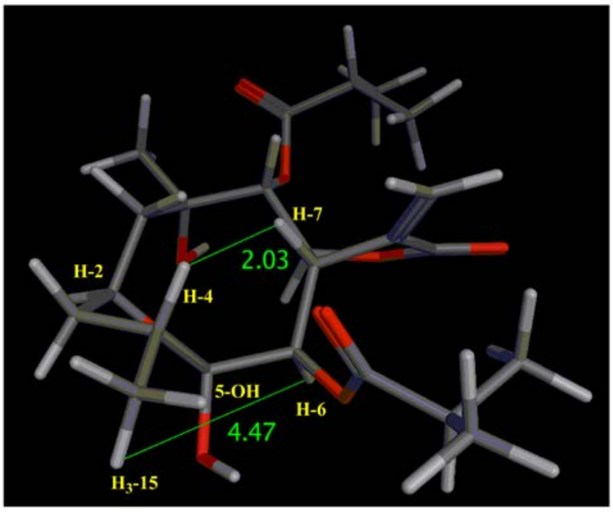

8

Fig. C3 The simulated distances of H-4/H-7 and H3-15/H-6 by MOE software

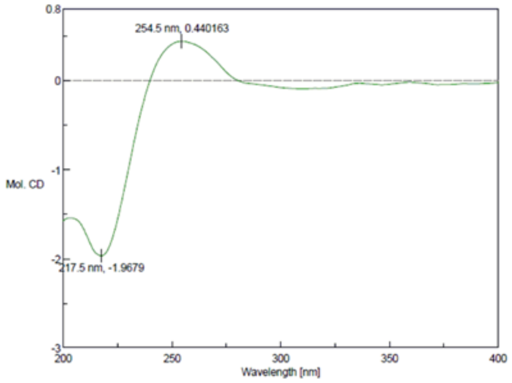

8

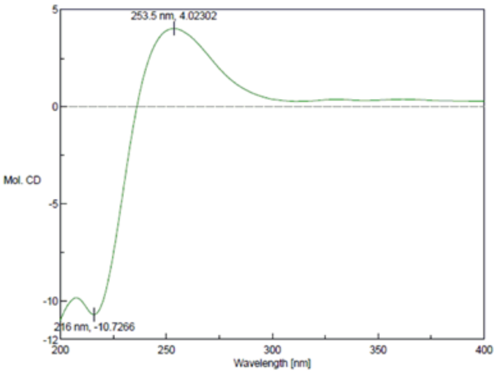

9

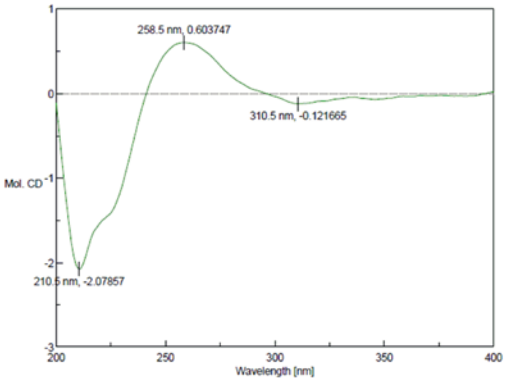

10

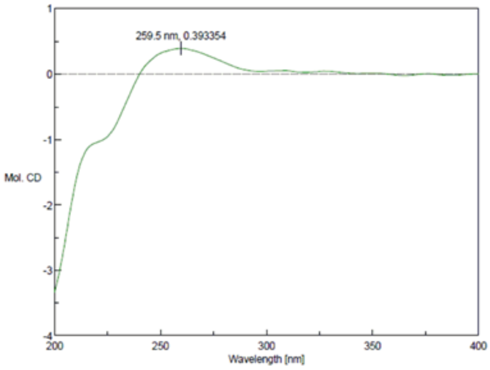

11

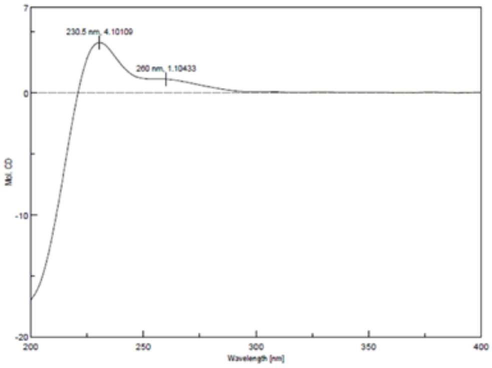

12

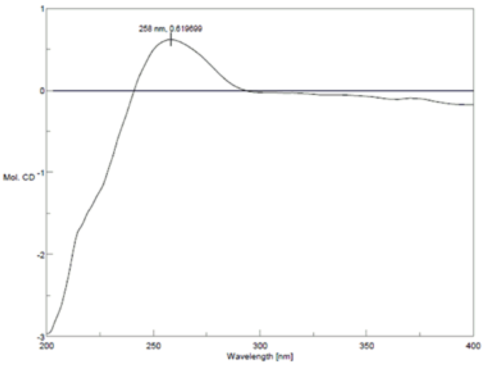

13

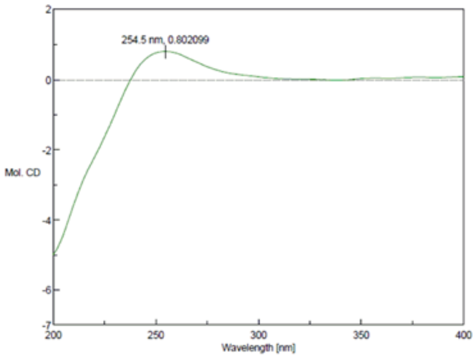

14

Fig. C4 CD spectra of compounds **8**-**14**

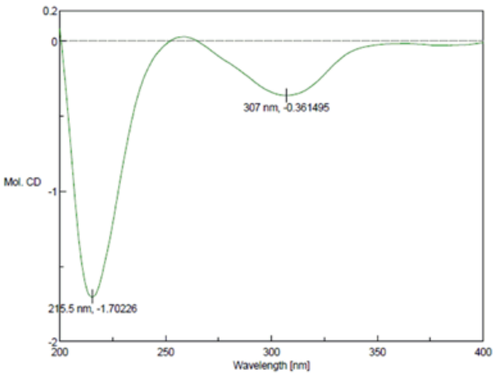

15

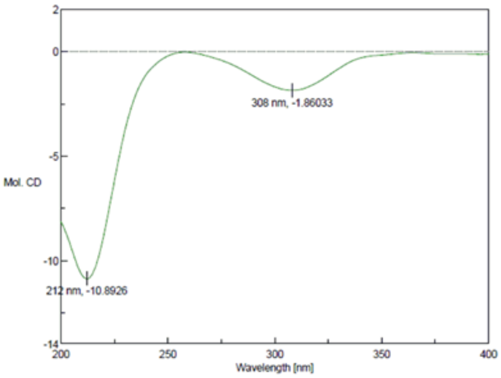

16

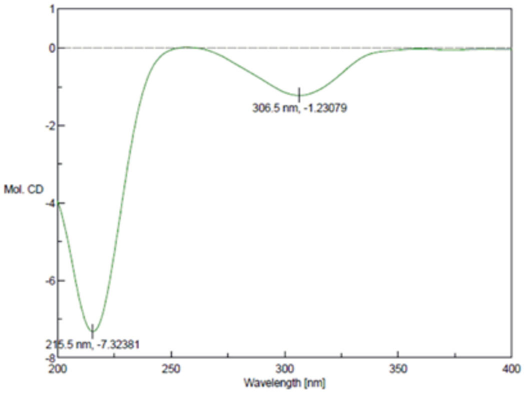

17

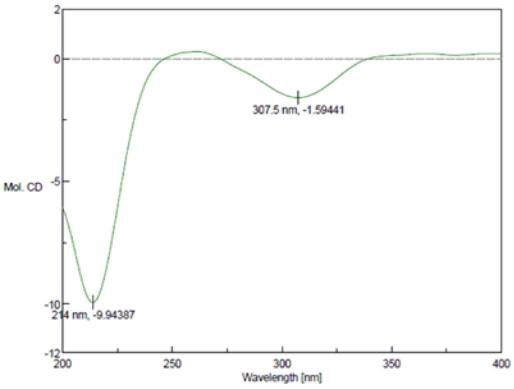

18

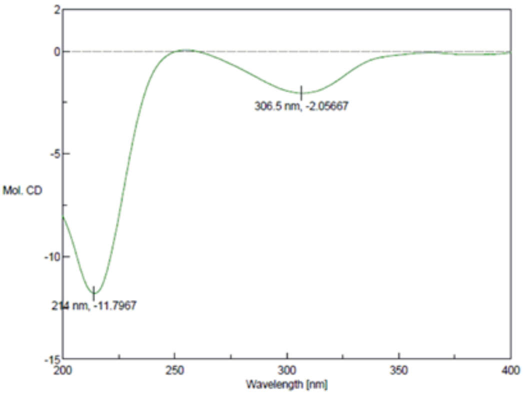

19

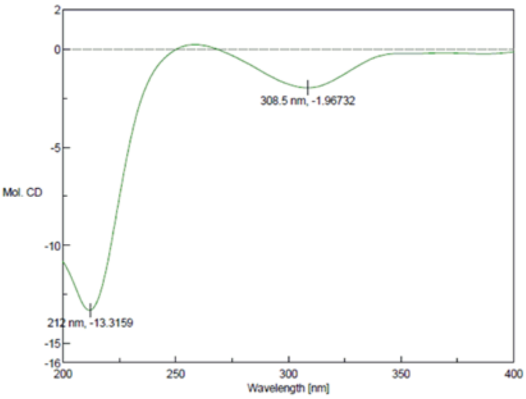

20

Fig. C5 CD spectra of compounds **15-20**

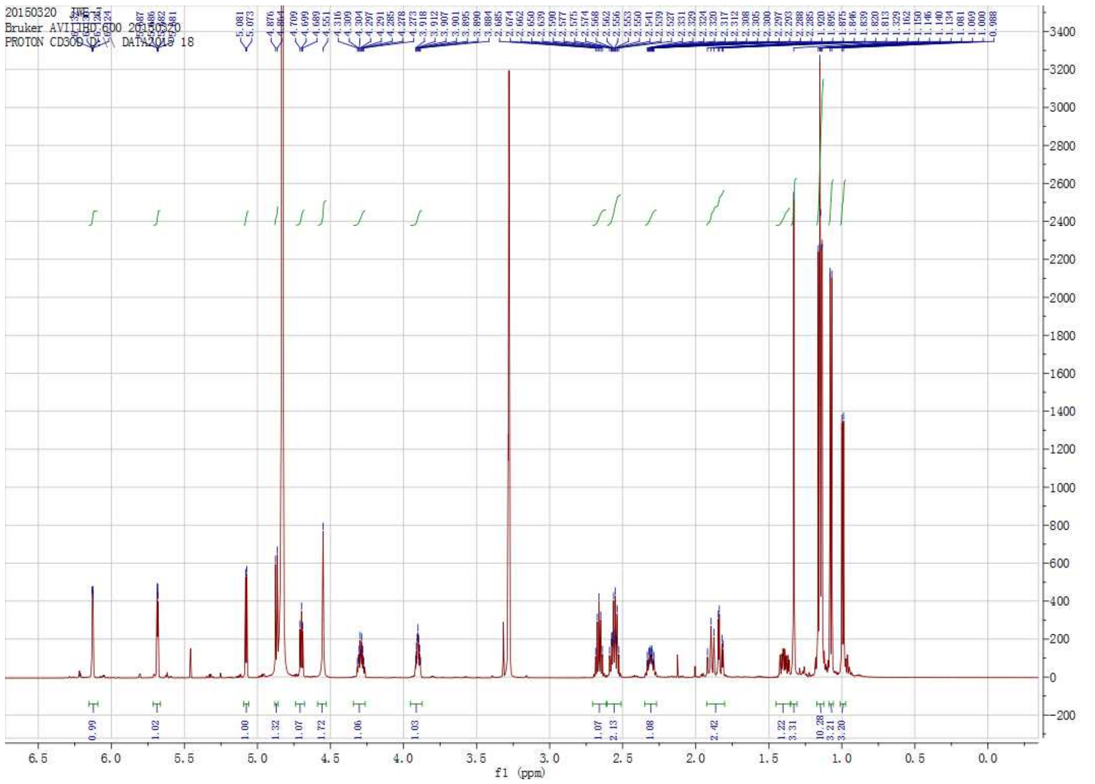

Fig. S1.1 ^{1}H NMR spectrum (600 MHz) of (2*R*, 5*S*)-cardivarolide A (**1**) in CD_3OD

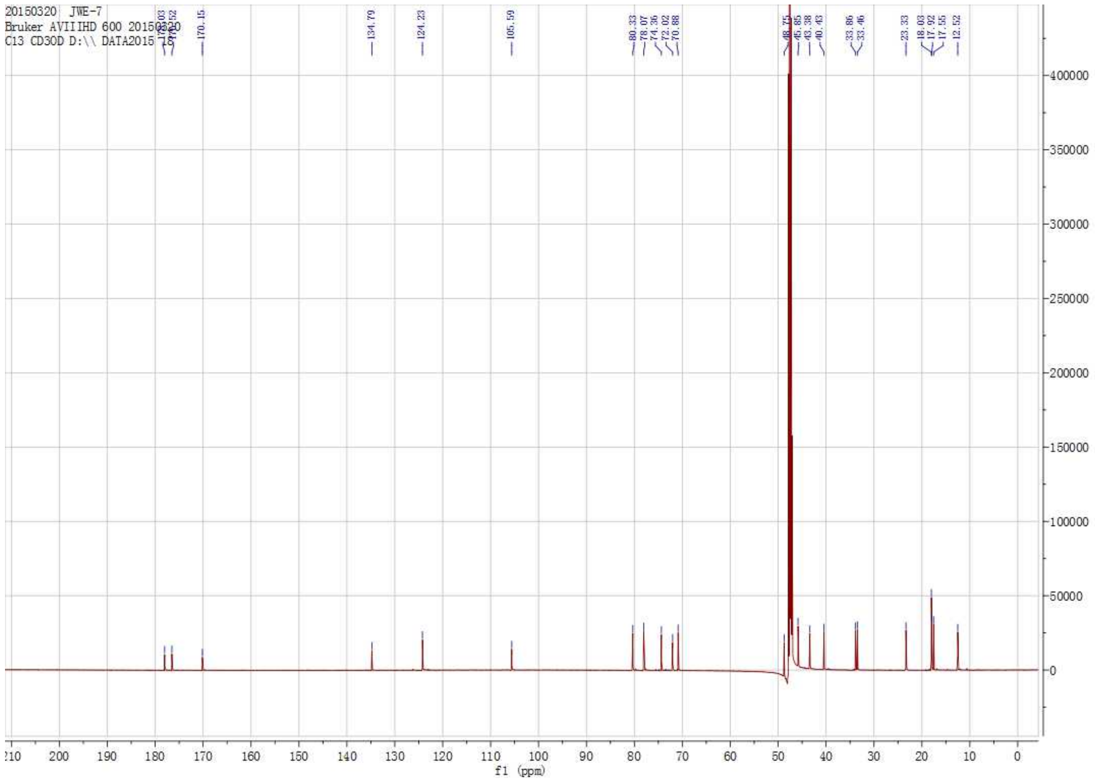

Fig. S1.2 ^{13}C NMR spectrum (150 MHz) of (2*R*, 5*S*)-cardivarolide A (**1**) in CD_3OD

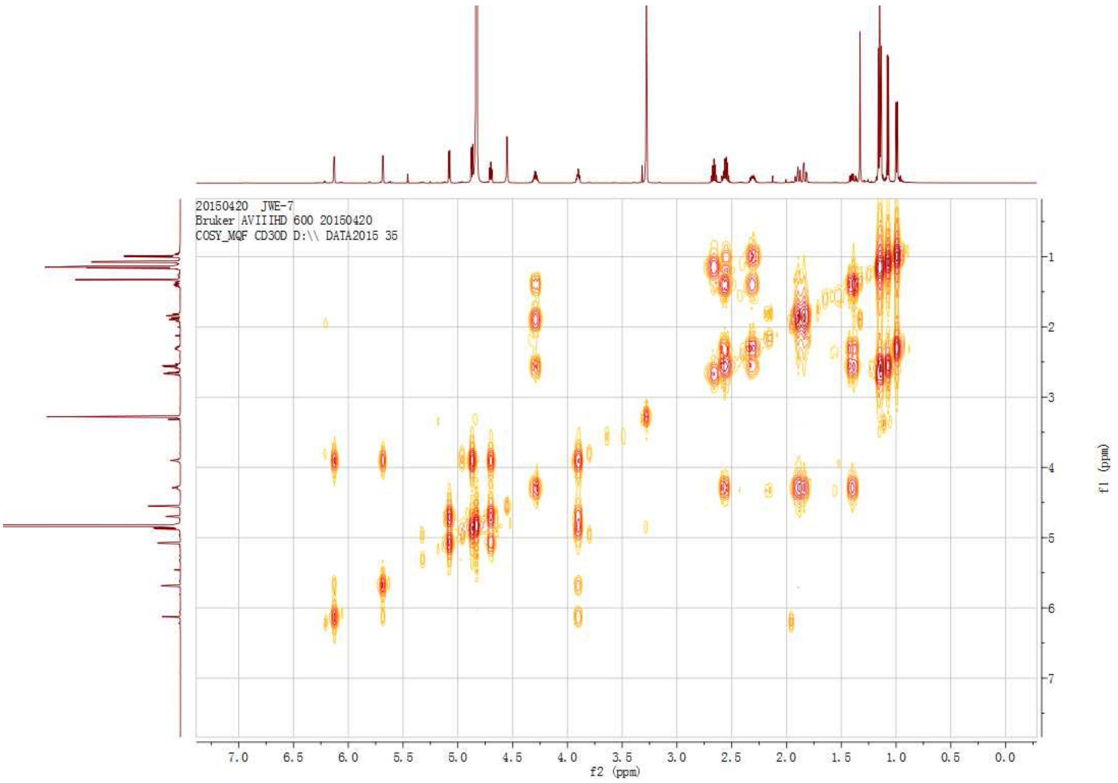

Fig. S1.3 ^{1}H-^{1}H COSY spectrum (600 MHz) of (2*R*, 5*S*)-cardivarolide A (**1**) in CD_3OD

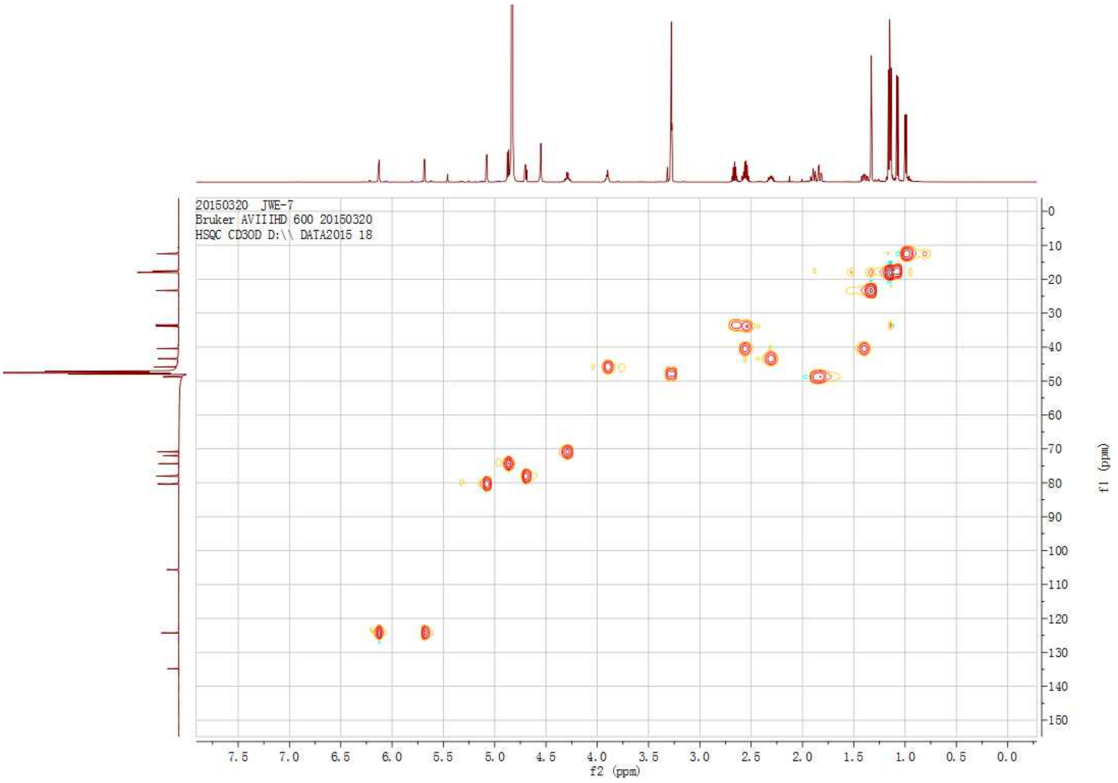

Fig. S1.4 HSQC spectrum (600 MHz) of (2*R*, 5*S*)-cardivarolide A (**1**) in CD_3OD

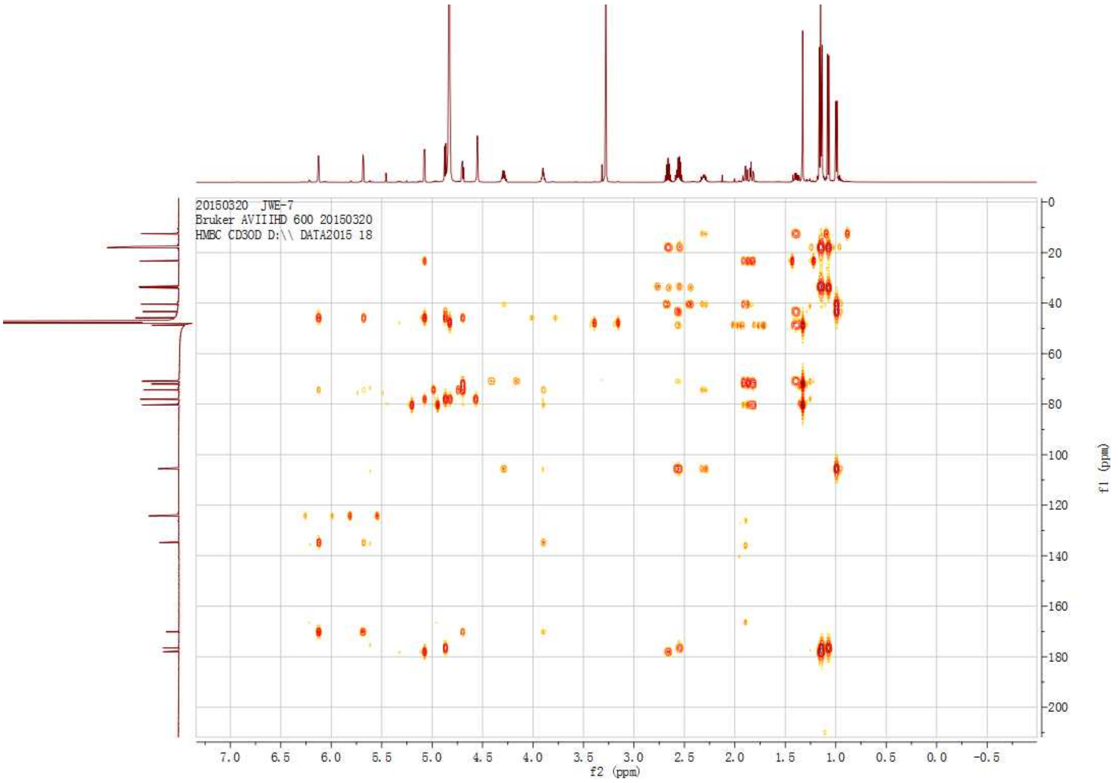

Fig. S1.5 HMBC spectrum (600 MHz) of (2*R*, 5*S*)-cardivarolide A (**1**) in CD_3OD

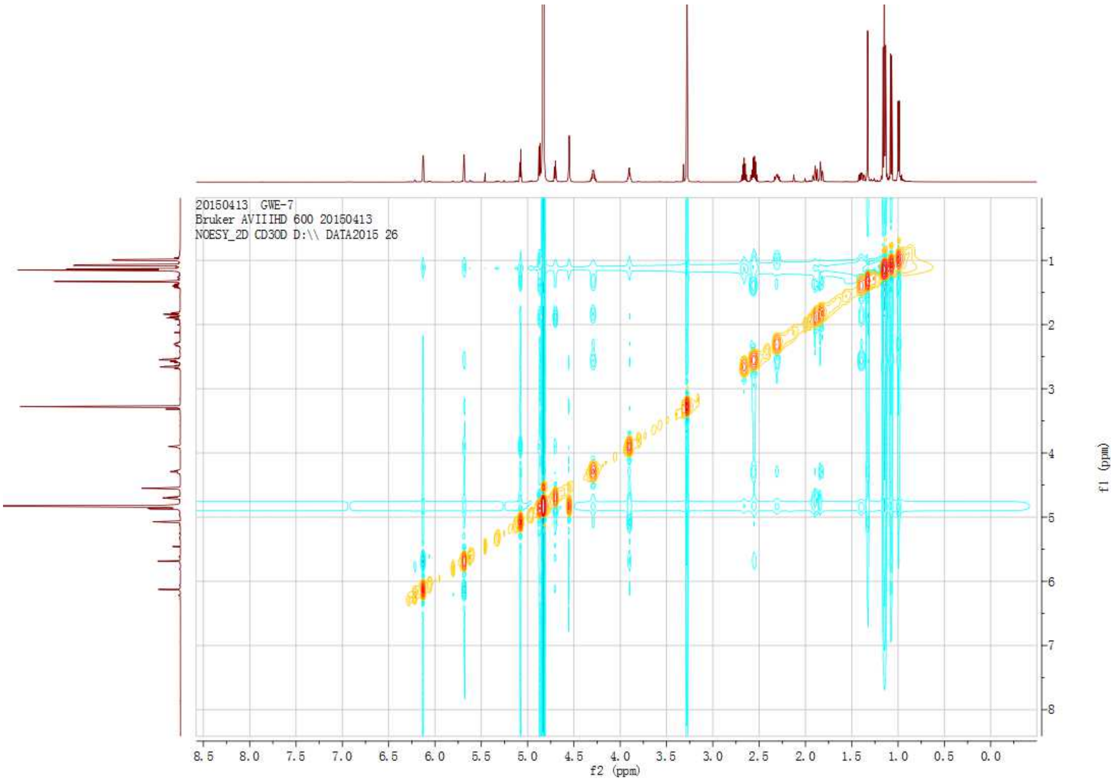

Fig. S1.6 NOESY spectrum (600 MHz) of (2*R*, 5*S*)-cardivarolide A (**1**) in CD_3OD

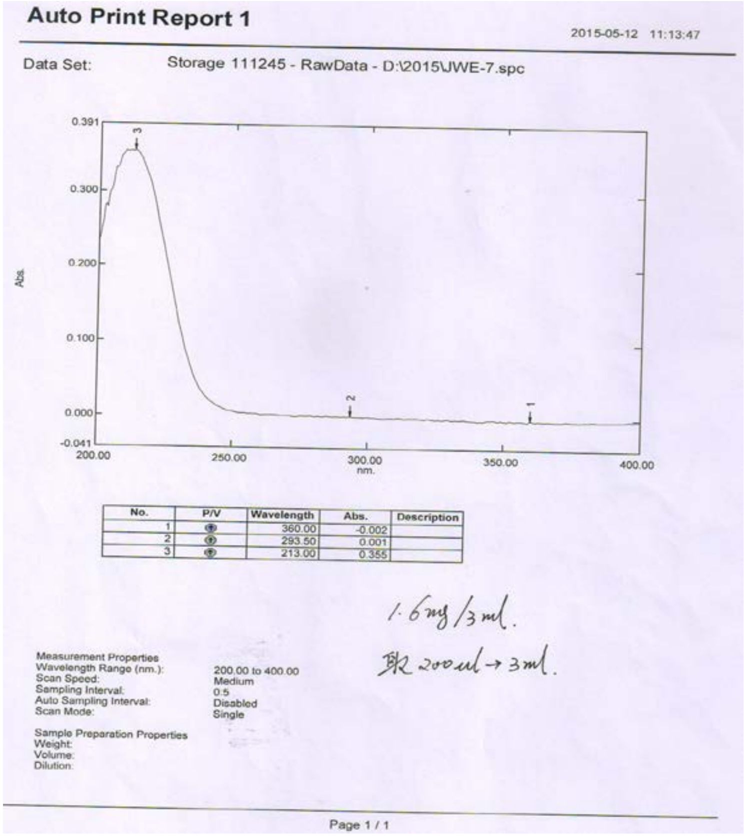

No.	P/V	Wavelength	Abs.	Description
1	⊕	360.00	-0.002	
2	⊕	293.50	0.001	
3	⊕	213.00	0.355	

Fig. S1.7 UV spectrum of (2*R*, 5*S*)-cardivarolide A (**1**)

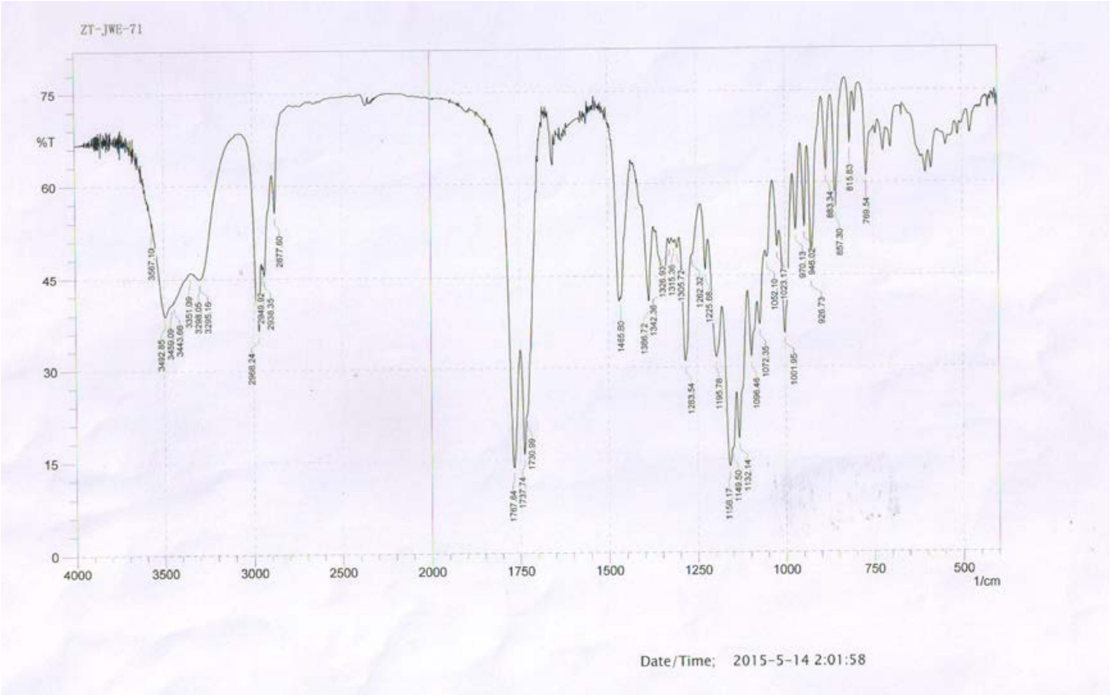

Fig. S1.8 IR spectrum of (2*R*, 5*S*)-cardivarolide A (**1**)

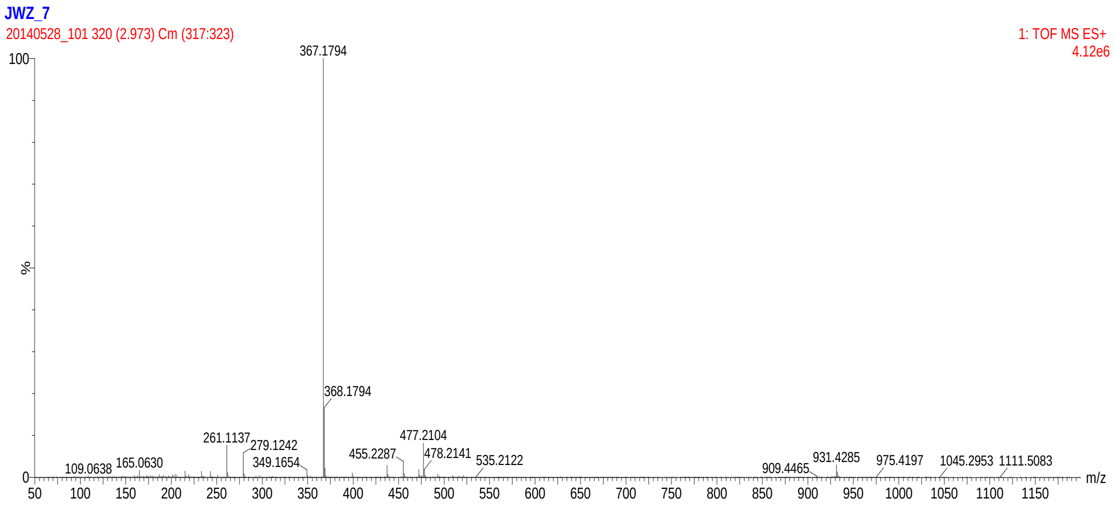

Fig. S1.9 HRESIMS spectrum of (2*R*, 5*S*)-cardivarolide A (**1**)

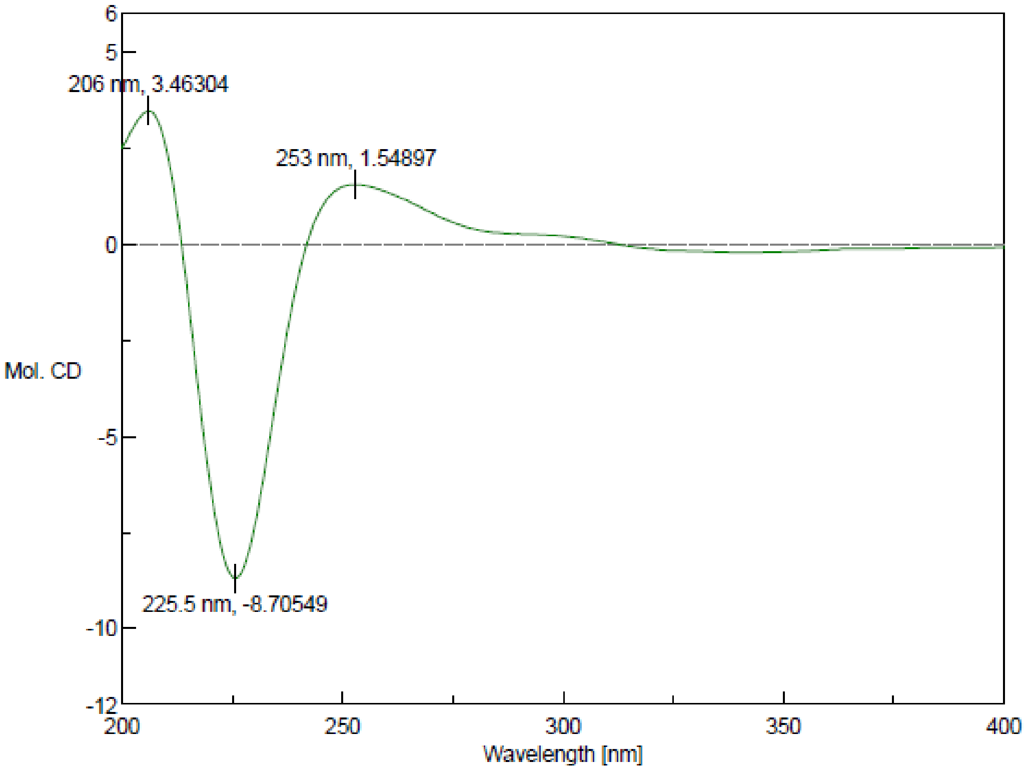

Fig. S1.10 CD spectrum of (2*R*, 5*S*)-cardivarolide A (**1**)

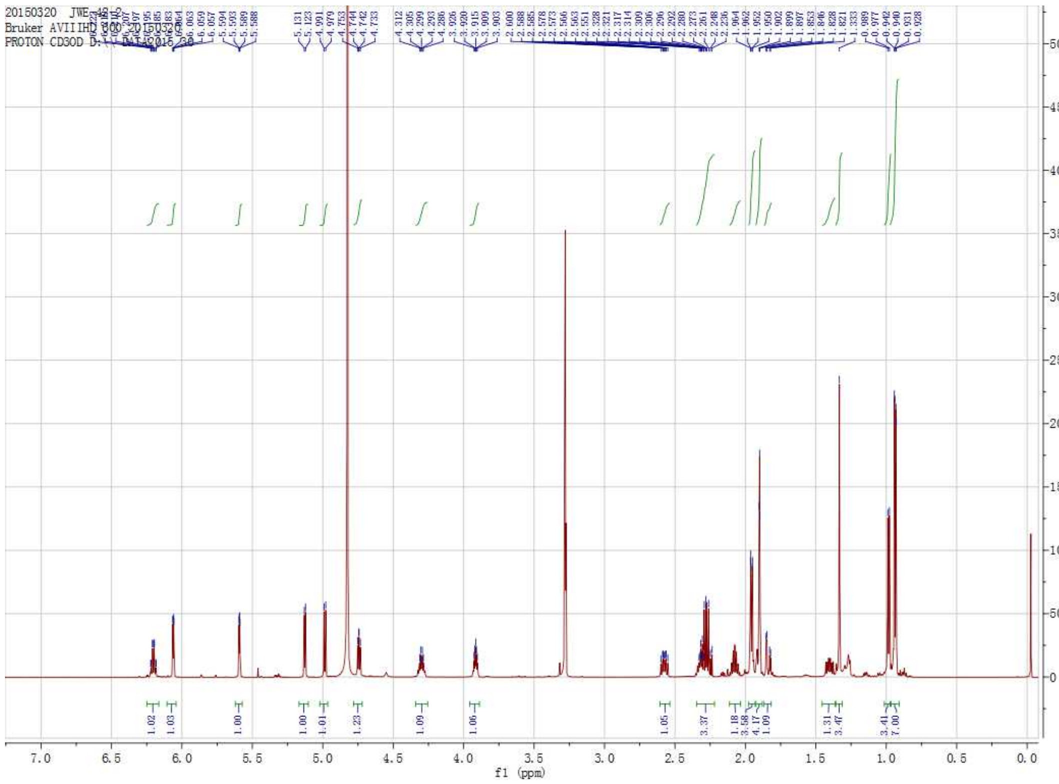

Fig. S2.1 ^{1}H NMR spectrum (600 MHz) of (2*R*, 5*S*)-cardivarolide B (**2**) in CD_3OD

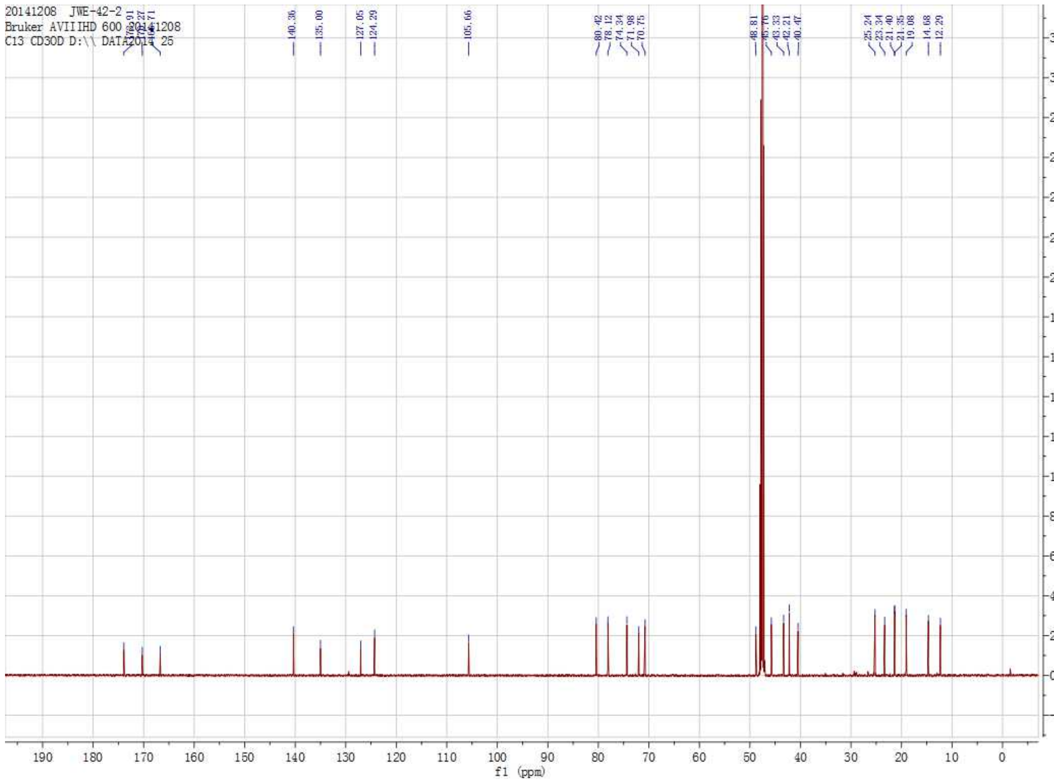

Fig. S2.2 ^{13}C NMR spectrum (150 MHz) of (2*R*, 5*S*)-cardivarolide B (**2**) in CD_3OD

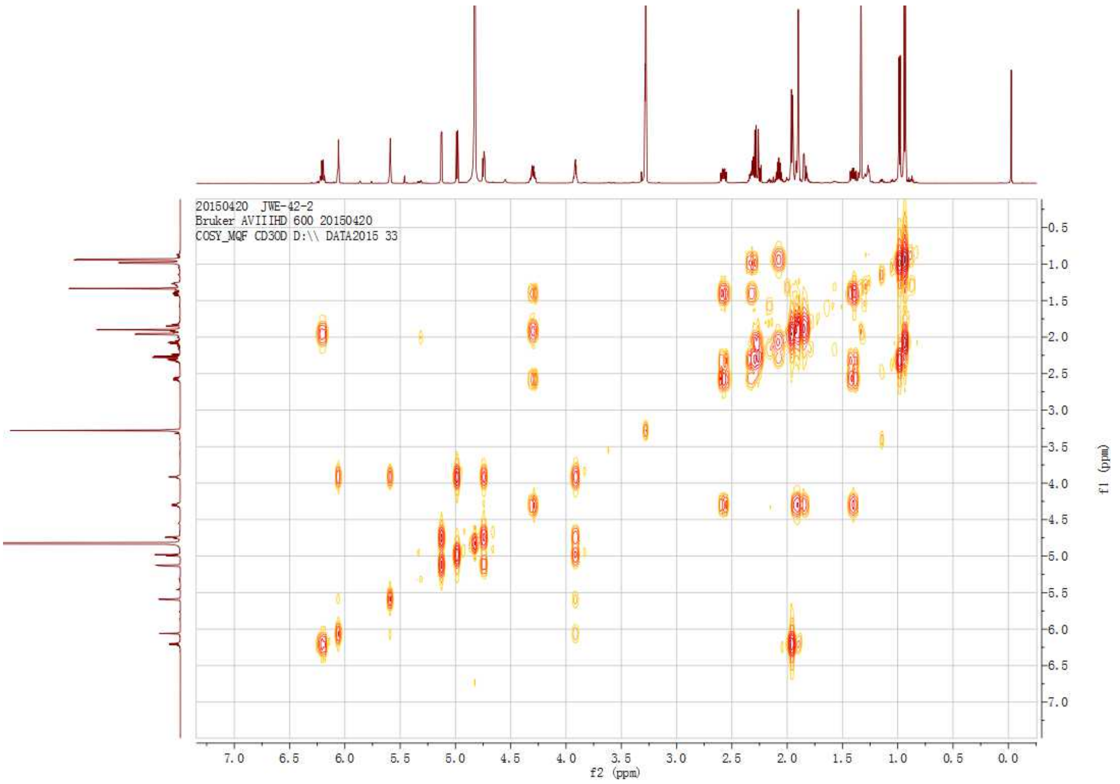

Fig. S2.3 ^{1}H-^{1}H COSY spectrum (600 MHz) of (2*R*, 5*S*)-cardivarolide B (**2**) in CD_3OD

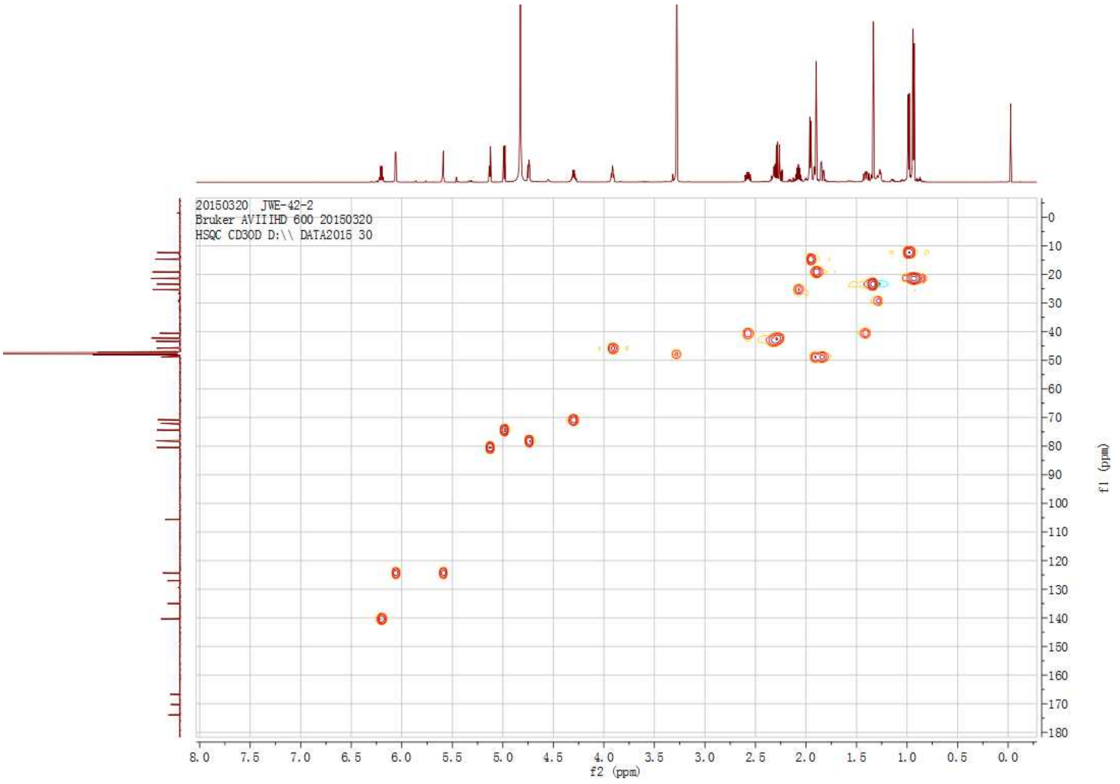

Fig. S2.4 HSQC spectrum (600 MHz) of (2*R*, 5*S*)-cardivarolide B (**2**) in CD_3OD

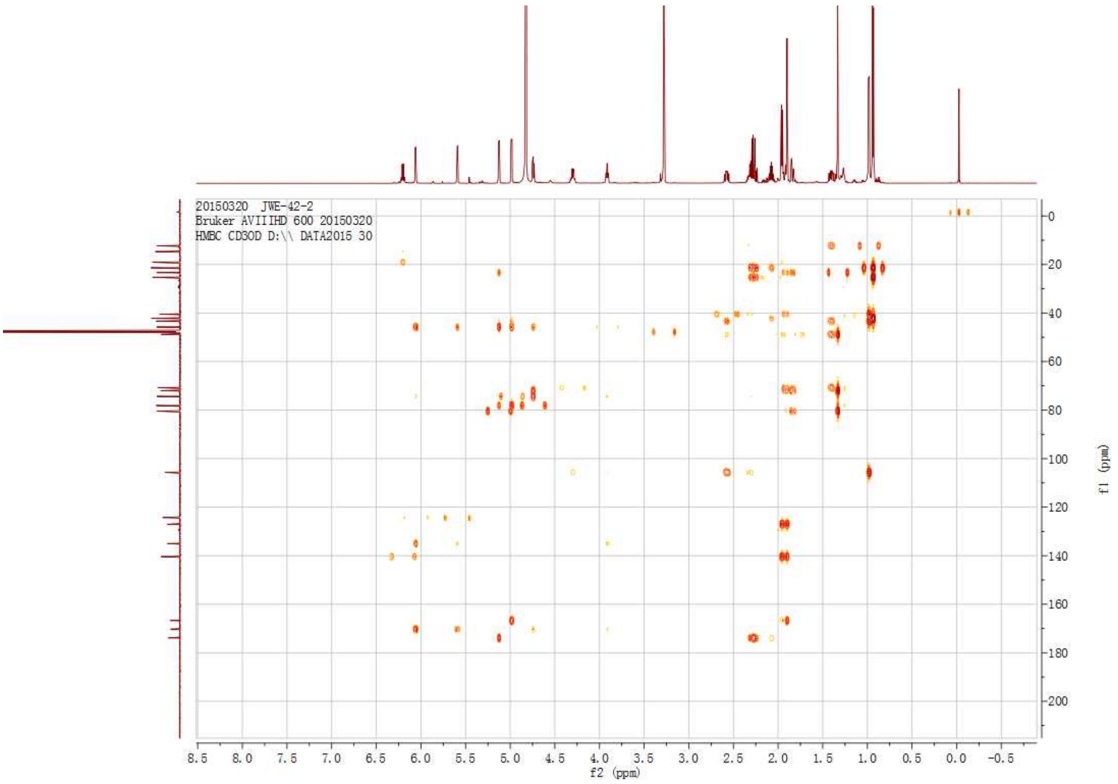

Fig. S2.5 HMBC spectrum (600 MHz) of (2*R*, 5*S*)-cardivarolide B (**2**) in CD_3OD

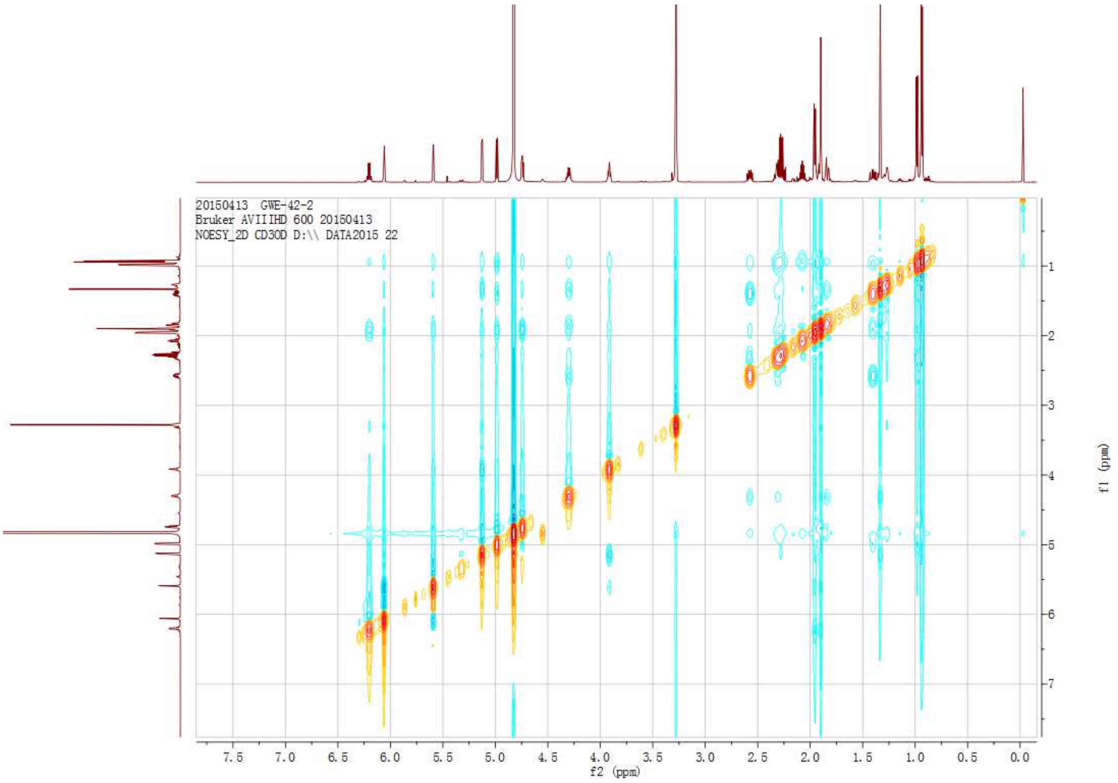

Fig. S2.6 NOESY spectrum (600 MHz) of (2*R*, 5*S*)-cardivarolide B (**2**) in CD_3OD

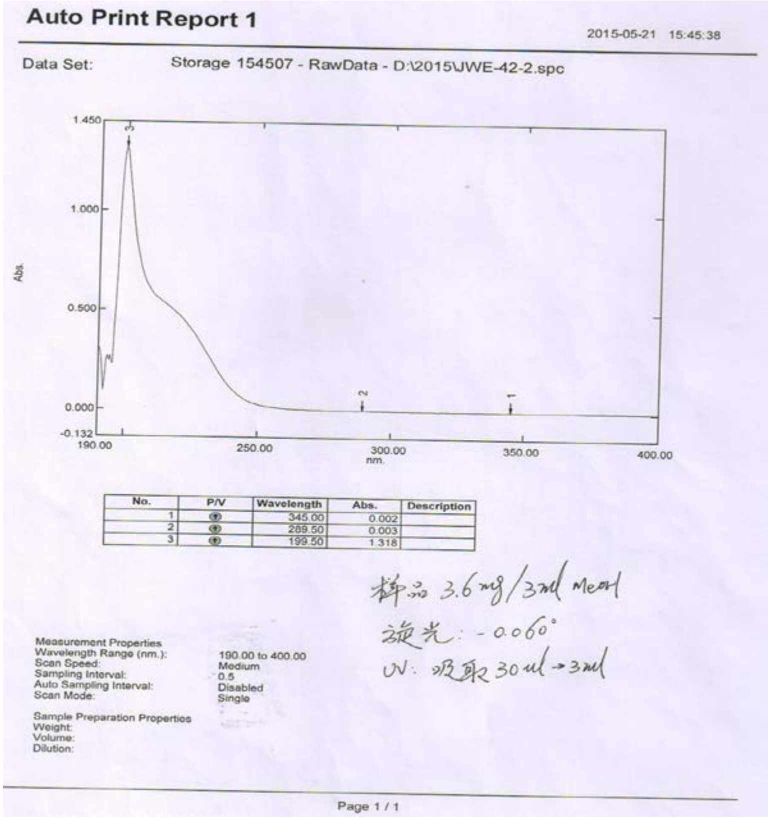

Fig. S2.7 UV spectrum of (2*R*, 5*S*)-cardivarolide B (**2**)

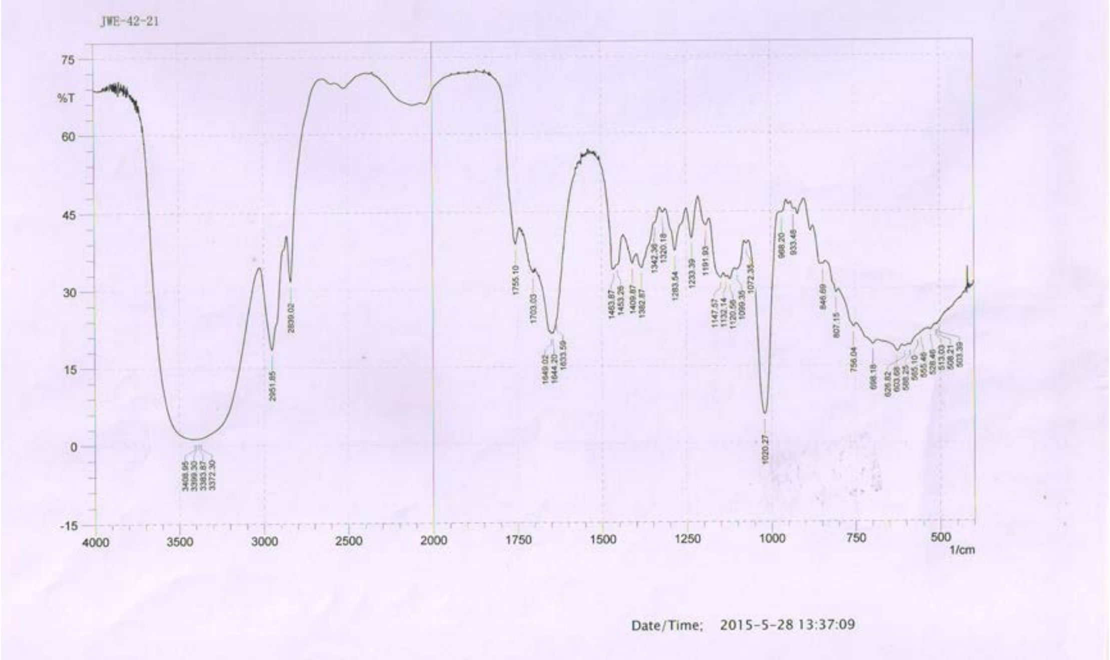

Fig. S2.8 IR spectrum of (2*R*, 5*S*)-cardivarolide B (**2**)

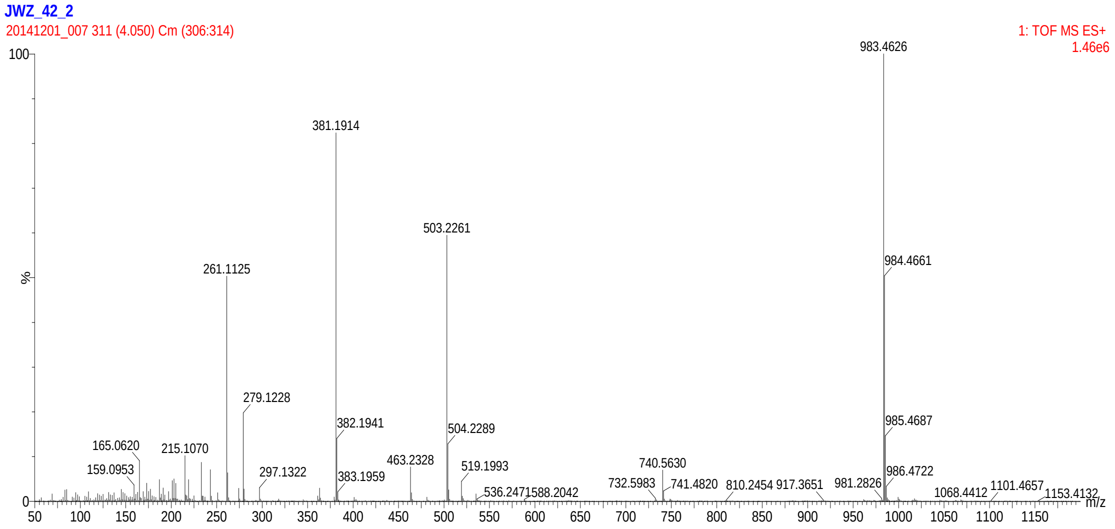

Fig. S2.9 HRESIMS spectrum of (2*R*, 5*S*)-cardivarolide B (**2**)

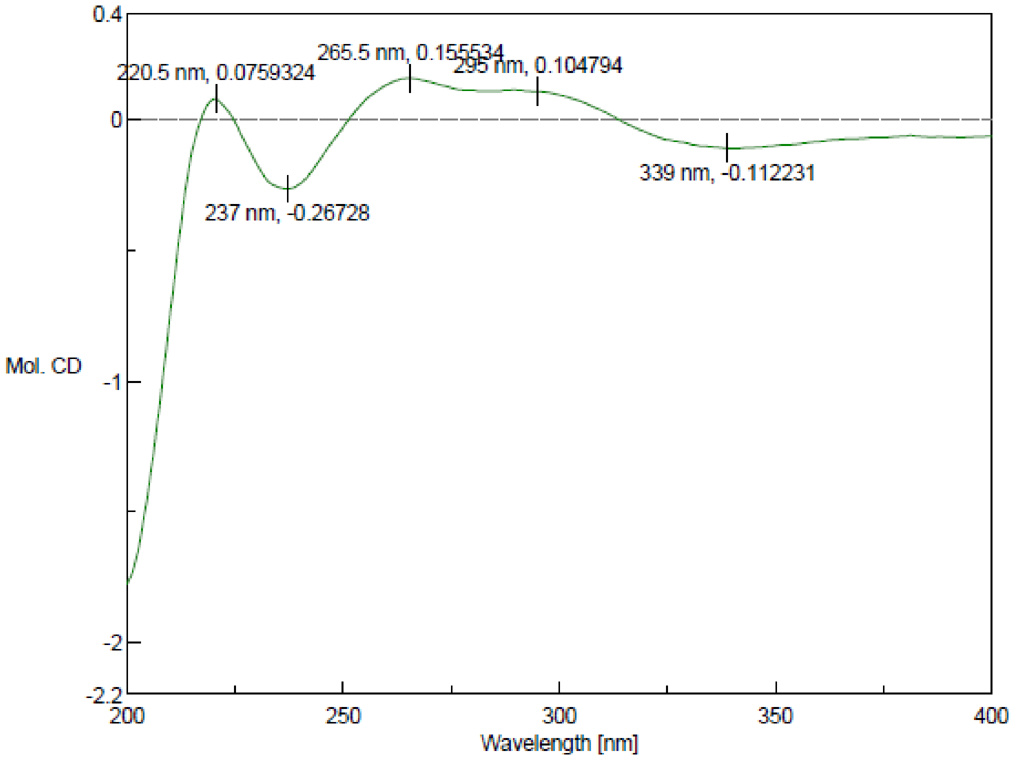

Fig. S2.10 CD spectrum of (2*R*, 5*S*)-cardivarolide B (**2**)

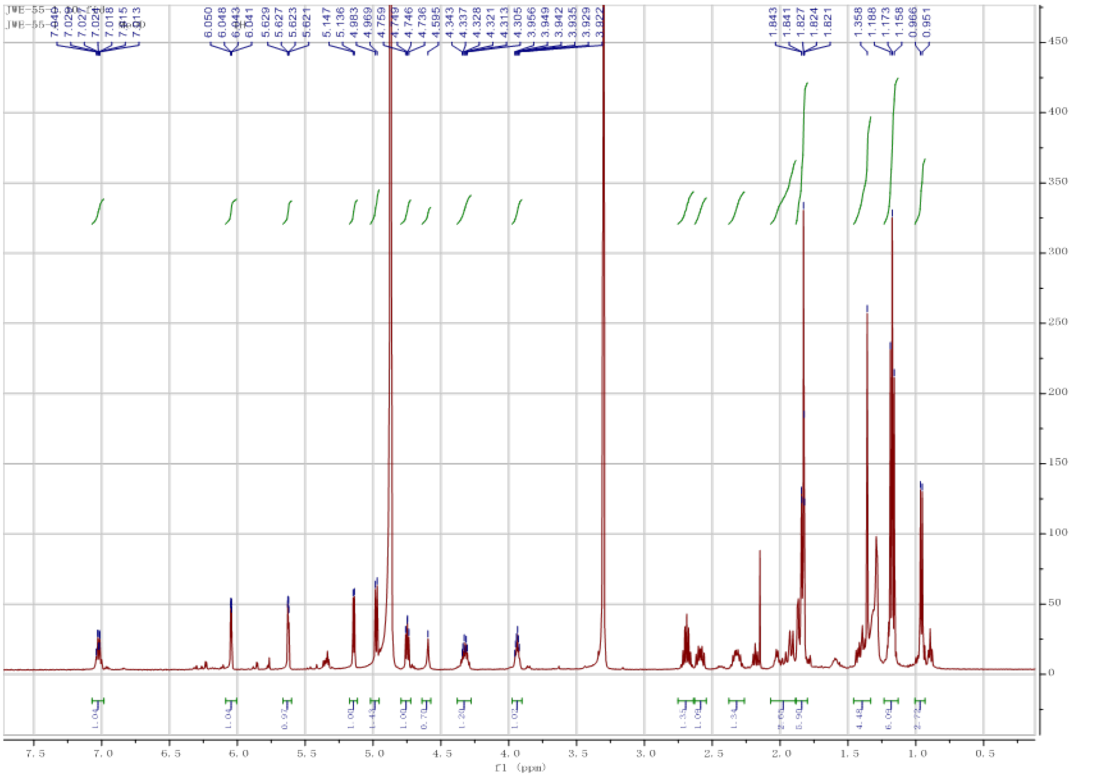

Fig. S3.1 ^{1}H NMR spectrum (500 MHz) of (2*R*, 5*S*)-ciscardivarolide C (**3**) in CD_3OD

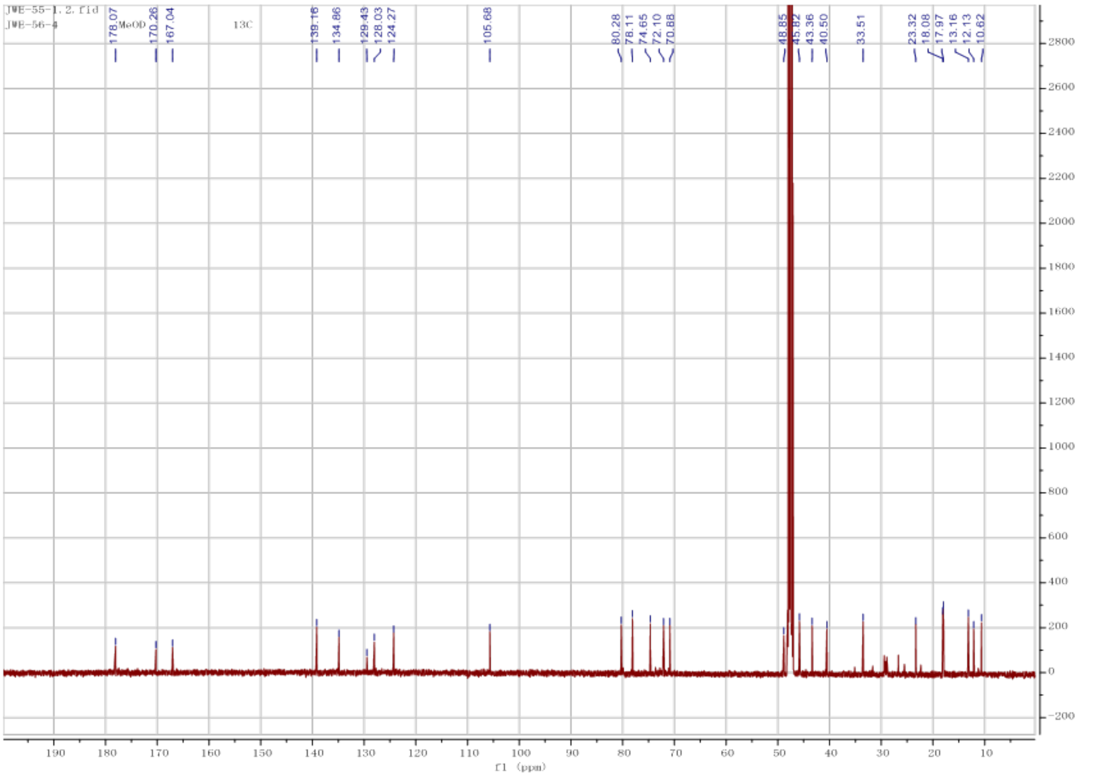

Fig. S3.2 ^{13}C NMR spectrum (125 MHz) of (2*R*, 5*S*)-ciscardivarolide C (**3**) in CD_3OD

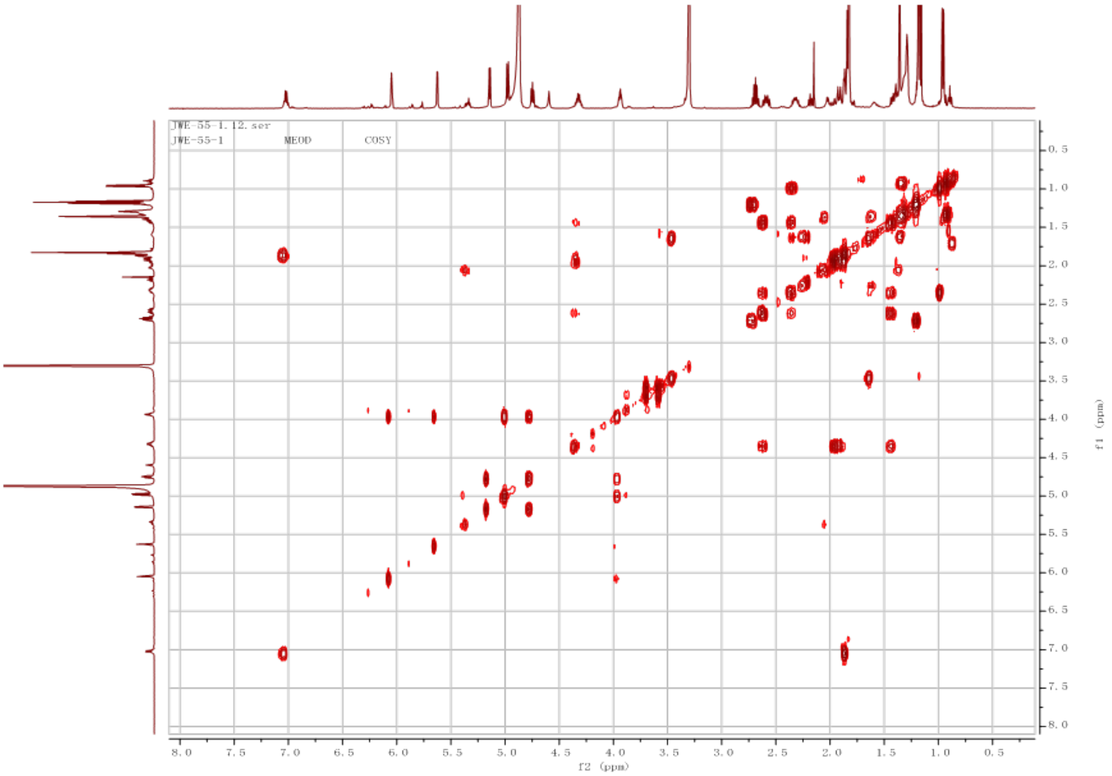

Fig. S3.3 ^{1}H-^{1}H COSY spectrum (500 MHz) of (2*R*, 5*S*)-ciscardivarolide C (**3**) in CD_3OD

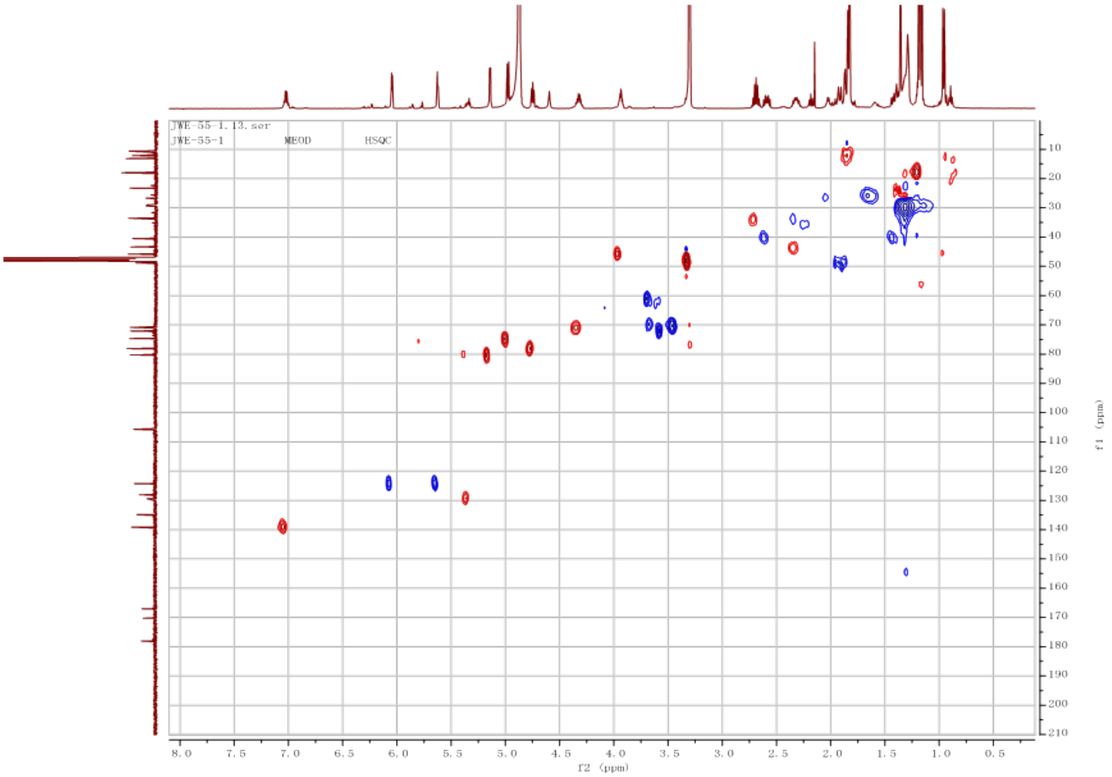

Fig. S3.4 HSQC spectrum (500 MHz) of (2*R*, 5*S*)-ciscardivarolide C (**3**) in CD_3OD

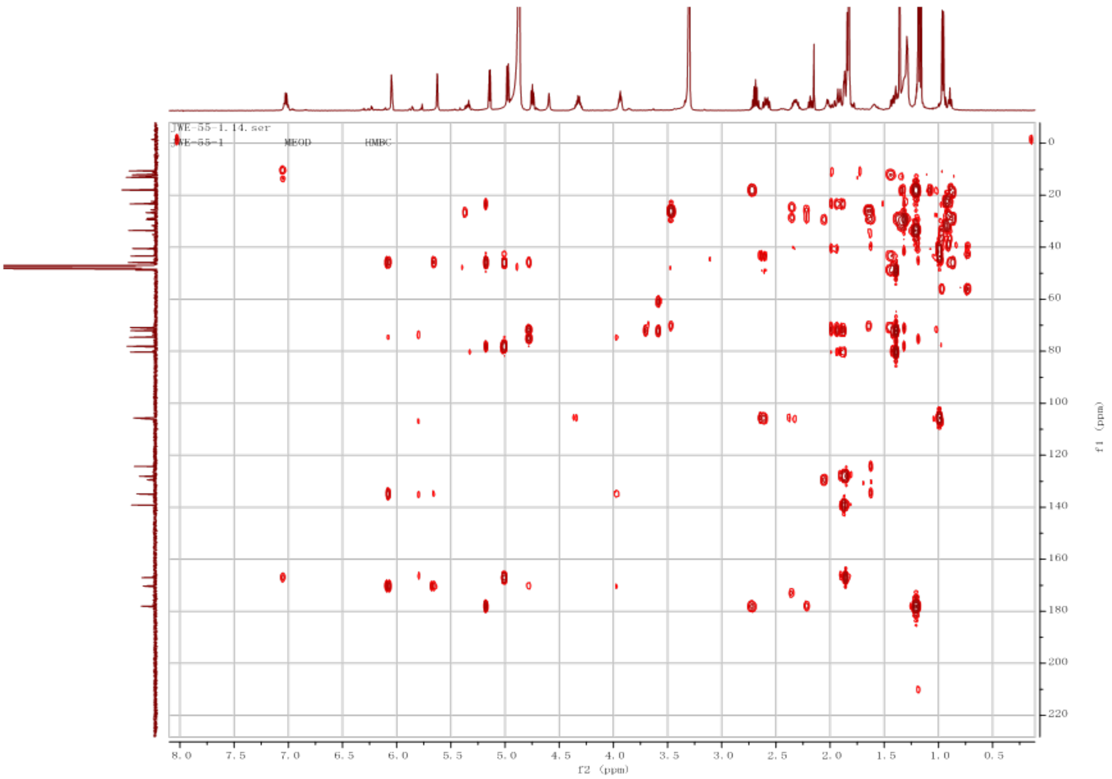

Fig. S3.5 HMBC spectrum (500 MHz) of (2*R*, 5*S*)-ciscardivarolide C (**3**) in CD_3OD

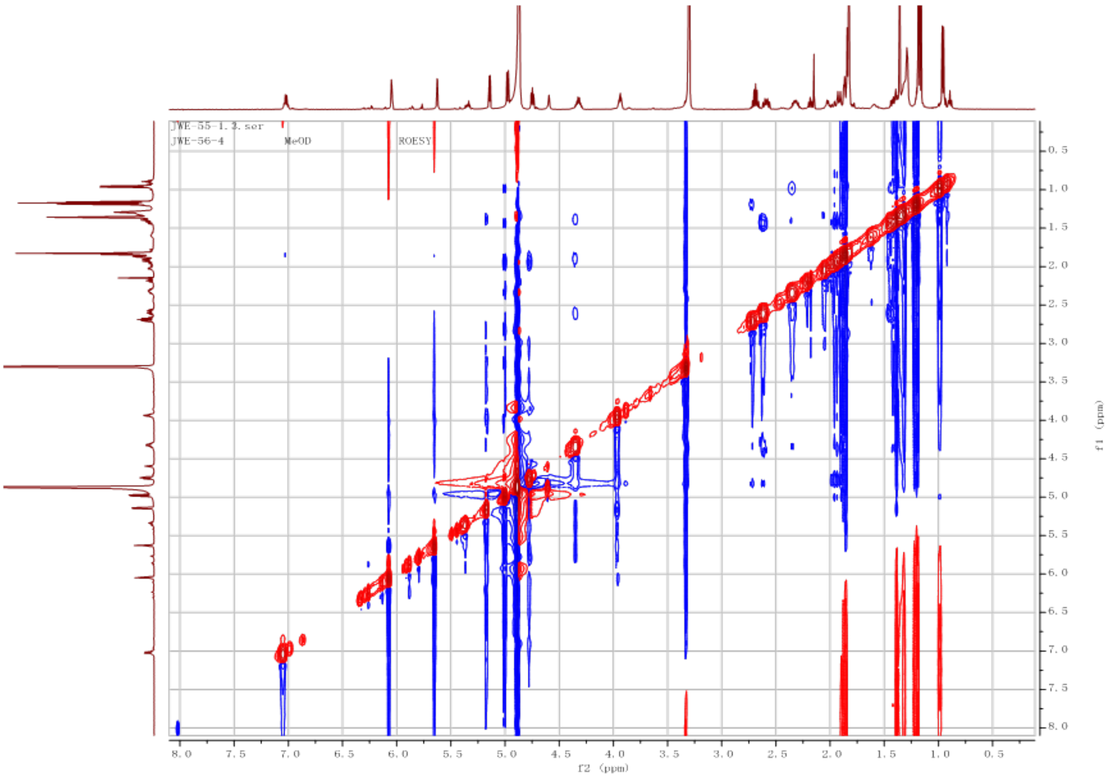

Fig. S3.6 ROESY spectrum (500 MHz) of (2*R*, 5*S*)-ciscardivarolide C (**3**) in CD_3OD

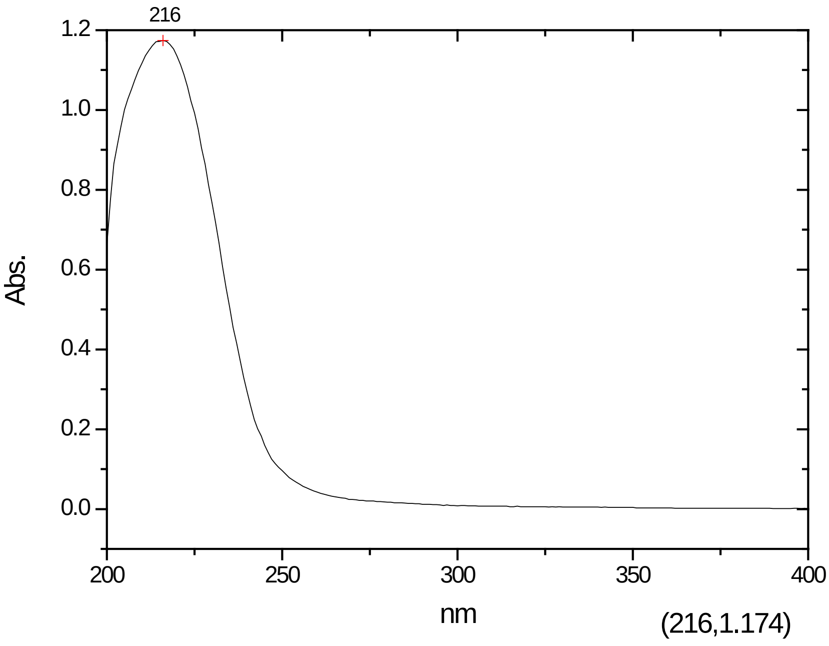

Fig. S3.7 UV spectrum of (2*R*, 5*S*)-ciscardivarolide C (**3**)

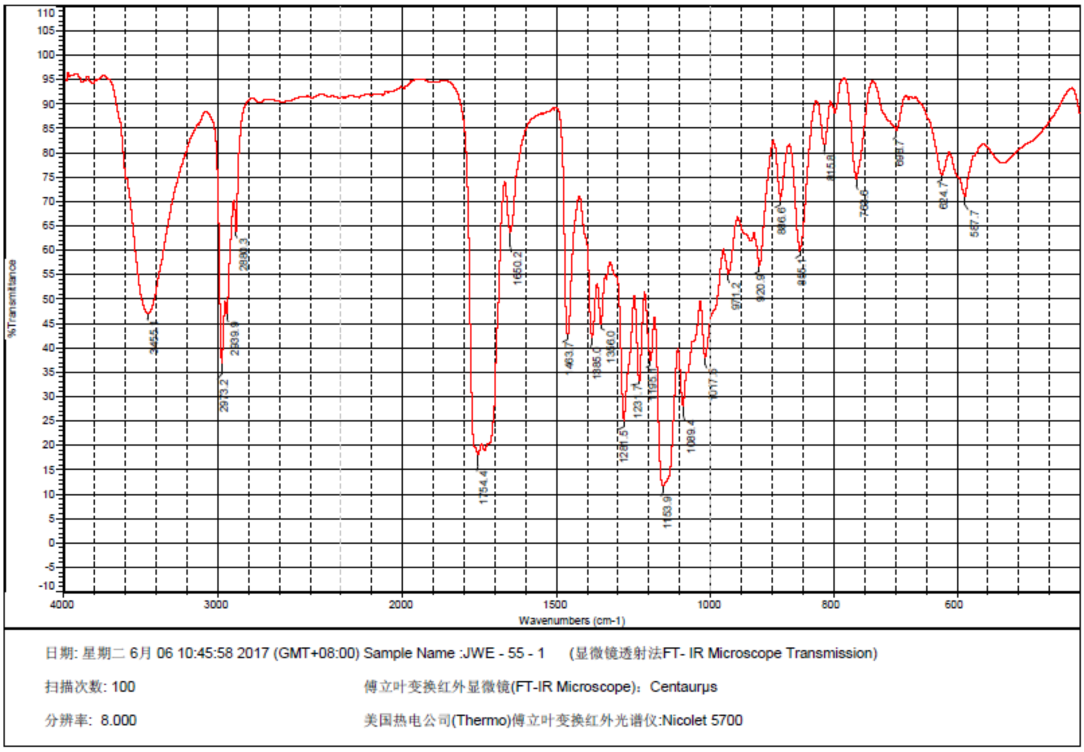

Fig. S3.8 IR spectrum of (2*R*, 5*S*)-ciscardivarolide C (**3**)

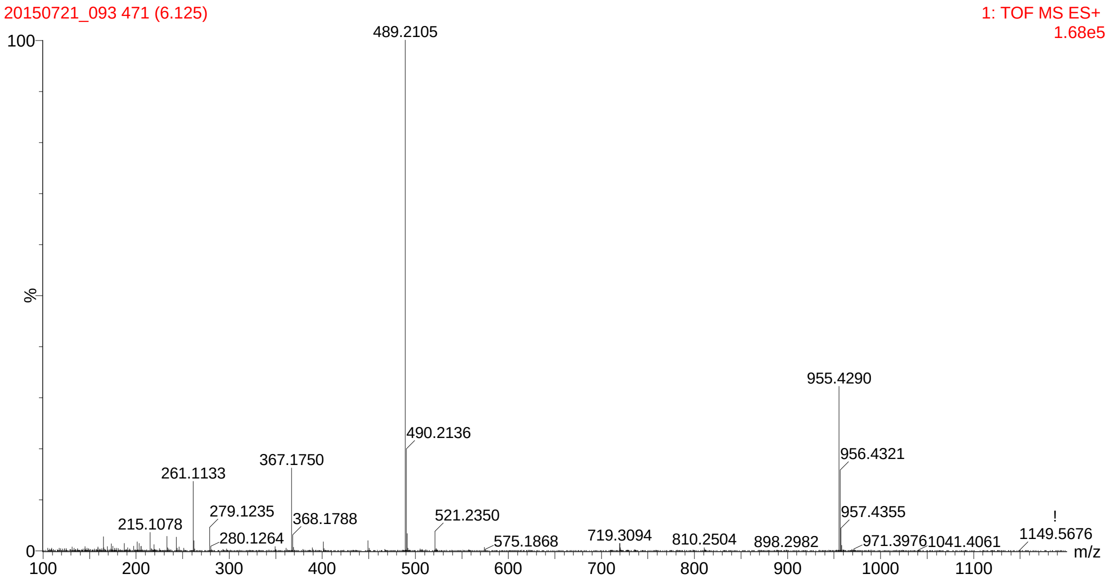

Fig. S3.9 HRESIMS spectrum of (2*R*, 5*S*)-ciscardivarolide C (**3**)

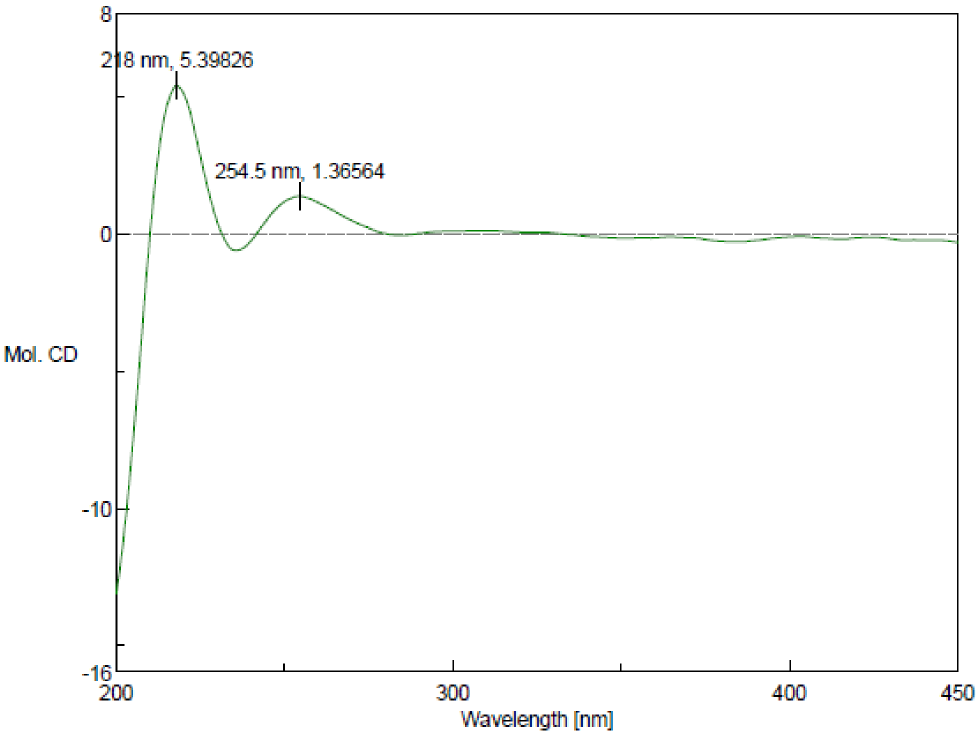

Fig. S3.10 CD spectrum of (2*R*, 5*S*)-ciscardivarolide C (**3**)

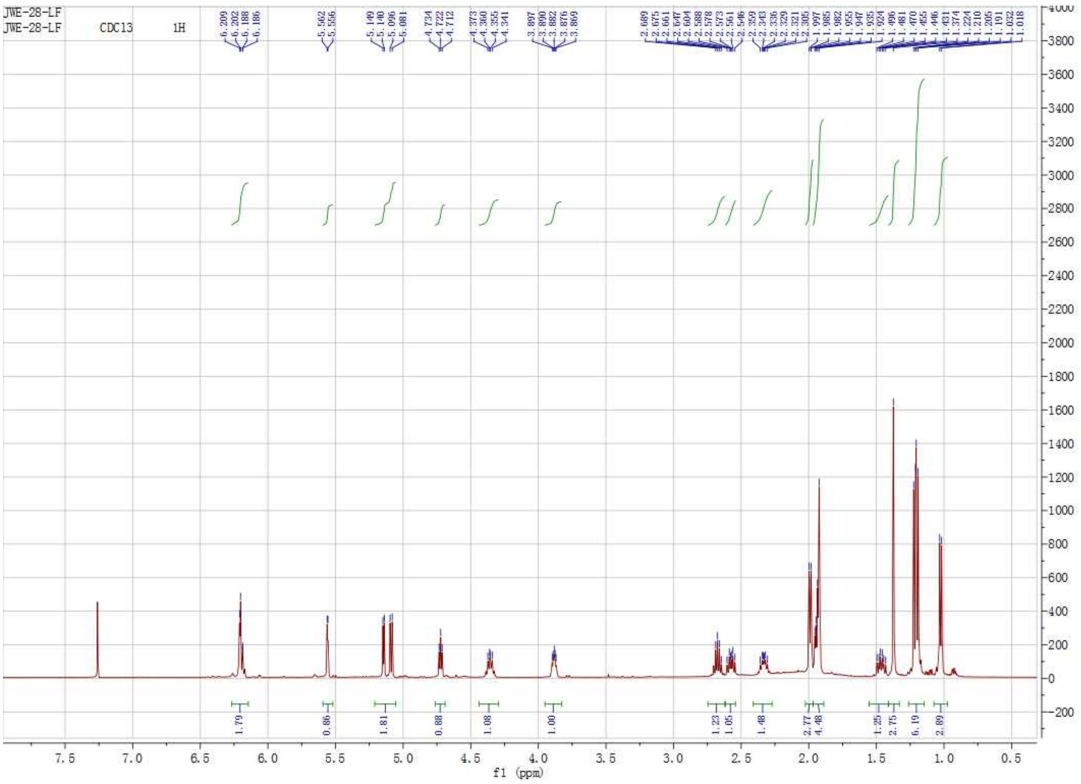

Fig. S4.1 ^{1}H NMR spectrum (500 MHz) of (2*R*, 5*S*)-cardivarolide C (**4**) in $CDCl_3$

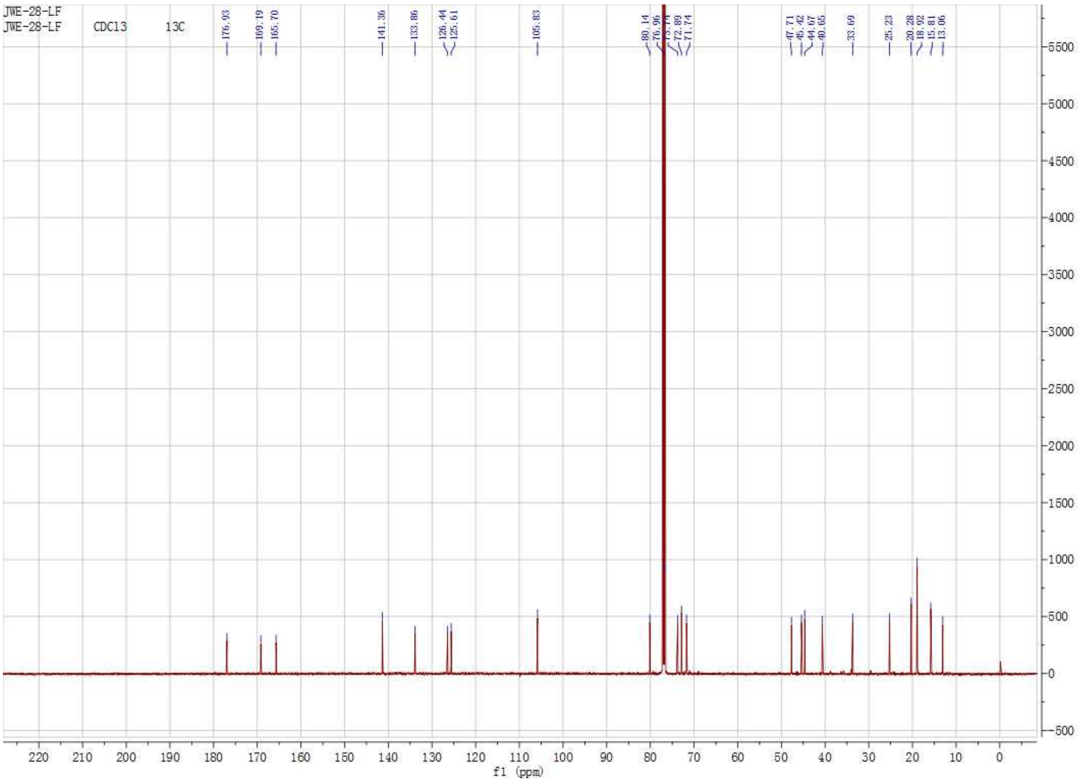

Fig. S4.2 ^{13}C NMR spectrum (125 MHz) of (2*R*, 5*S*)-cardivarolide C (**4**) in $CDCl_3$

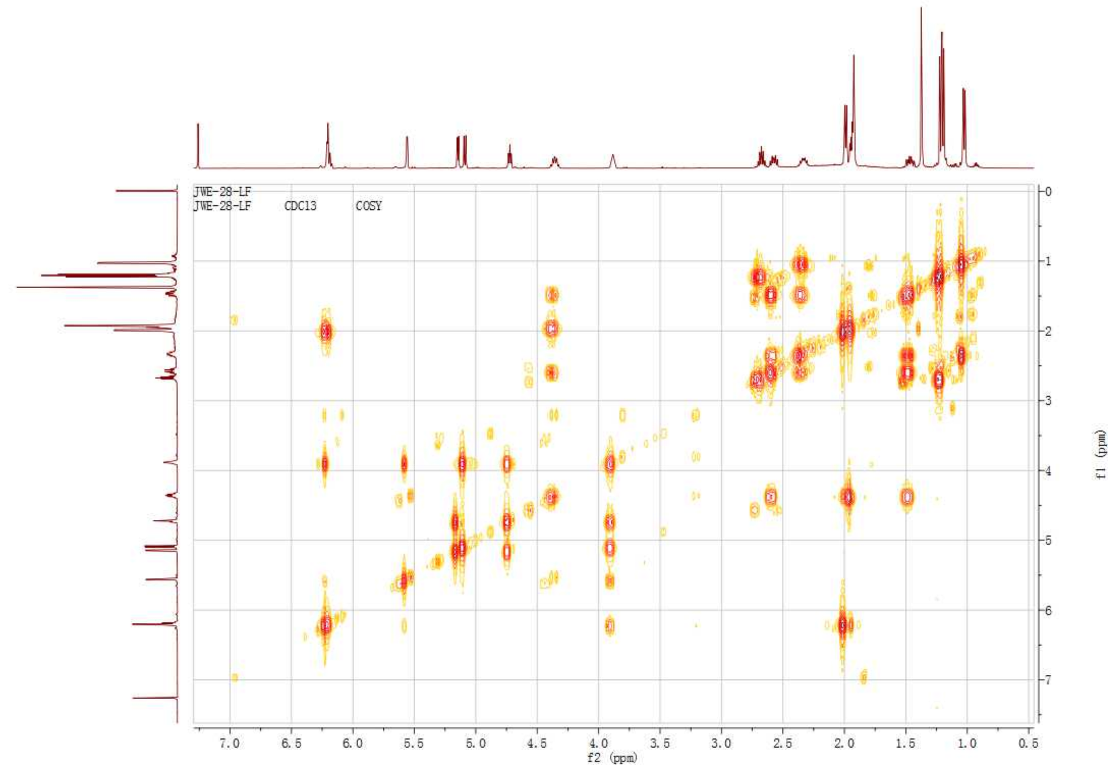

Fig. S4.3 ^{1}H-^{1}H COSY spectrum (500 MHz) of (2*R*, 5*S*)-cardivarolide C (**4**) in $CDCl_3$

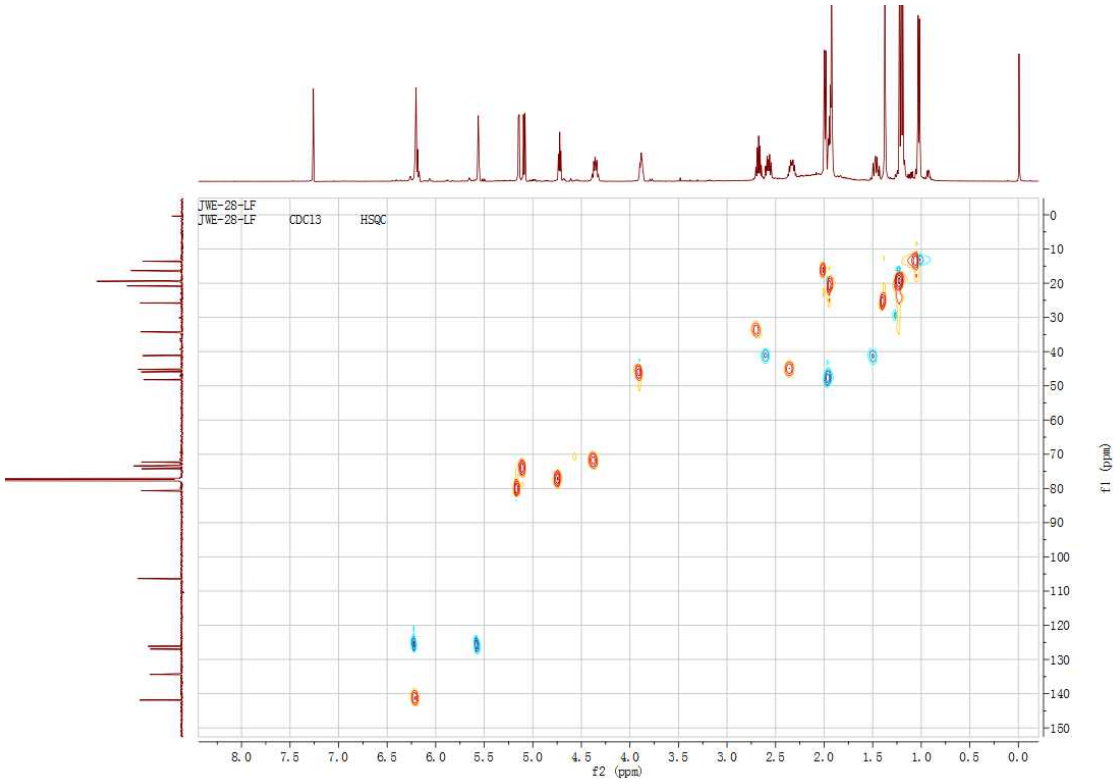

Fig. S4.4 HSQC spectrum (500 MHz) of (2*R*, 5*S*)-cardivarolide C (**4**) in $CDCl_3$

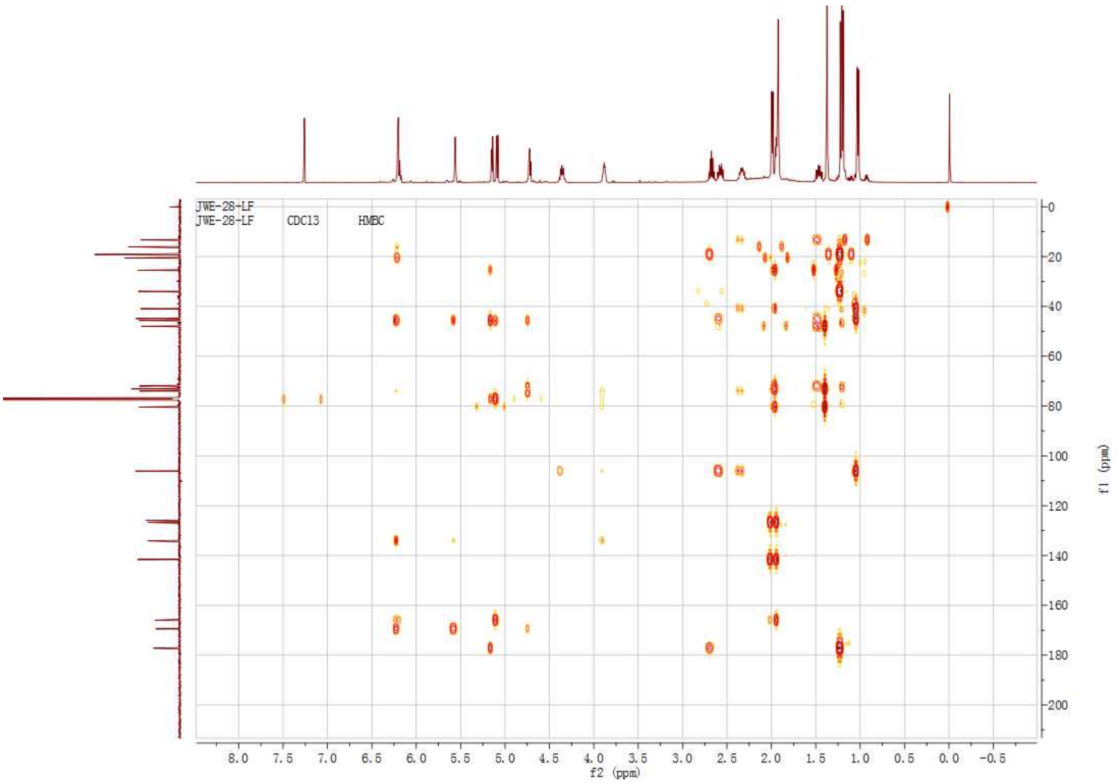

Fig. S4.5 HMBC spectrum (500 MHz) of (2*R*, 5*S*)-cardivarolide C (**4**) in $CDCl_3$

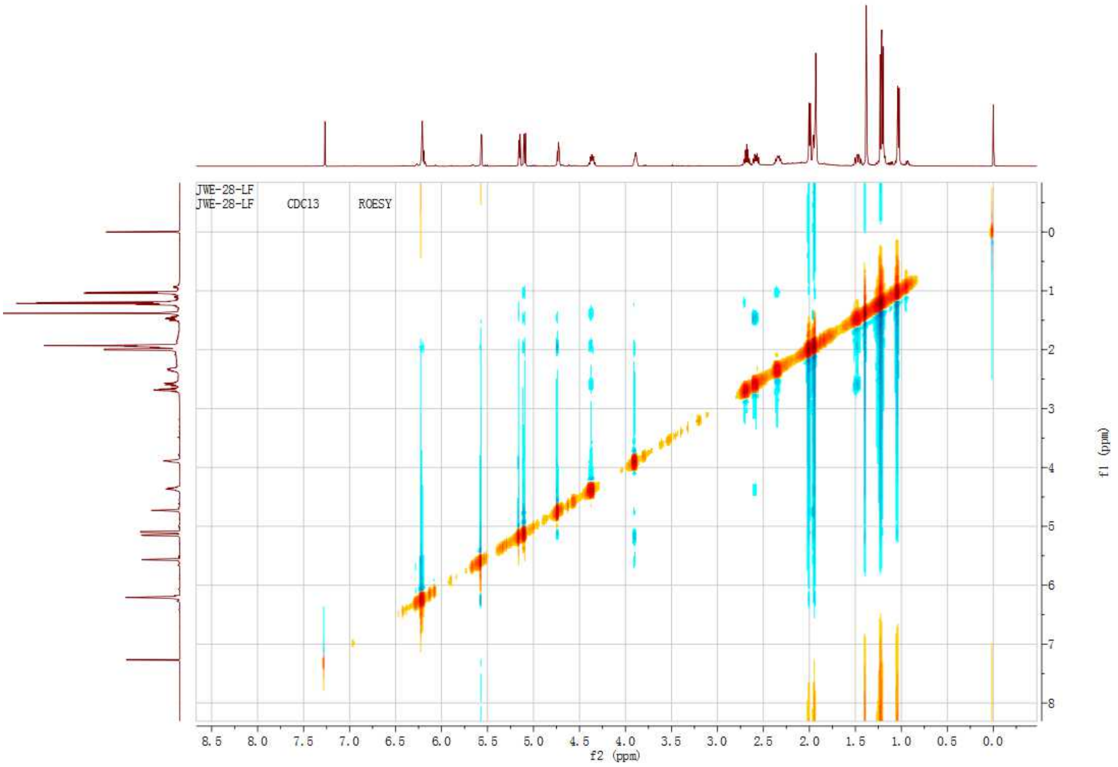

Fig. S4.6 ROESY spectrum (500 MHz) of (2*R*, 5*S*)-cardivarolide C (**4**) in $CDCl_3$

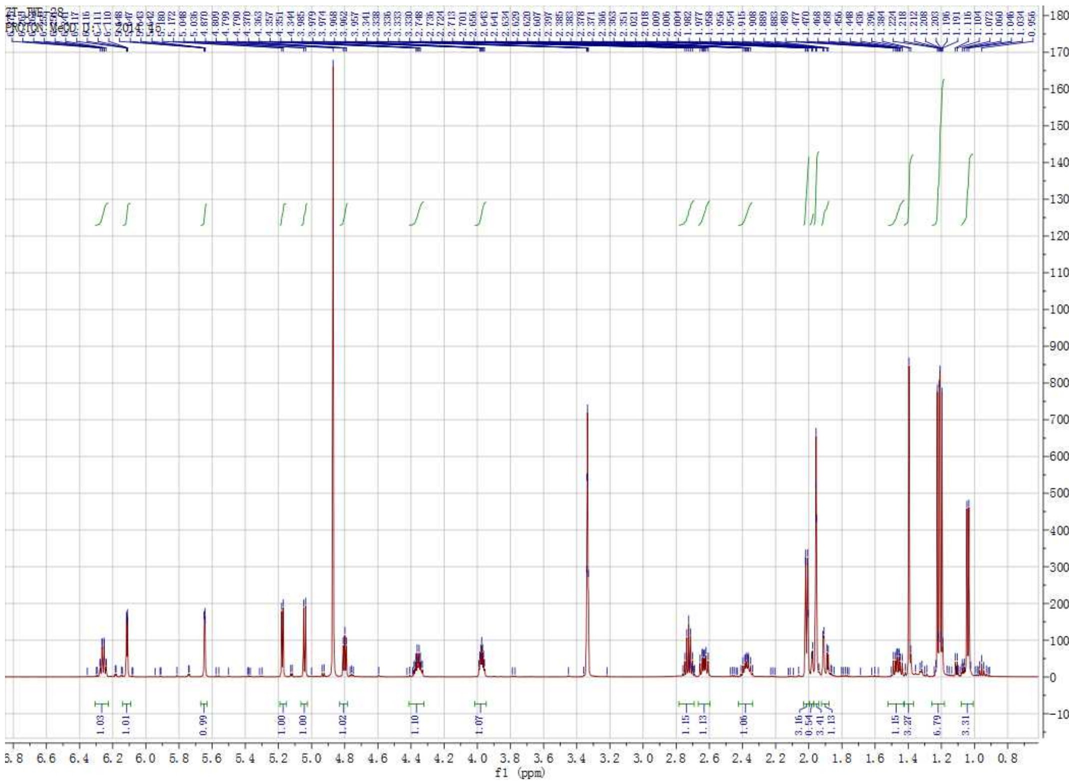

Fig. S4.7 ^{1}H NMR spectrum (600 MHz) of (2*R*, 5*S*)-cardivarolide C (**4**) in CD_3OD

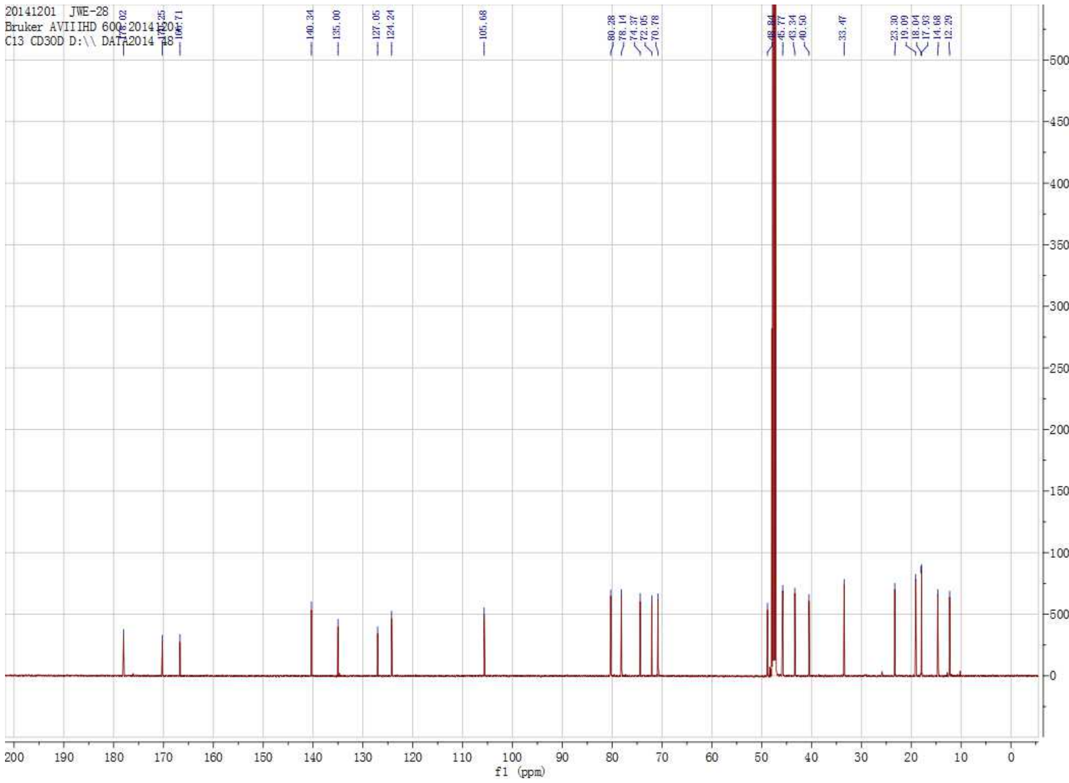

Fig. S4.8 ^{13}C NMR spectrum (150 MHz) of (2*R*, 5*S*)-cardivarolide C (**4**) in CD_3OD

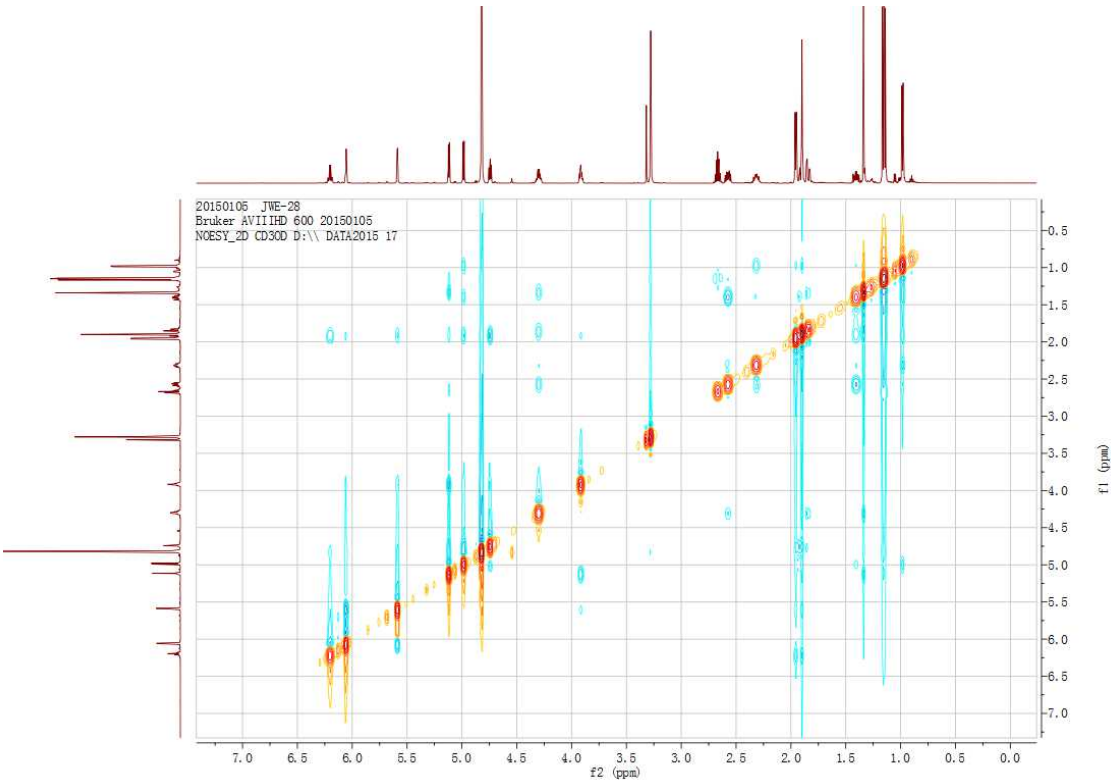

Fig. S4.9 NOESY spectrum (600 MHz) of (2*R*, 5*S*)-cardivarolide C (**4**) in CD_3OD

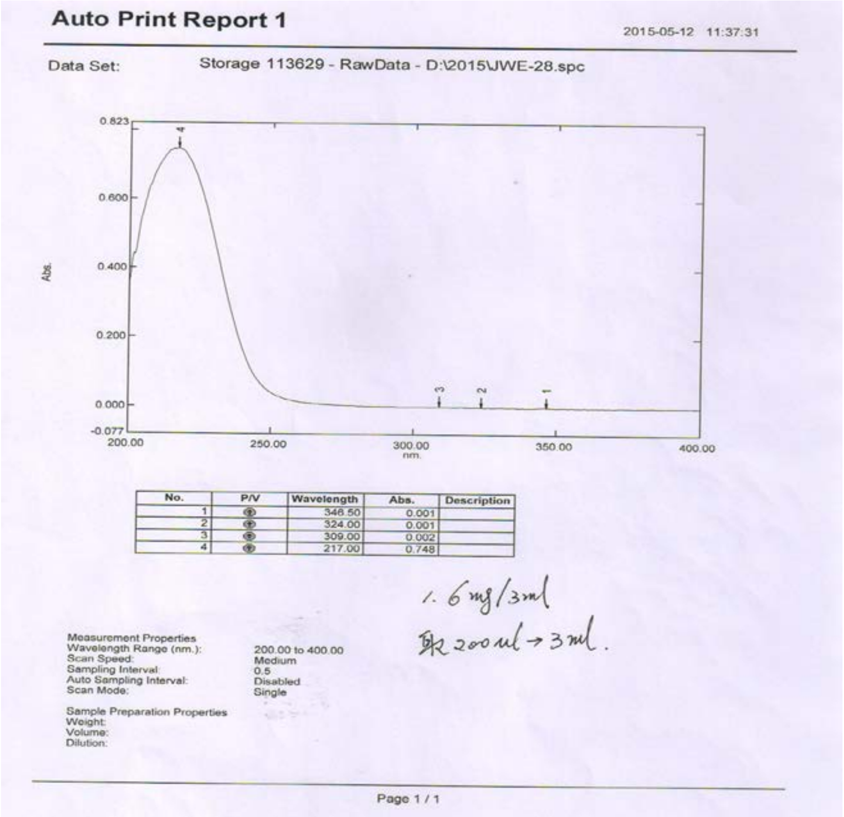

Fig. S4.10 UV spectrum of (2*R*, 5*S*)-cardivarolide C (**4**)

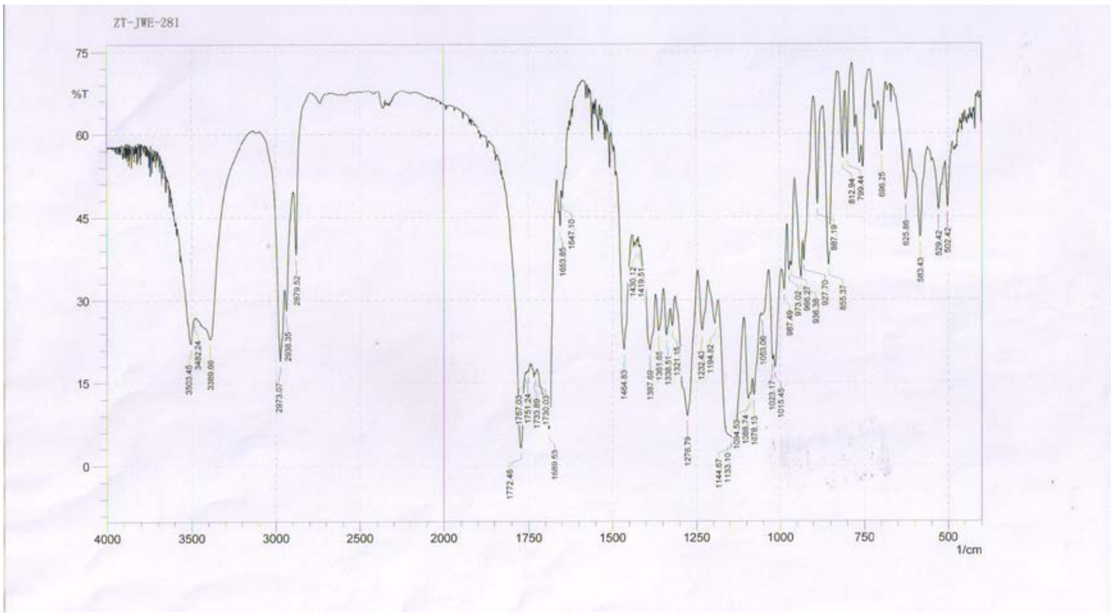

Fig. S4.11 IR spectrum of (2*R*, 5*S*)-cardivarolide C (**4**)

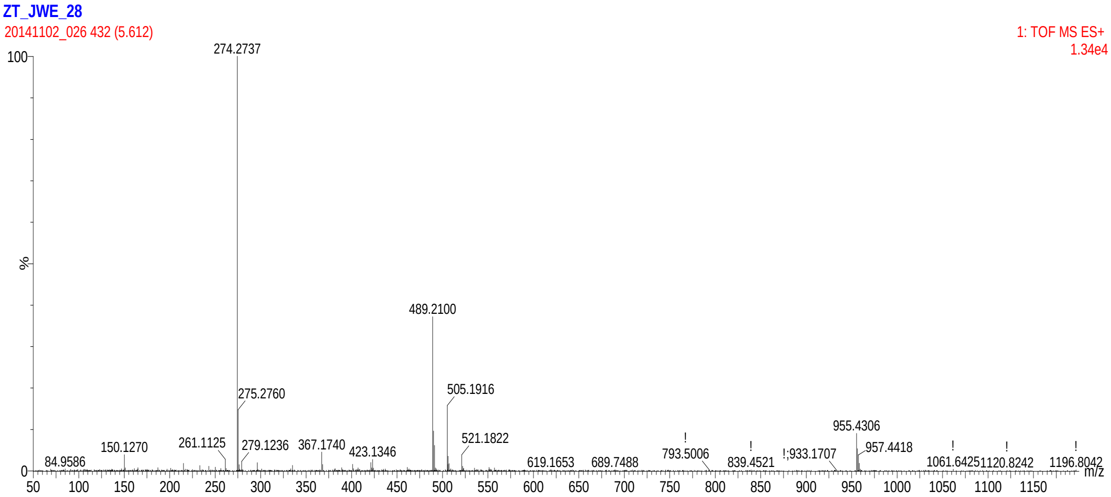

Fig. S4.12 HRESIMS spectrum of (2*R*, 5*S*)-cardivarolide C (**4**)

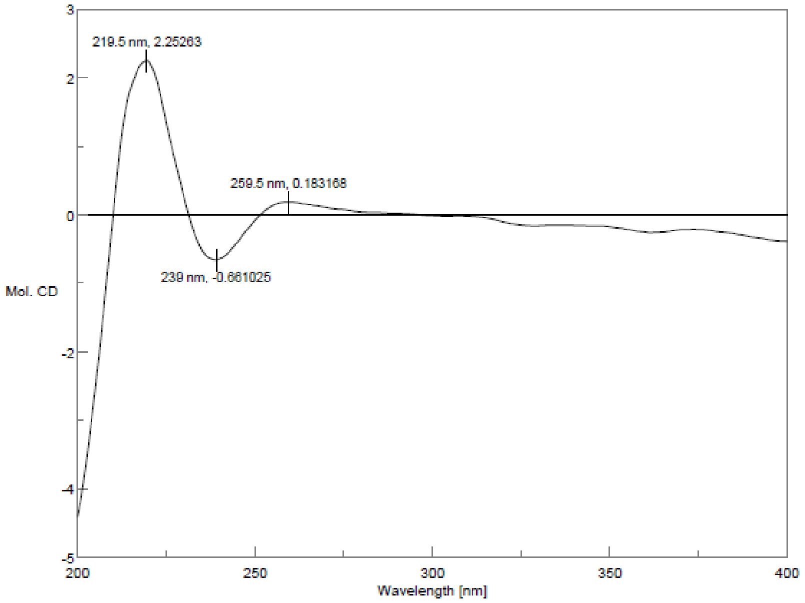

Fig. S3.13 CD spectrum of (2*R*, 5*S*)-cardivarolide C (**4**)

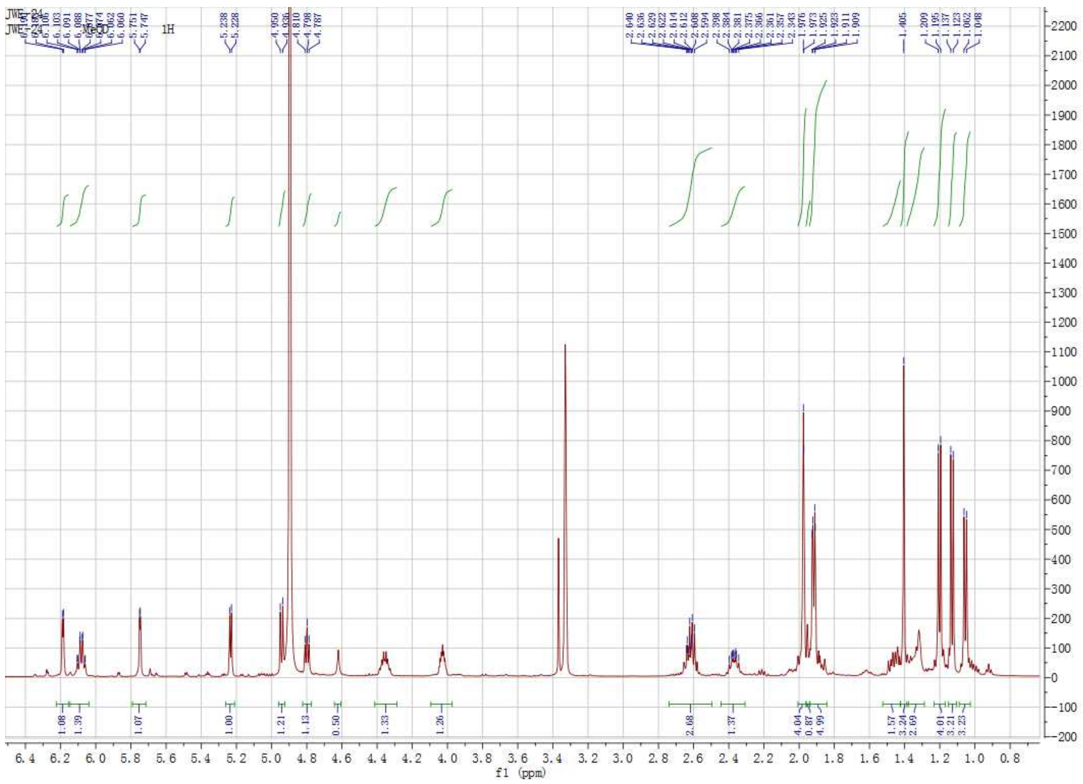

Fig. S5.1 ^{1}H NMR spectrum (500 MHz) of ineupatolide A (**5**) in CD_3OD

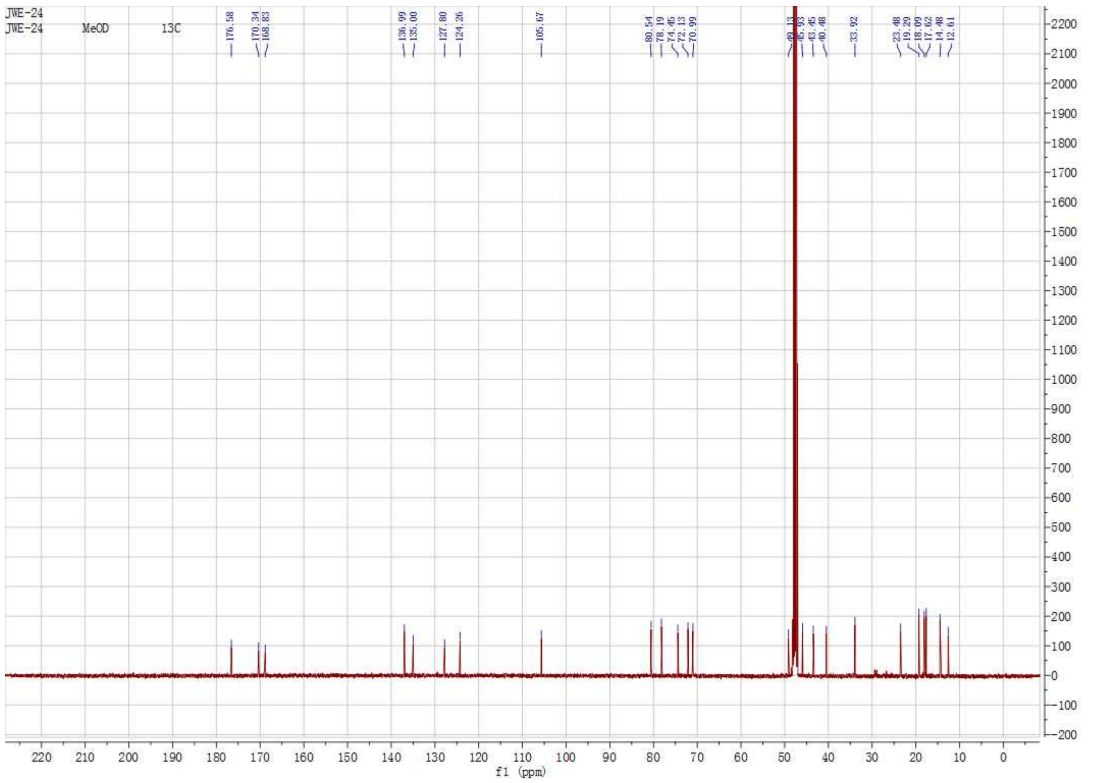

Fig. S5.2 ^{13}C NMR spectrum (125 MHz) of ineupatolide A (**5**) in CD_3OD

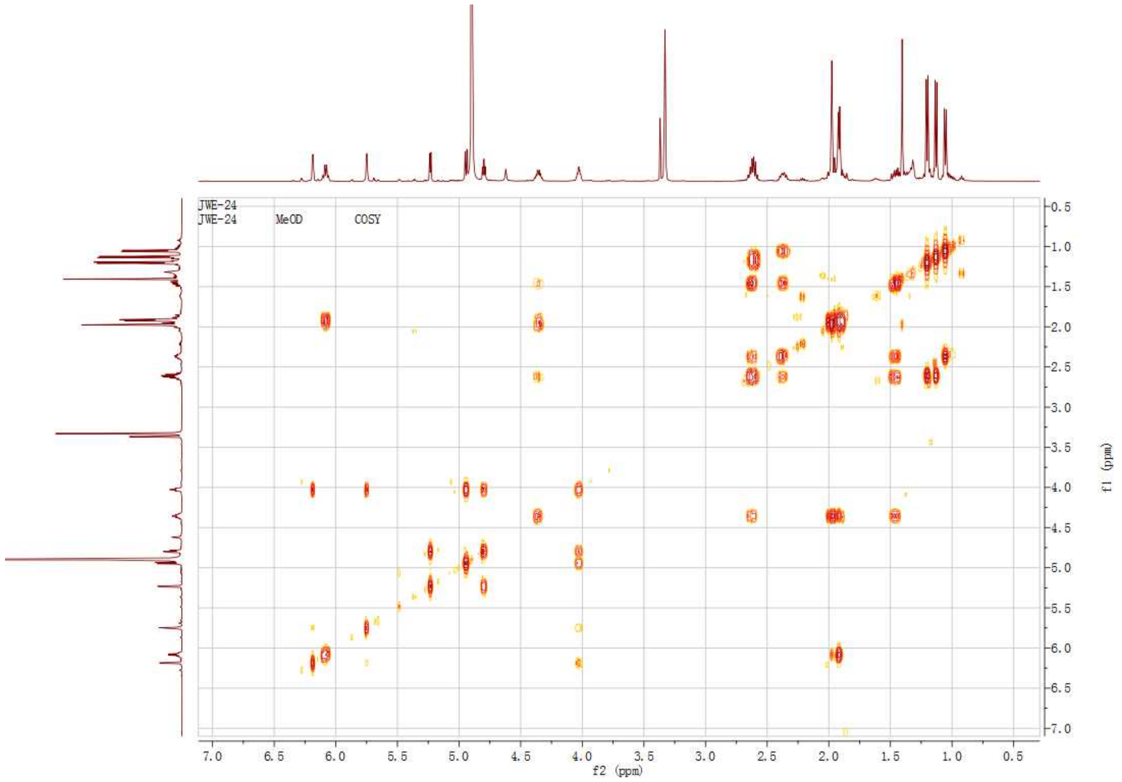

Fig. S5.3 ^{1}H-^{1}H COSY spectrum (500 MHz) of ineupatolide A (**5**) in CD_3OD

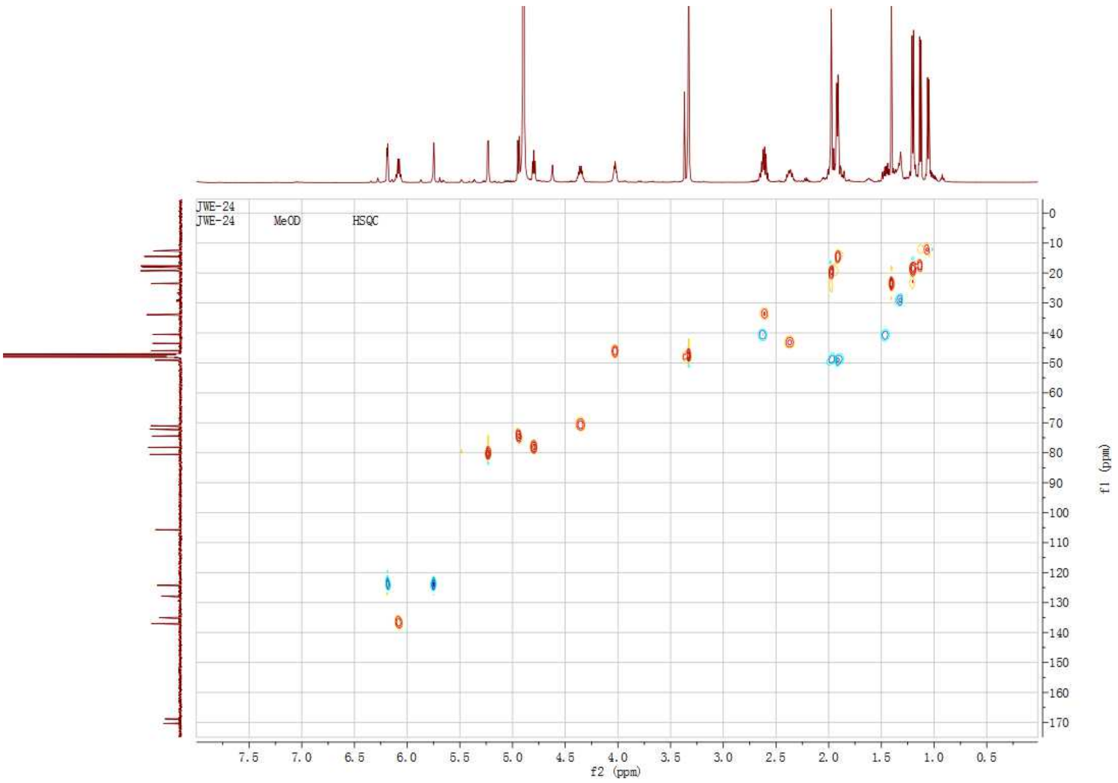

Fig. S5.4 HSQC spectrum (500 MHz) of ineupatolide A (**5**) in CD_3OD

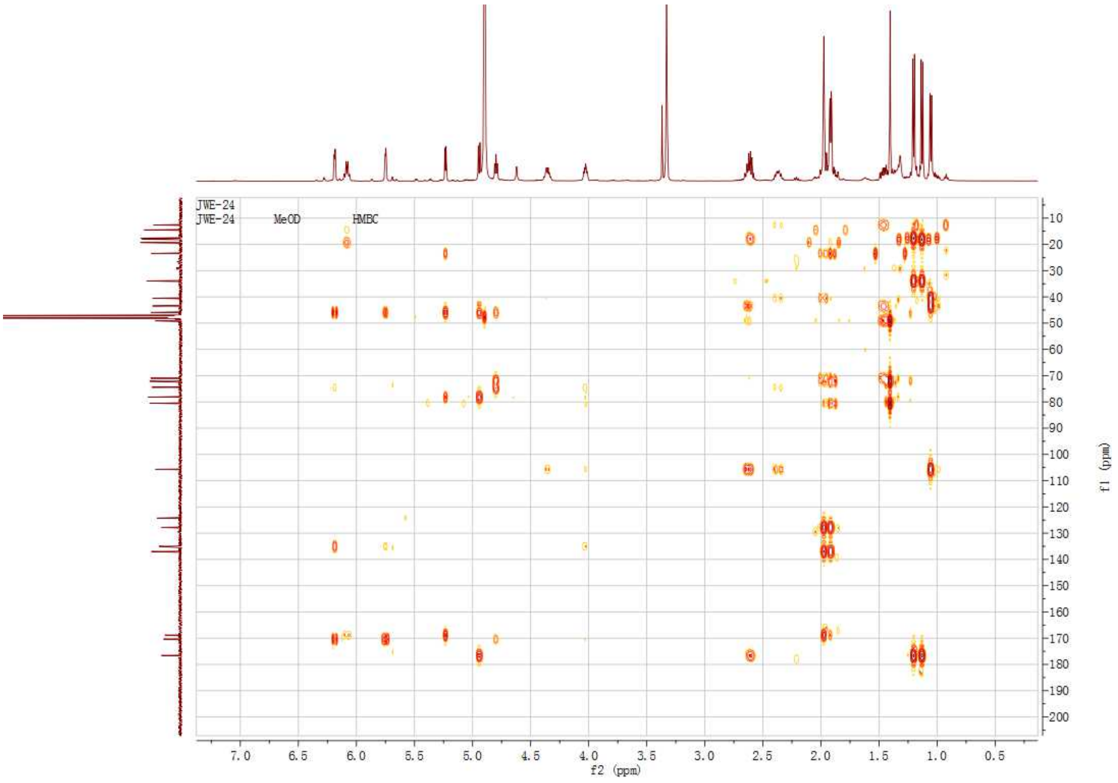

Fig. S5.5 HMBC spectrum (500 MHz) of ineupatolide A (**5**) in CD_3OD

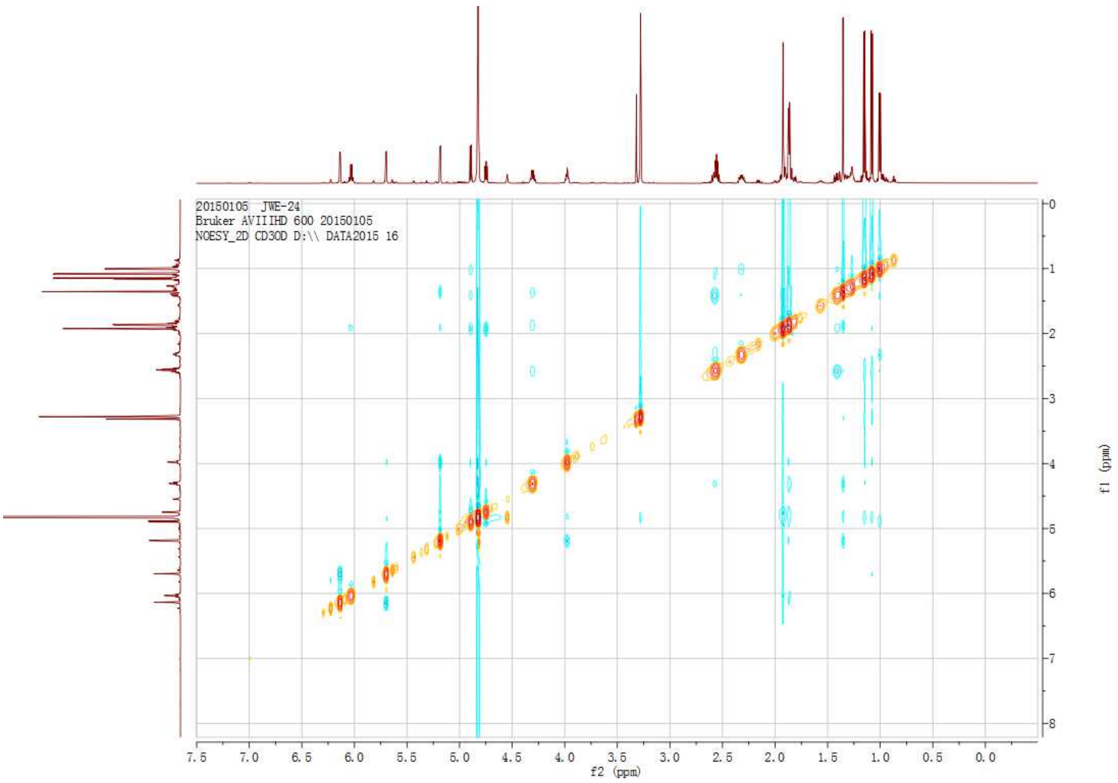

Fig. S5.6 NOESYspectrum (600 MHz) of ineupatolide A (**5**) in CD_3OD

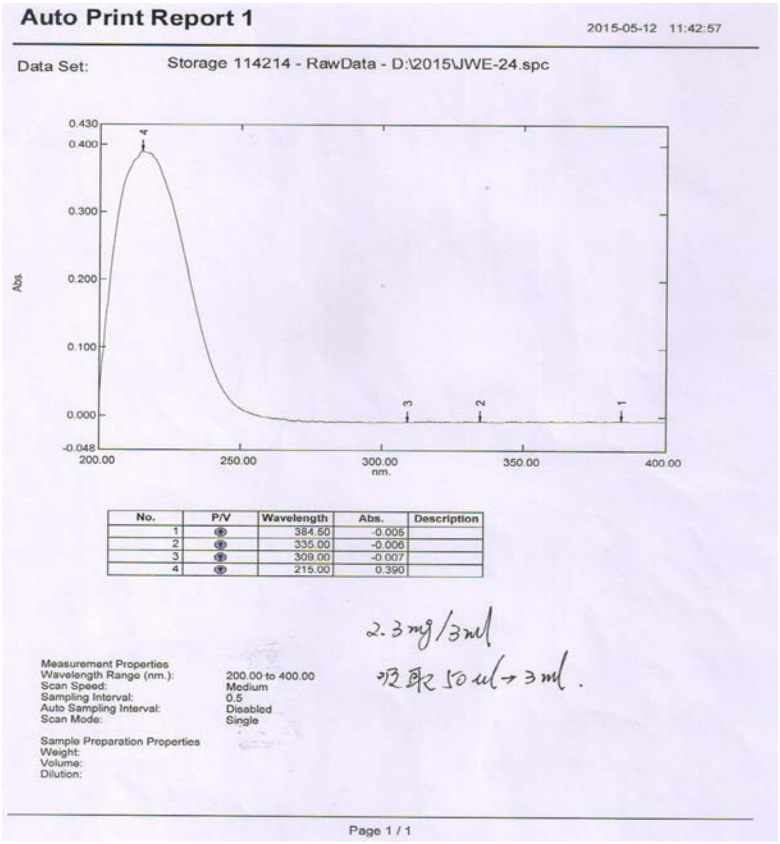

Fig. S5.7 UV spectrum of ineupatolide A (**5**)

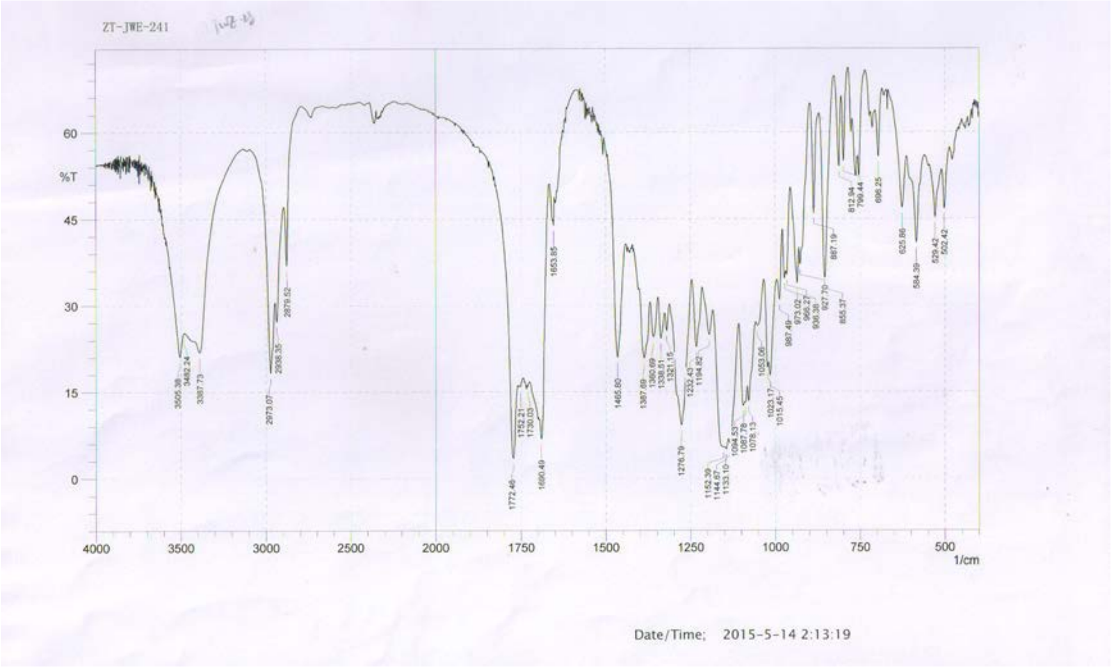

Fig. S5.8 IR spectrum of ineupatolide A (**5**)

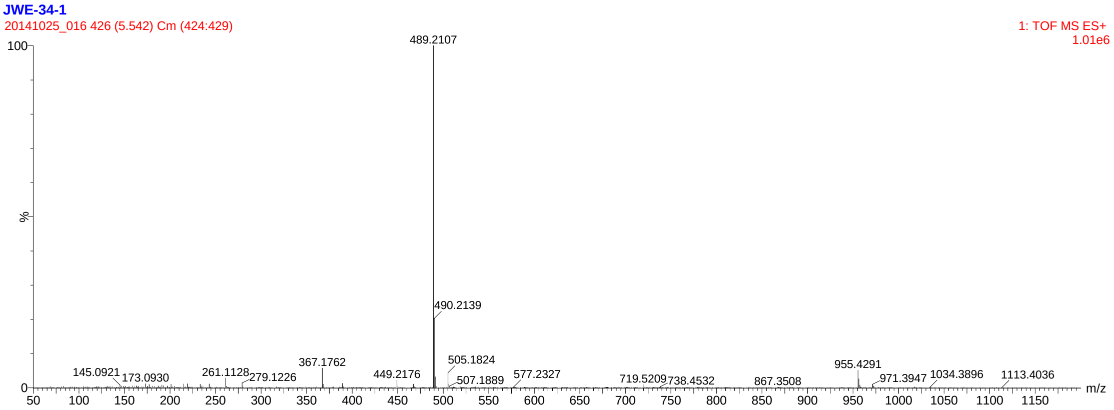

Fig. S5.9 HRESIMS spectrum of ineupatolide A (**5**)

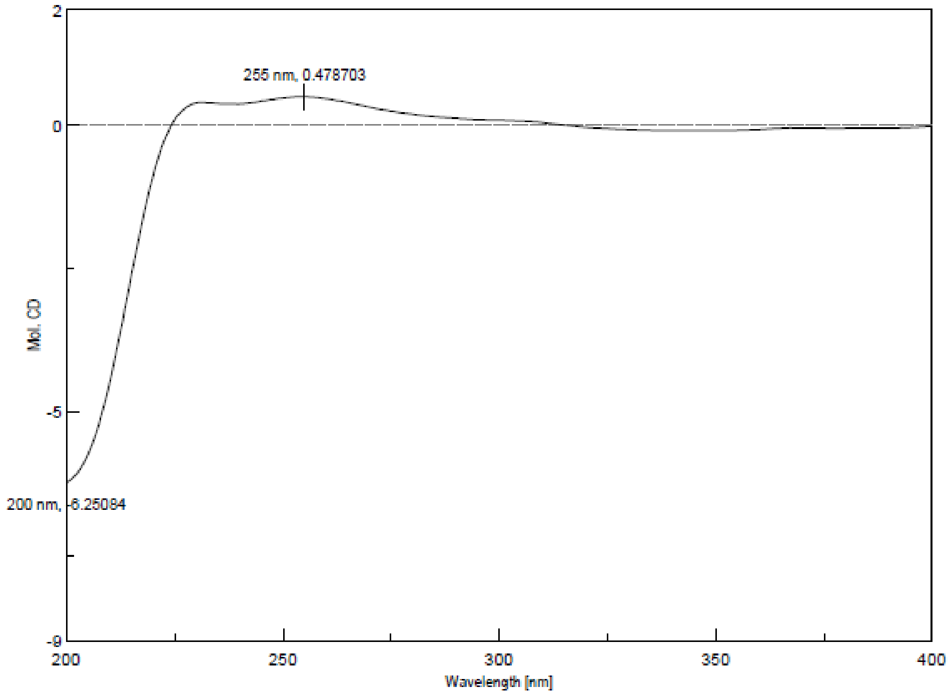

Fig. S5.10 CD spectrum of ineupatolide A (**5**)

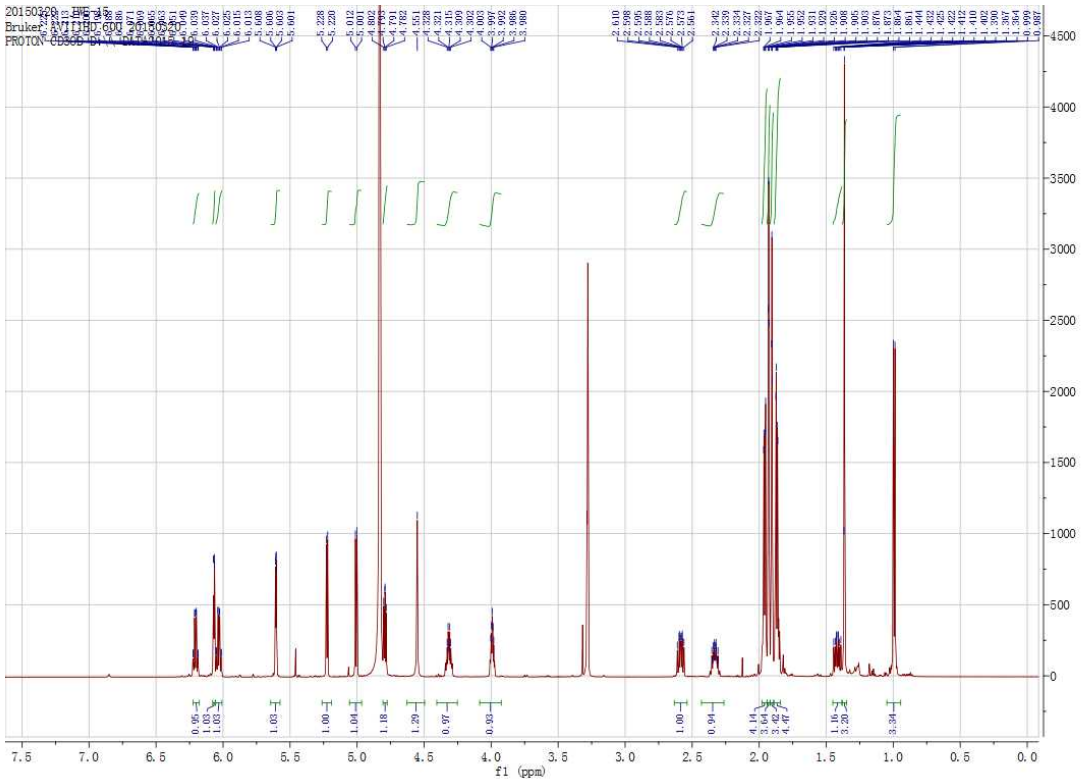

Fig. S6.1 ^{1}H NMR spectrum (600 MHz) of (2*R*, 5*S*)-cardivarolide D (**6**) in CD_3OD

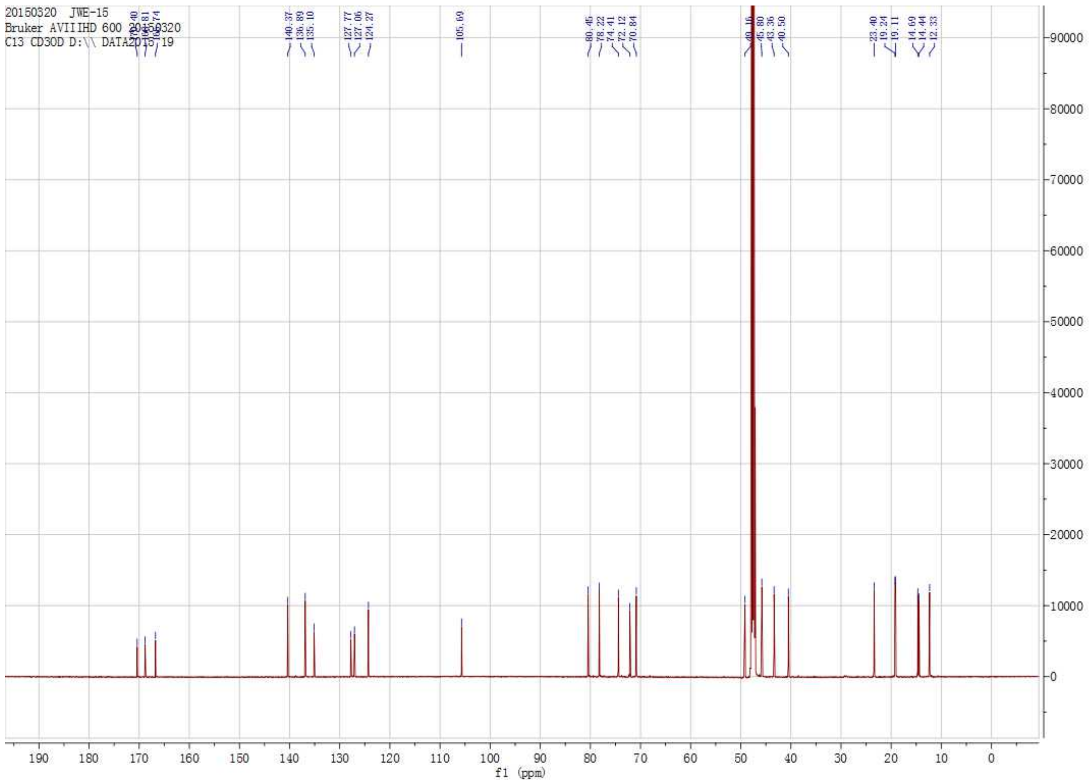

Fig. S6.2 ^{13}C NMR spectrum (150 MHz) of (2*R*, 5*S*)-cardivarolide D (**6**) in CD_3OD

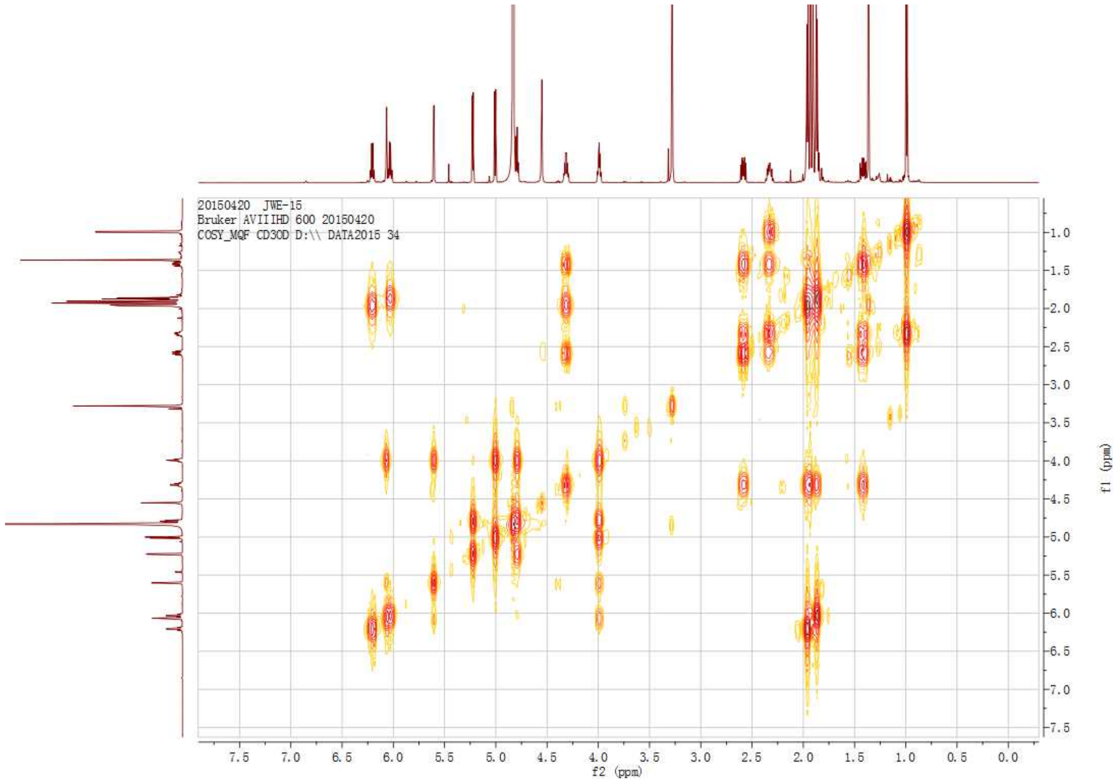

Fig. S6.3 ^{1}H-^{1}H COSY spectrum (600 MHz) of (2*R*, 5*S*)-cardivarolide D (**6**) in CD_3OD

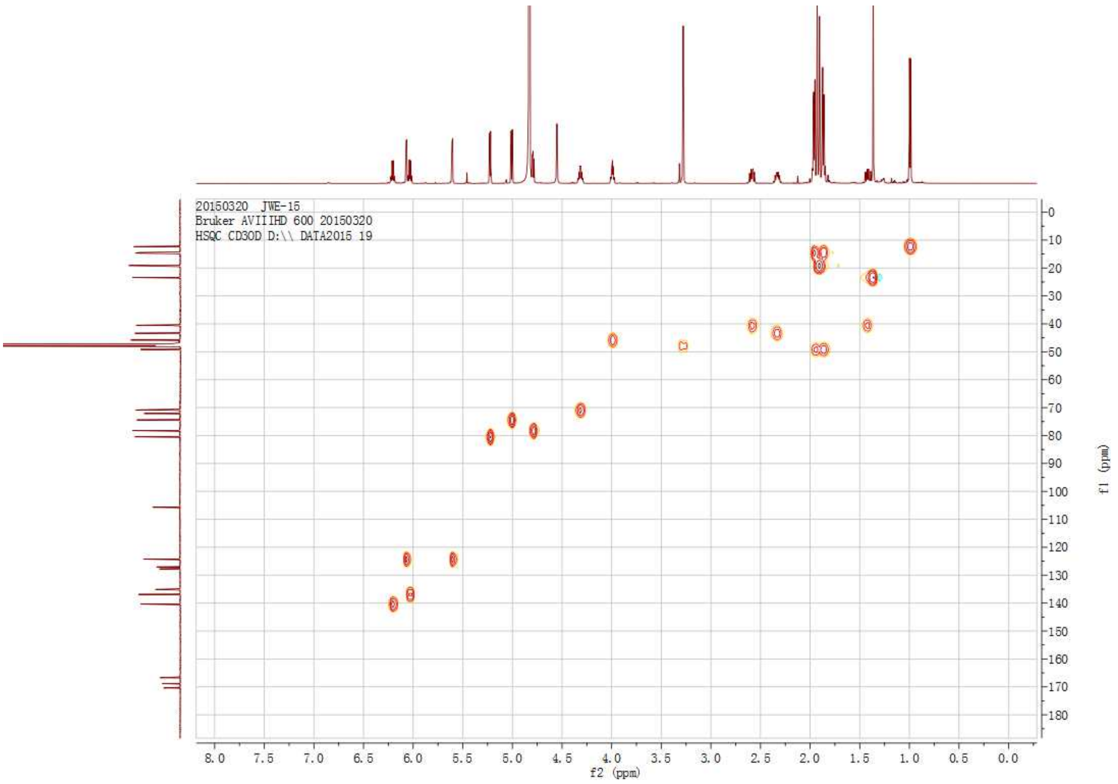

Fig. S6.4 HSQC spectrum (600 MHz) of (2*R*, 5*S*)-cardivarolide D (**6**) in CD_3OD

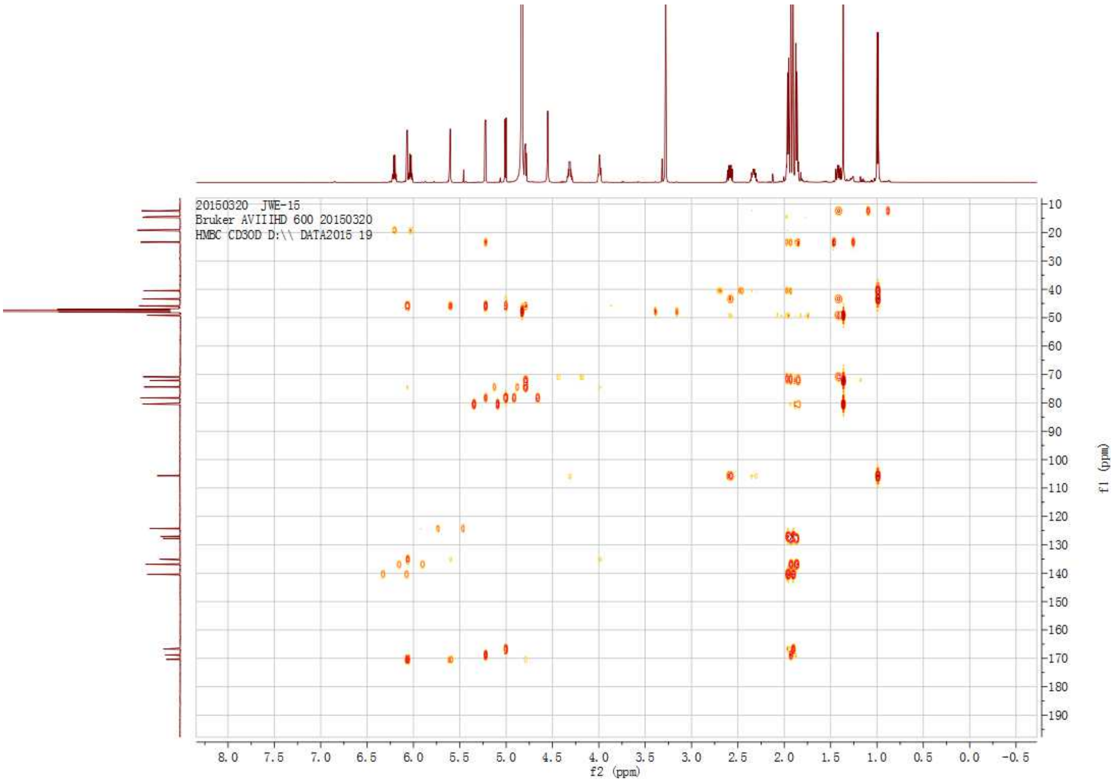

Fig. S6.5 HMBC spectrum (600 MHz) of (2*R*, 5*S*)-cardivarolide D (**6**) in CD_3OD

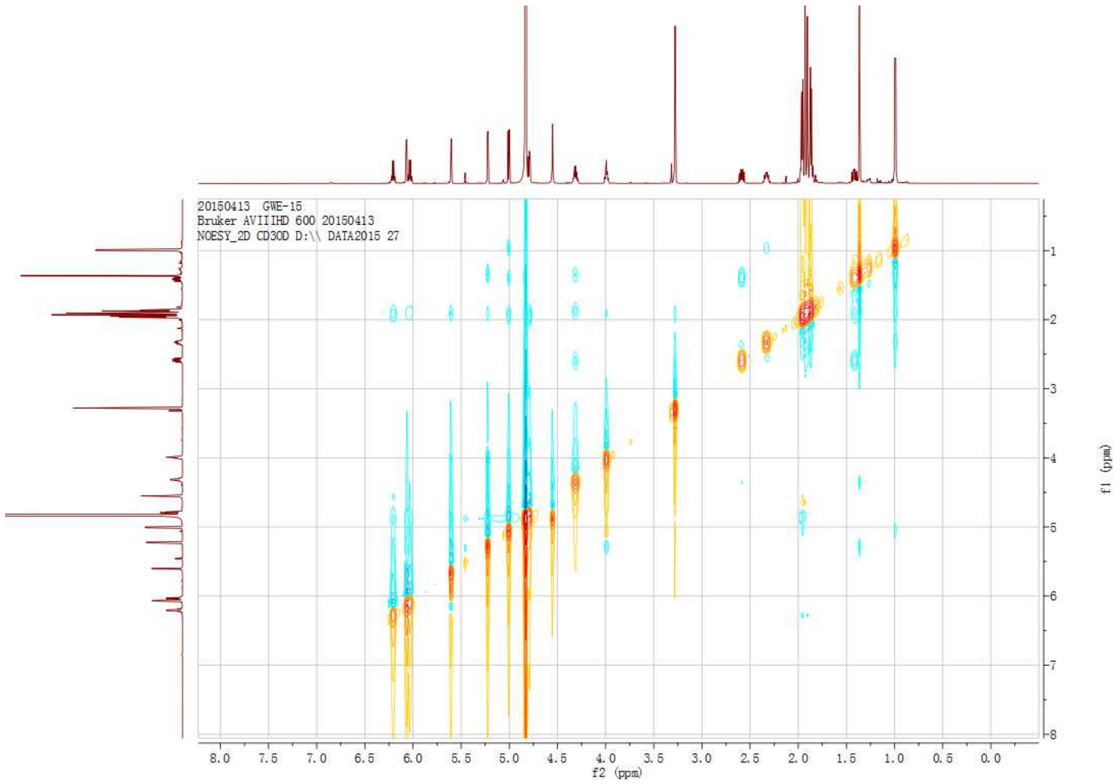

Fig. S6.6 NOESY spectrum (600 MHz) of (2*R*, 5*S*)-cardivarolide D (**6**) in CD_3OD

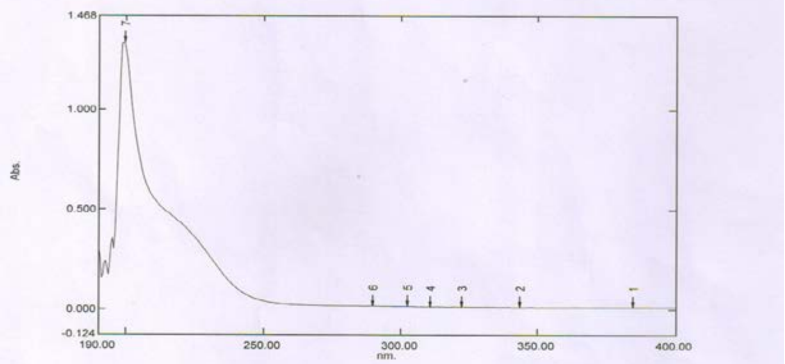

Auto Print Report 1 2015-05-21 15:35:26

Data Set: Storage 153420 - RawData - D:\2015\JWE-15.spc

No.	P/V	Wavelength	Abs.	Description
1	⊕	384.50	0.010	
2	⊕	343.50	0.010	
3	⊕	322.50	0.013	
4	⊕	311.00	0.013	
5	⊕	302.50	0.015	
6	⊕	290.00	0.016	
7	⊕	199.50	1.335	

Measurement Properties
Wavelength Range (nm.): 190.00 to 400.00
Scan Speed: Medium
Sampling Interval: 0.5
Auto Sampling Interval: Disabled
Scan Mode: Single

Sample Preparation Properties
Weight:
Volume:
Dilution:

样品 4.5 mg/4 ml
旋光: -0.048°
UV: 吸取 30 ul → 3 ml

Page 1 / 1

Fig. S6.7 UV spectrum of (2*R*, 5*S*)-cardivarolide D (**6**)

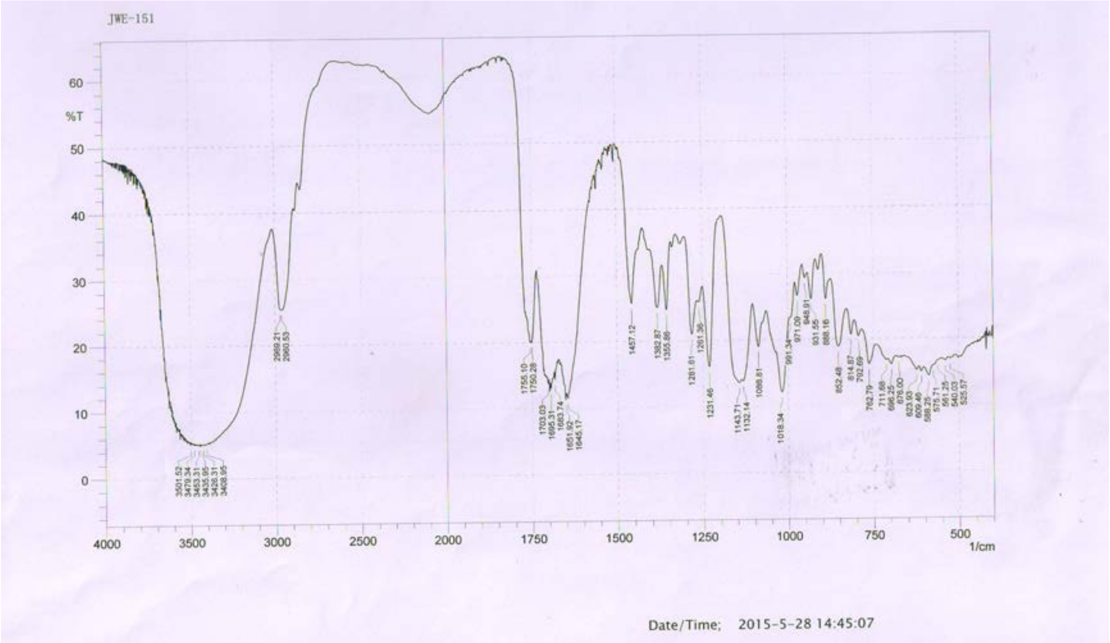

Fig. S6.8 IR spectrum of (2*R*, 5*S*)-cardivarolide D (**6**)

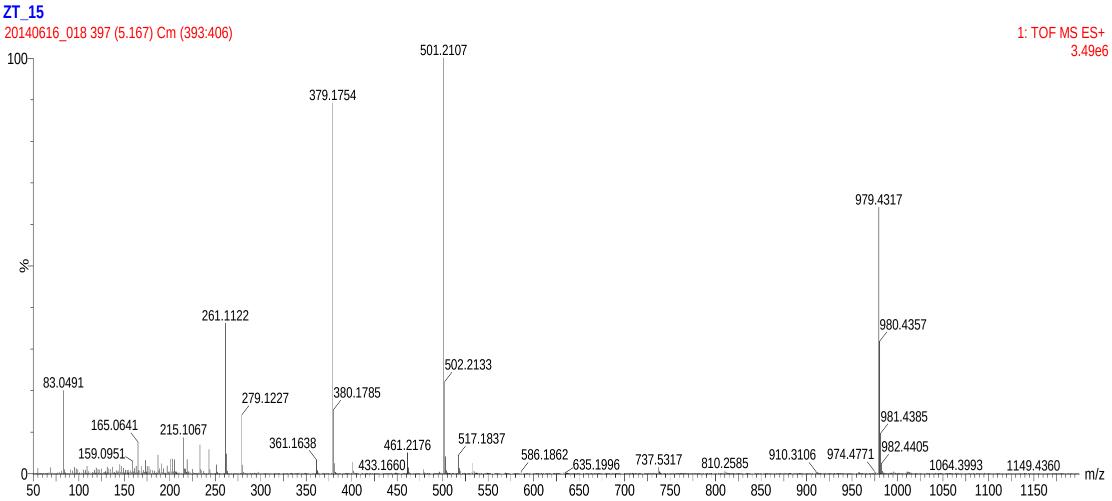

Fig. S6.9 HRESIMS spectrum of (2*R*, 5*S*)-cardivarolide D (**6**)

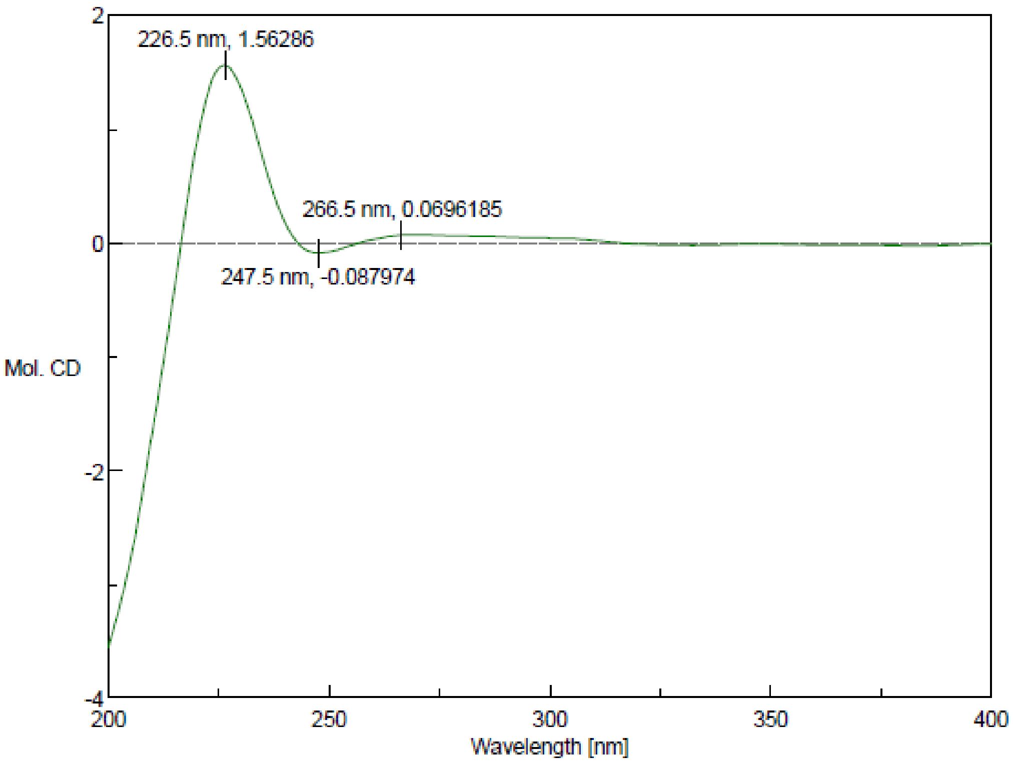

Fig. S6.10 CD spectrum of (2*R*, 5*S*)-cardivarolide D (**6**)

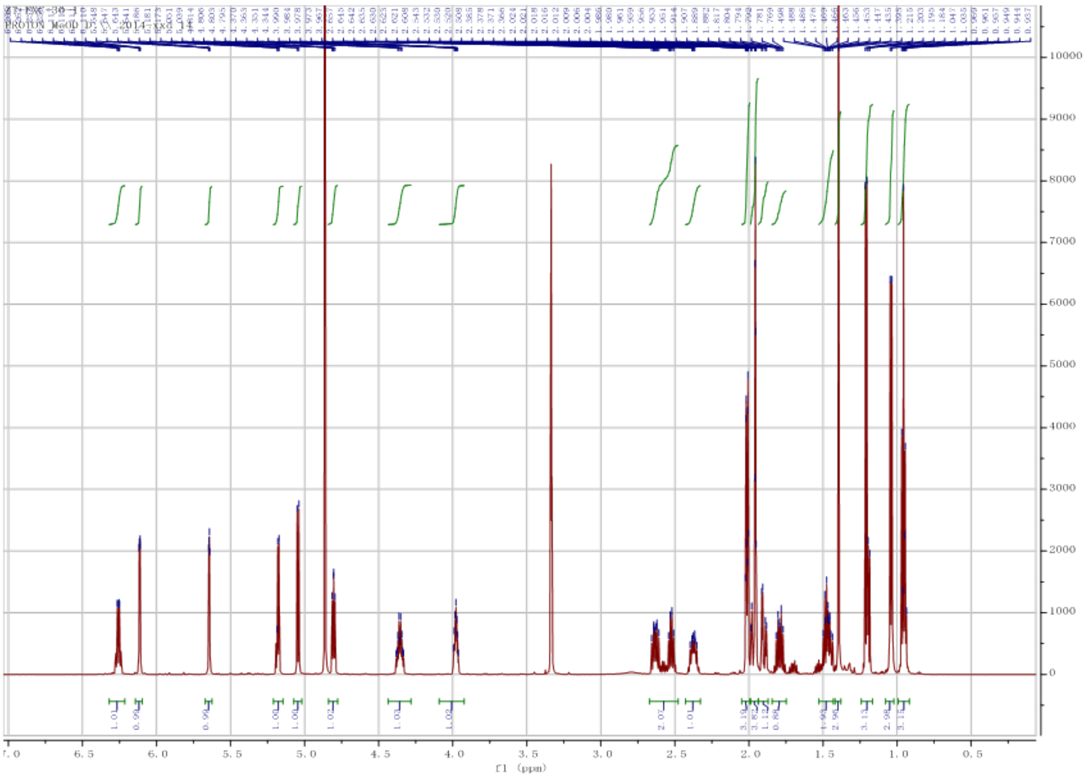

Fig. S7.1 ^{1}H NMR spectrum (600 MHz) of ineupatolide (**7**) in CD_3OD

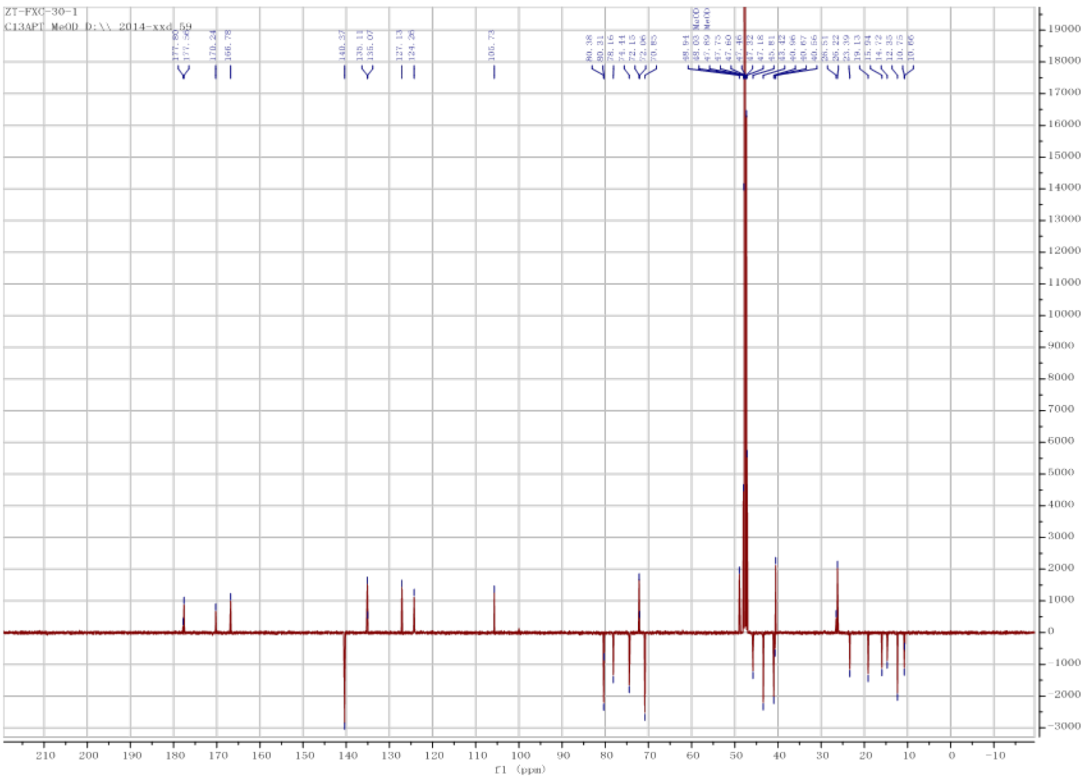

Fig. S7.2 ^{13}C NMR spectrum (150 MHz) of ineupatolide (**7**) in CD_3OD

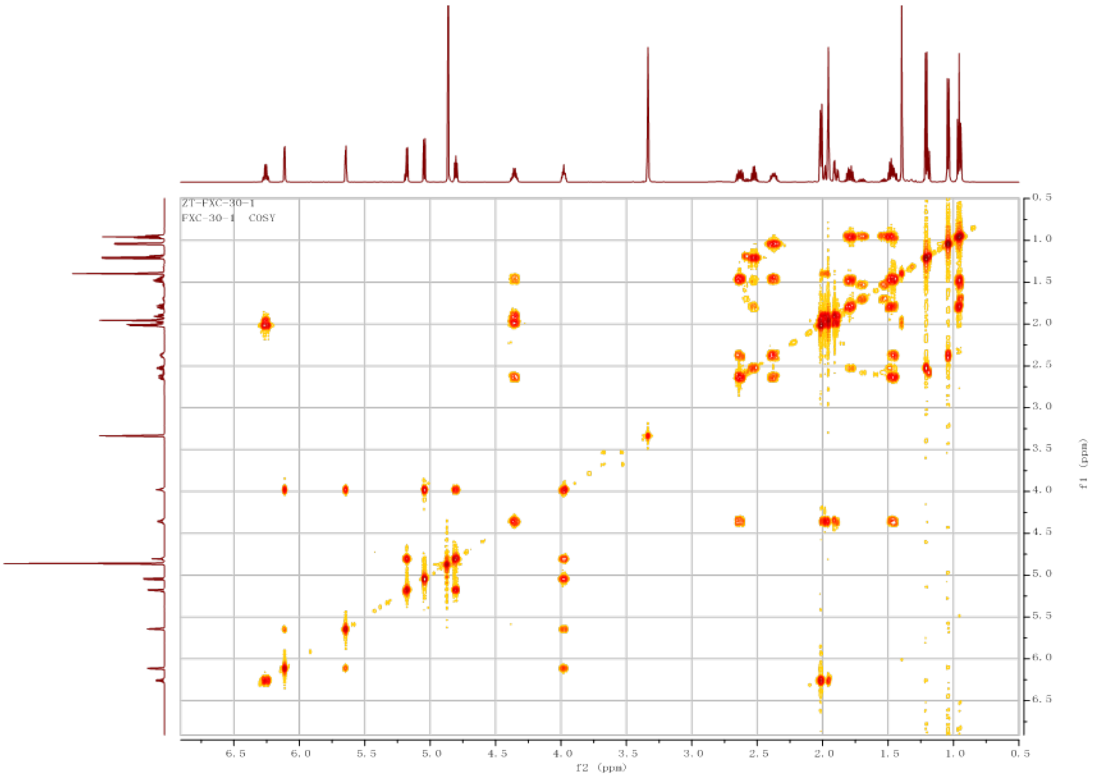

Fig. S7.3 ^{1}H-^{1}H COSY spectrum (600 MHz) of ineupatolide (**7**) in CD_3OD

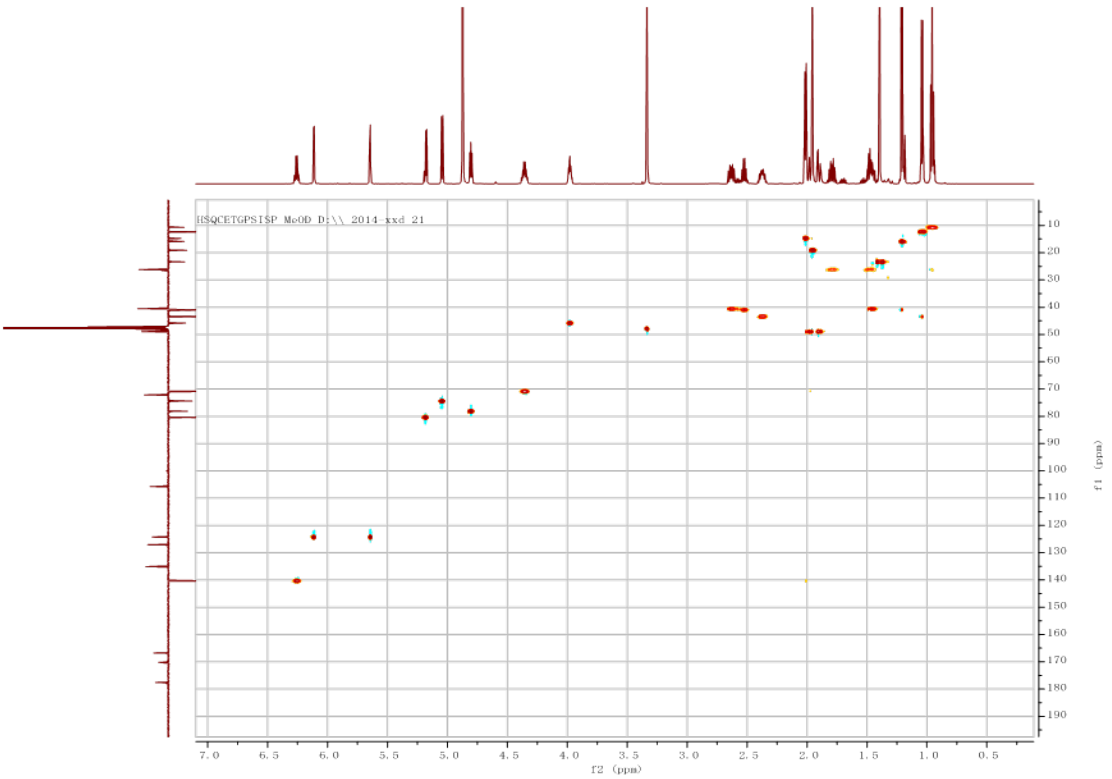

Fig. S7.4 HSQC spectrum (600 MHz) of ineupatolide (**7**) in CD_3OD

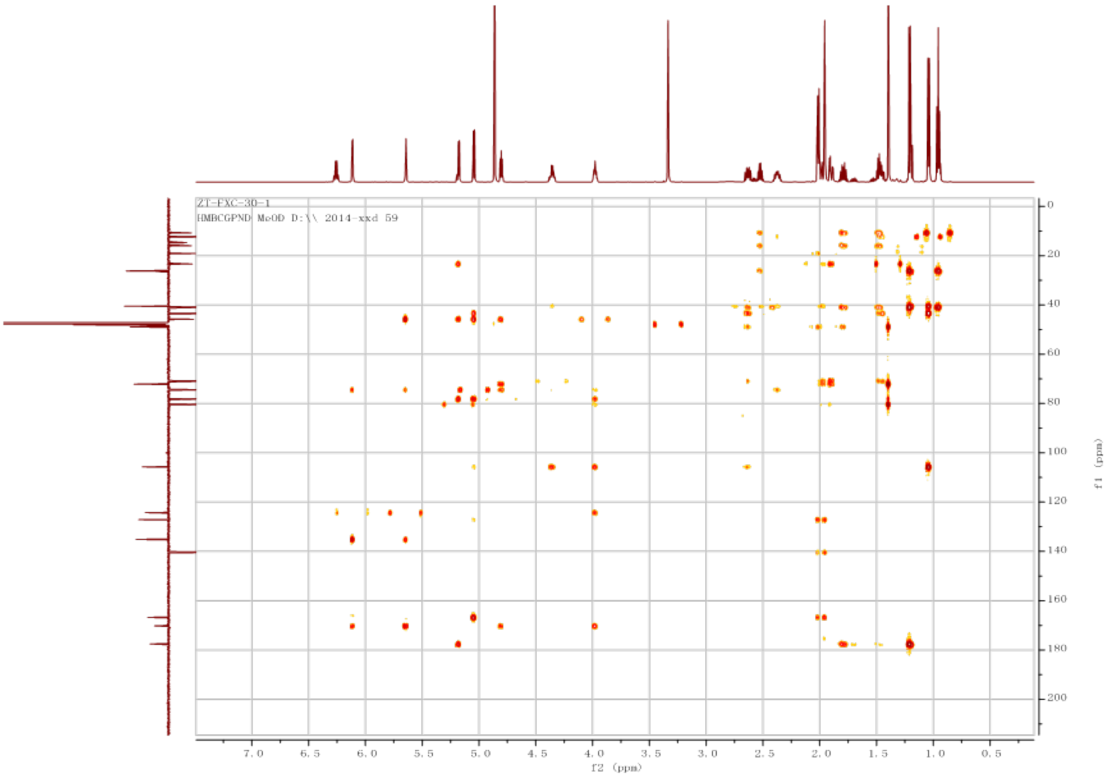

Fig. S7.5 HMBC spectrum (600 MHz) of ineupatolide (**7**) in CD_3OD

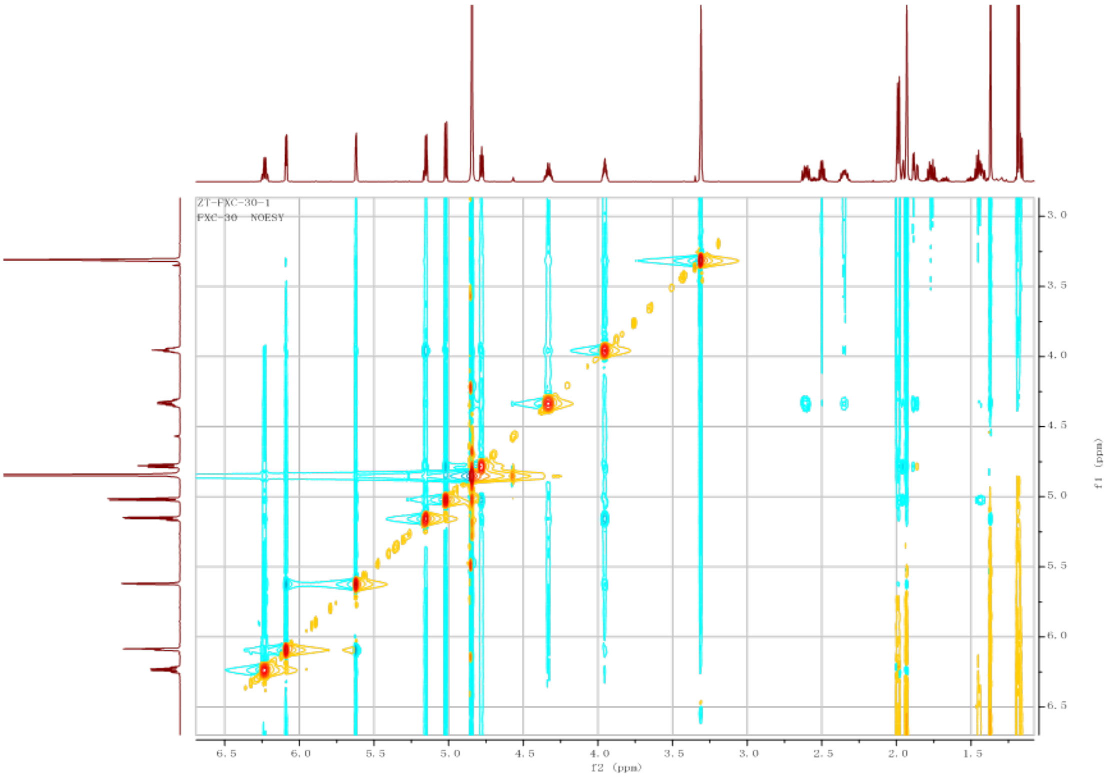

Fig. S7.6 NOESY spectrum (600 MHz) of ineupatolide (**7**) in CD_3OD

Auto Print Report 1

2016-05-22 10 20:06

Data Set: Storage 101823 - RawData - D:\2015\JWE-24a.spc

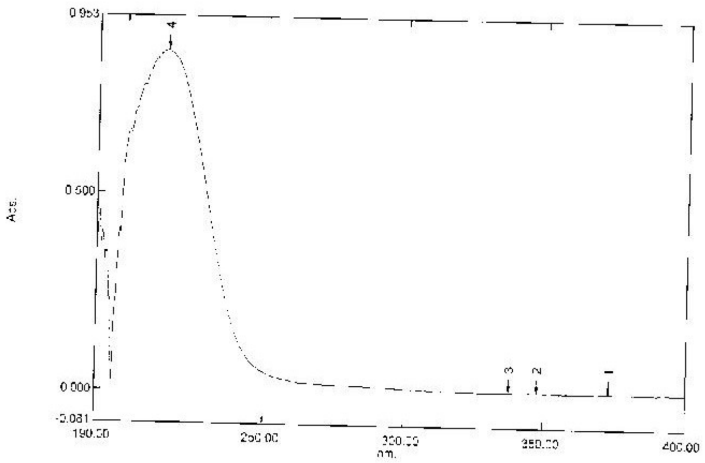

No.	P/V	Wavelength	Abs.	Description
1	[illegible]	373.00	0.007	
2	[illegible]	347.50	0.008	
3	[illegible]	337.50	0.007	
4	[illegible]	214.50	0.567	

Fig. S7.7 UV spectrum of ineupatolide (**7**)

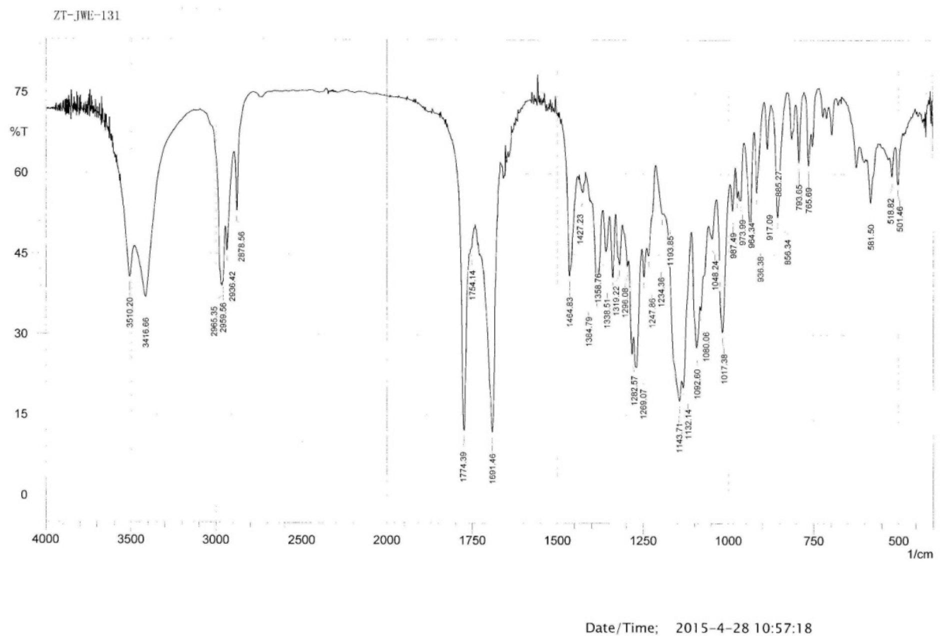

Fig. S7.8 IR spectrum of ineupatolide (**7**)

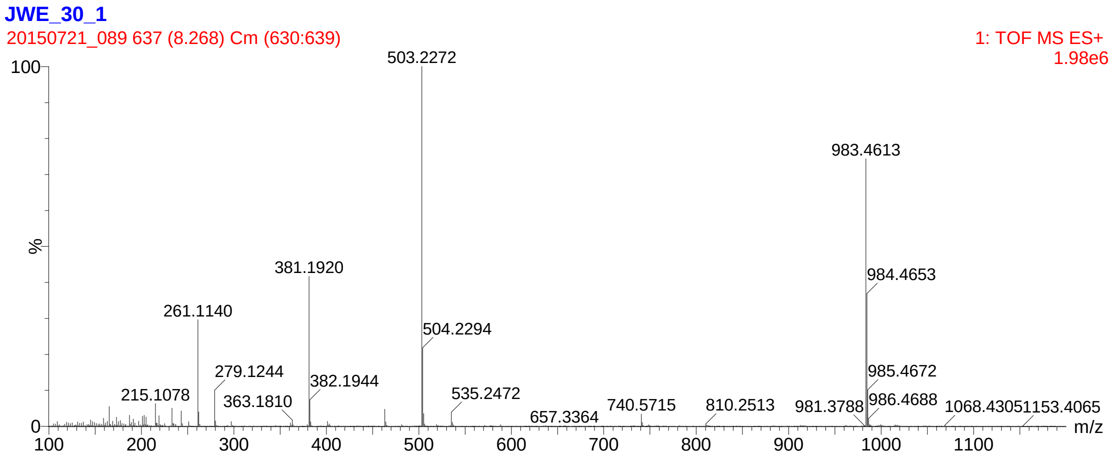

Fig. S7.9 HRESIMS spectrum of ineupatolide (**7**)

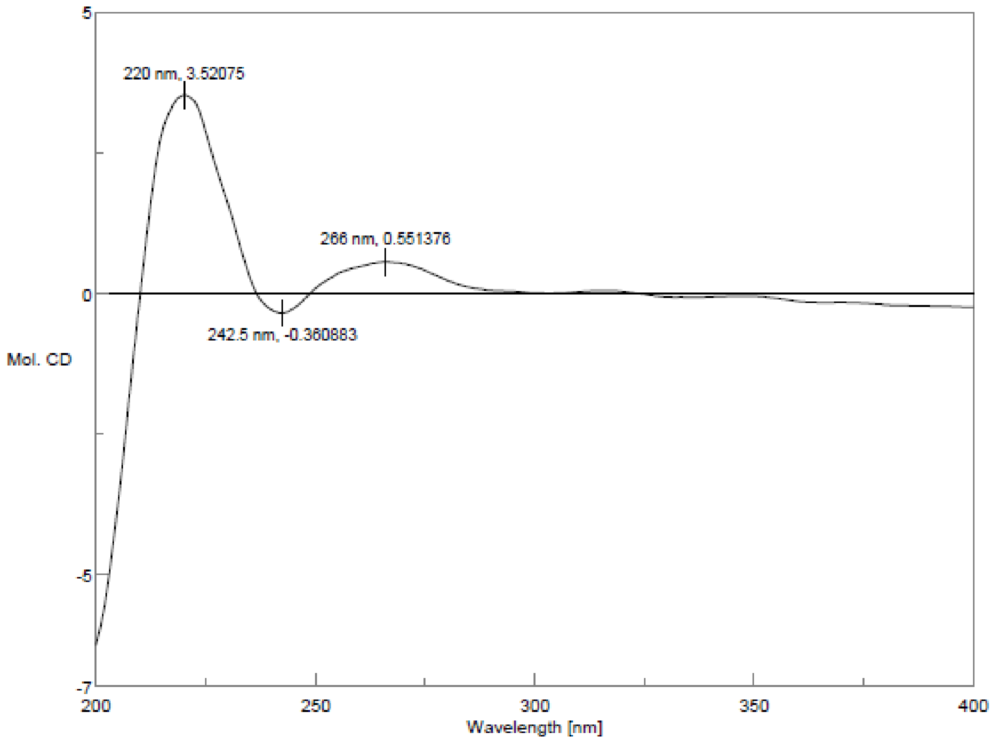

Fig. S7.10 CD spectrum of ineupatolide (**7**)

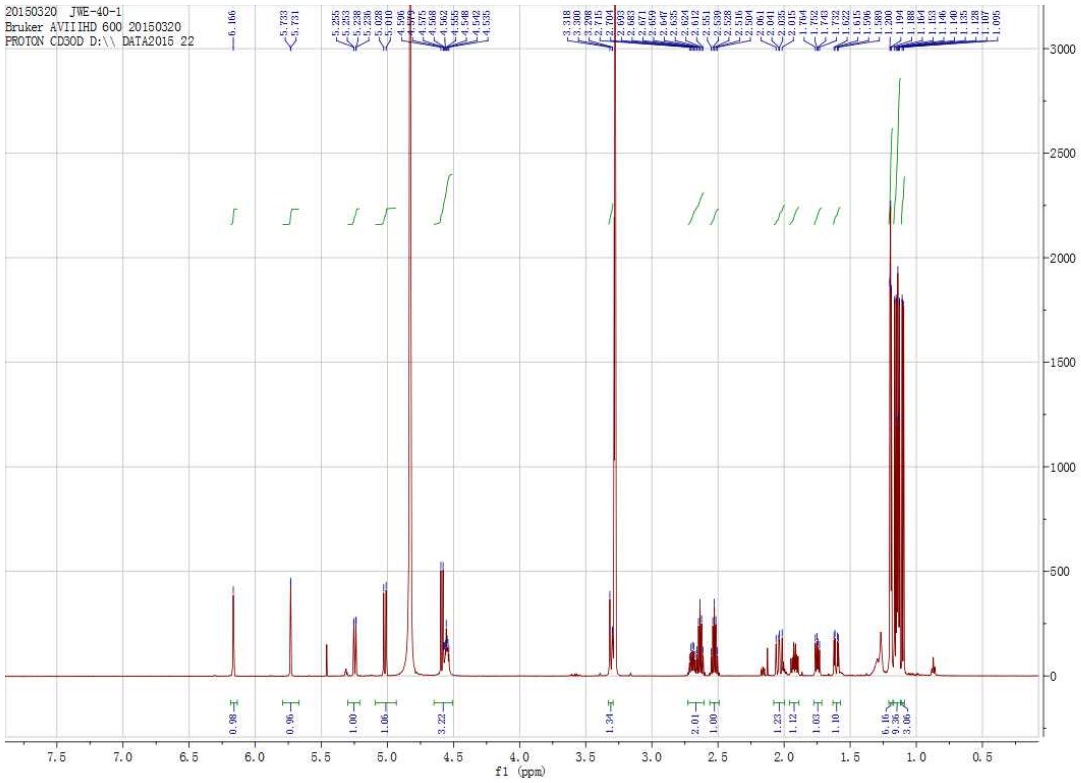

Fig. S8.1 ^{1}H NMR spectrum (600 MHz) of (2*S*, 5*R*)-isocardivarolide A (**8**) in CD_3OD

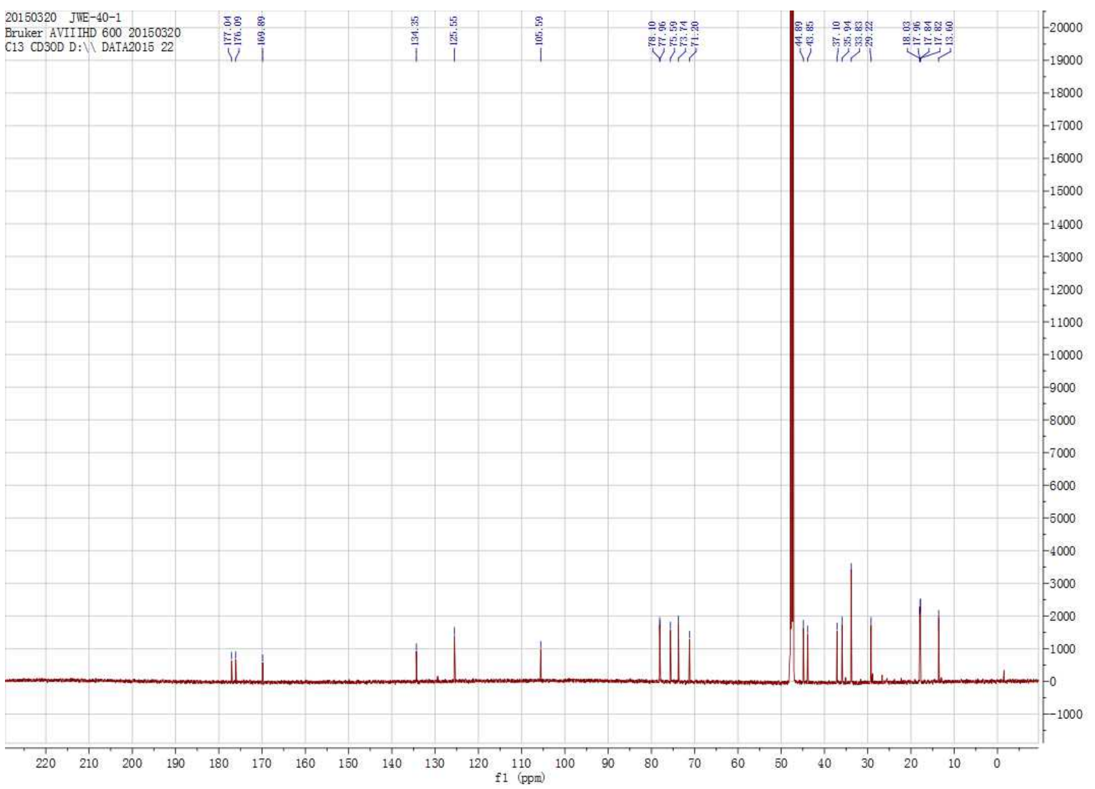

Fig. S8.2 ^{13}C NMR spectrum (150 MHz) of (2*S*, 5*R*)-isocardivarolide A (**8**) in CD_3OD

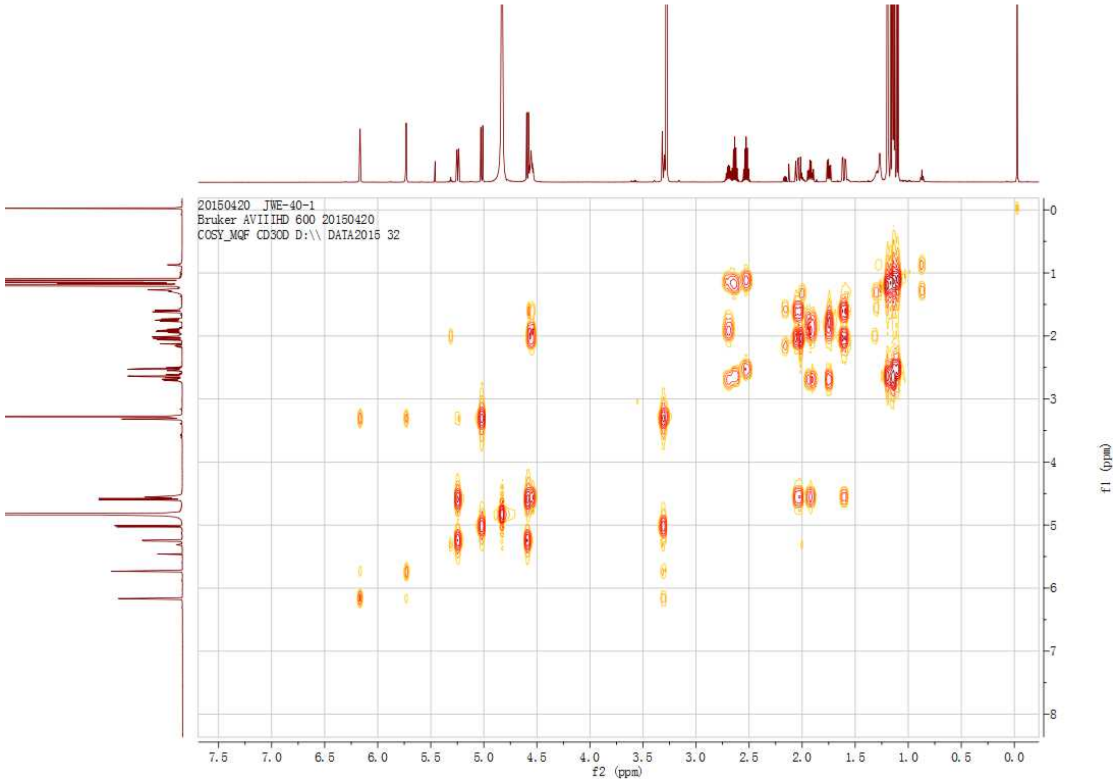

Fig. S8.3 ^{1}H-^{1}H COSY spectrum (600 MHz) of (2*S*, 5*R*)-isocardivarolide A (**8**) in CD_3OD

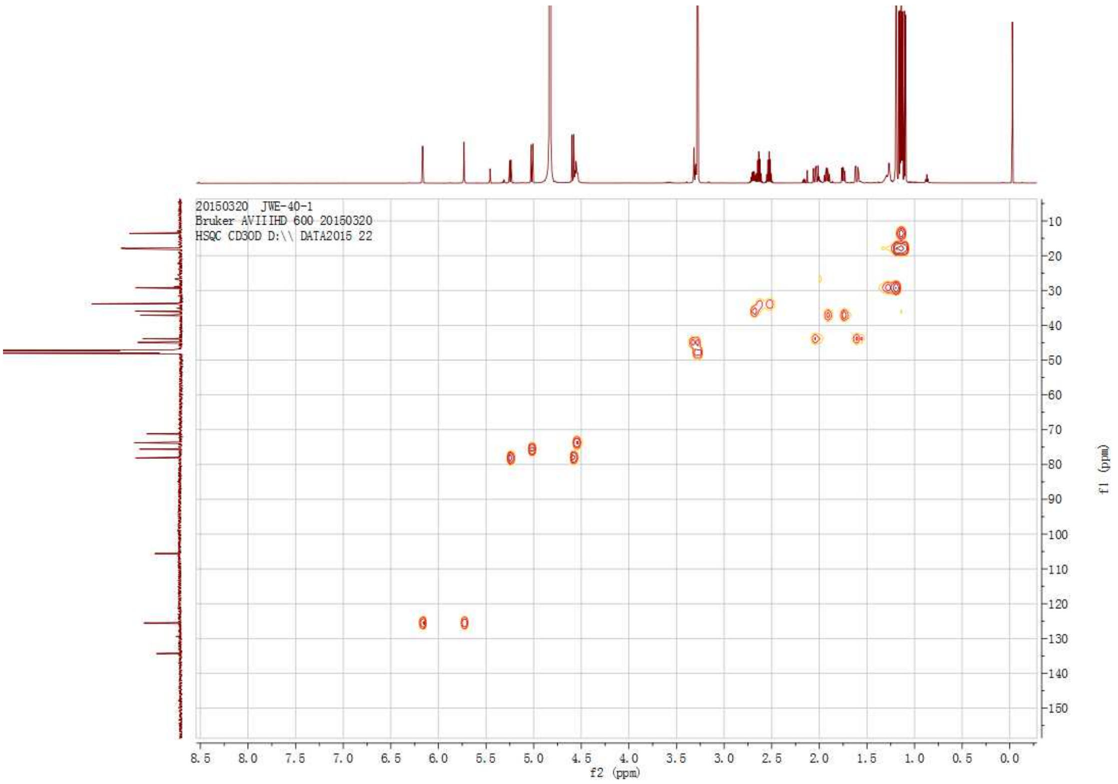

Fig. S8.4 HSQC spectrum (600 MHz) of (2*S*, 5*R*)-isocardivarolide A (**8**) in CD_3OD

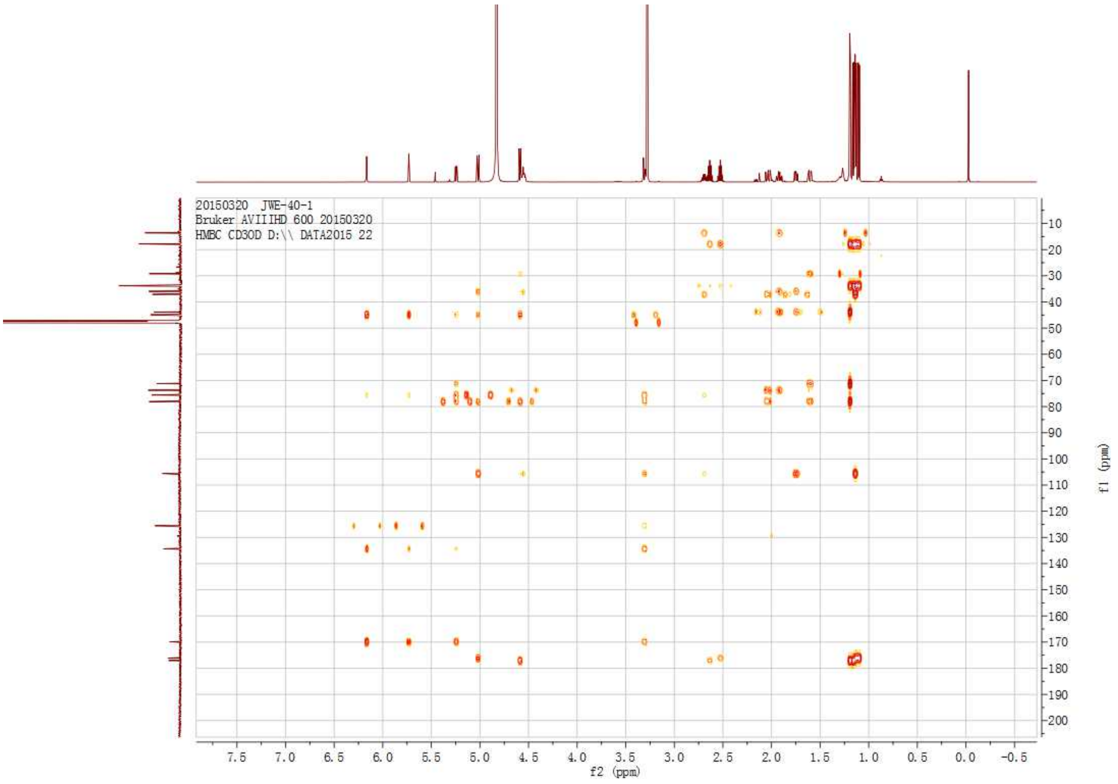

Fig. S8.5 HMBC spectrum (600 MHz) of (2*S*, 5*R*)-isocardivarolide A (**8**) in CD_3OD

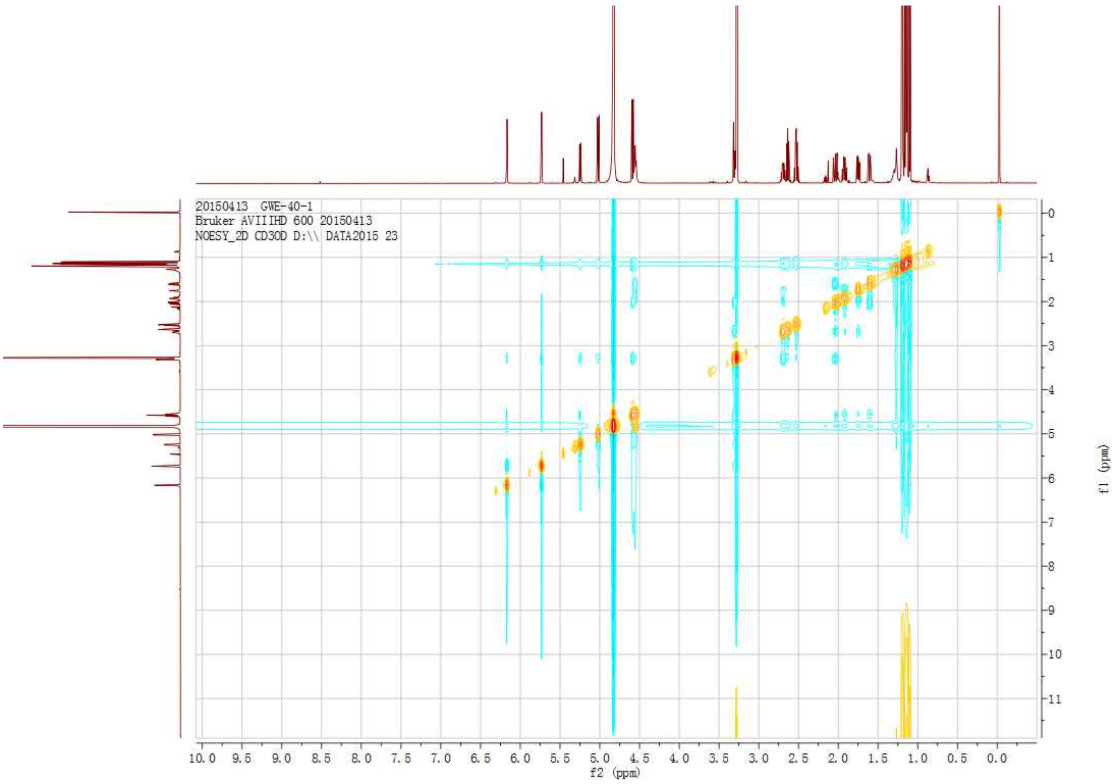

Fig. S8.6 NOESY spectrum (600 MHz) of (2*S*, 5*R*)-isocardivarolide A (**8**) in CD_3OD

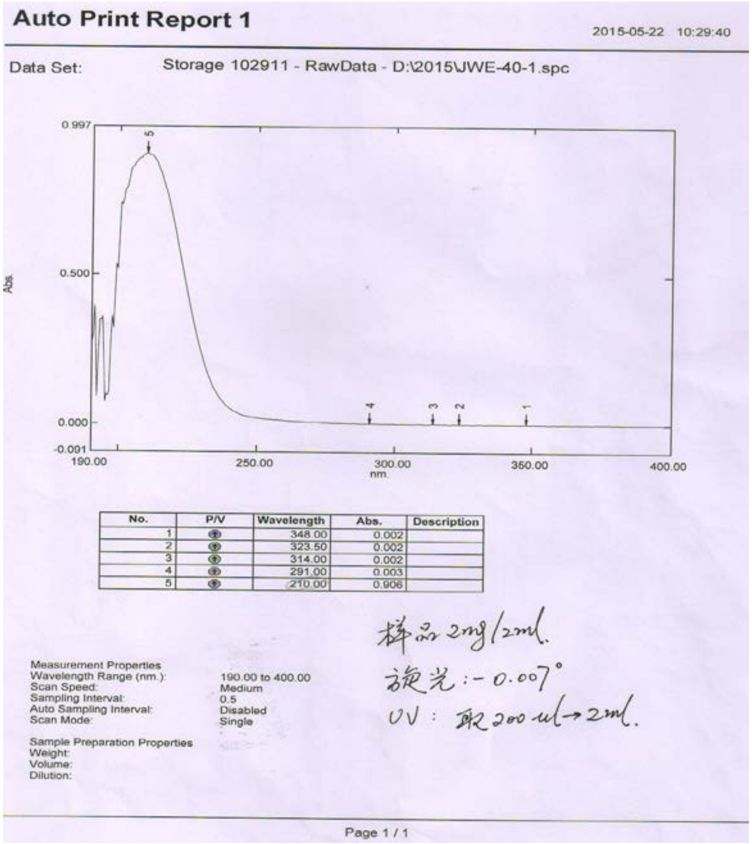

No.	P/V	Wavelength	Abs.	Description
1		348.00	0.002	
2		323.50	0.002	
3		314.00	0.002	
4		291.00	0.003	
5		210.00	0.906	

Fig. S8.7 UV spectrum of (2*S*, 5*R*)-isocardivarolide A (**8**)

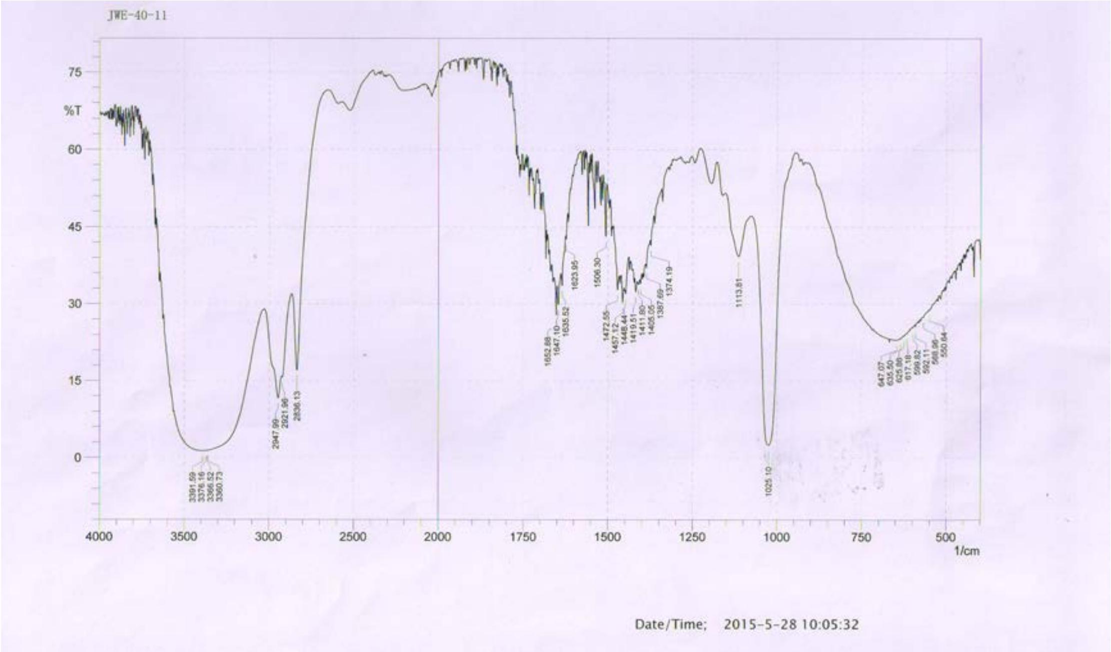

Fig. S8.8 IR spectrum of (2*S*, 5*R*)-isocardivarolide A (**8**)

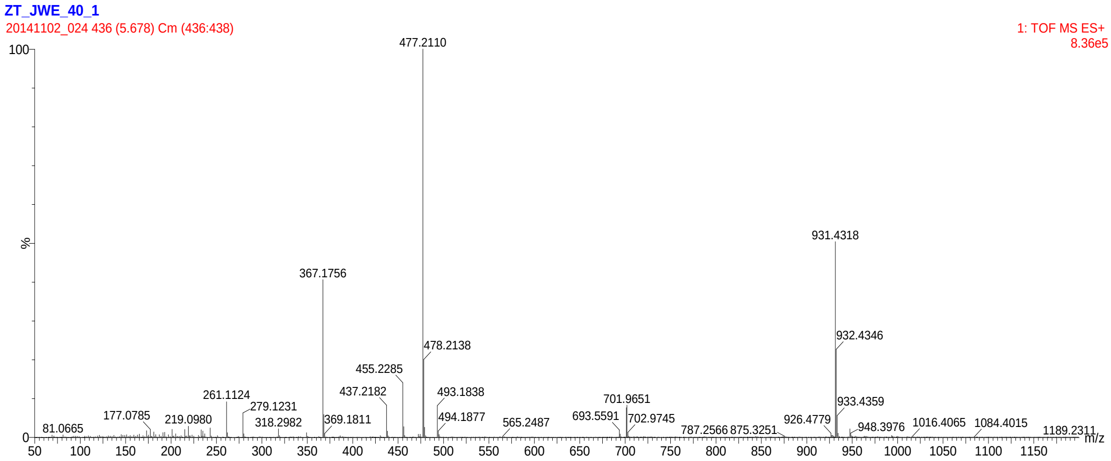

Fig. S8.9 HRESIMS spectrum of (2*S*, 5*R*)-isocardivarolide A (**8**)

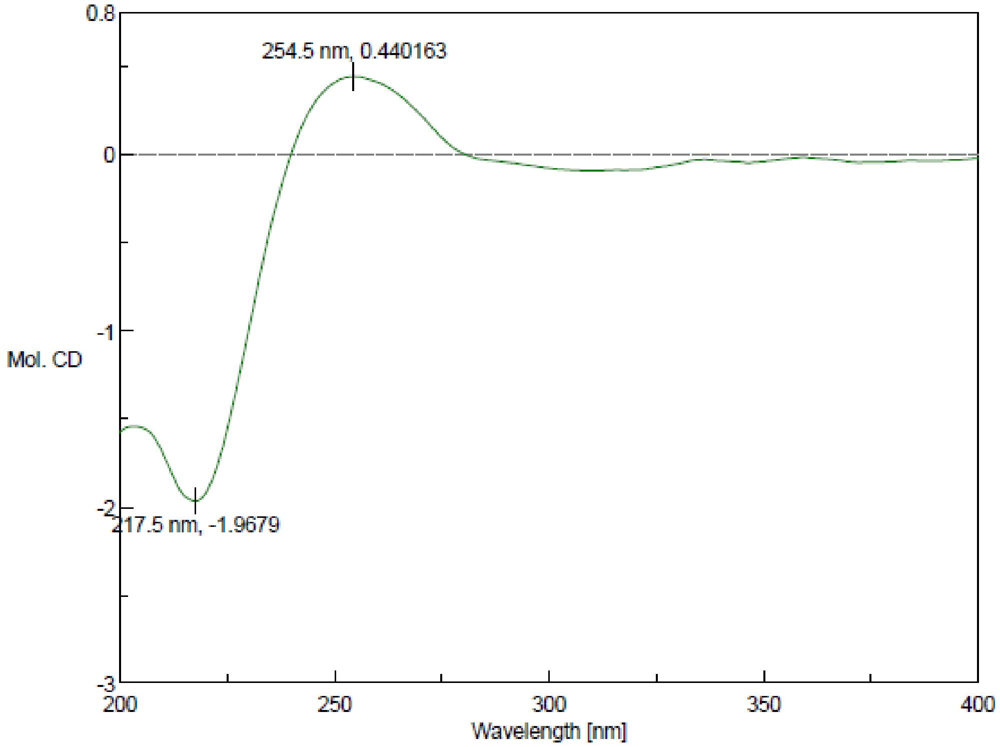

Fig. S8.10 HRESIMS spectrum of (2*S*, 5*R*)-isocardivarolide A (**8**)

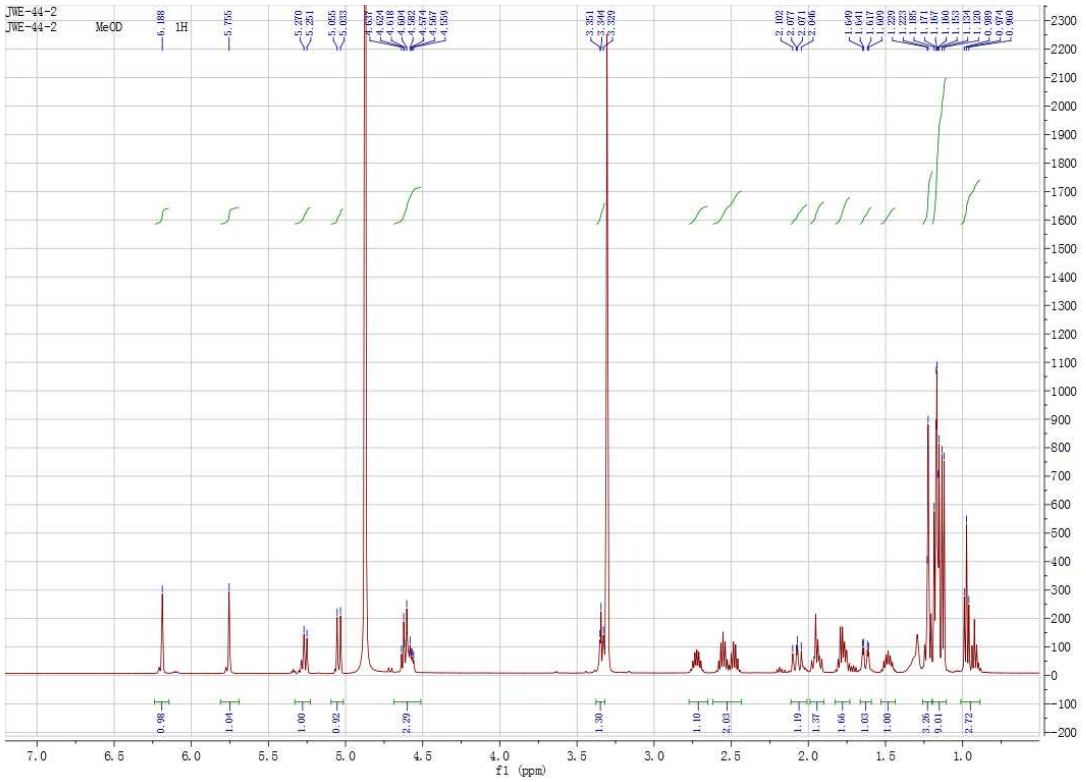

Fig. S9.1 ^{1}H NMR spectrum (500 MHz) of (2*S*, 5*R*)-isocardivarolide F (**9**) in CD_3OD

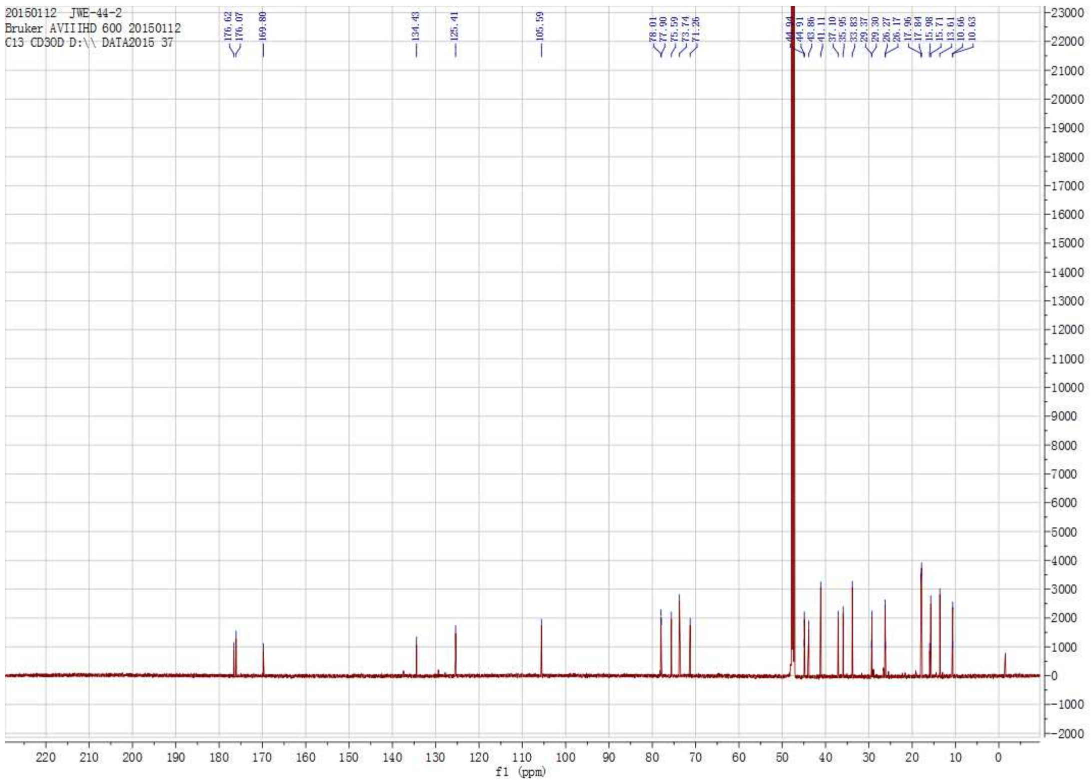

Fig. S9.2 ^{13}C NMR spectrum (125 MHz) of (2*S*, 5*R*)-isocardivarolide F (**9**) in CD_3OD

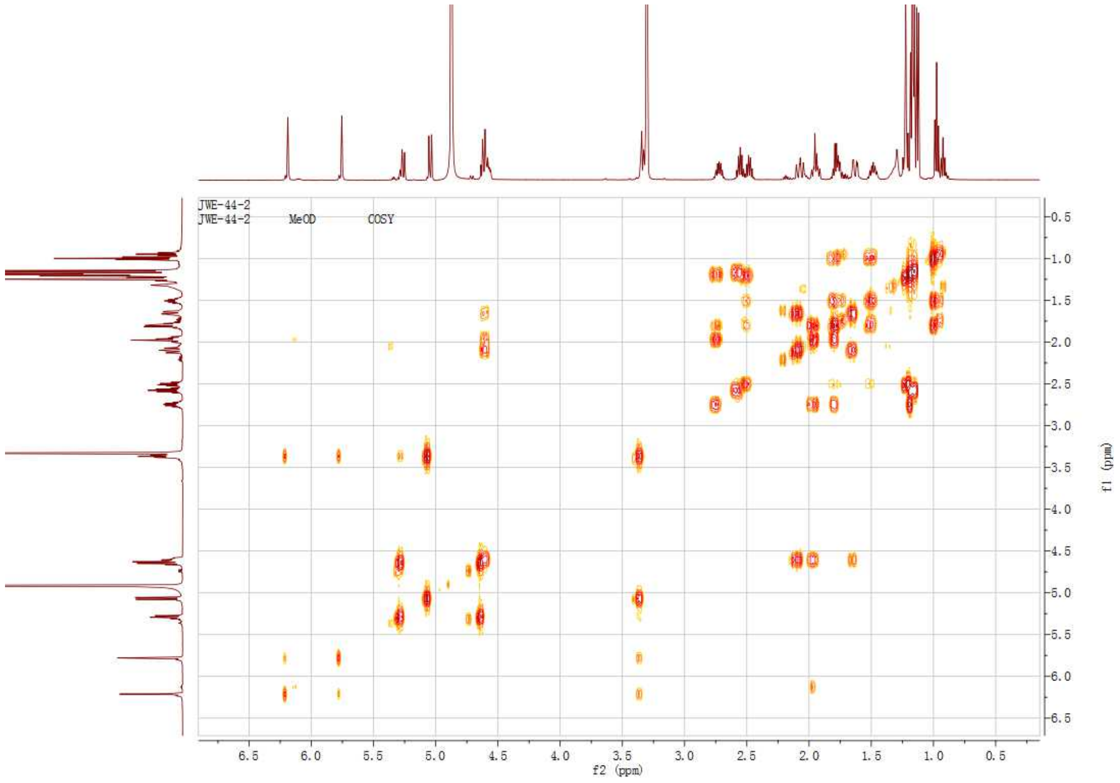

Fig. S9.3 ^{1}H-^{1}H COSY spectrum (500 MHz) of (2*S*, 5*R*)-isocardivarolide F (**9**) in CD_3OD

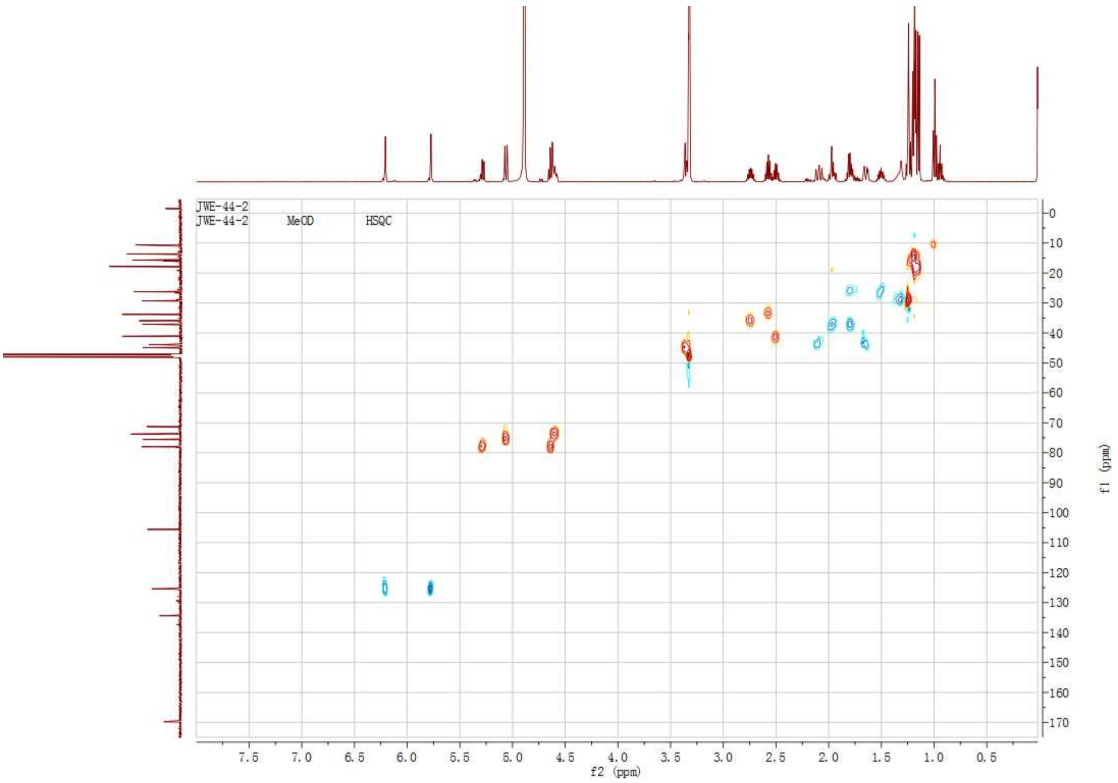

Fig. S9.4 HSQC spectrum (500 MHz) of (2*S*, 5*R*)-isocardivarolide F (**9**) in CD_3OD

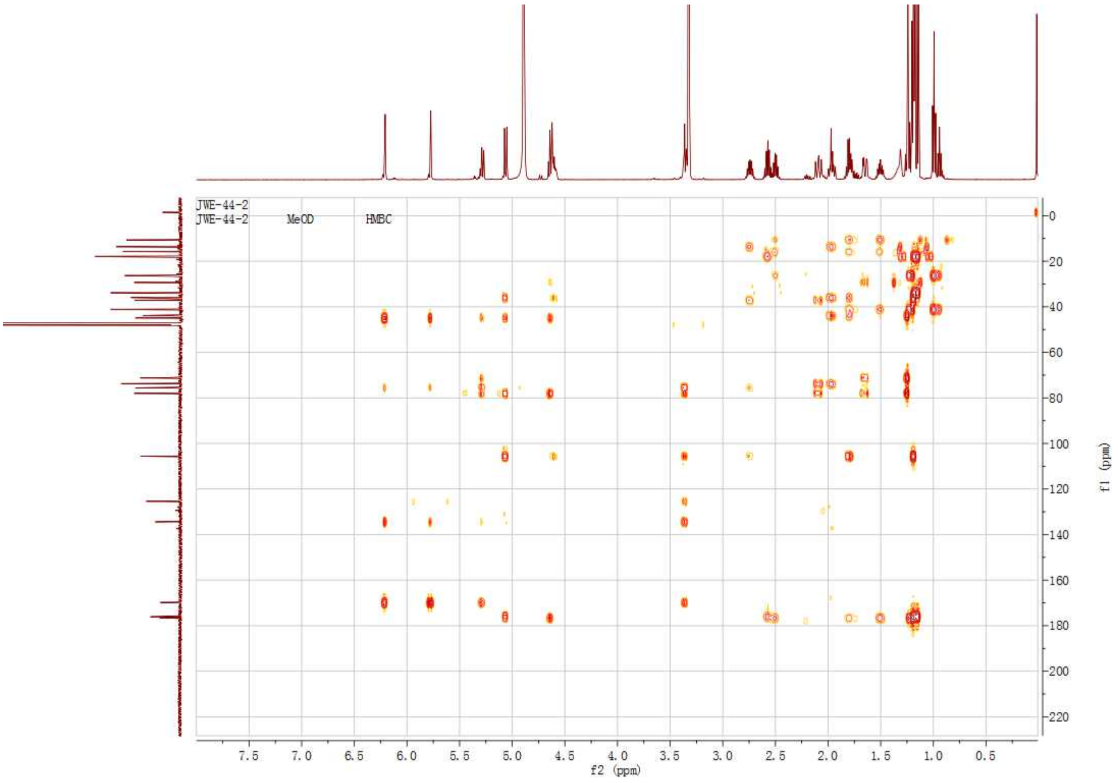

Fig. S9.5 HMBC spectrum (500 MHz) of (2*S*, 5*R*)-isocardivarolide F (**9**) in CD_3OD

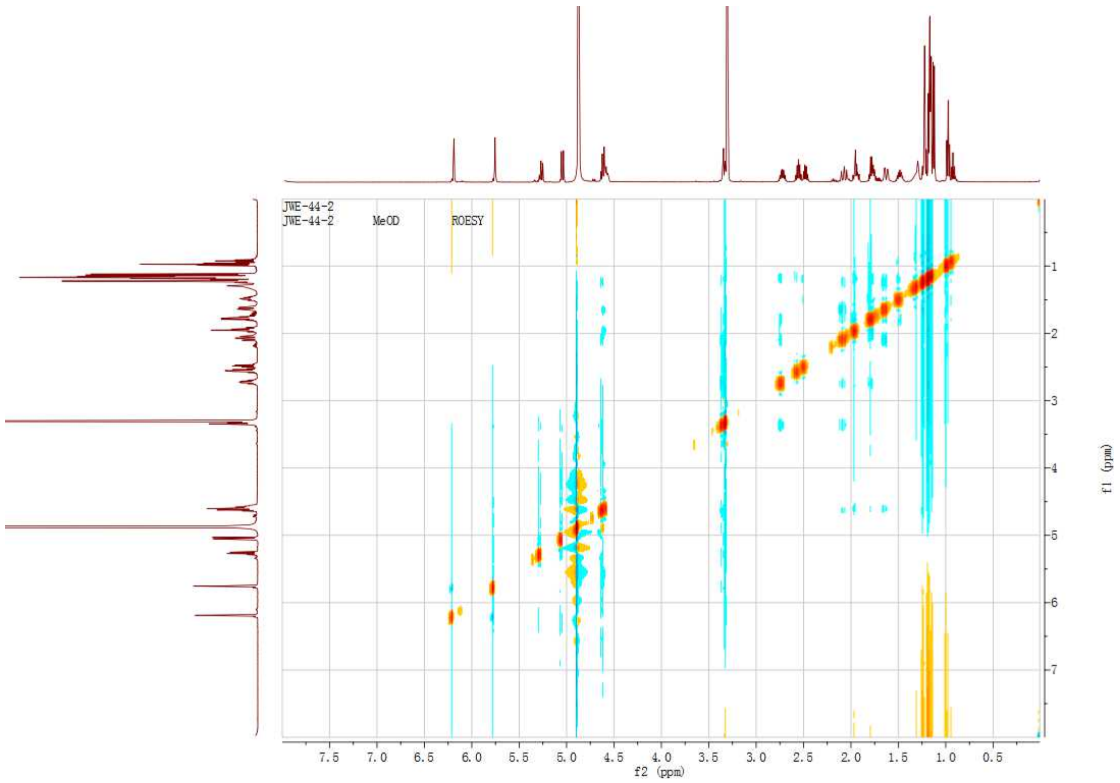

Fig. S9.6 ROESY spectrum (500 MHz) of (2*S*, 5*R*)-isocardivarolide F (**9**) in CD_3OD

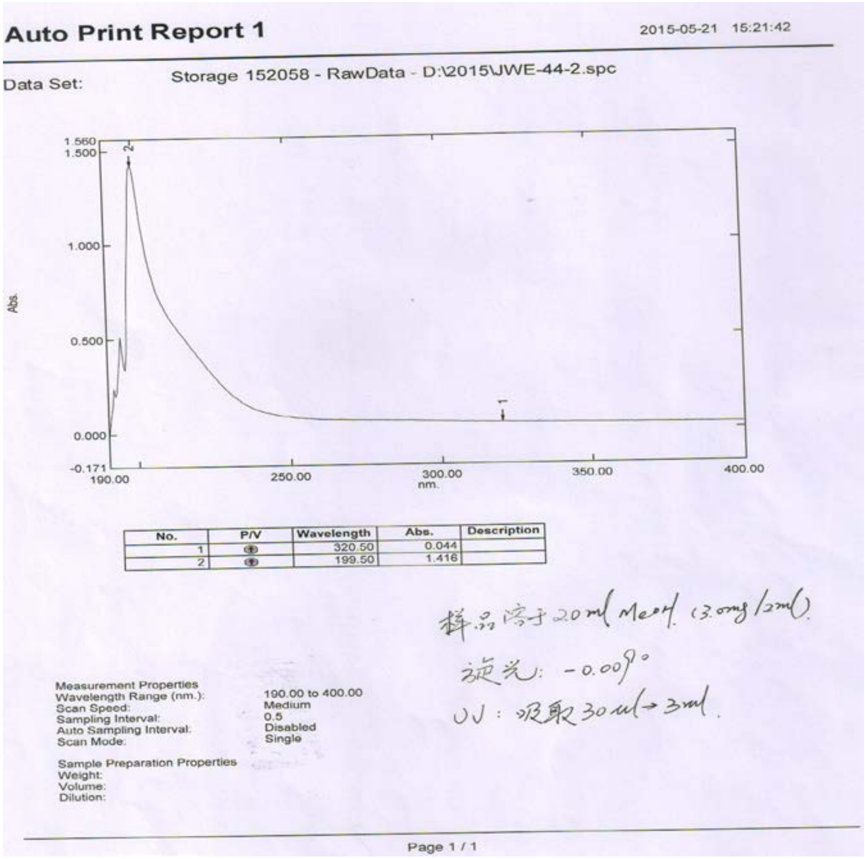
Auto Print Report 1 2015-05-21 15:21:42

Data Set: Storage 152058 - RawData - D:\2015\JWE-44-2.spc

No.	P/V	Wavelength	Abs.	Description
1	⊕	320.50	0.044	
2	⊕	199.50	1.416	

样品溶于2.0ml MeOH (3.0mg/2ml)
旋光: -0.009°
UV: 吸取30ul→3ml

Measurement Properties
Wavelength Range (nm.): 190.00 to 400.00
Scan Speed: Medium
Sampling Interval: 0.5
Auto Sampling Interval: Disabled
Scan Mode: Single

Sample Preparation Properties
Weight:
Volume:
Dilution:

Page 1 / 1

Fig. S9.7 UV spectrum of (2*S*, 5*R*)-isocardivarolide F (**9**)

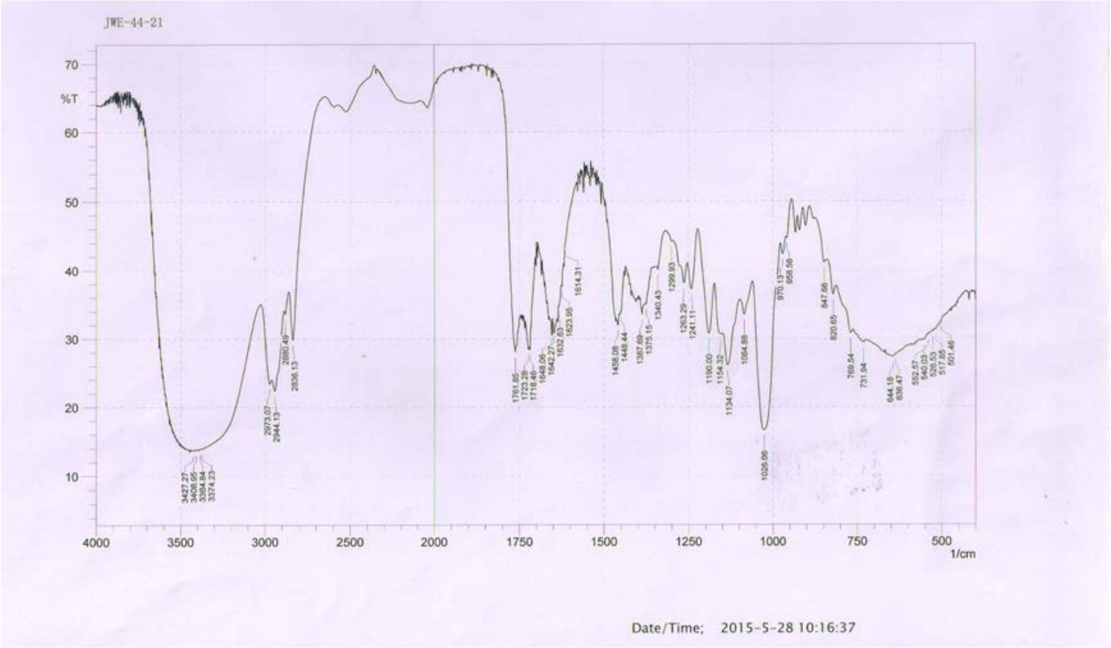

Fig. S9.8 IR spectrum of (2*S*, 5*R*)-isocardivarolide F (**9**)

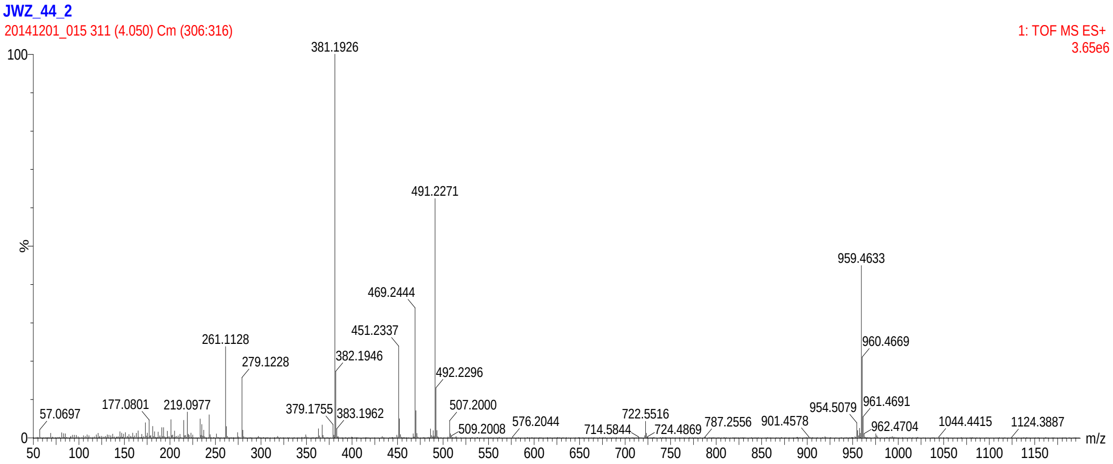

Fig. S9.9 HRESIMS spectrum of (2*S*, 5*R*)-isocardivarolide F (**9**)

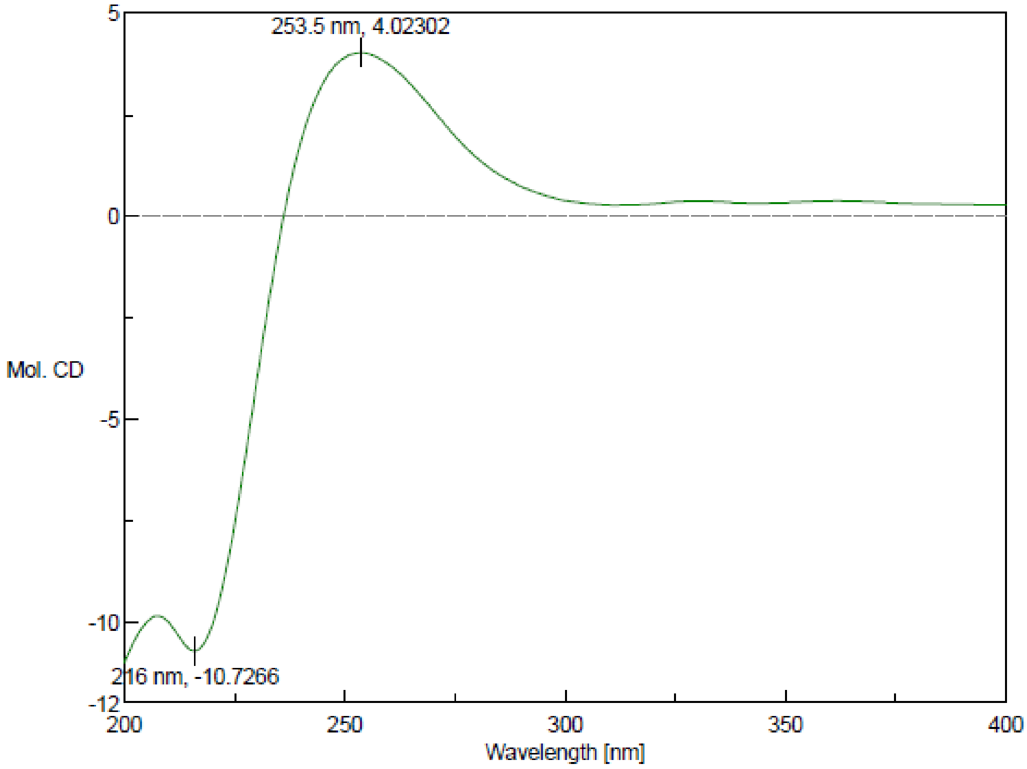

Fig. S9.9 CD spectrum of (2*S*, 5*R*)-isocardivarolide F (**9**)

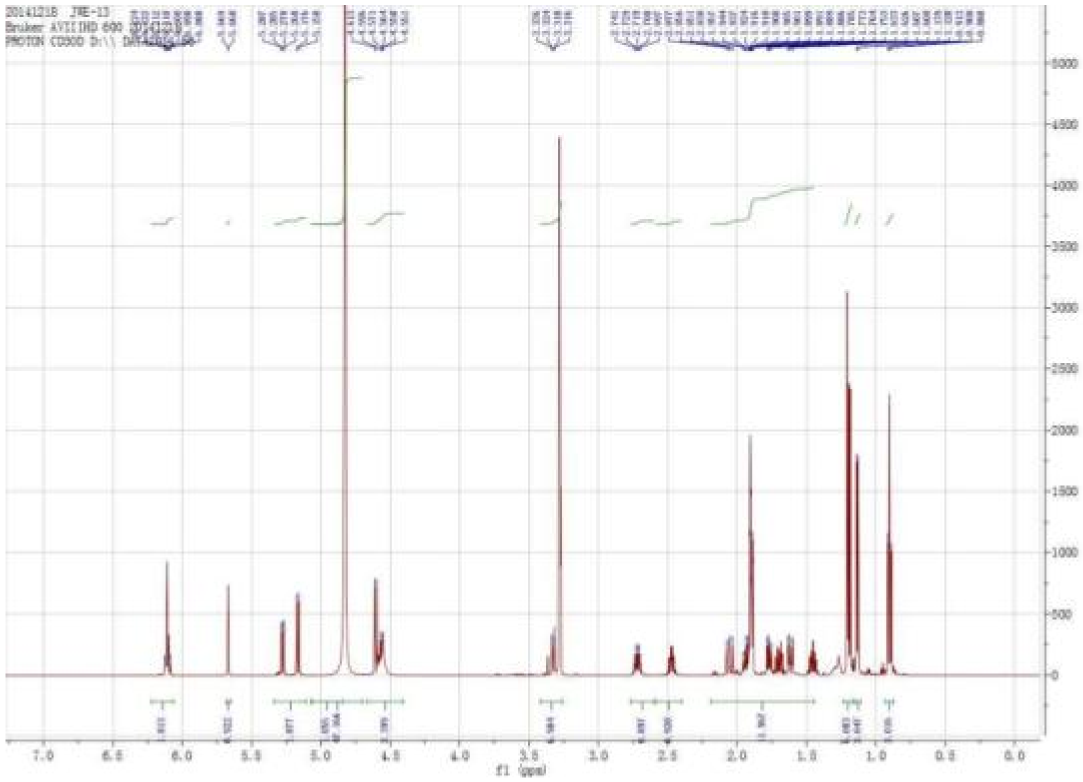

Fig. S10.1 ^{1}H NMR spectrum (600 MHz) of (2*S*, 5*R*, 2″*R*)-ineupatolide (**10**) in CD_3OD

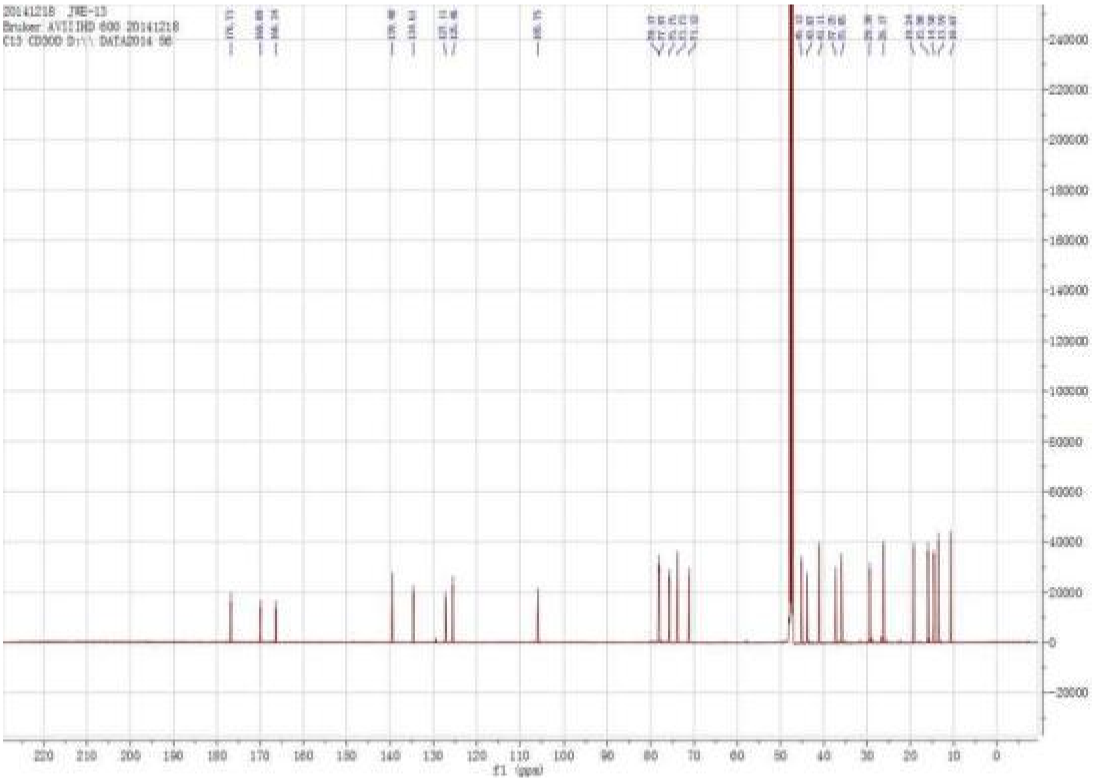

Fig. S10.2 ^{13}C NMR spectrum (150 MHz) of (2*S*, 5*R*, 2″*R*)-ineupatolide (**10**) in CD_3OD

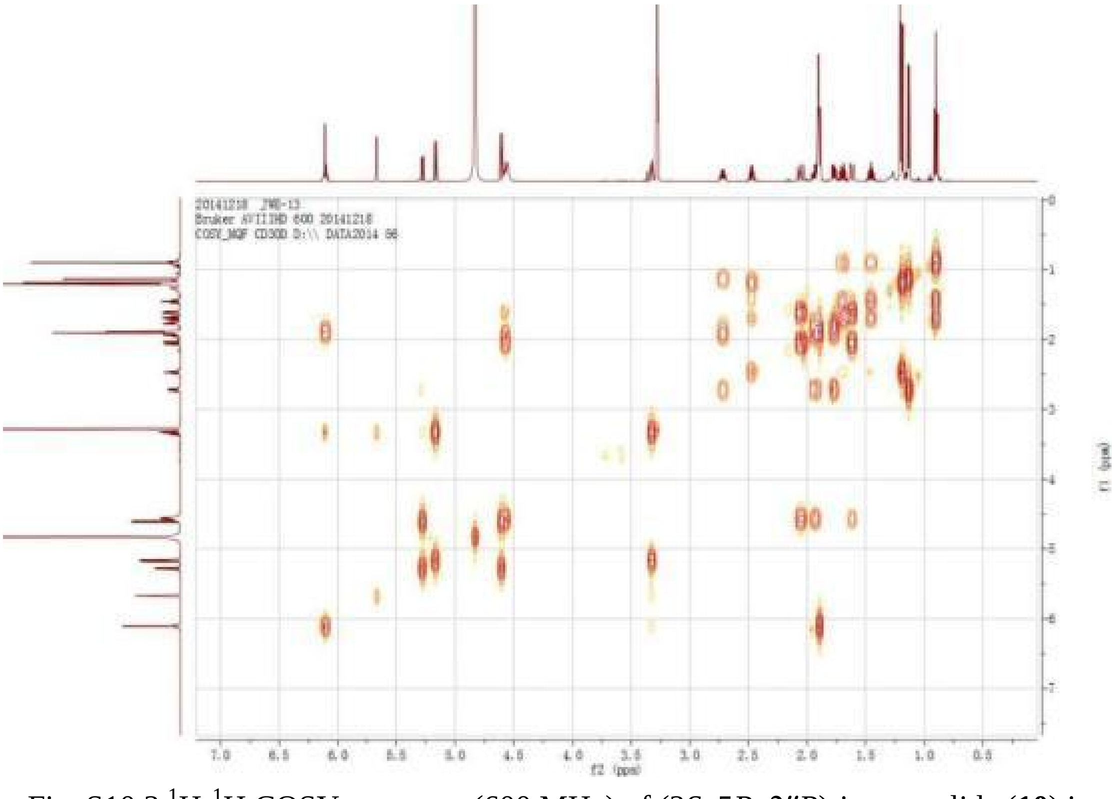

Fig. S10.3 ^{1}H-^{1}H COSY spectrum (600 MHz) of (2*S*, 5*R*, 2″*R*)-ineupatolide (**10**) in CD_3OD

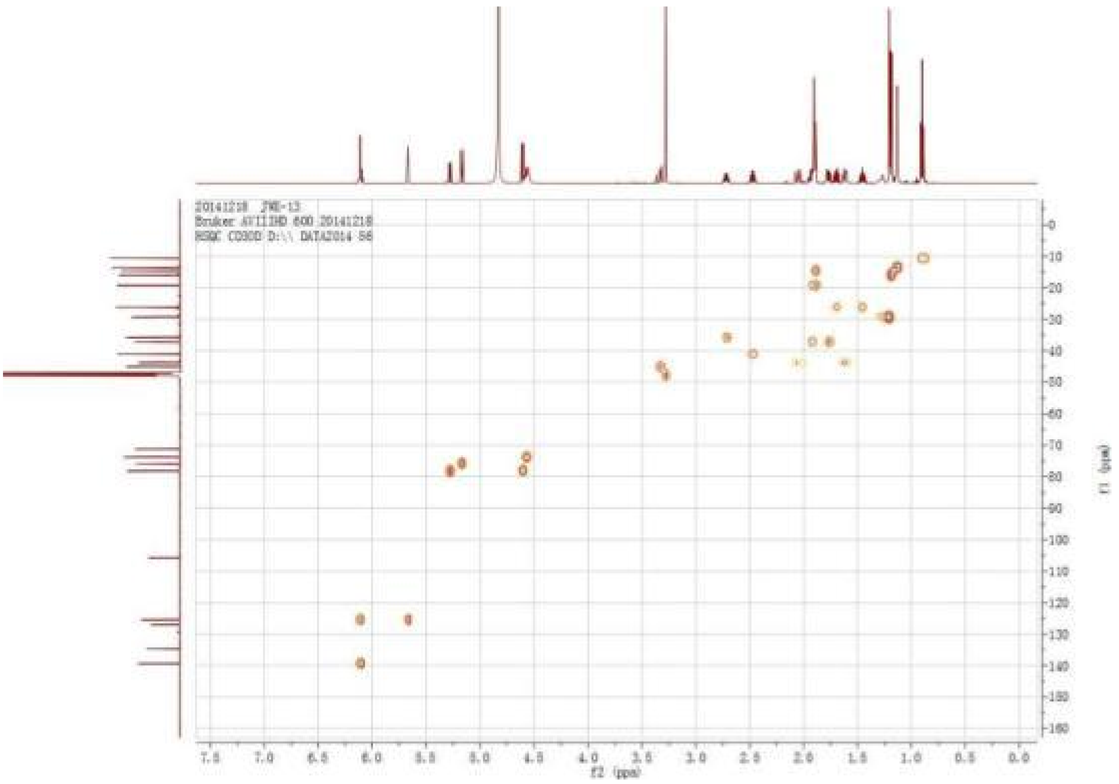

Fig. S10.4 HSQC spectrum (600 MHz) of (2*S*, 5*R*, 2″*R*)-ineupatolide (**10**) in CD_3OD

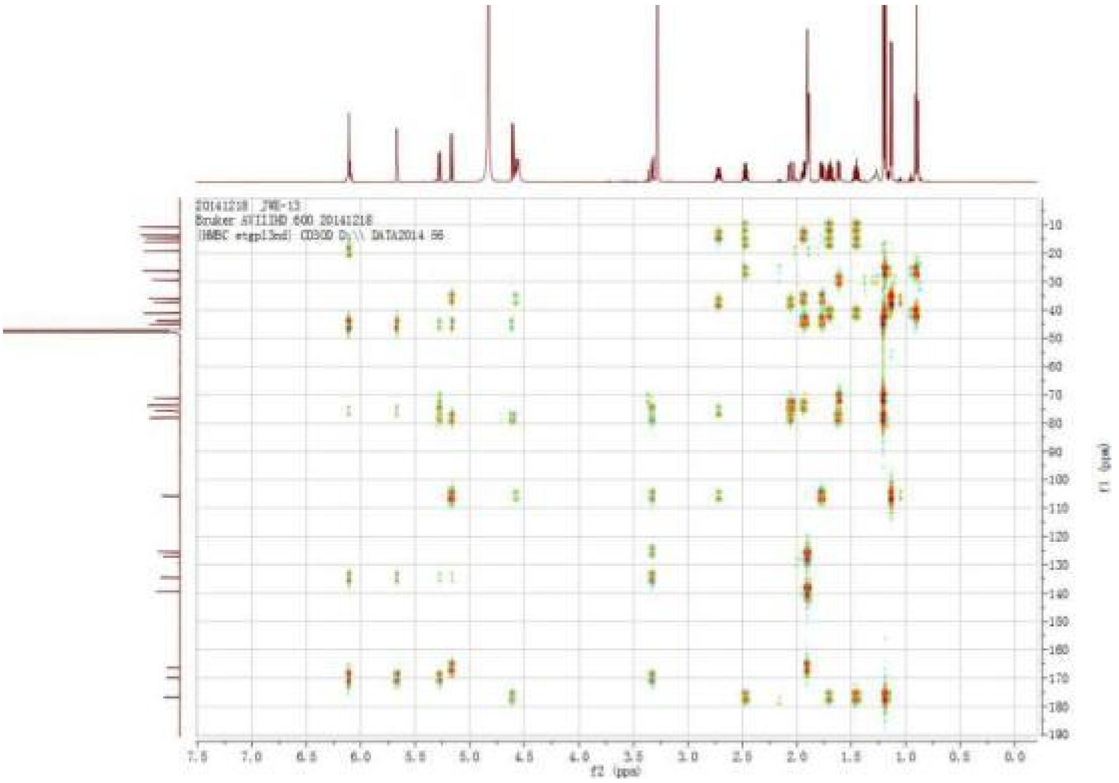

Fig. S10.5 HMBC spectrum (600 MHz) of (2*S*, 5*R*, 2″*R*)-ineupatolide (**10**) in CD_3OD

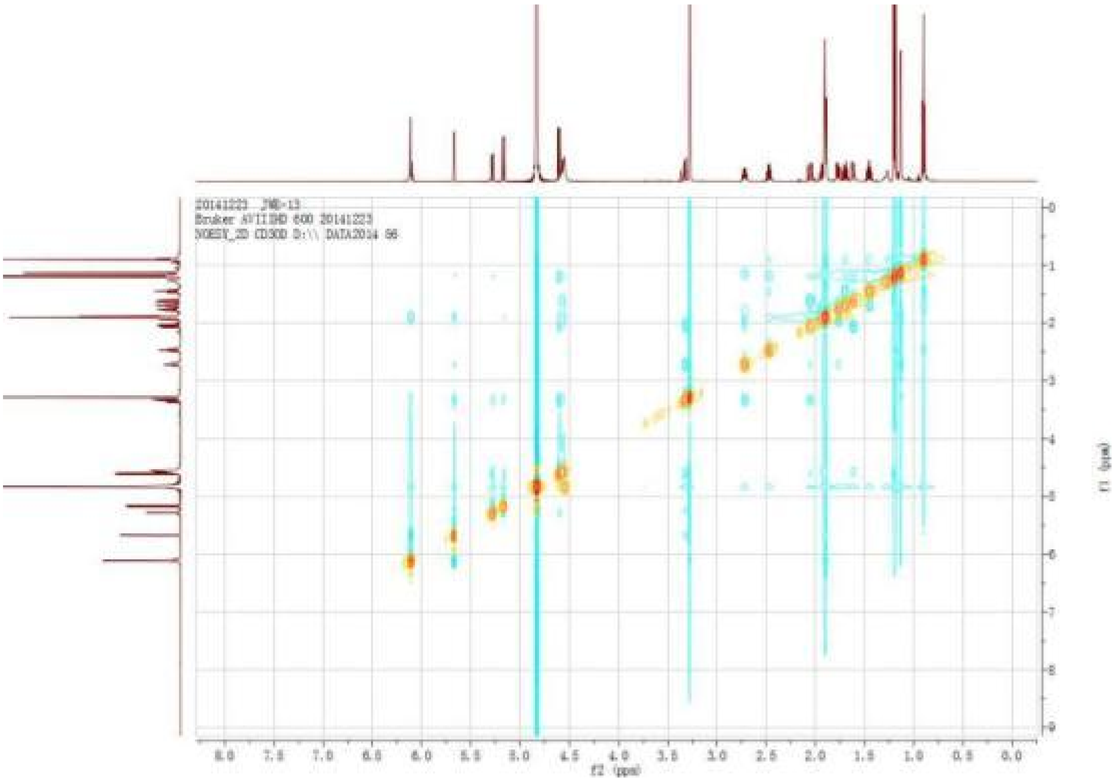

Fig. S10.6 NOESY spectrum (600 MHz) of (2*S*, 5*R*, 2″*R*)-ineupatolide (**10**) in CD_3OD

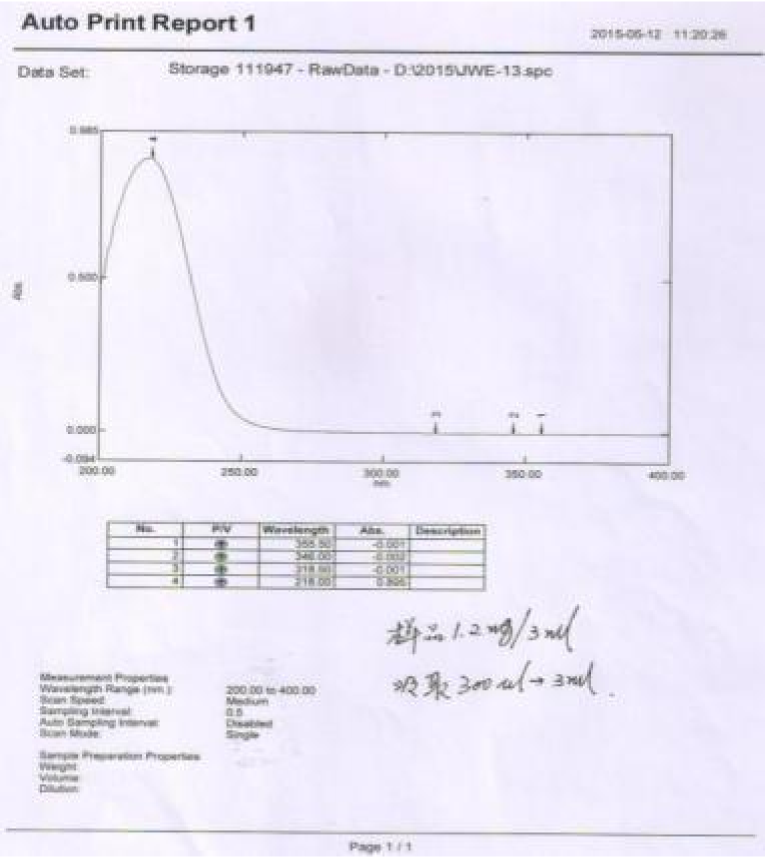

Fig. S10.7 UV spectrum of (2*S*, 5*R*, 2″*R*)-ineupatolide (**10**)

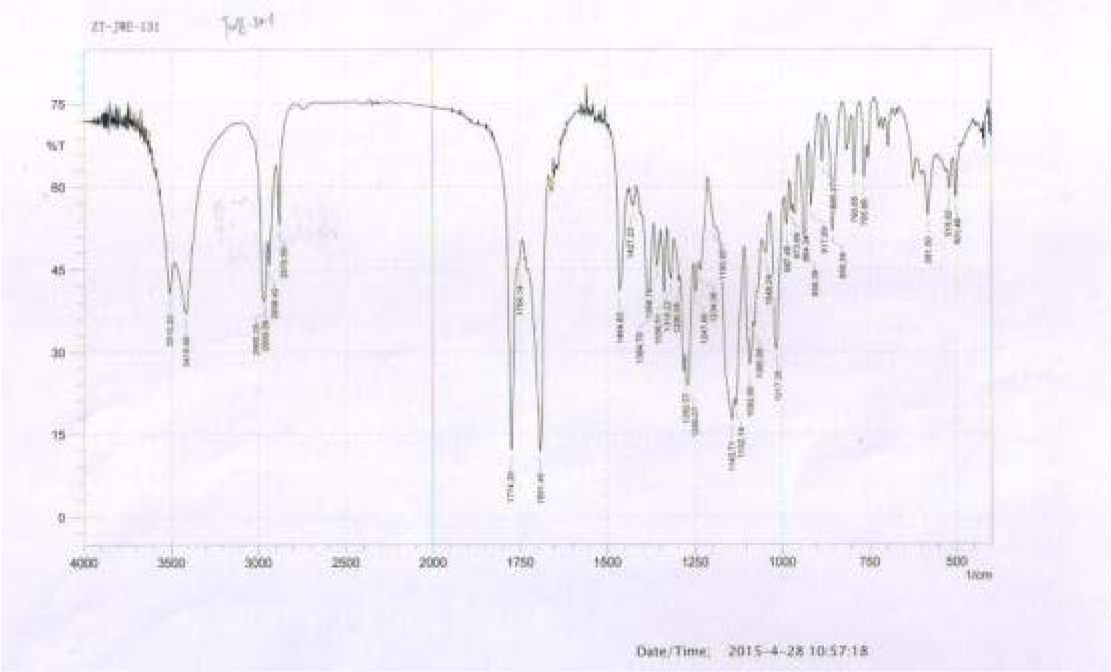

Fig. S10.8 IR spectrum of (2*S*, 5*R*, 2″*R*)-ineupatolide (**10**)

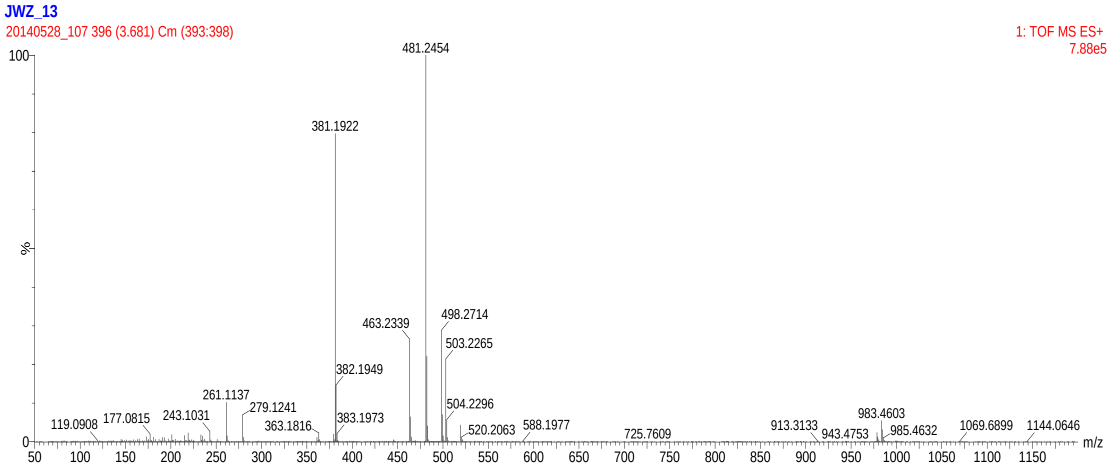

Fig. S10.9 HRESIMS spectrum of (2*S*, 5*R*, 2″*R*)-ineupatolide (**10**)

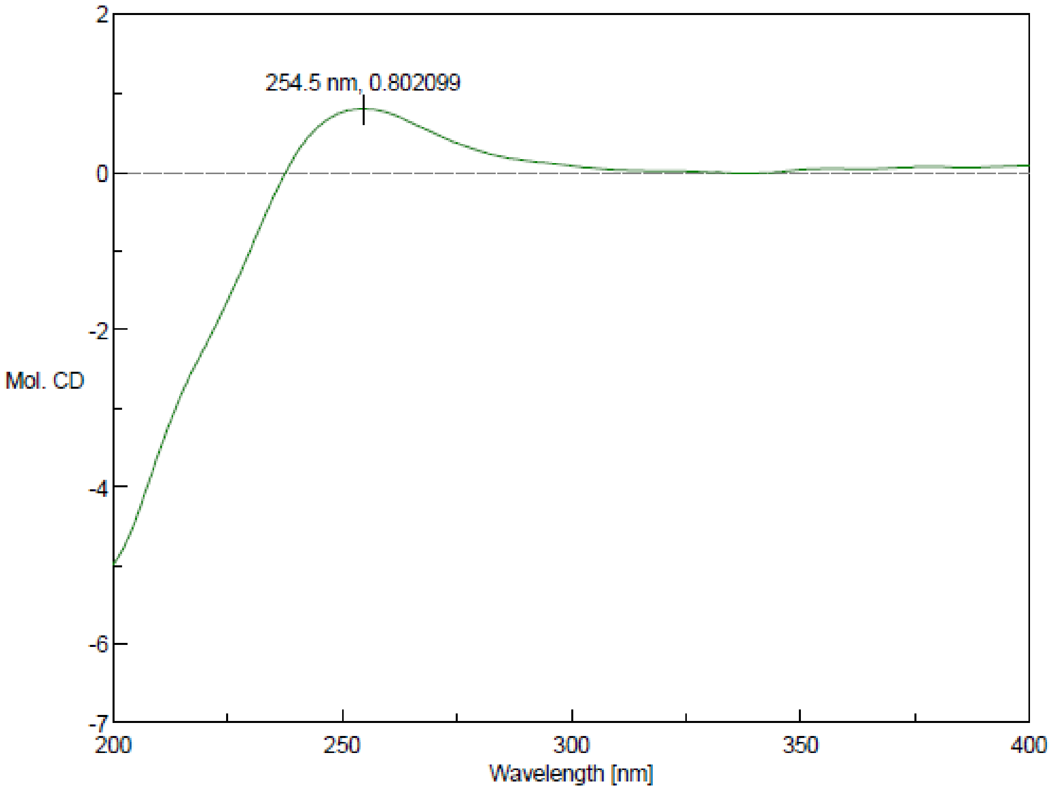

Fig. S10.10 HRESIMS spectrum of (2*S*, 5*R*, 2″*R*)-ineupatolide (**10**)

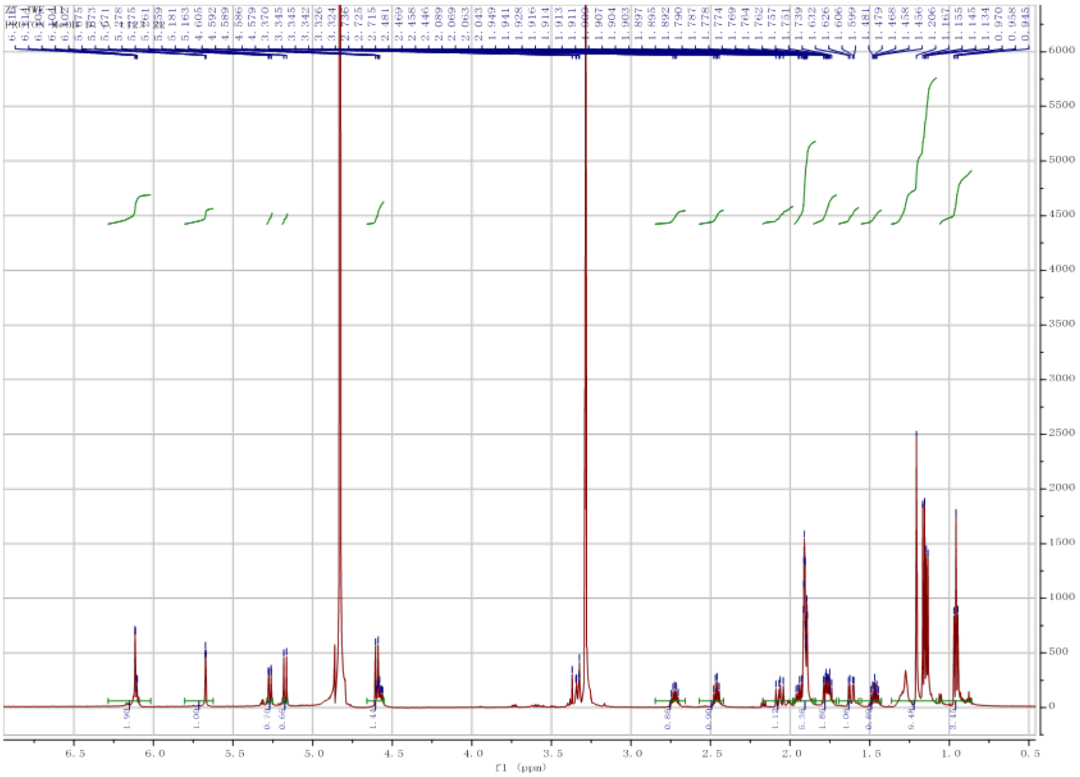

Fig. S11.1 ^{1}H NMR spectrum (600 MHz) of (2*S*, 5*R*, 2″*S*)-ineupatolide (**11**) in CD_3OD

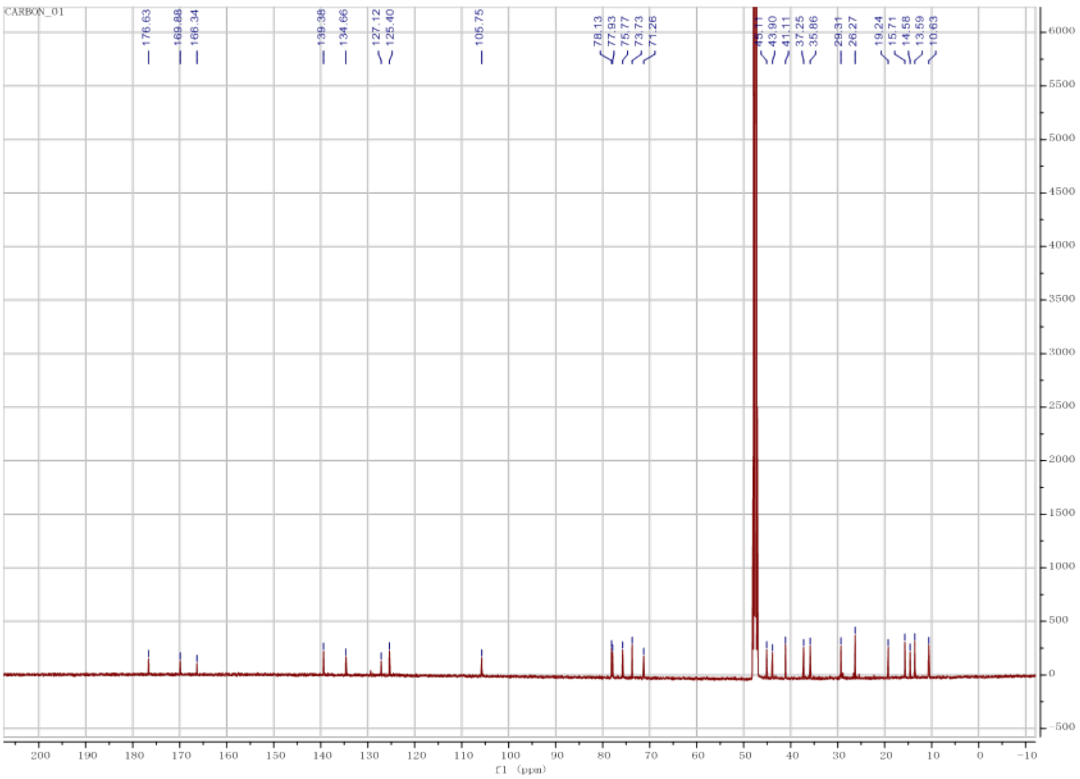

Fig. S11.2 ^{13}C NMR spectrum (150 MHz) of (2*S*, 5*R*, 2″*S*)-ineupatolide (**11**) in CD_3OD

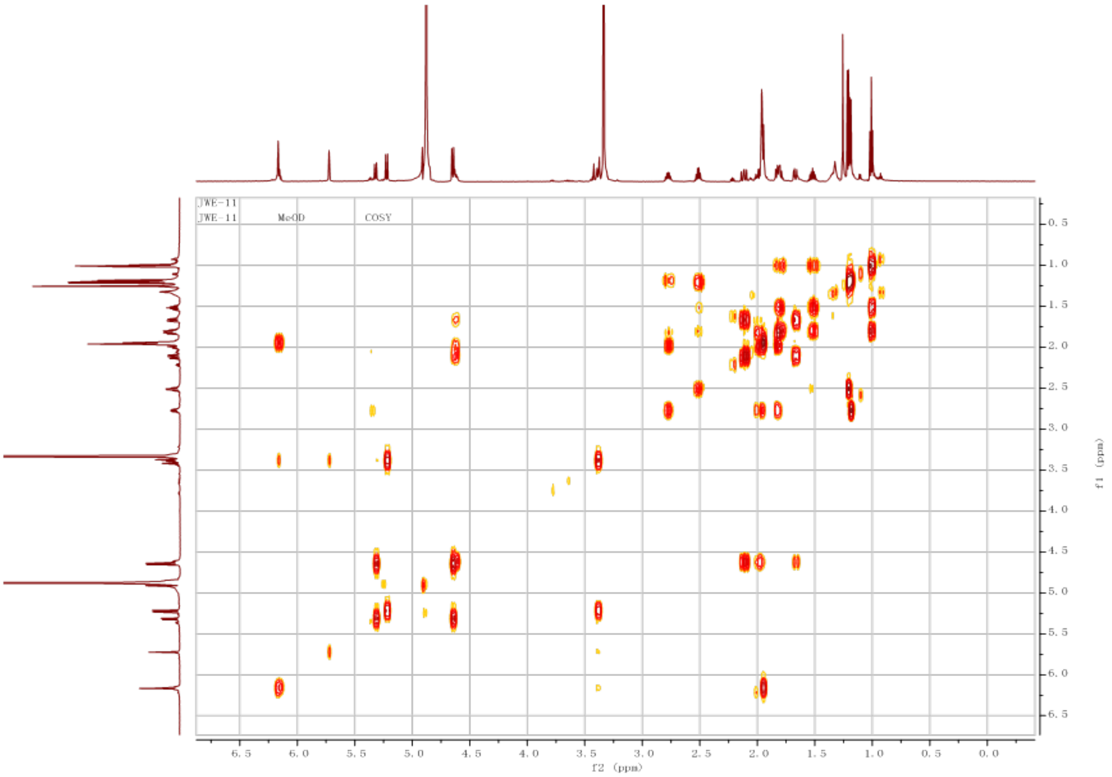

Fig. S11.3 ^{1}H-^{1}H COSY spectrum (600 MHz) of (2*S*, 5*R*, 2″*S*)-ineupatolide (**11**) in CD_3OD

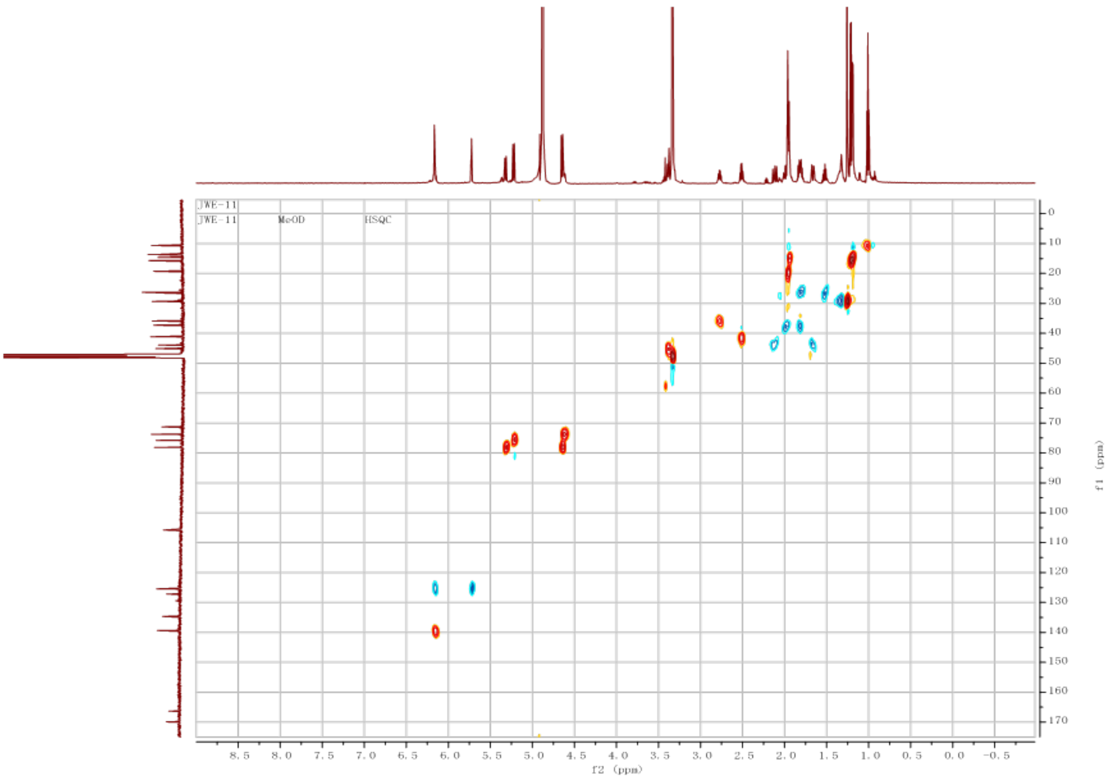

Fig. S11.4 HSQC spectrum (600 MHz) of (2*S*, 5*R*, 2″*S*)-ineupatolide (**11**) in CD_3OD

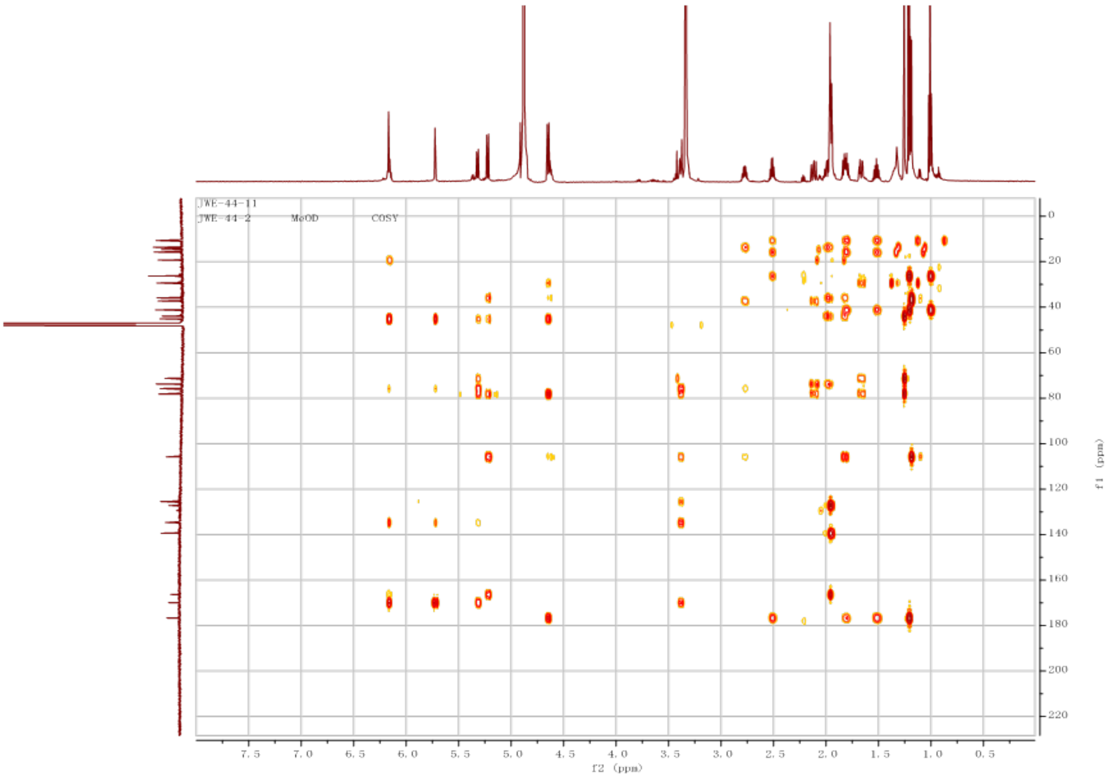

Fig. S11.5 HMBC spectrum (600 MHz) of (2*S*, 5*R*, 2″*S*)-ineupatolide (**11**) in CD_3OD

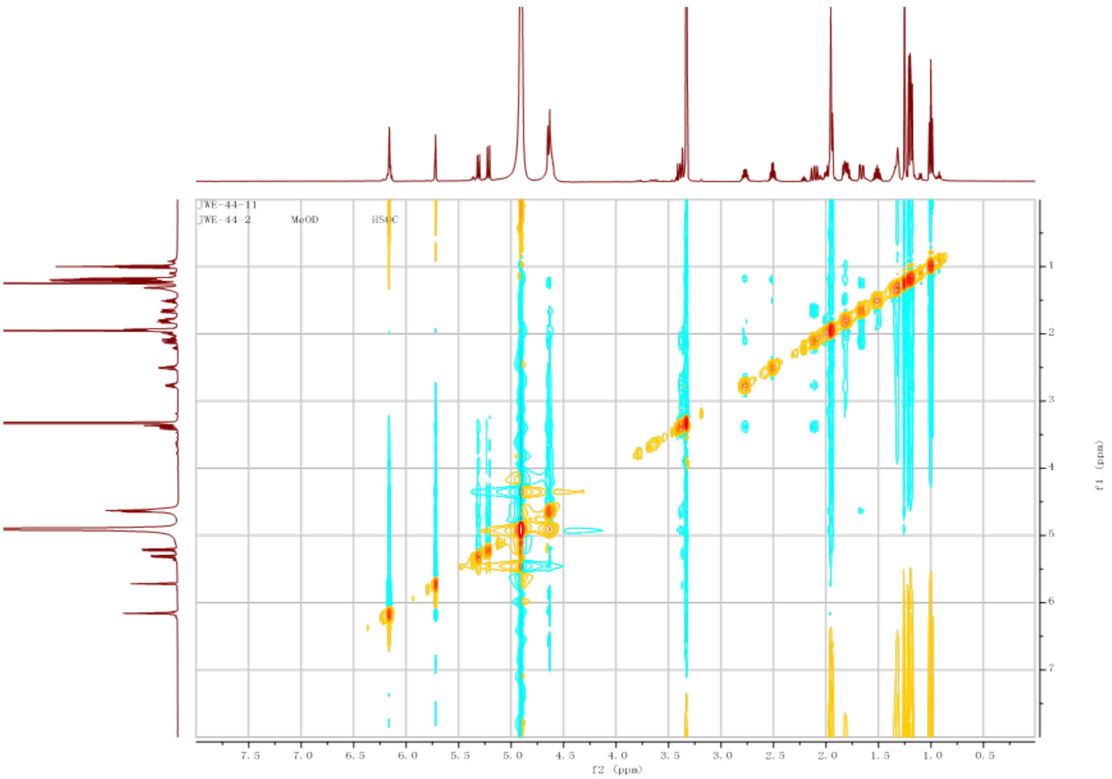

Fig. S11.6 NOESY spectrum (600 MHz) of (2*S*, 5*R*, 2″*S*)-ineupatolide (**11**) in CD_3OD

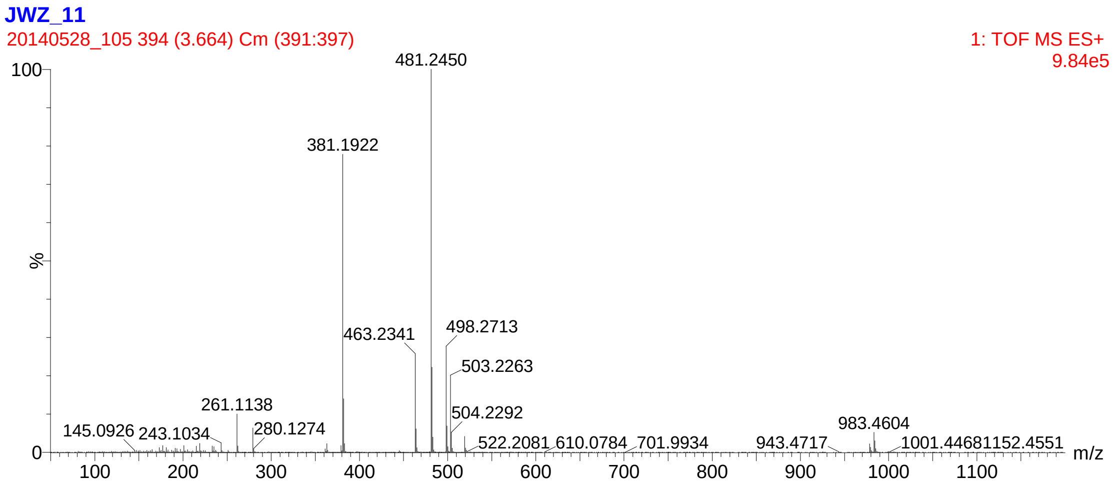

Fig. S11.7 HRESIMS spectrum of (2*S*, 5*R*, 2″*S*)-ineupatolide (**11**)

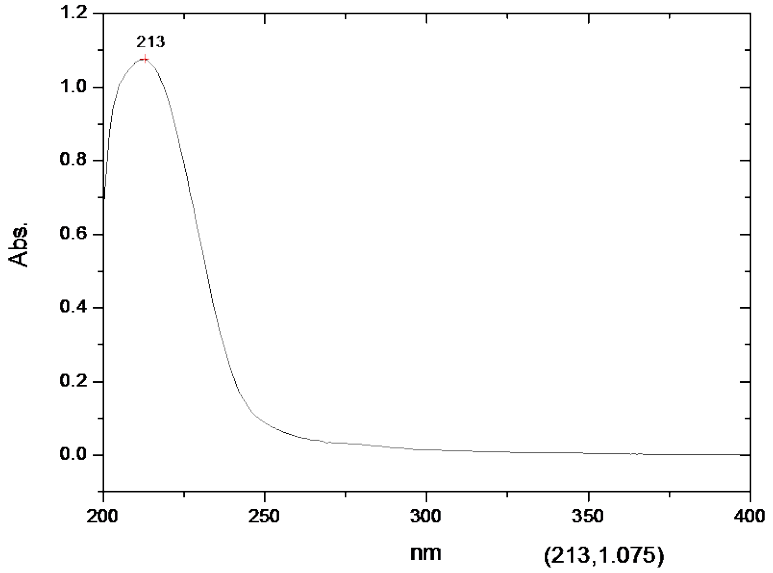

Fig. S11.8 UV spectrum of (2*S*, 5*R*, 2″*S*)-ineupatolide (**11**)

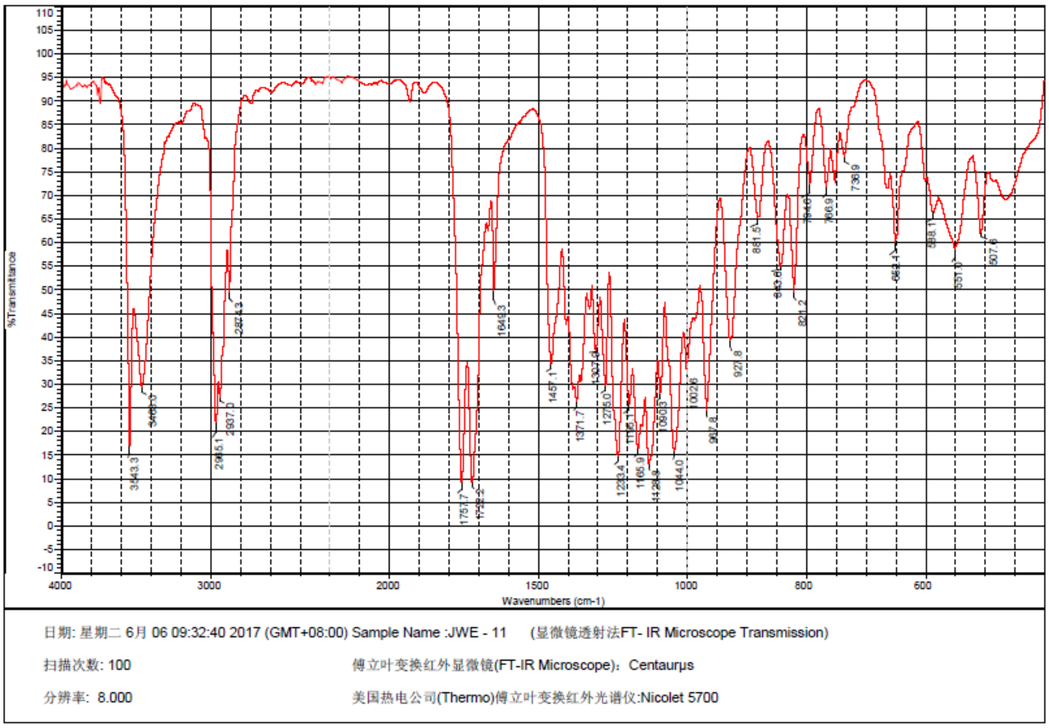

Fig. S11.9 IR spectrum of (2*S*, 5*R*, 2″*S*)-ineupatolide (**11**)

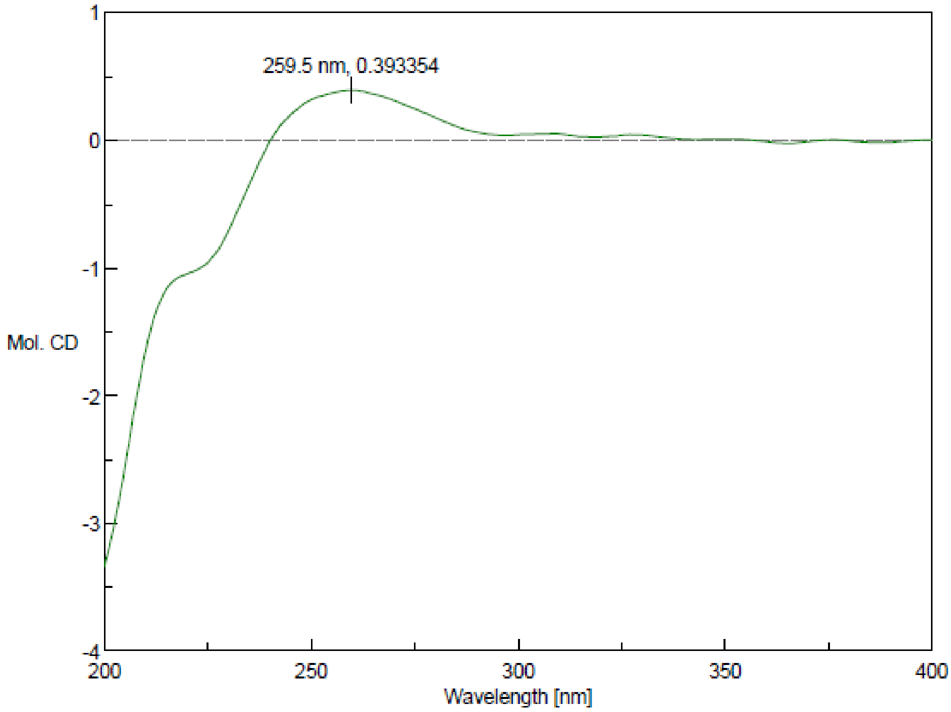

Fig. S11.10 CD spectrum of (2*S*, 5*R*, 2″*S*)-ineupatolide (**11**)

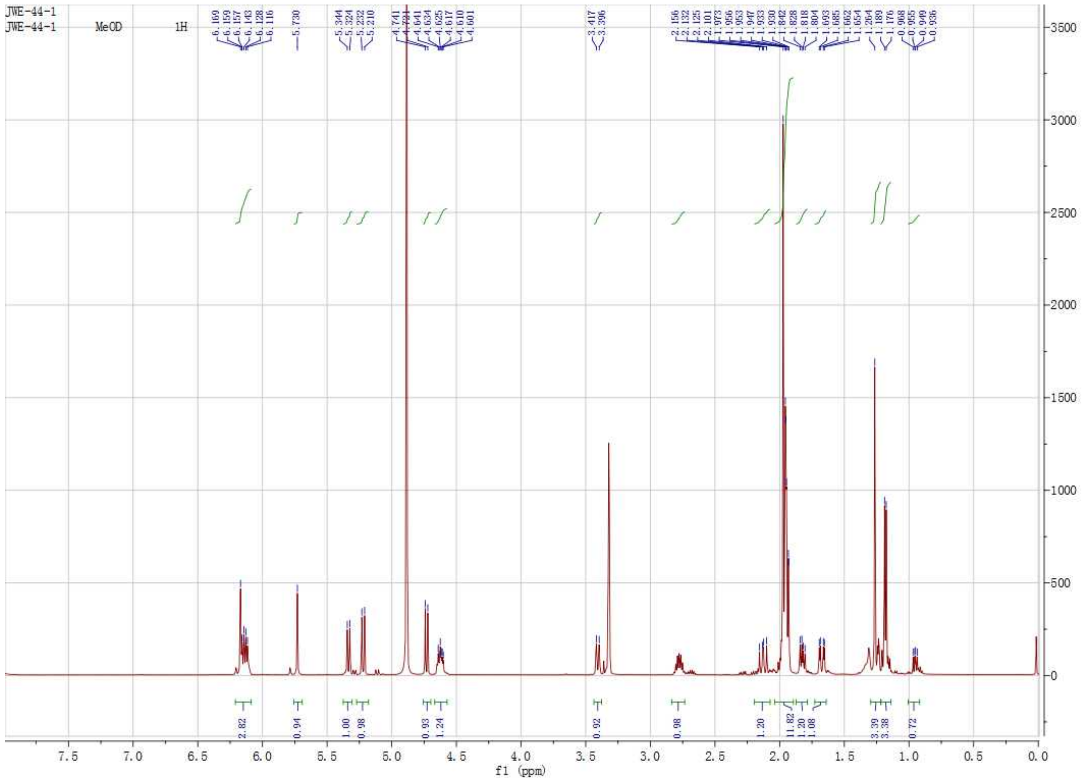

Fig. S12.1 ^{1}H NMR spectrum (500 MHz) of divaricin B (**12**) in CD_3OD

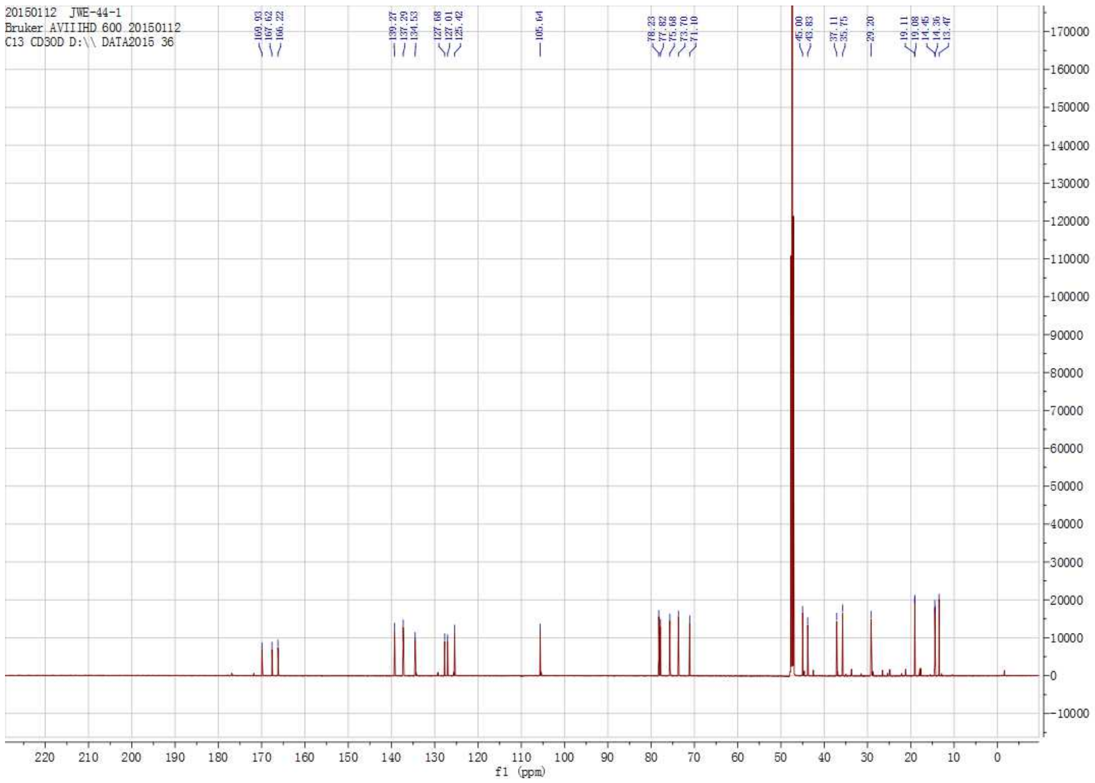

Fig. S12.2 ^{13}C NMR spectrum (125 MHz) of divaricin B (**12**) in CD_3OD

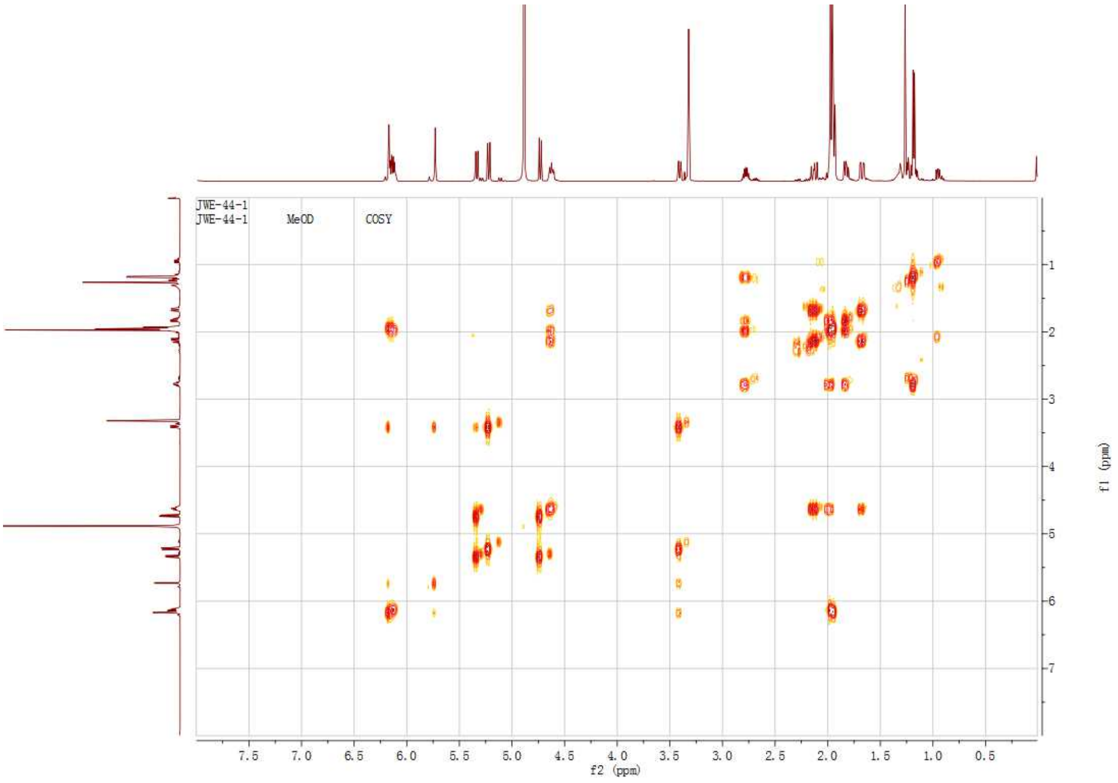

Fig. S12.3 ^{1}H-^{1}H COSY spectrum (500 MHz) of divaricin B (**12**) in CD_3OD

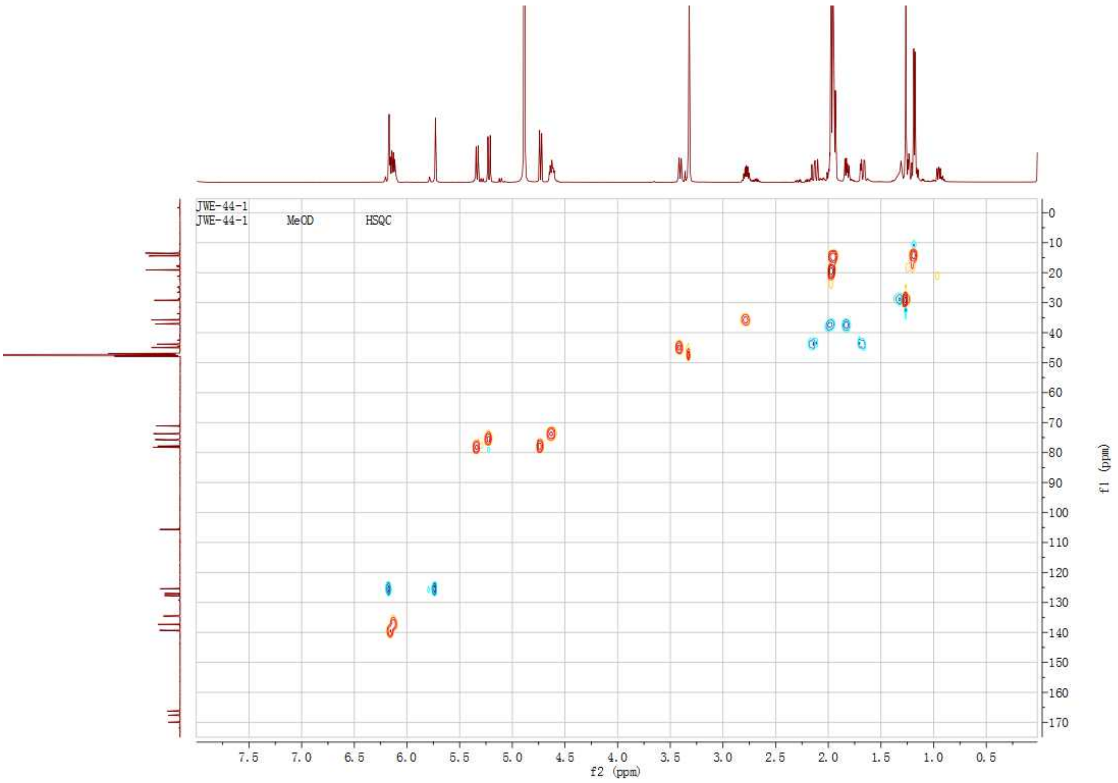

Fig. S12.4 HSQC spectrum (500 MHz) of divaricin B (**12**) in CD_3OD

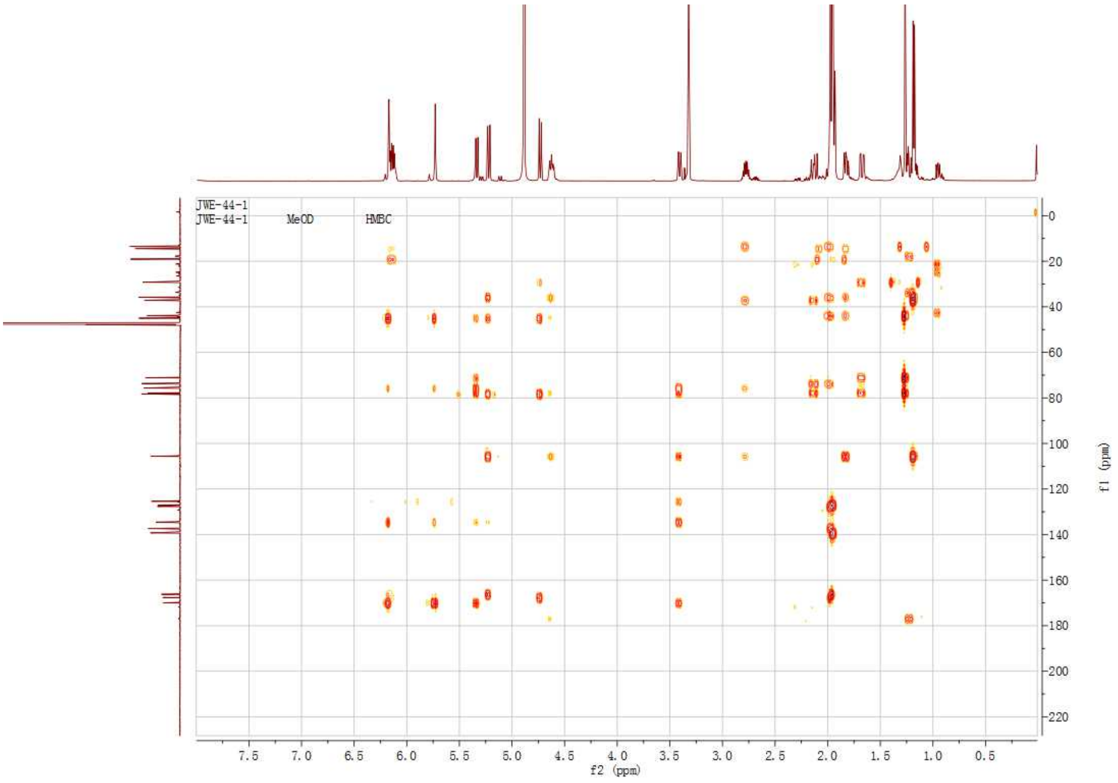

Fig. S12.5 HMBC spectrum (500 MHz) of divaricin B (**12**) in CD_3OD

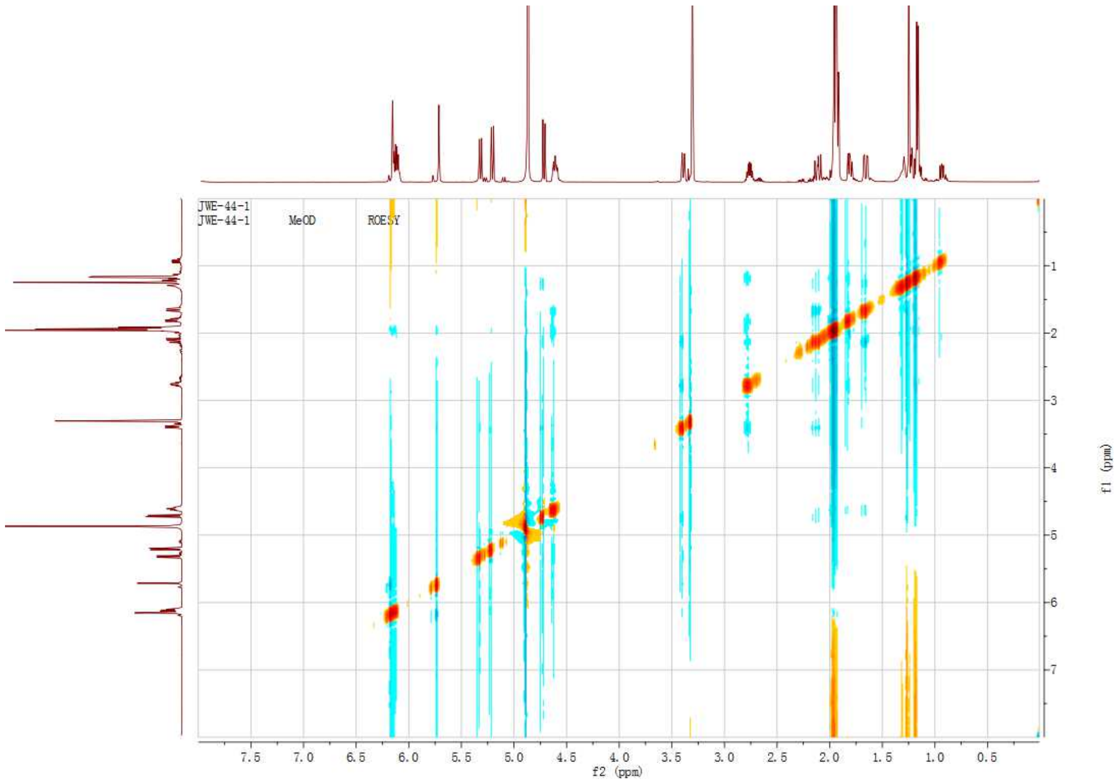

Fig. S12.6 ROESY spectrum (500 MHz) of divaricin B (**12**) in CD_3OD

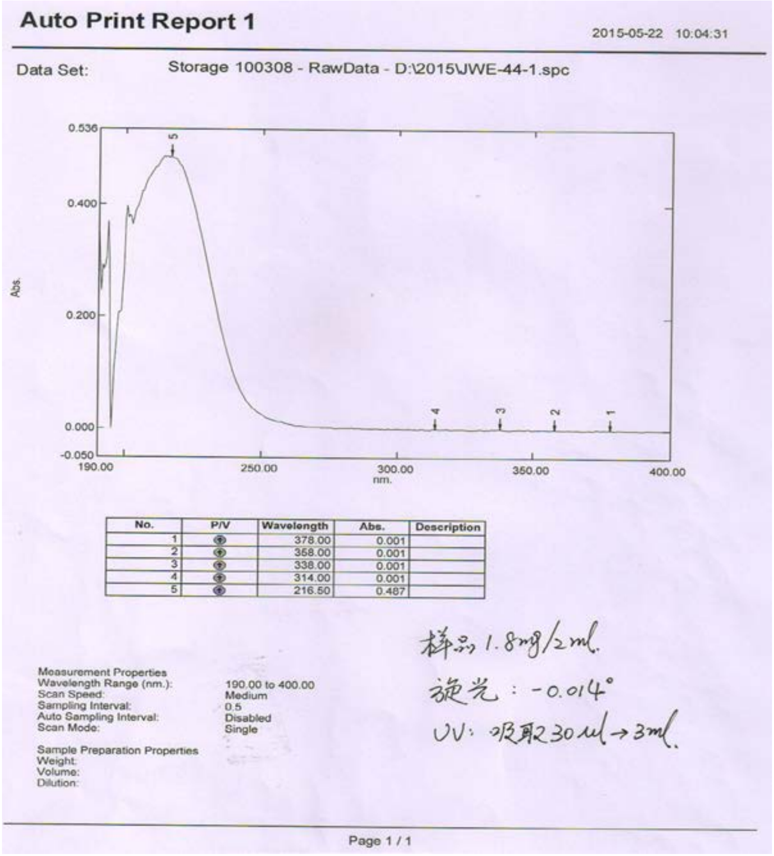

Auto Print Report 1 2015-05-22 10:04:31

Data Set: Storage 100308 - RawData - D:\2015\JWE-44-1.spc

No.	P/V	Wavelength	Abs.	Description
1	⊕	378.00	0.001	
2	⊕	358.00	0.001	
3	⊕	338.00	0.001	
4	⊕	314.00	0.001	
5	⊕	216.50	0.487	

样品 1.8mg/2ml.
旋光：-0.014°
UV: 吸取30ul→3ml.

Measurement Properties
Wavelength Range (nm.): 190.00 to 400.00
Scan Speed: Medium
Sampling Interval: 0.5
Auto Sampling Interval: Disabled
Scan Mode: Single

Sample Preparation Properties
Weight:
Volume:
Dilution:

Page 1 / 1

Fig. S12.7 UV spectrum of divaricin B (**12**)

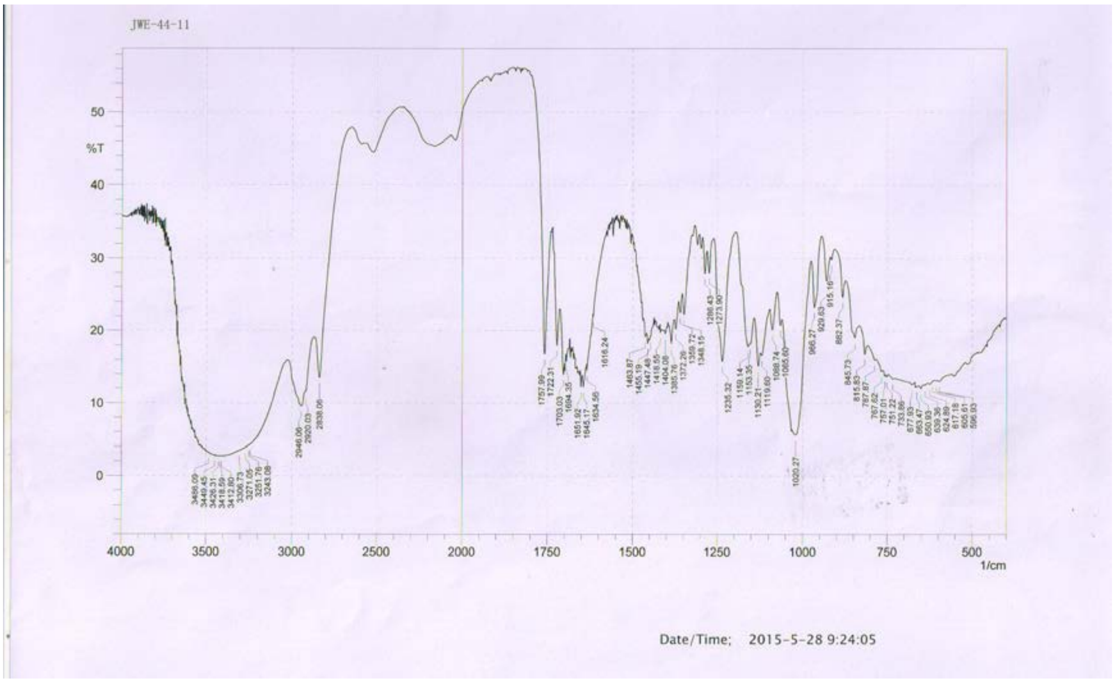

Fig. S12.8 IR spectrum of divaricin B (**12**)

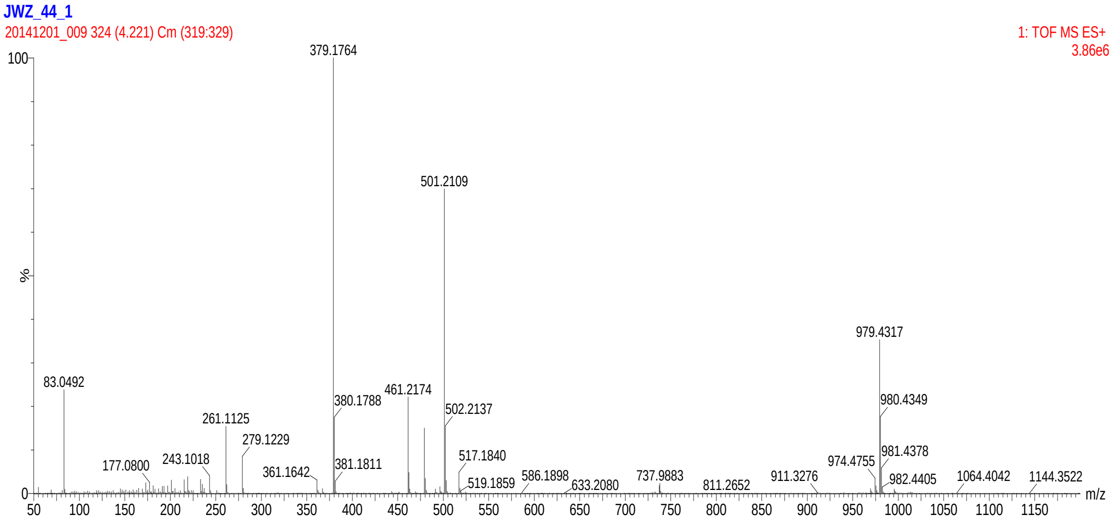

Fig. S12.9 HRESIMS spectrum of divaricin B (**12**)

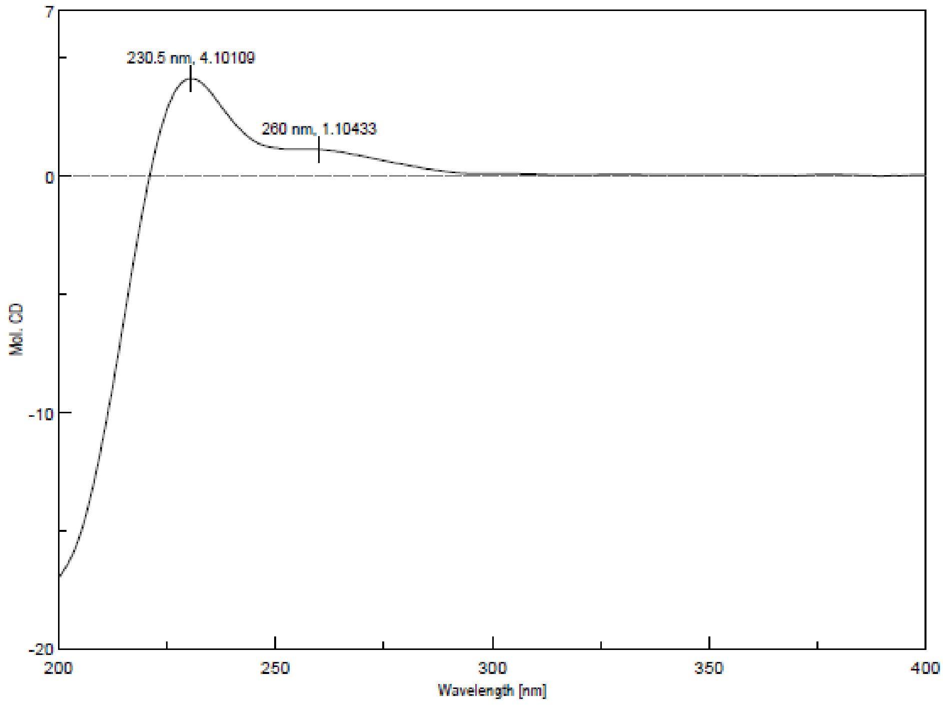

Fig. S12.10 CD spectrum of divaricin B (**12**)

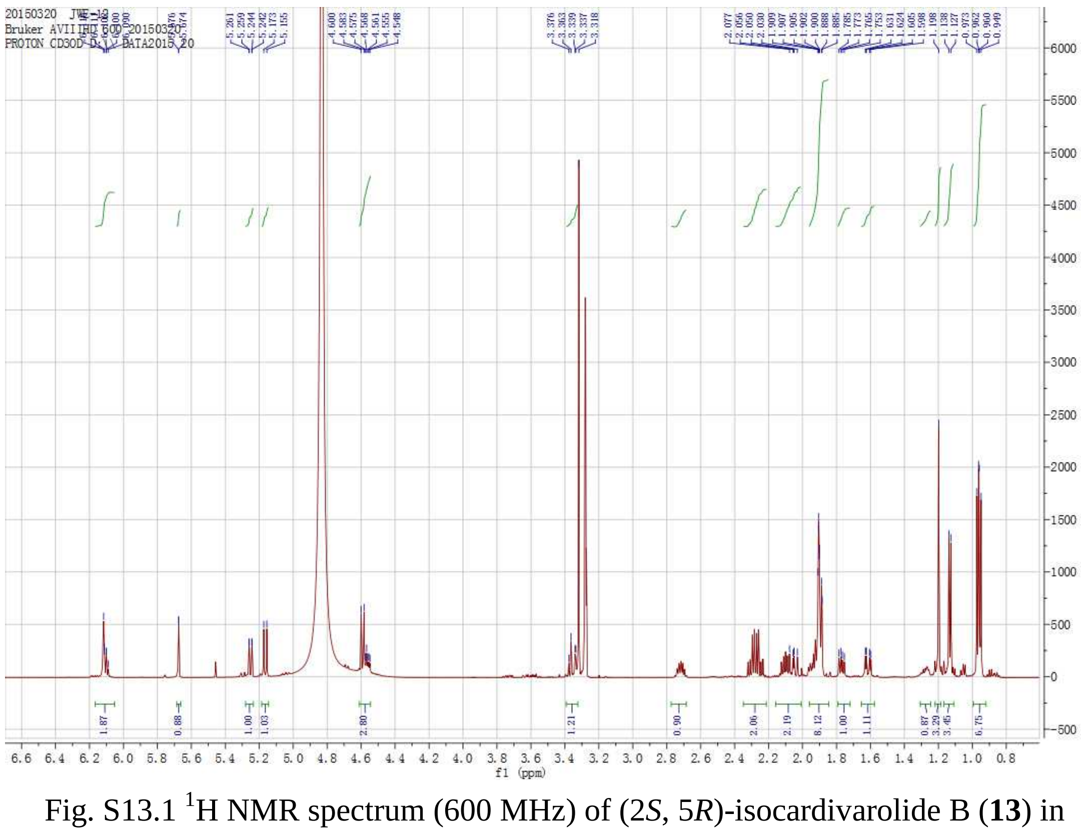

Fig. S13.1 ^{1}H NMR spectrum (600 MHz) of (2*S*, 5*R*)-isocardivarolide B (**13**) in CD_3OD

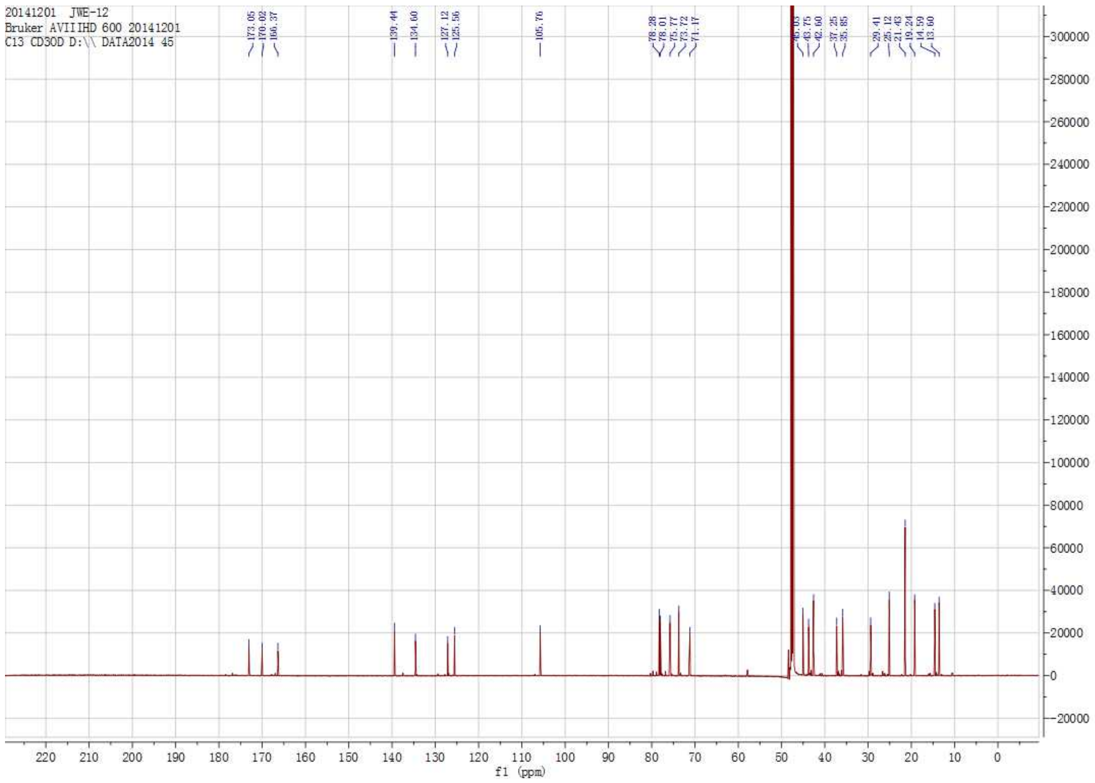

Fig. S13.2 ^{13}C NMR spectrum (150 MHz) of (2*S*, 5*R*)-isocardivarolide B (**13**) in CD_3OD

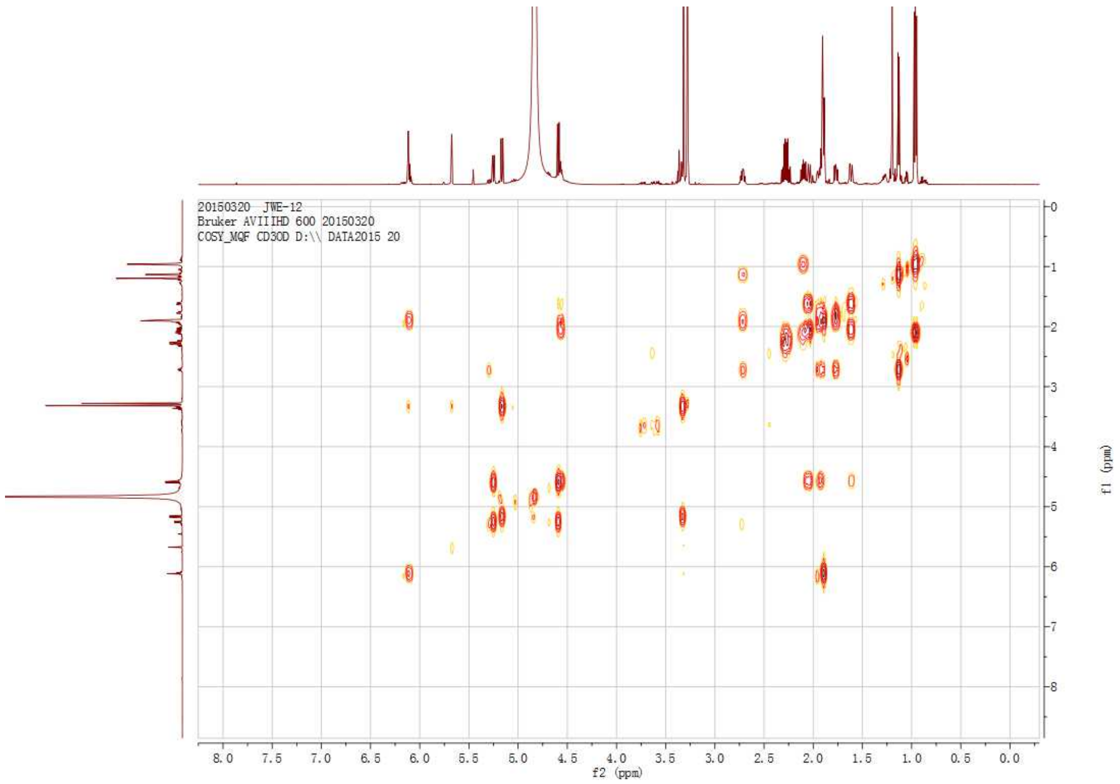

Fig. S13.3 ^{1}H-^{1}H COSY spectrum (600 MHz) of (2*S*, 5*R*)-isocardivarolide B (**13**) in CD_3OD

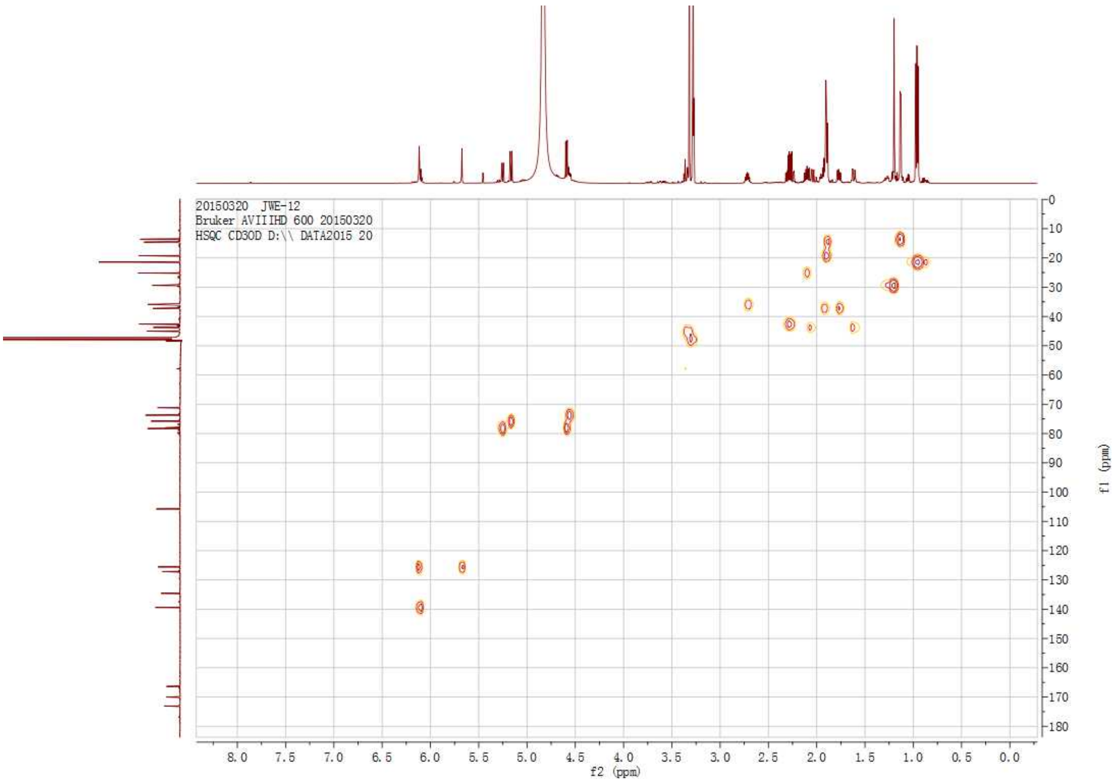

Fig. S13.4 HSQC spectrum (600 MHz) of (2*S*, 5*R*)-isocardivarolide B (**13**) in CD_3OD

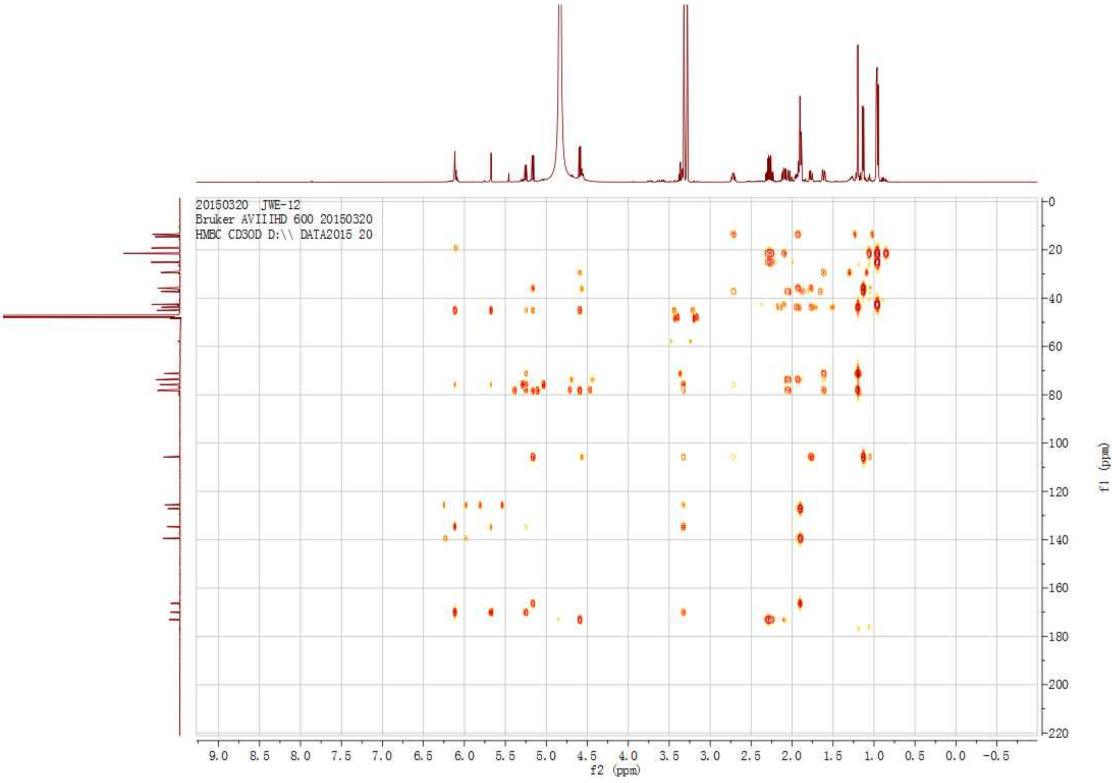

Fig. S13.5 HMBC spectrum (600 MHz) of (2*S*, 5*R*)-isocardivarolide B (**13**) in CD_3OD

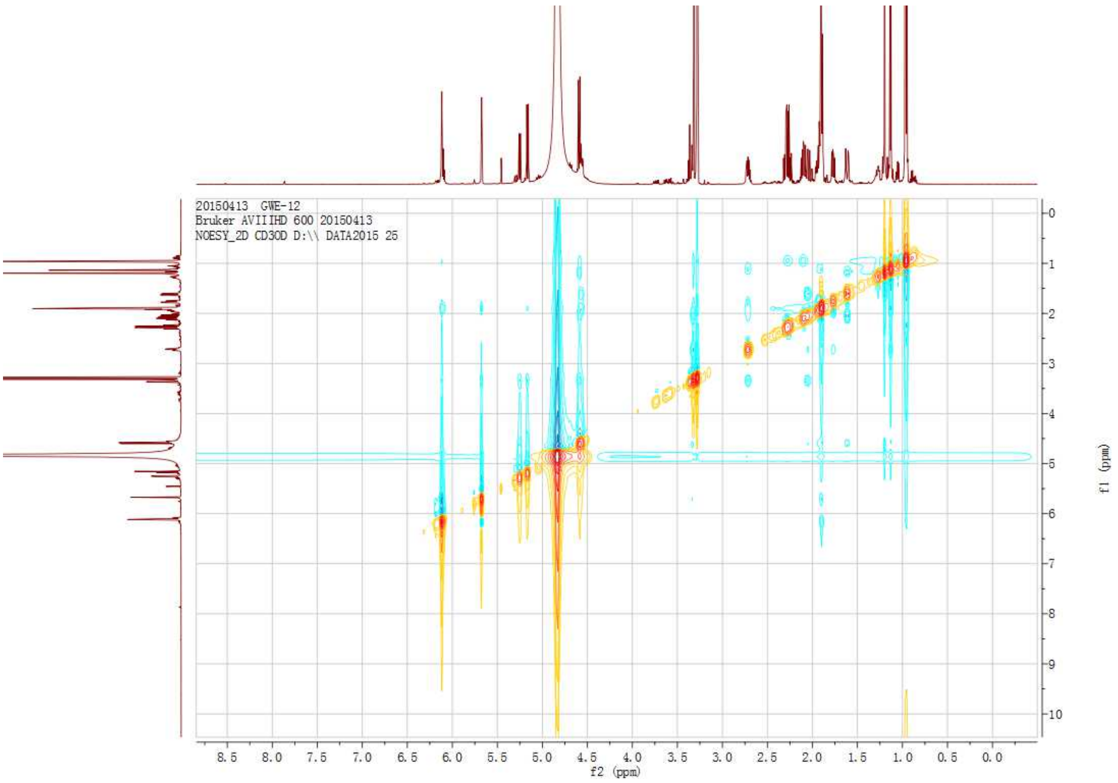

Fig. S13.6 NOESY spectrum (600 MHz) of (2*S*, 5*R*)-isocardivarolide B (**13**) in CD_3OD

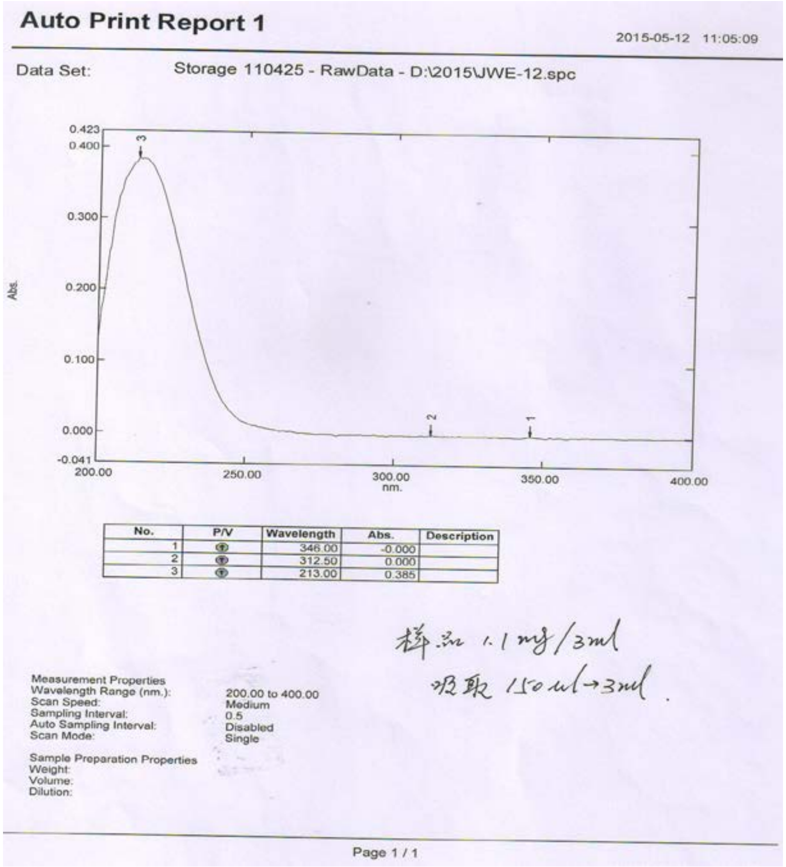

No.	P/V	Wavelength	Abs.	Description
1		346.00	-0.000	
2		312.50	0.000	
3		213.00	0.385	

Fig. S13.7 UV spectrum of (2*S*, 5*R*)-isocardivarolide B (**13**)

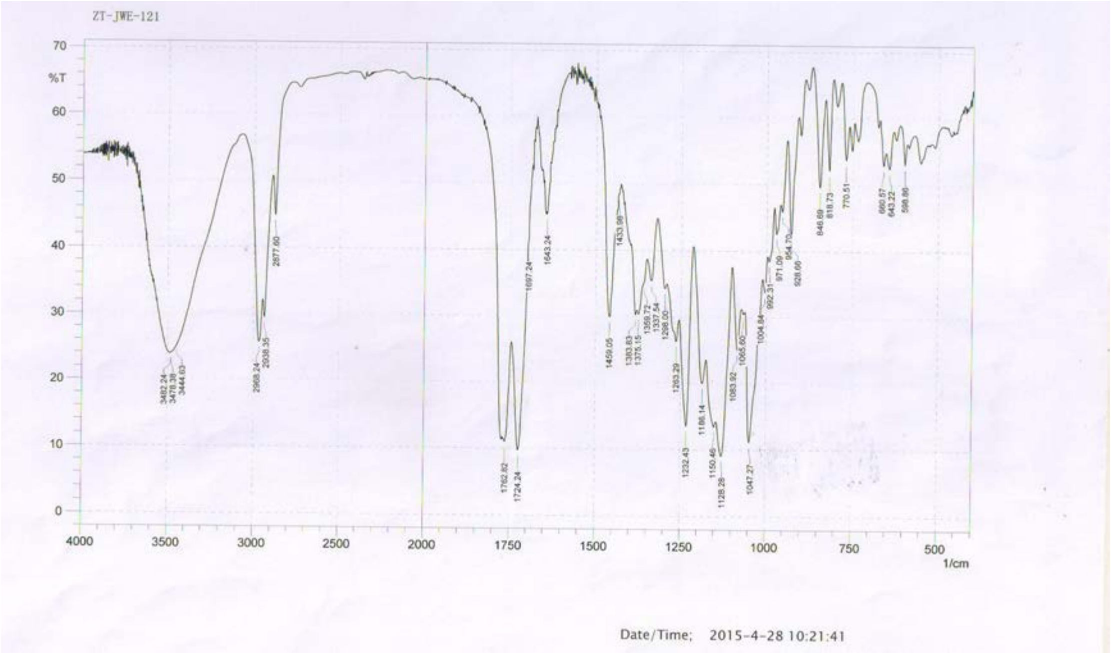

Fig. S13.8 IR spectrum of (2*S*, 5*R*)-isocardivarolide B (**13**)

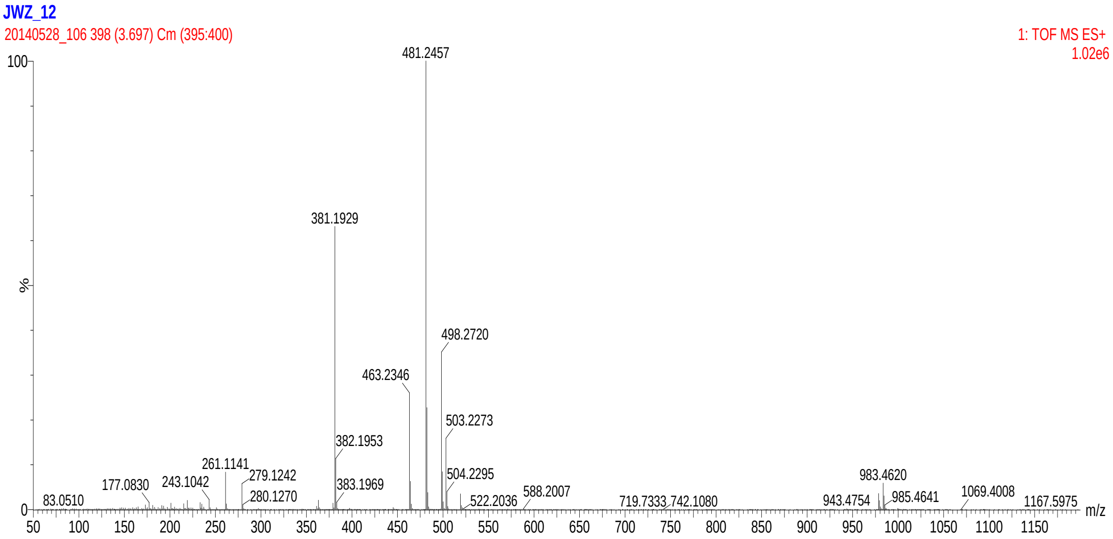

Fig. S13.9 HRESIMS spectrum of (2*S*, 5*R*)-isocardivarolide B (**13**)

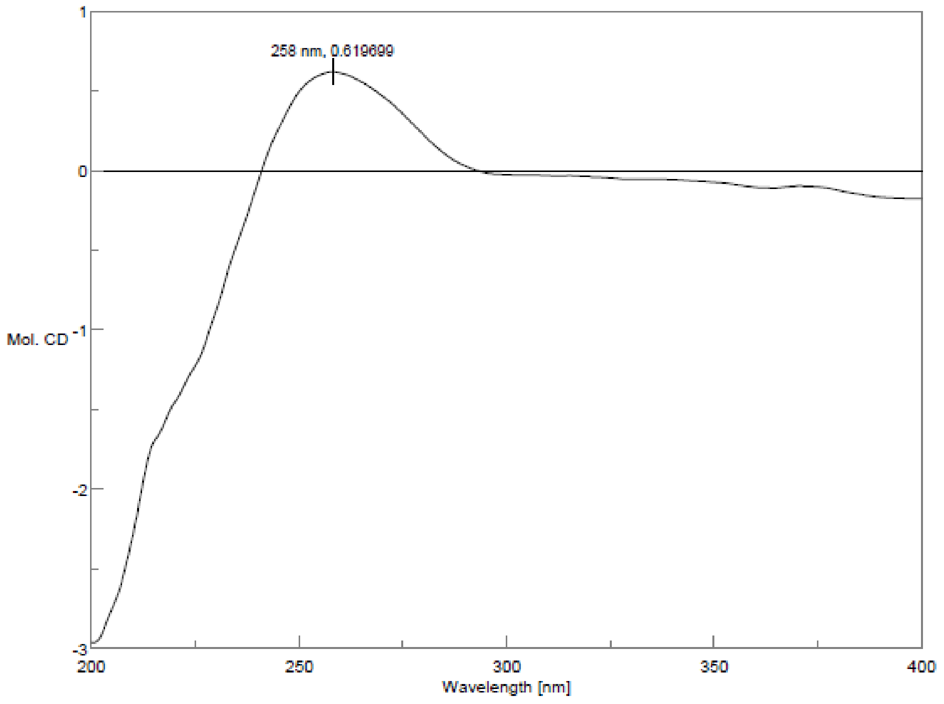

Fig. S13.10 CD spectrum of (2*S*, 5*R*)-isocardivarolide B (**13**)

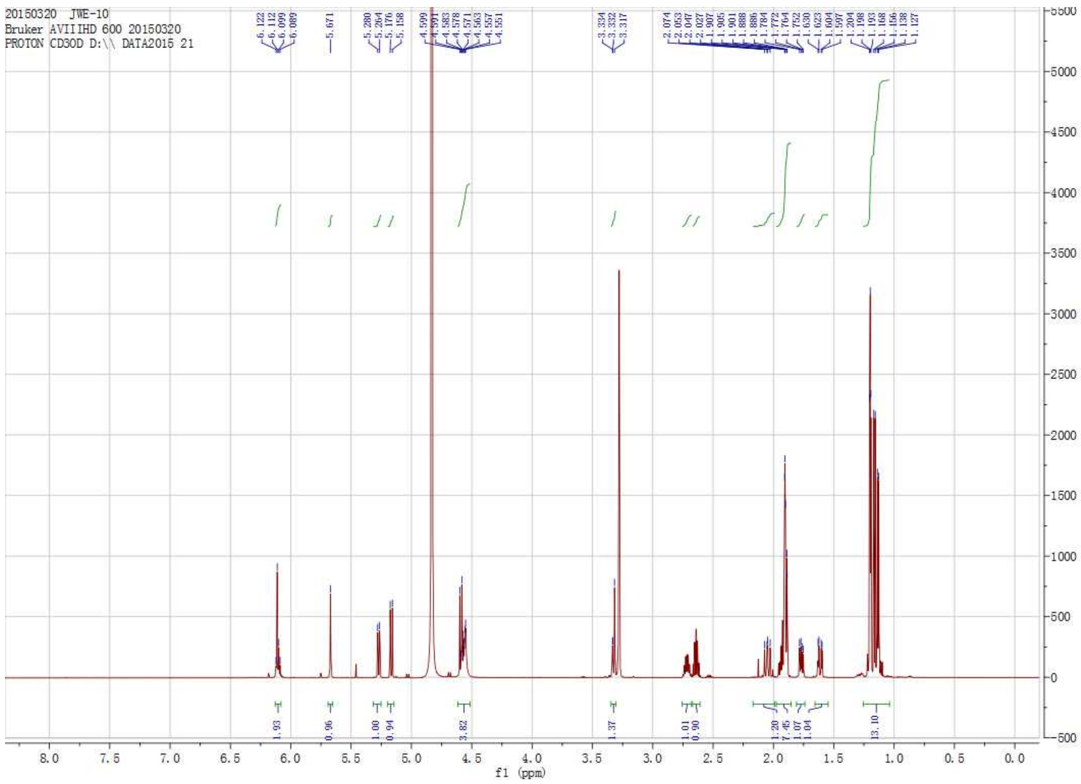

Fig. S14.1 ^{1}H NMR spectrum (600 MHz) of (2*S*, 5*R*)-isocardivarolide C (**14**) in CD_3OD

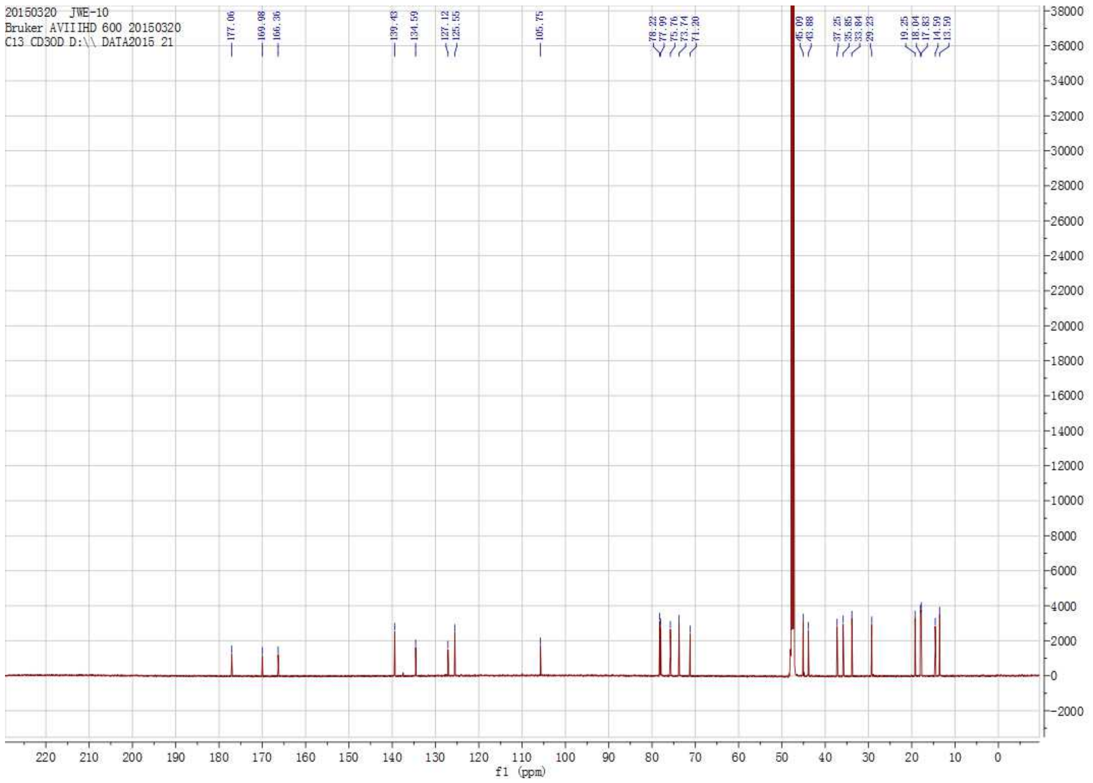

Fig. S14.2 ^{13}C NMR spectrum (150 MHz) of (2*S*, 5*R*)-isocardivarolide C (**14**) in CD_3OD

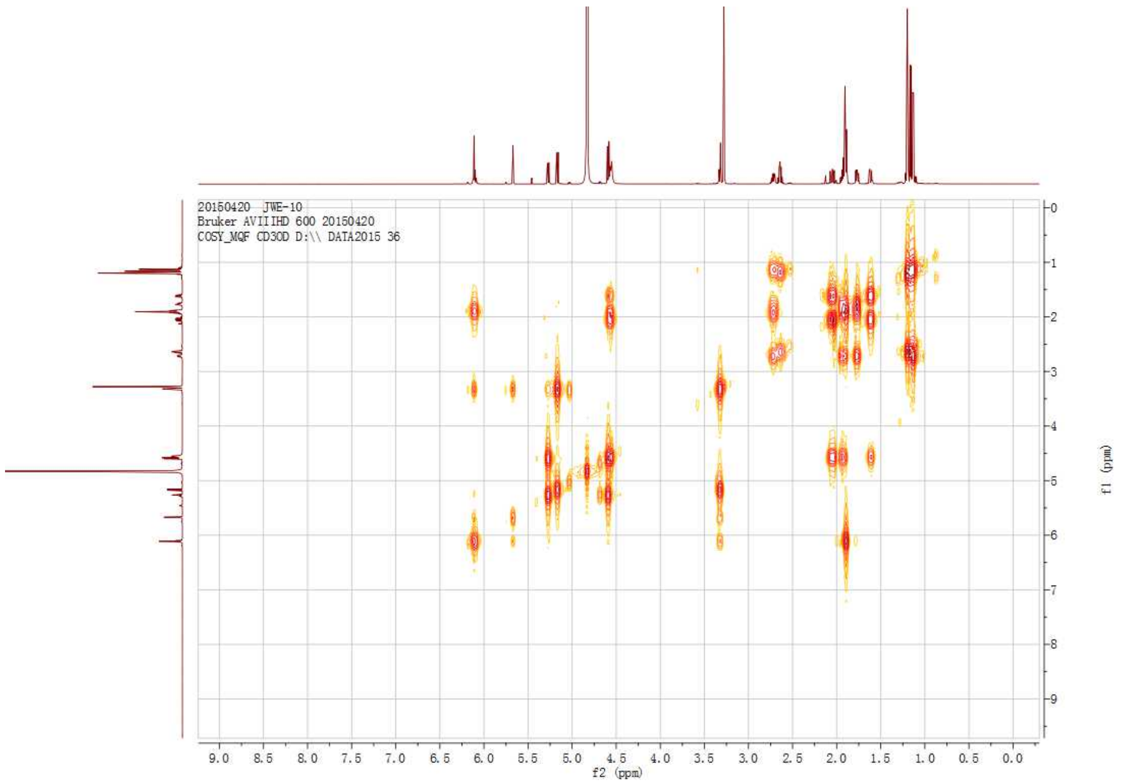

Fig. S14.3 ^{1}H-^{1}H COSY spectrum (600 MHz) of (2*S*, 5*R*)-isocardivarolide C (**14**) in CD_3OD

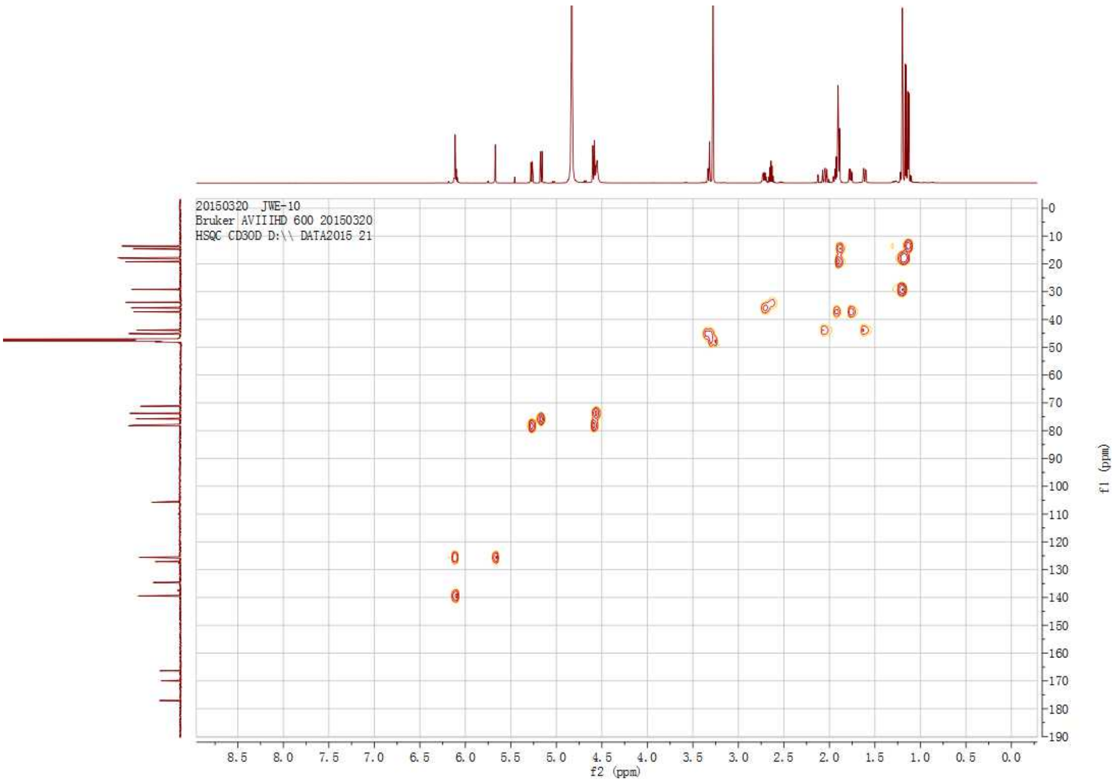

Fig. S14.4 HSQC spectrum (600 MHz) of (2*S*, 5*R*)-isocardivarolide C (**14**) in CD_3OD

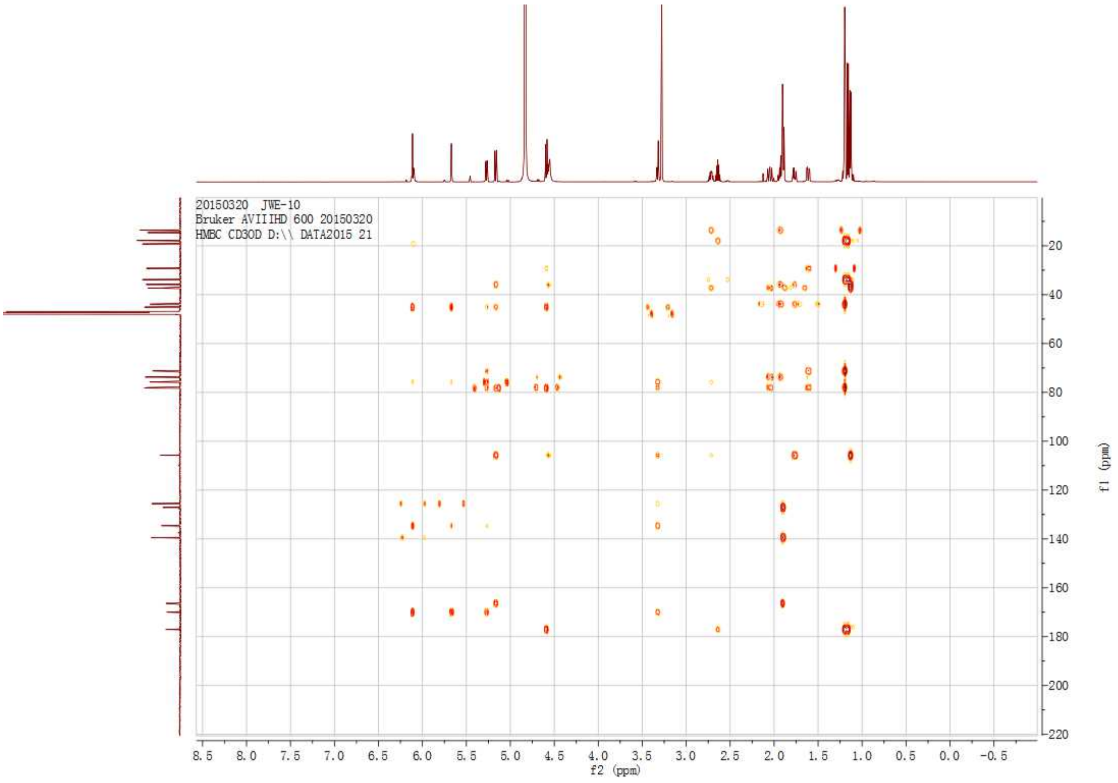

Fig. S14.5 HMBC spectrum (600 MHz) of (2*S*, 5*R*)-isocardivarolide C (**14**) in CD_3OD

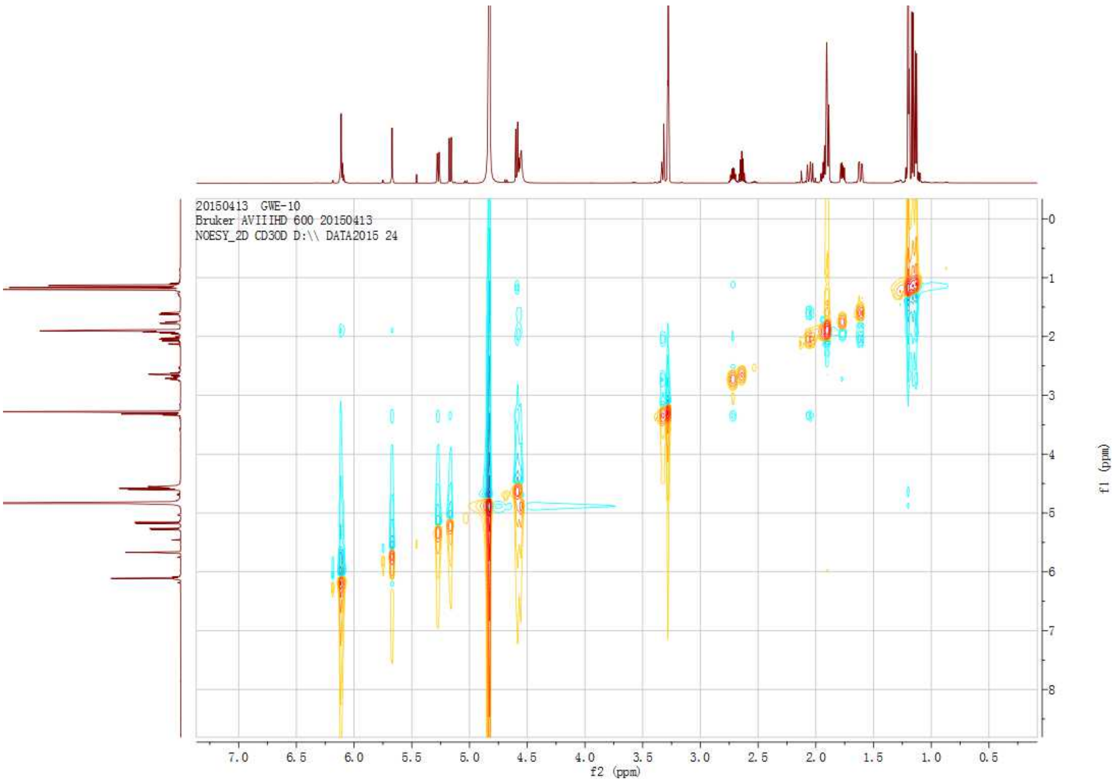

Fig. S14.6 NOESY spectrum (600 MHz) of (2*S*, 5*R*)-isocardivarolide C (**14**) in CD_3OD

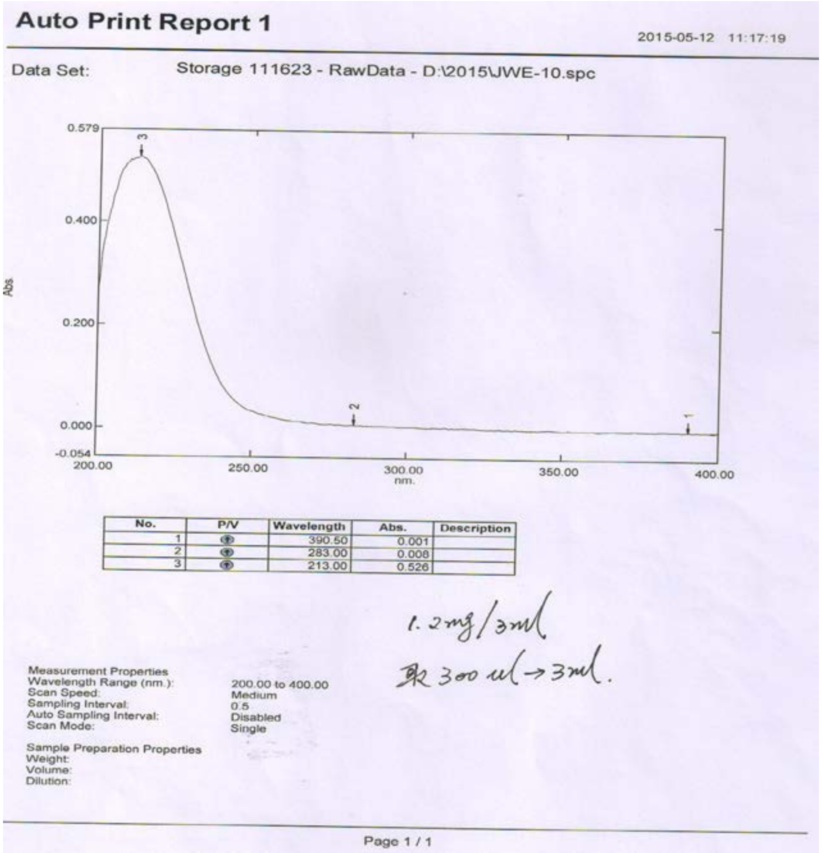

Fig. S14.7 UV spectrum of (2*S*, 5*R*)-isocardivarolide C (**14**)

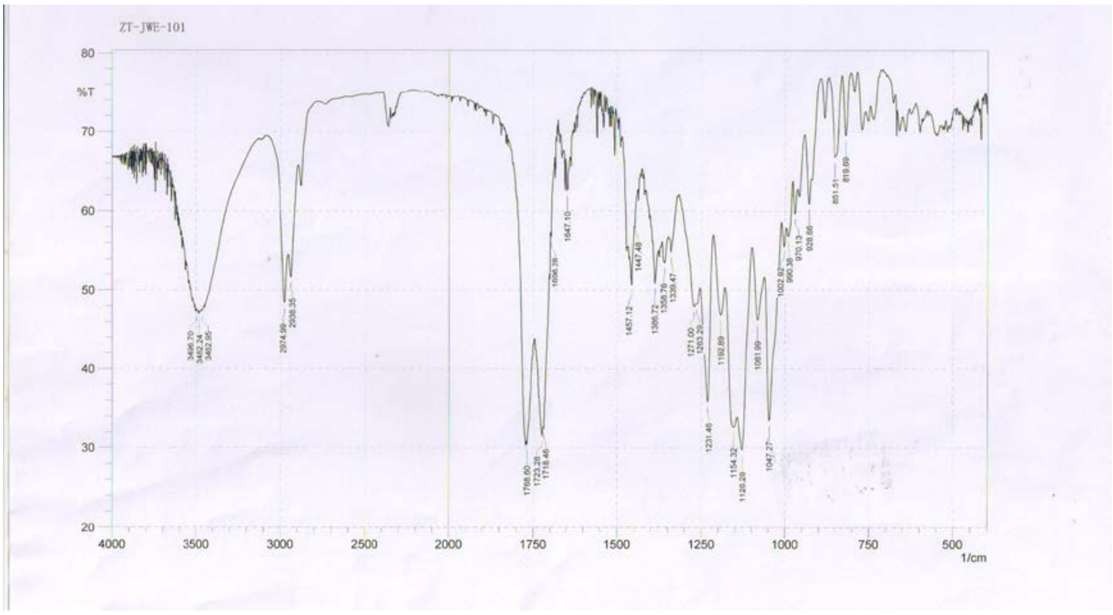

Fig. S14.8 IR spectrum of (2*S*, 5*R*)-isocardivarolide C (**14**)

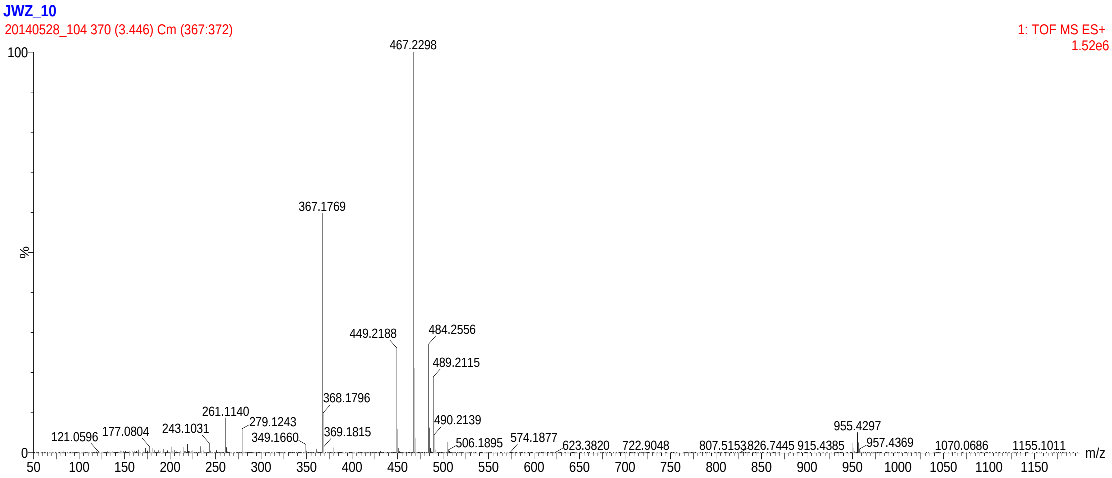

Fig. S14.9 HRESIMS spectrum of (2*S*, 5*R*)-isocardivarolide C (**14**)

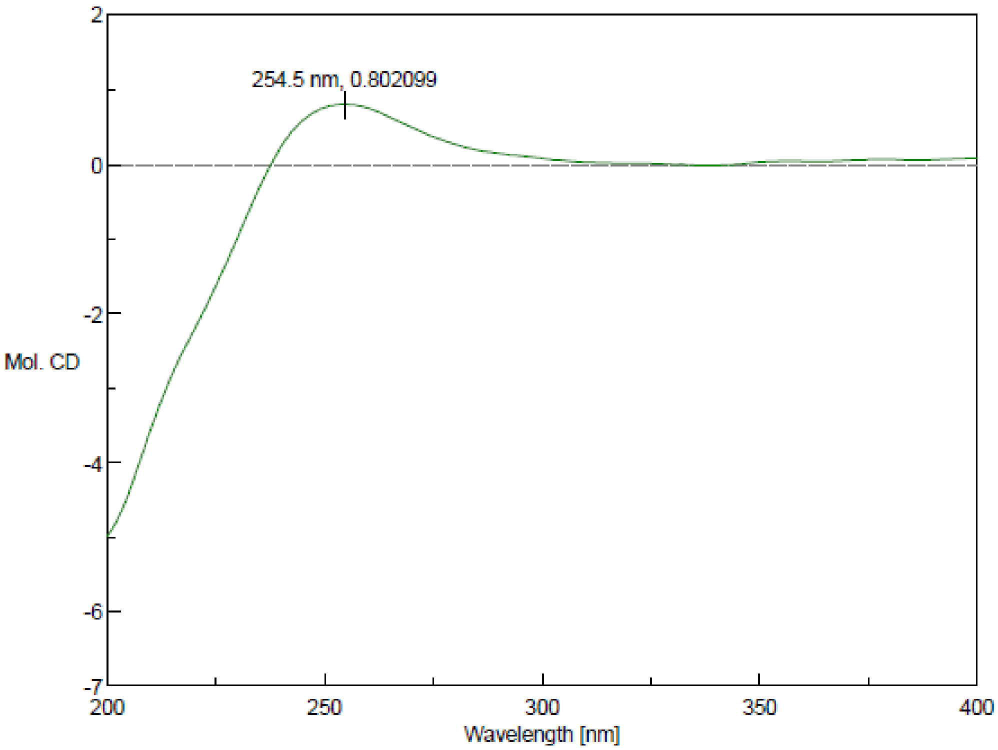

Fig. S14.10 CD spectrum of (2*S*, 5*R*)-isocardivarolide C (**14**)

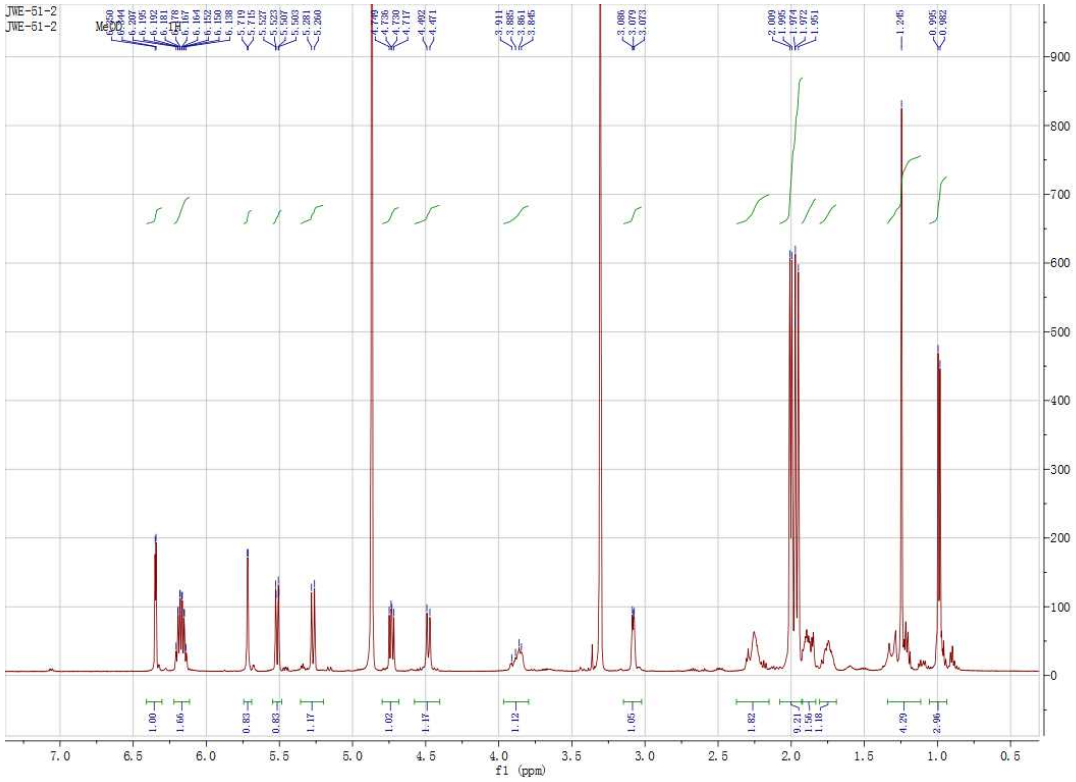

Fig. S15.1 ^{1}H NMR spectrum (500 MHz) of cardivarolide F (**15**) in CD_3OD

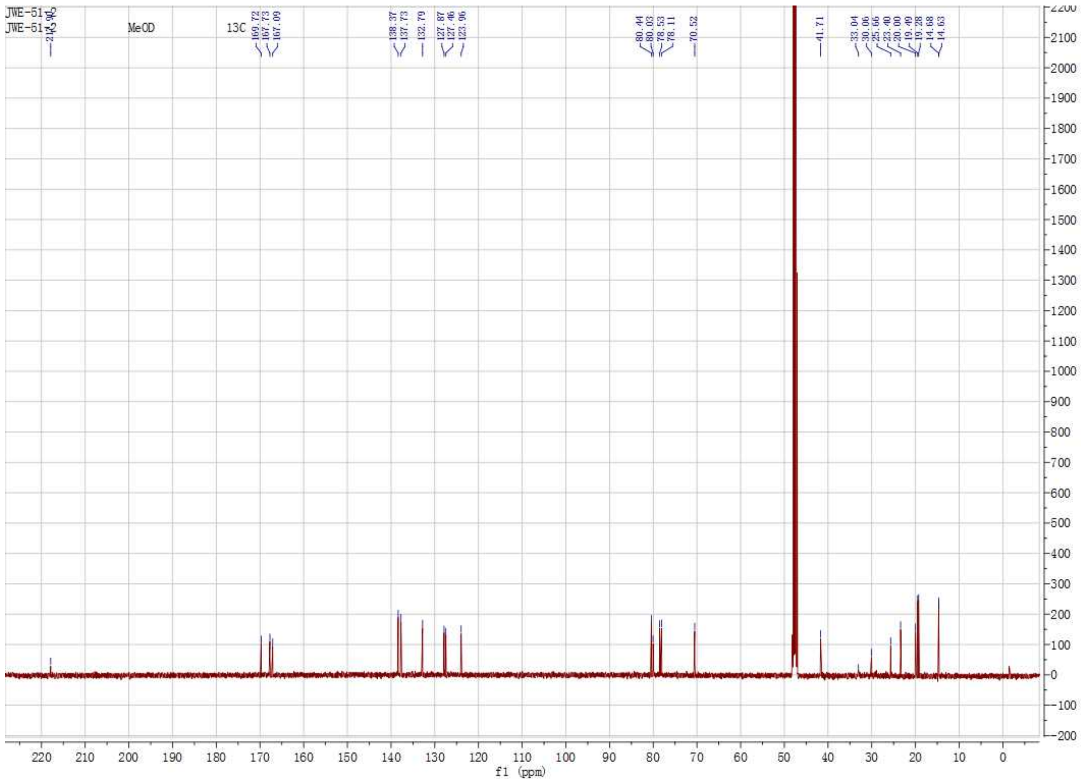

Fig. S15.2 ^{13}C NMR spectrum (125 MHz) of cardivarolide F (**15**) in CD_3OD

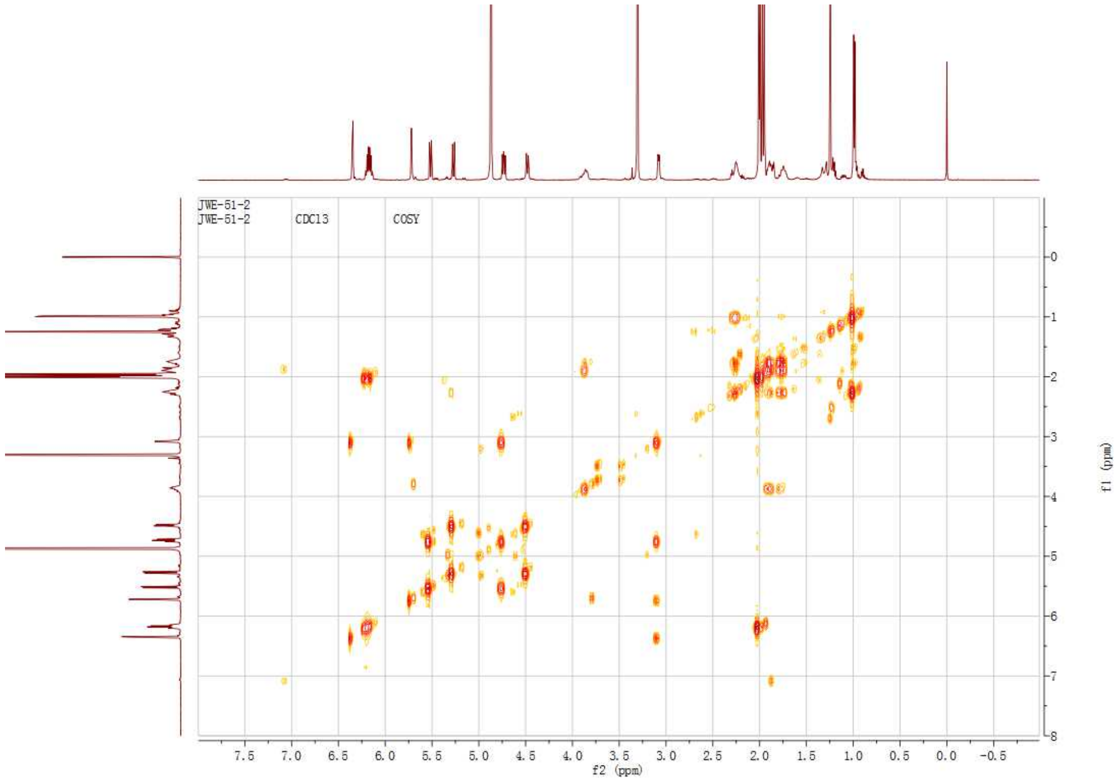

Fig. S15.3 ^{1}H-^{1}H COSY spectrum (500 MHz) of cardivarolide F (**15**) in CD_3OD

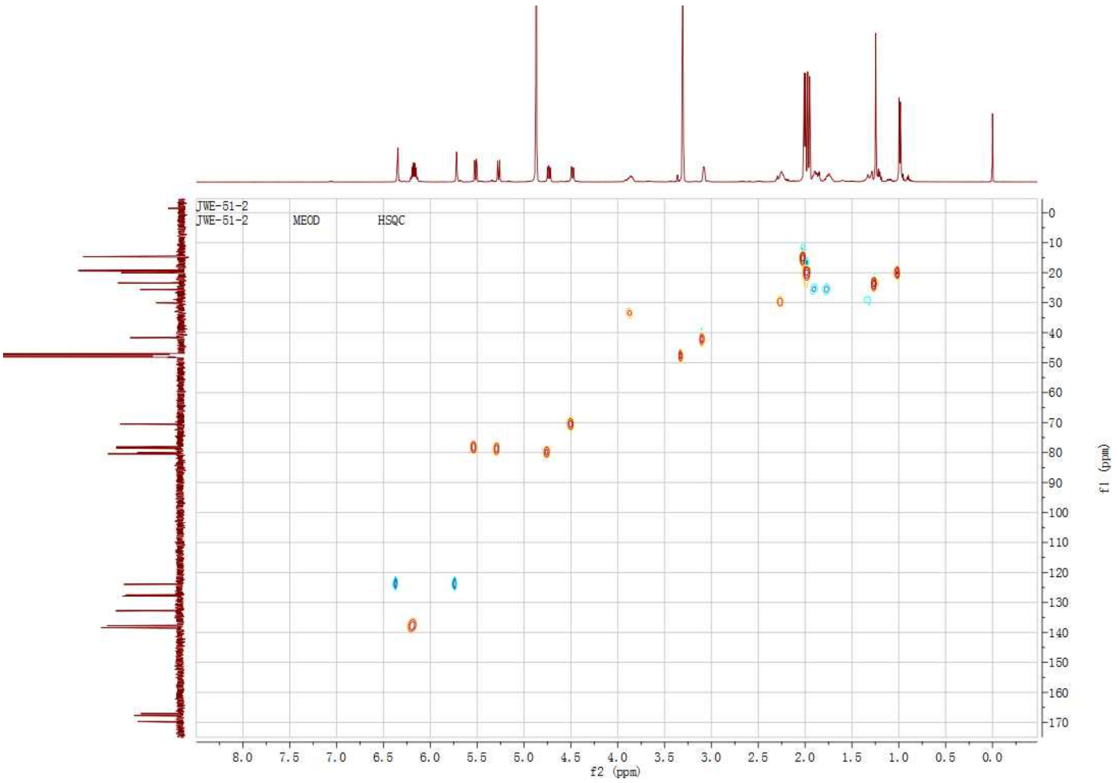

Fig. S15.4 HSQC spectrum (500 MHz) of cardivarolide F (**15**) in CD_3OD

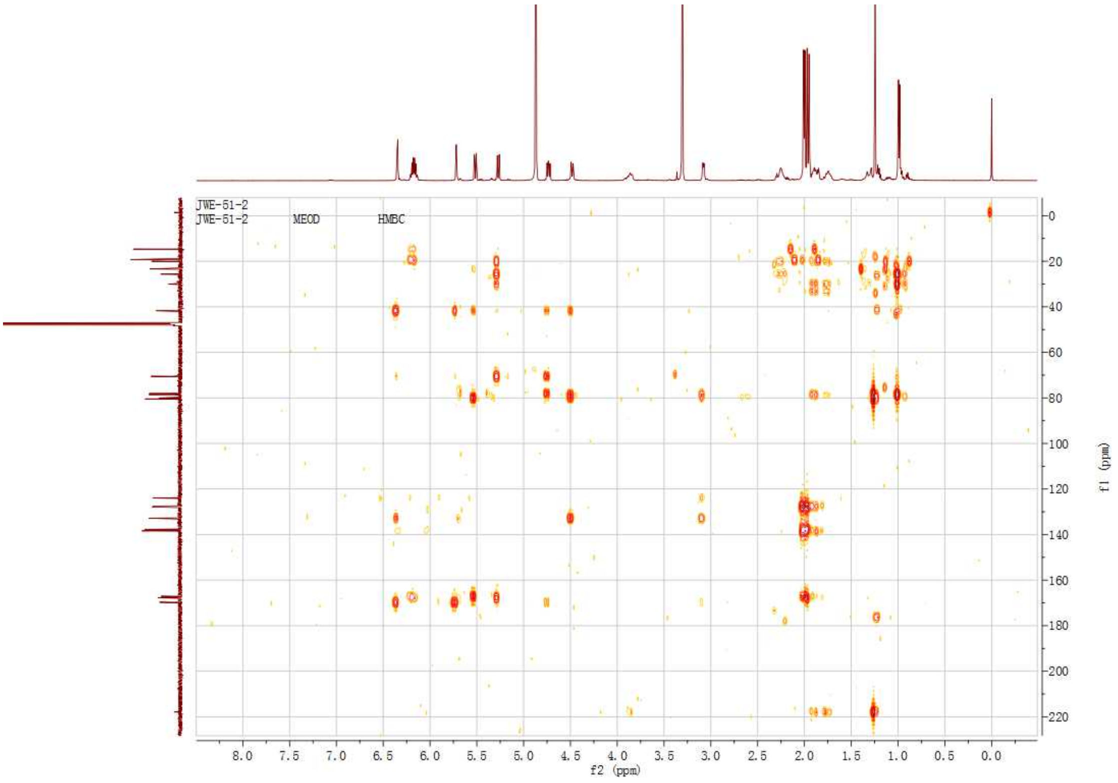

Fig. S15.5 HMBC spectrum (500 MHz) of cardivarolide F (**15**) in CD_3OD

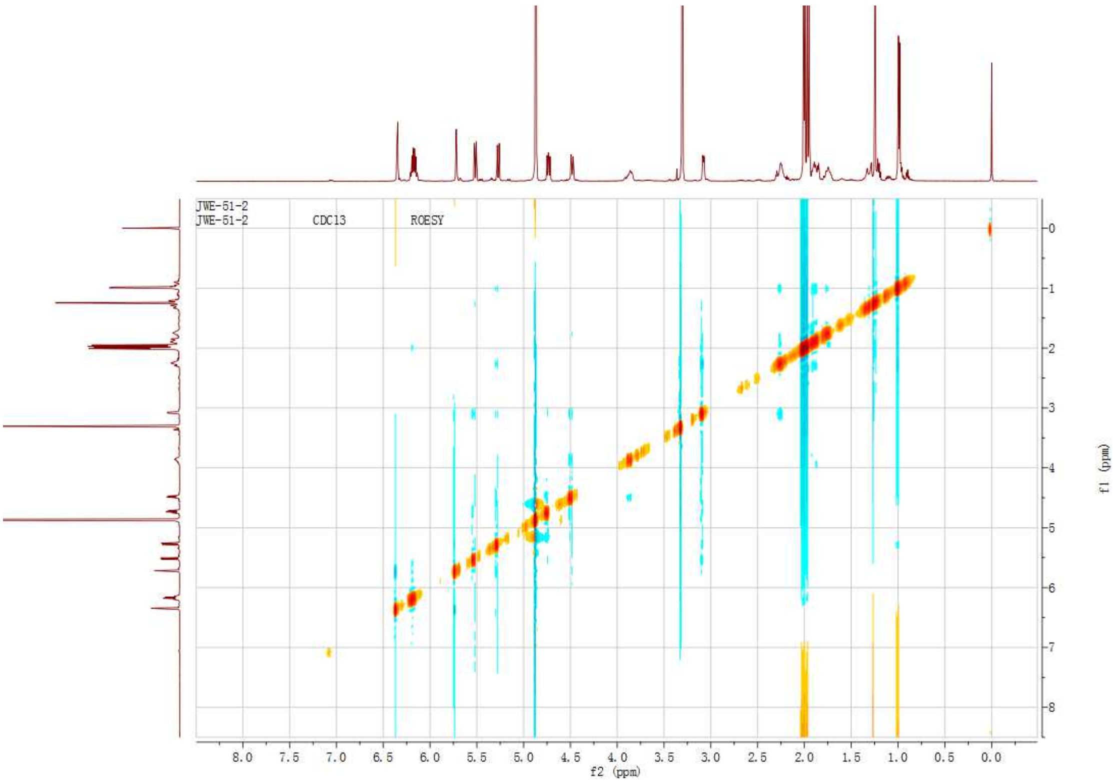

Fig. S15.6 ROESY spectrum (500 MHz) of cardivarolide F (**15**) in CD_3OD

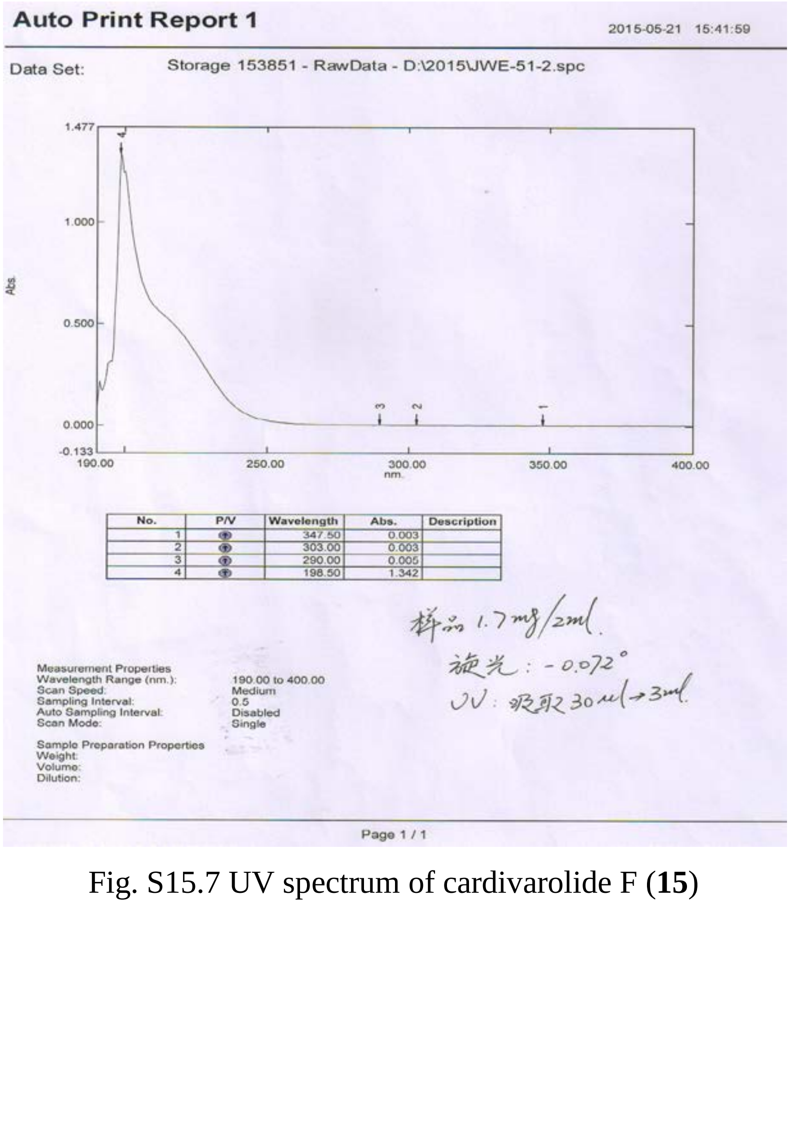

Fig. S15.7 UV spectrum of cardivarolide F (**15**)

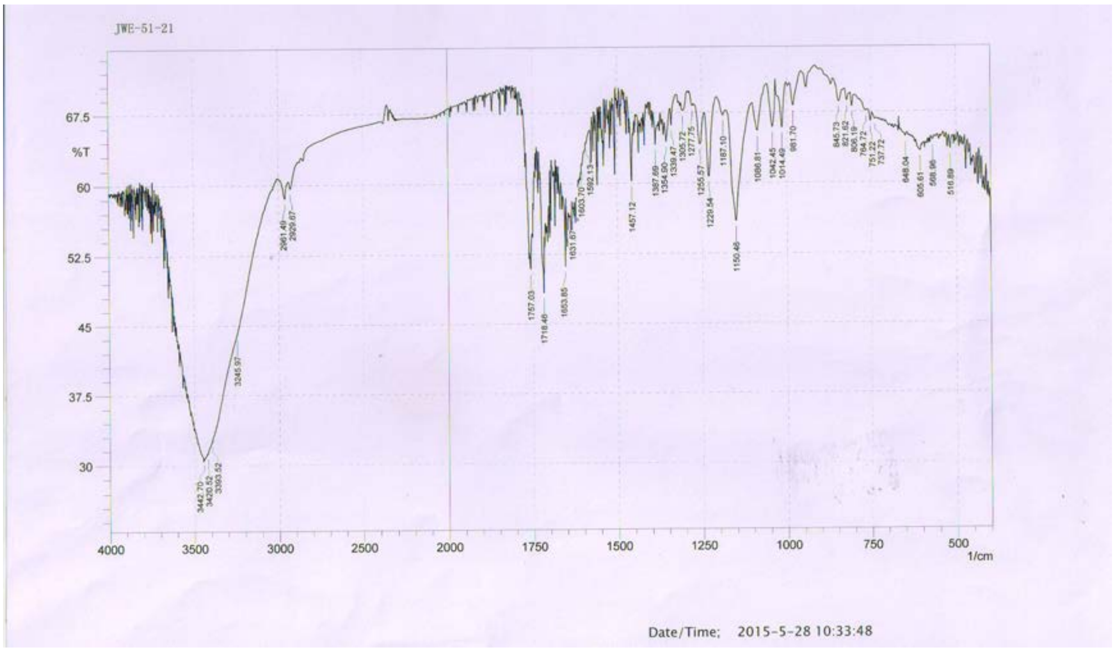

Fig. S15.8 IR spectrum of cardivarolide F (**15**)

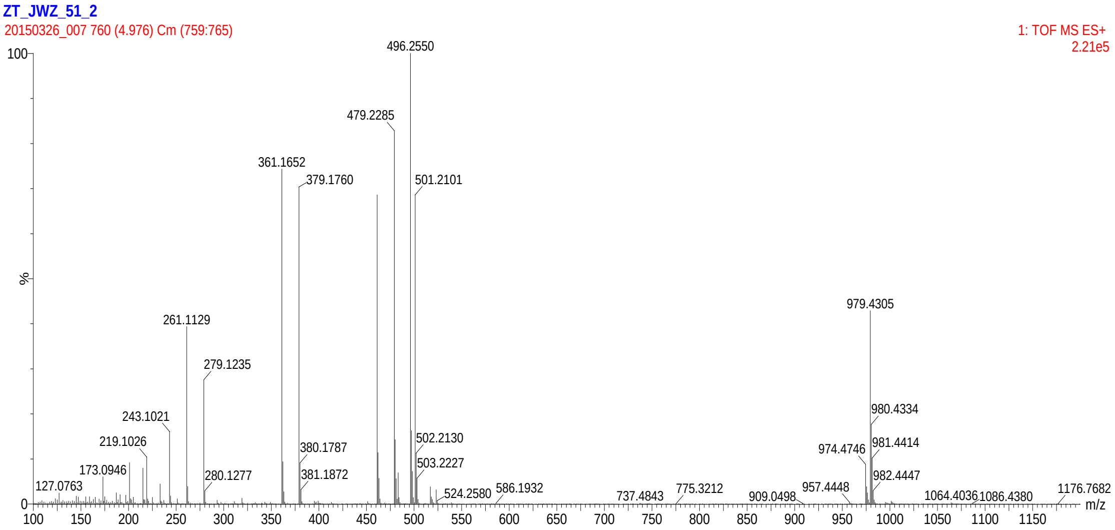

Fig. S15.9 HRESIMS spectrum of cardivarolide F (**15**)

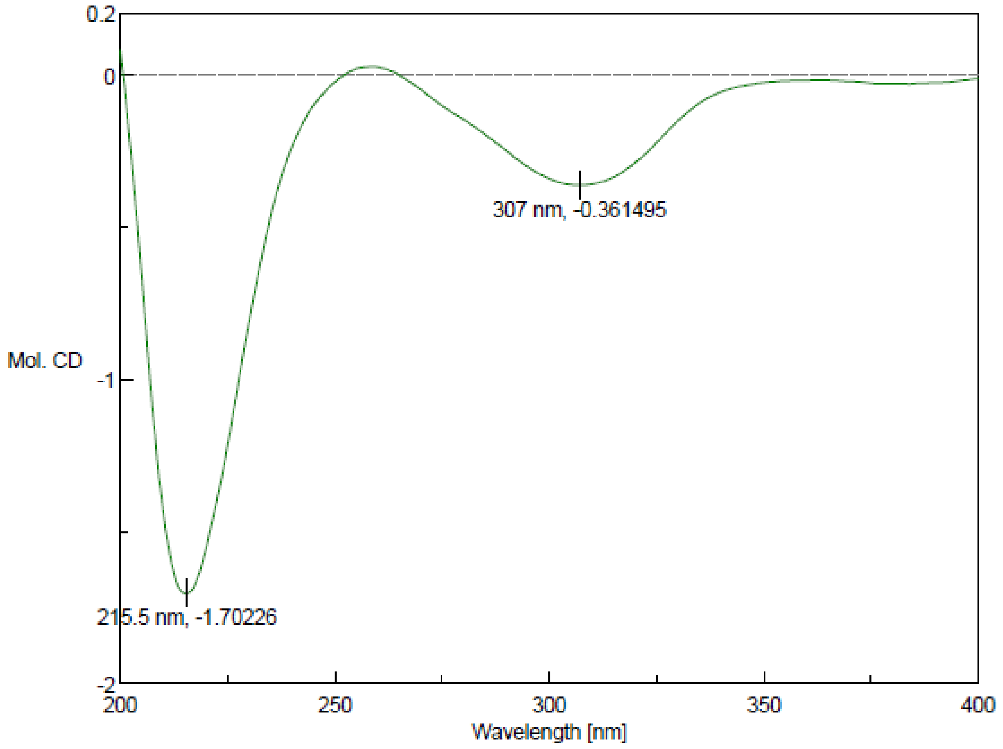

Fig. S15.10 CD spectrum of cardivarolide F (**15**)

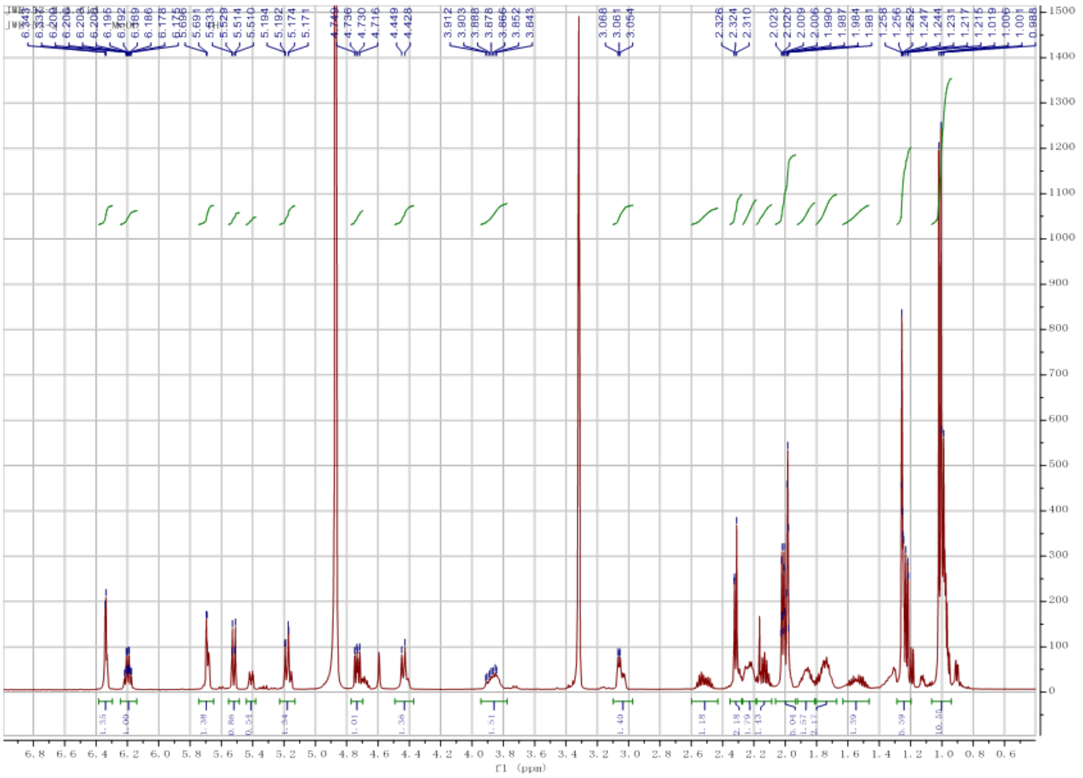

Fig. S16.1 ^{1}H NMR spectrum (500 MHz) of cardivarolide G (**16**) in CD_3OD

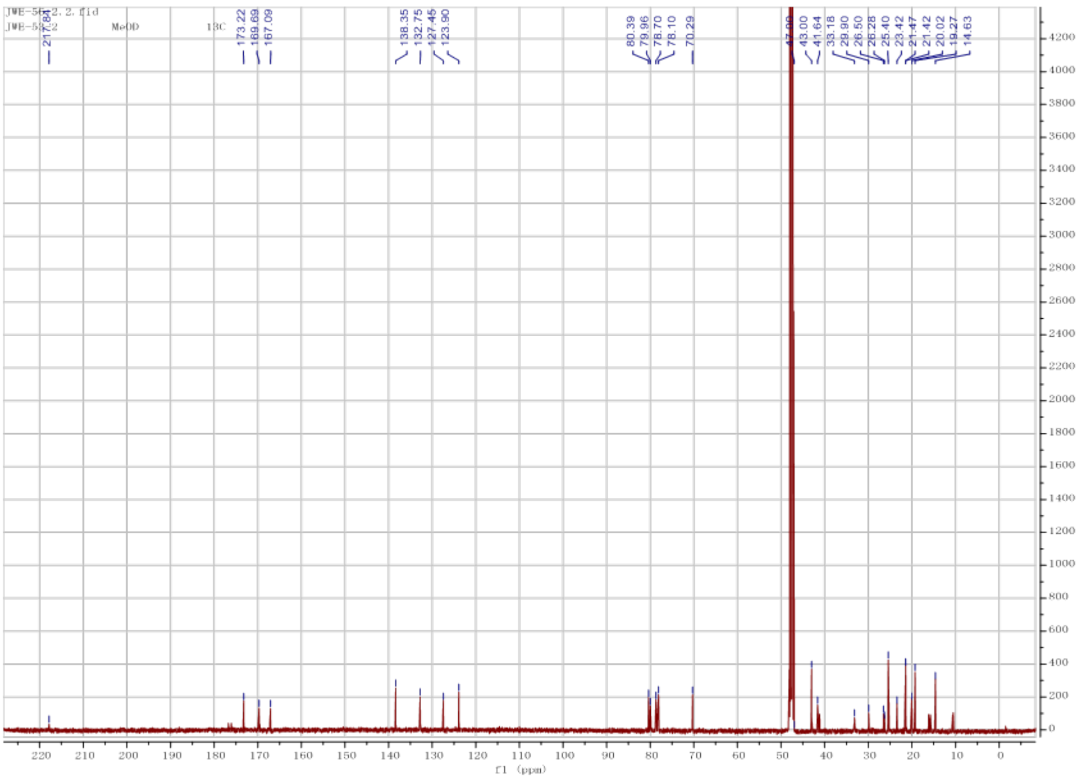

Fig. S16.2 ^{13}C NMR spectrum (125 MHz) of cardivarolide G (**16**) in CD_3OD

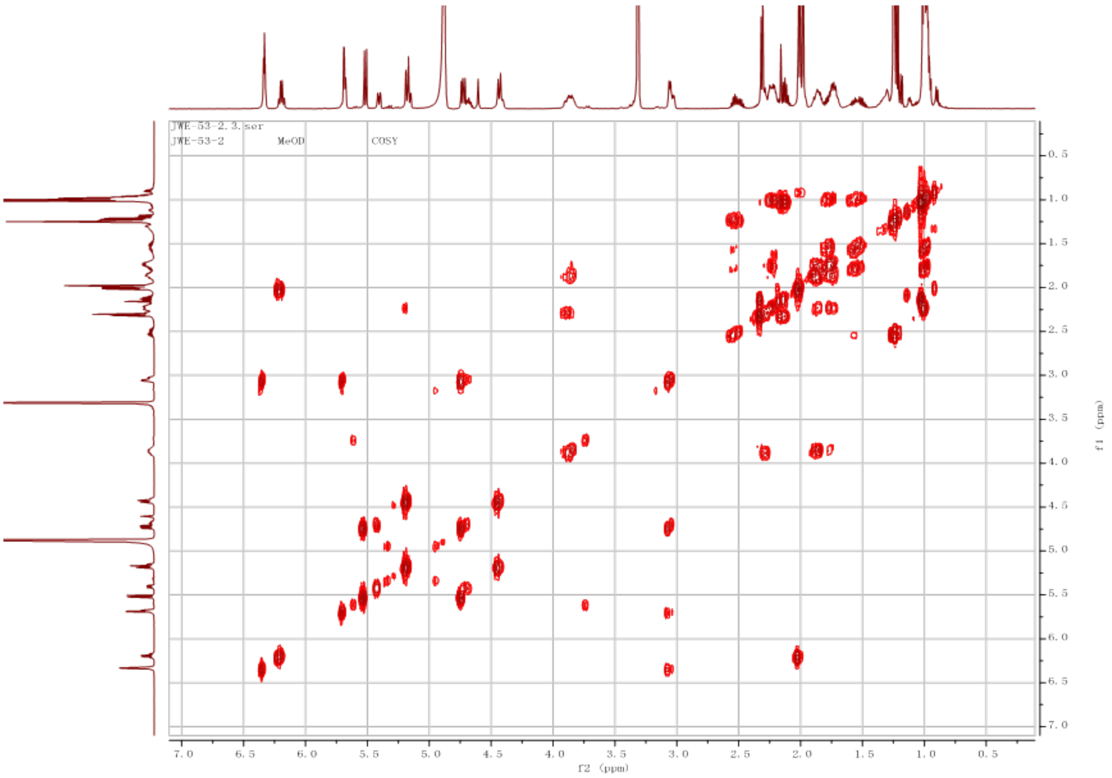

Fig. S16.3 ^{1}H-^{1}H COSY spectrum (500 MHz) of cardivarolide G (**16**) in CD_3OD

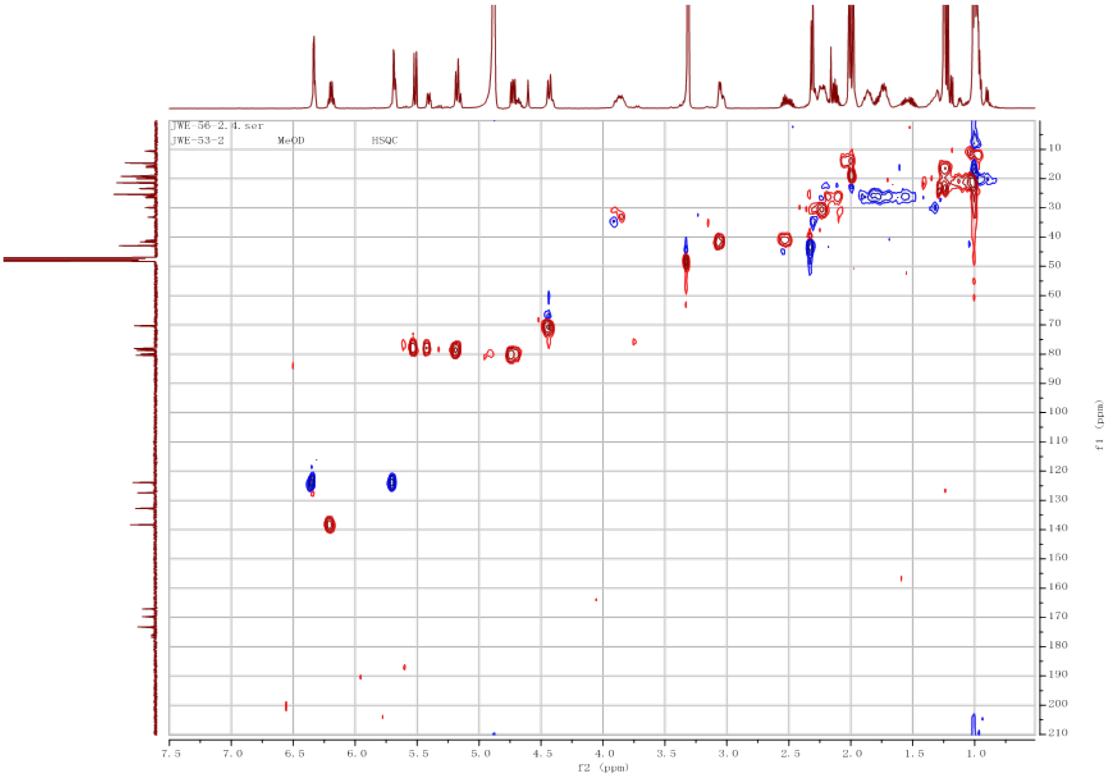

Fig. S16.4 HSQC spectrum (500 MHz) of cardivarolide G (**16**) in CD_3OD

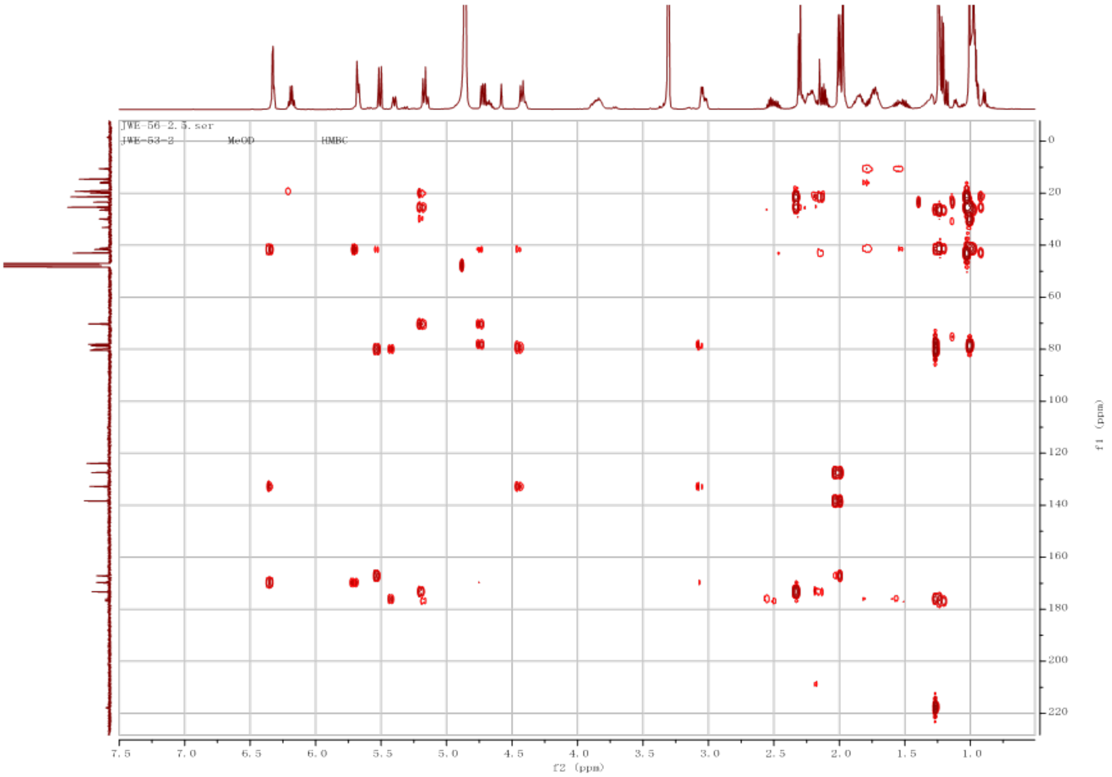

Fig. S16.5 HMBC spectrum (500 MHz) of cardivarolide G (**16**) in CD_3OD

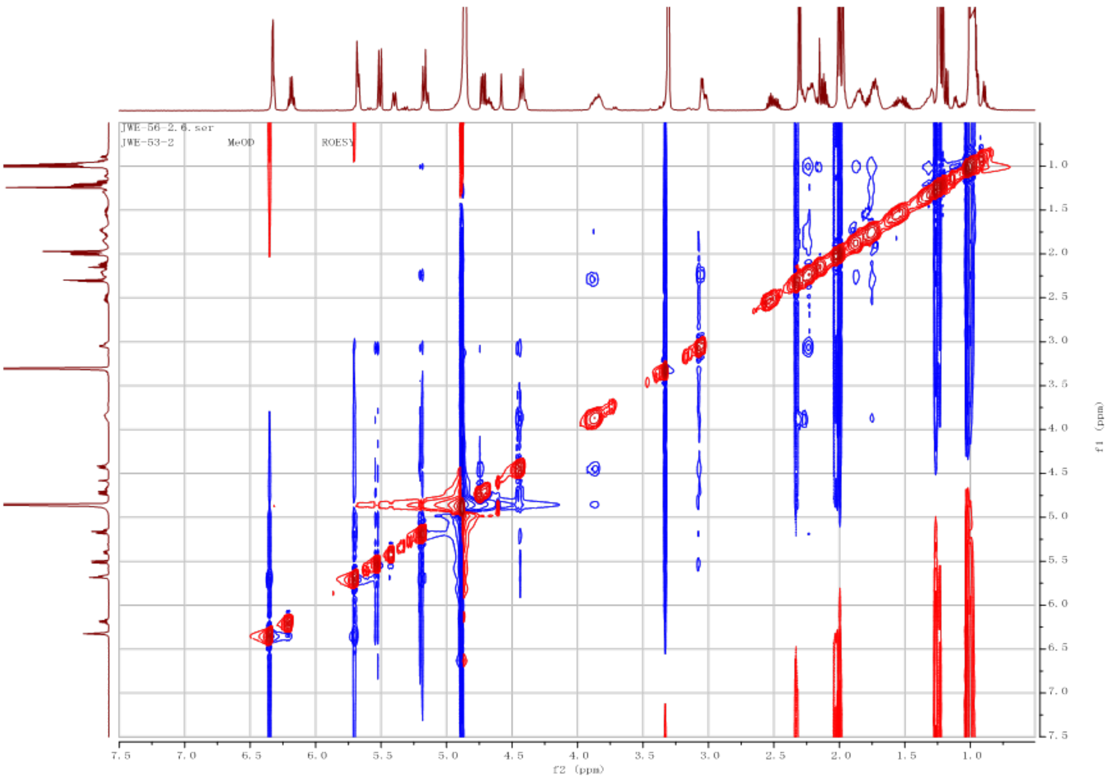

Fig. S16.6 ROESYspectrum (500 MHz) of cardivarolide G (**16**) in CD_3OD

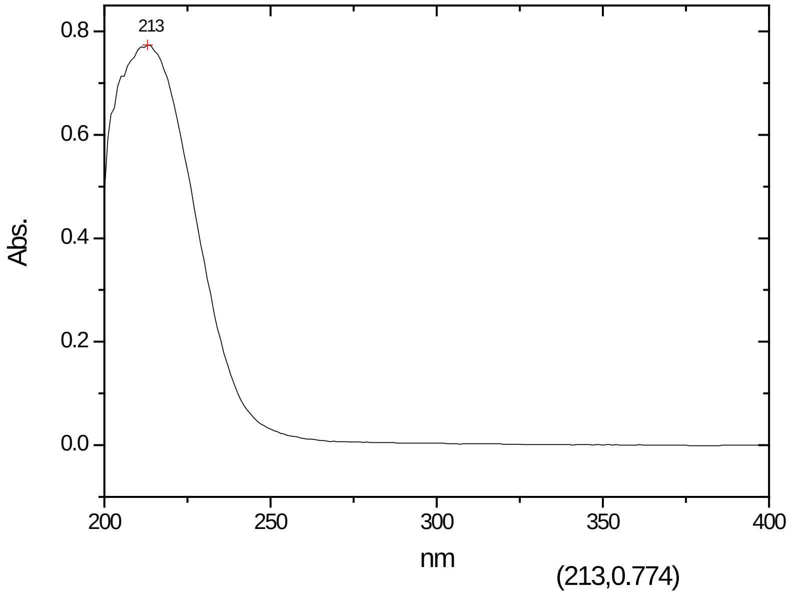

Fig. S16.7 UV spectrum (500 MHz) of cardivarolide G (**16**) in CD_3OD

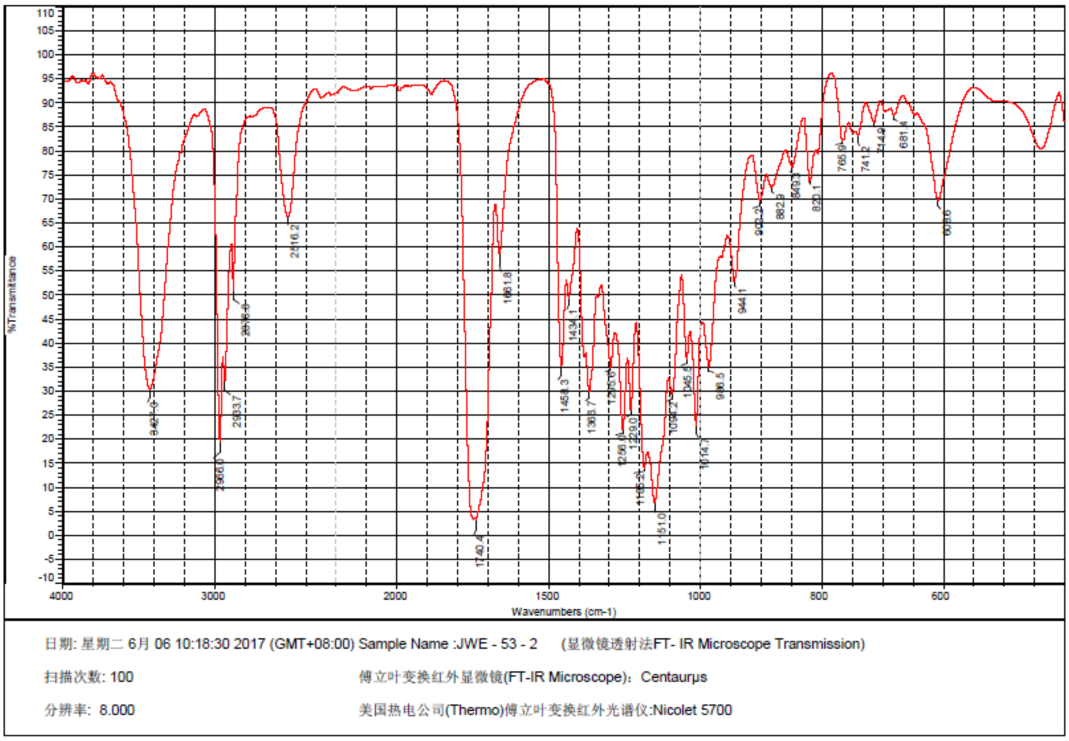

Fig. S16.8 IR spectrum (500 MHz) of cardivarolide G (**16**) in CD_3OD

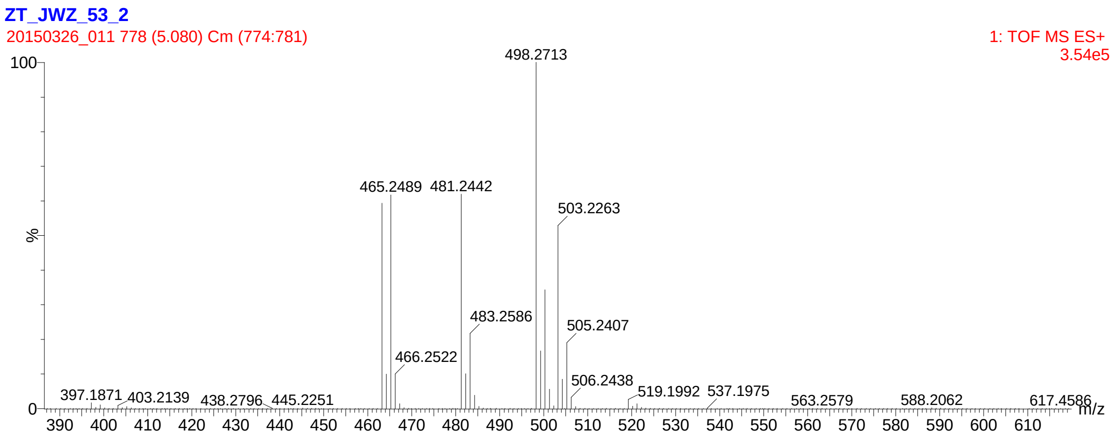

Fig. S16.9 HRESIMS spectrum of cardivarolide G (**16**)

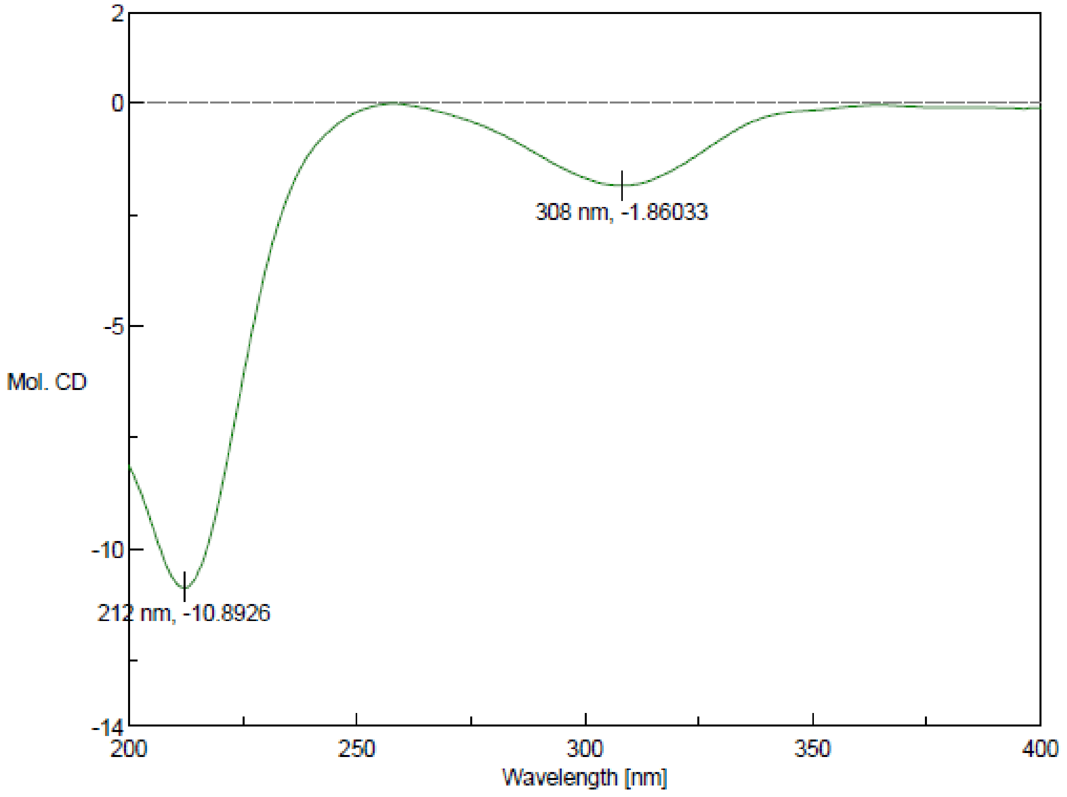

Fig. S16.10 CD spectrum of cardivarolide G (**16**)

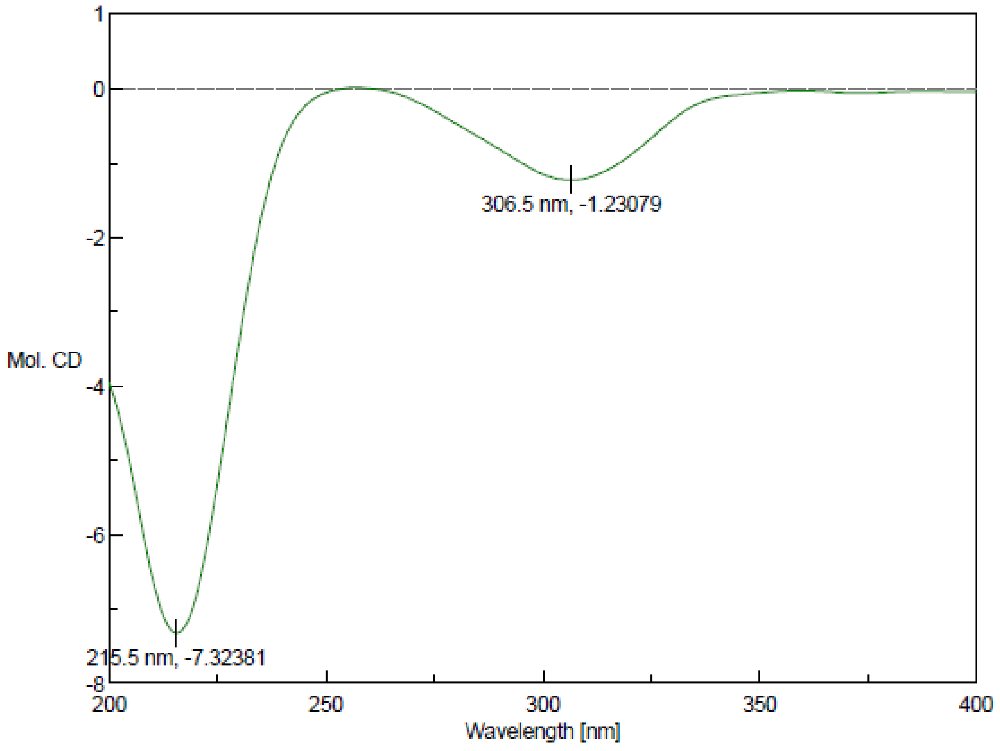

Fig. S17 CD spectrum of incaspitolide D (**17**)

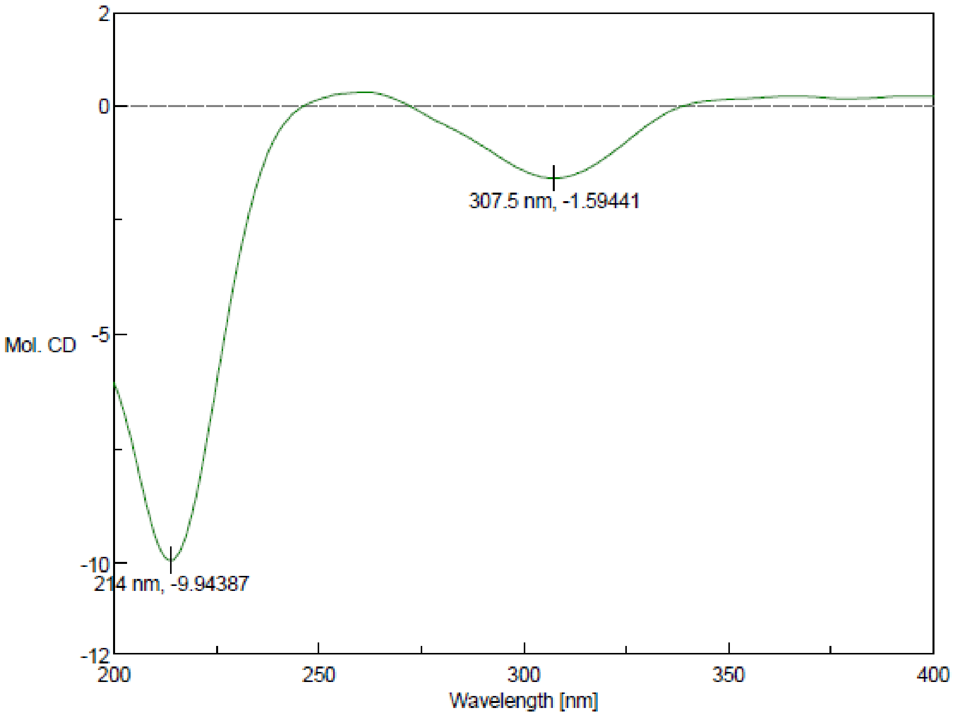

Fig. S18 CD spectrum of compound **18**

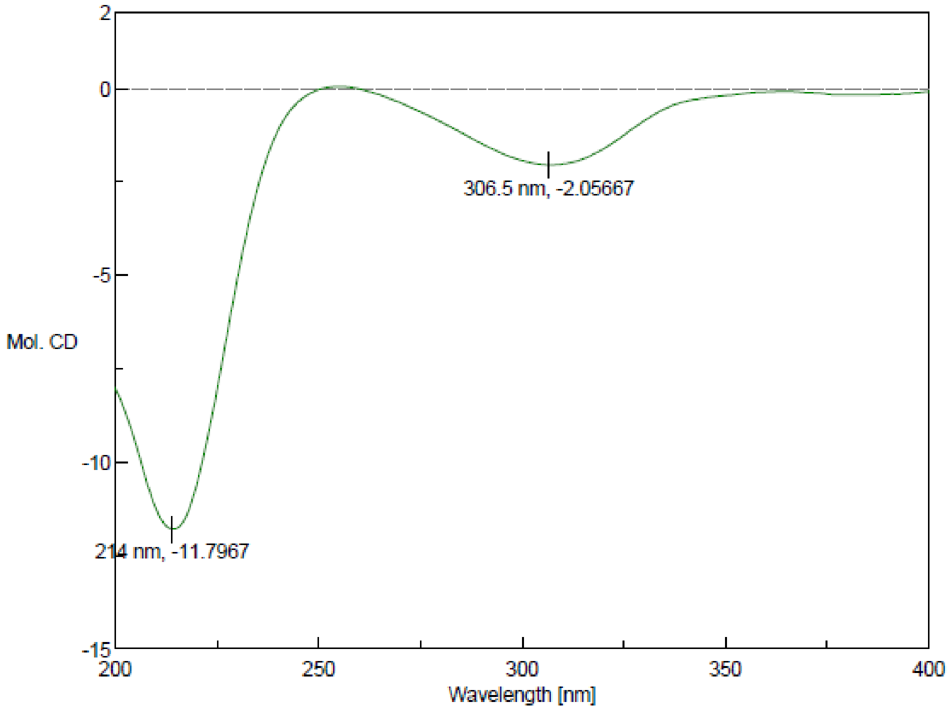

Fig. S19 CD spectrum of compound **19**

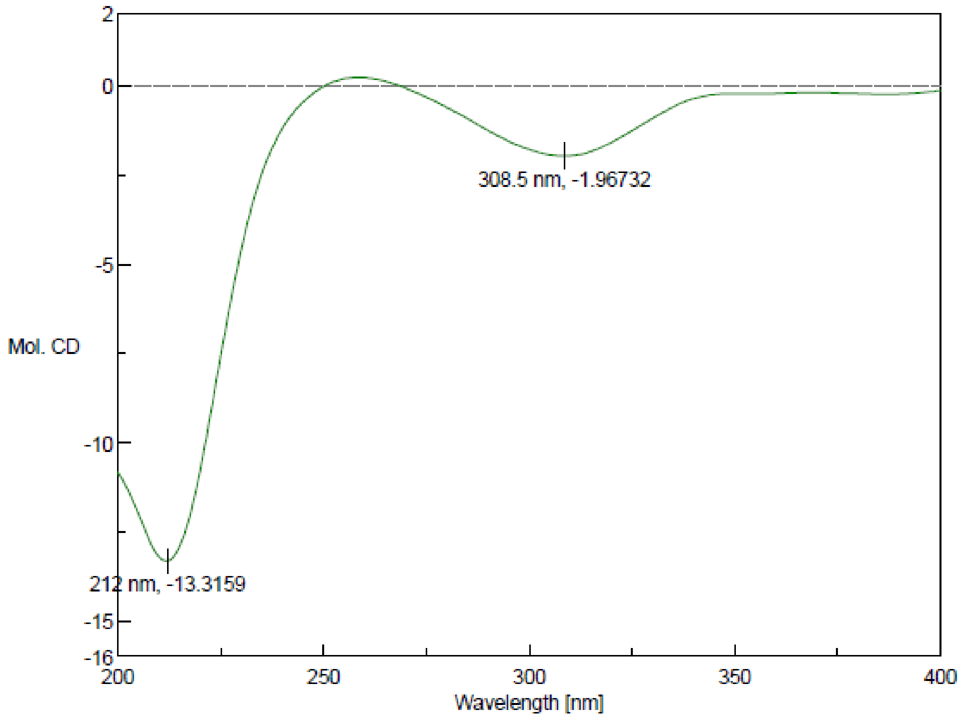

Fig. S20 CD spectrum of compound **20**

S21 X-ray data of (2*R*, 5*S*)-cardivarolide C (**4**)

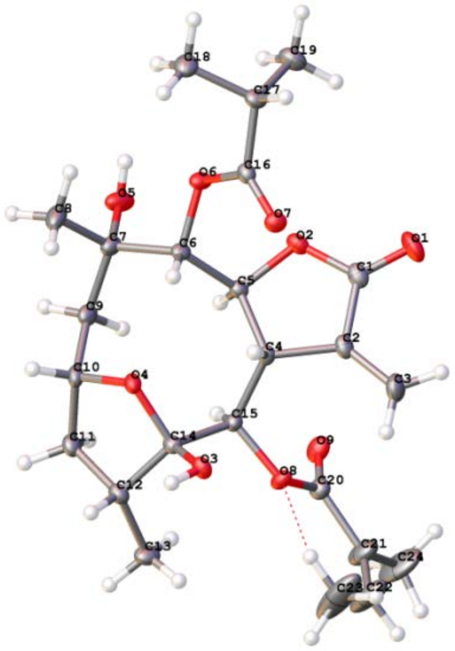

**Table 1** Crystal data and structure refinement for **4**	
Identification code	exp_2706
Empirical formula	$C_{24}H_{34}O_9$
Formula weight	466.51

Temperature / K	99.5
Crystal system	orthorhombic
Space group	$P2_12_12_1$
a / Å, b / Å, c / Å	9.05085(16), 14.4036(3), 19.1015(3)
α/°, β/°, γ/°	90.00, 90.00, 90.00
Volume / $Å^3$	2490.16(8)
Z	4
ρ_{calc} / mg mm^{-3}	1.244
μ / mm^{-1}	0.790
F(000)	1000
Crystal size / mm^3	0.58 × 0.48 × 0.45
2Θ range for data collection	7.68 to 141.96°
Index ranges	$-10 \le h \le 10$, $-17 \le k \le 17$, $-17 \le l \le 23$
Reflections collected	8581
Independent reflections	4706[R(int) = 0.0189 (inf-0.9Å)]
Data/restraints/parameters	4706/0/306
Goodness-of-fit on F^2	1.055
Final R indexes [I>2σ (I) i.e. F_o>4σ (F_o)]	R_1 = 0.0338, wR_2 = 0.0884
Final R indexes [all data]	R_1 = 0.0345, wR_2 = 0.0890
Largest diff. peak/hole / e $Å^{-3}$	0.247/-0.197
Flack Parameters	-0.10(12)
Completeness	0.988

Table 2 Fractional Atomic Coordinates (×10^4) and Equivalent Isotropic Displacement Parameters ($Å^2$×10^3) for **4**. U_{eq} is defined as 1/3 of of the trace of the orthogonalised U_{IJ} tensor.

Atom	*x*	*y*	*z*	U(eq)
O6	8723.0(12)	8054.5(7)	2162.6(5)	17.4(2)
O4	11470.4(11)	7116.9(7)	586.6(5)	15.6(2)
O1	6173.2(13)	5424.2(9)	2156.4(7)	30.2(3)
O7	7036.0(12)	7741.1(7)	1341.9(5)	17.4(2)
O2	8218.3(12)	6283.0(8)	2207.0(5)	18.5(2)
O5	11364.6(13)	7664.6(9)	2772.5(6)	27.2(3)
O3	10530.2(11)	6286.6(7)	-336.1(5)	16.7(2)
O9	10404.8(13)	3874.7(8)	1324.6(6)	23.1(2)
O8	10291.5(12)	4740.8(7)	353.9(5)	17.4(2)
C16	7388.8(16)	8139.7(10)	1875.9(8)	15.2(3)
C10	12852.0(16)	7251.8(10)	959.8(8)	18.1(3)
C17	6408.0(16)	8798.2(11)	2280.2(8)	18.5(3)
C12	13064.8(16)	6027.2(11)	98.7(8)	17.5(3)

C4	9225.9(15)	6036.9(10)	1051.4(7)	13.7(3)
C14	11427.7(16)	6229.3(9)	250.9(7)	14.2(3)
C9	12597.3(17)	7255.7(11)	1747.4(8)	19.9(3)
C11	13866.9(17)	6449.7(11)	727.1(8)	19.3(3)
C20	10246.5(17)	3929.9(11)	698.7(8)	19.7(3)
C13	13472.3(19)	5022.6(12)	-59.5(9)	23.9(3)
C6	9800.1(16)	7544(1)	1749.2(8)	15.4(3)
C18	7157.3(18)	9739.1(11)	2370.6(10)	25.2(3)
C15	10630.8(16)	5559.5(10)	754.8(7)	13.6(3)
C5	9504.9(16)	6483.4(10)	1779.8(7)	14.5(3)
C2	7871.6(16)	5450.8(11)	1176.1(8)	17.7(3)
C3	7146.7(18)	4877.6(12)	759.6(10)	26.4(4)
C1	7292.8(17)	5686.7(11)	1884.3(8)	19.7(3)
C7	11322.0(17)	7845.9(11)	2039.4(8)	19.9(3)
C21	10016(3)	3111.1(13)	236(1)	37.2(5)
C19	6002(2)	8362.8(13)	2987.8(9)	27.6(4)
C24	10137(4)	2203.4(15)	623.2(13)	61.8(8)
C8	11557.6(19)	8874.4(12)	1885.7(11)	30.0(4)
C23	9618(5)	3914.6(19)	-962.4(12)	83.4(12)
C22	9771(4)	3144.8(16)	-454.3(12)	58.4(8)

Table 3 Anisotropic Displacement Parameters ($Å^2×10^3$) for **4**.

The Anisotropic displacement factor exponent takes the form: $-2\pi^2[h^2a^{*2}U_{11}+...+2hka\times b\times U_{12}]$

Atom	U_{11}	U_{22}	U_{33}	U_{23}	U_{13}	U_{12}
O6	14.3(5)	19.7(5)	18.3(5)	-5.1(4)	-1.6(4)	4.5(4)
O4	11.8(5)	12.8(5)	22.1(5)	0.6(4)	1.3(4)	-0.1(4)
O1	20.0(6)	34.9(7)	35.7(7)	10.4(5)	10.2(5)	-2.7(5)
O7	18.1(5)	19.2(5)	14.9(5)	0.0(4)	-2.1(4)	1.8(4)
O2	17.4(5)	21.5(5)	16.5(5)	3.3(4)	3.8(4)	1.9(4)
O5	20.9(6)	37.6(7)	23.1(6)	-13.0(5)	-5.3(5)	7.9(5)
O3	15.1(5)	19.6(5)	15.4(5)	4.7(4)	1.0(4)	-0.6(4)
O9	31.1(6)	16.7(5)	21.5(6)	4.3(4)	-4.0(5)	-4.7(5)
O8	24.4(6)	13.2(5)	14.6(5)	-1.7(4)	-0.1(4)	-2.6(4)
C16	14.1(7)	14.2(6)	17.2(7)	2.9(5)	1.2(5)	0.4(5)
C10	11.5(7)	14.9(7)	27.9(8)	-0.5(6)	0.4(6)	-2.7(5)
C17	13.7(7)	21.0(7)	20.8(7)	-2.5(6)	0.4(6)	4.1(6)
C12	14.5(7)	18.7(7)	19.2(7)	2.1(6)	2.8(6)	1.8(6)
C4	13.0(6)	13.4(7)	14.6(7)	0.9(5)	-1.6(5)	-1.4(5)
C14	14.7(7)	11.6(6)	16.2(7)	1.6(5)	1.6(5)	1.5(5)

C9	11.8(7)	20.7(7)	27.3(8)	-5.7(6)	-2.7(6)	0.6(6)
C11	12.3(7)	19.2(7)	26.3(8)	-0.1(6)	0.1(6)	0.3(6)
C20	21.2(8)	15.2(7)	22.6(8)	0.0(6)	1.8(6)	-0.8(6)
C13	18.7(7)	24.9(8)	28.2(8)	-7.9(7)	-0.4(6)	7.1(7)
C6	11.7(7)	15.5(7)	18.8(7)	-3.1(5)	0.3(5)	3.3(5)
C18	19.8(8)	19.6(7)	36.4(9)	-4.7(7)	1.3(7)	4.1(6)
C15	13.8(7)	11.9(6)	15.3(6)	-0.3(5)	0.4(5)	-0.2(5)
C5	12.3(7)	16.5(7)	14.8(7)	0.0(5)	0.4(5)	1.3(5)
C2	12.2(7)	18.4(7)	22.6(7)	4.5(6)	-0.8(6)	0.1(6)
C3	16.3(8)	28.5(8)	34.5(9)	-0.2(7)	-2.5(7)	-6.0(7)
C1	15.9(7)	19.2(7)	24.0(8)	7.1(6)	0.6(6)	1.6(6)
C7	15.2(7)	19.9(7)	24.6(8)	-7.5(6)	-2.3(6)	0.4(6)
C21	58.0(13)	20.6(8)	33.0(9)	-8.1(8)	6.2(9)	-10.2(9)
C19	26.1(8)	33.3(9)	23.3(8)	0.2(7)	7.4(7)	3.1(7)
C24	114(2)	16.7(9)	54.6(14)	-8.2(9)	1.5(15)	-8.8(12)
C8	19.4(8)	19.0(8)	51.5(11)	-11.7(7)	1.2(7)	-1.4(6)
C23	167(4)	55.4(16)	27.7(11)	-3.1(11)	-17.6(17)	-50(2)
C22	108(2)	32.9(11)	34.4(11)	-13.8(9)	2.8(13)	-26.8(13)

Table 4 Bond Lengths for **4**.

**Atom**	**Atom**	**Length/Å**	**Atom**	**Atom**	**Length/Å**
O6	C16	1.3316(18)	C12	C14	1.538(2)
O6	C6	1.4542(17)	C12	C11	1.529(2)
O4	C10	1.4525(18)	C12	C13	1.524(2)
O4	C14	1.4307(17)	C4	C15	1.553(2)
O1	C1	1.200(2)	C4	C5	1.5534(19)
O7	C16	1.2133(19)	C4	C2	1.507(2)
O2	C5	1.4509(17)	C14	C15	1.5419(19)
O2	C1	1.349(2)	C9	C7	1.538(2)
O5	C7	1.4249(19)	C20	C21	1.488(2)
O3	C14	1.3871(18)	C6	C5	1.552(2)
O9	C20	1.207(2)	C6	C7	1.547(2)
O8	C20	1.3416(19)	C2	C3	1.321(2)
O8	C15	1.4392(17)	C2	C1	1.490(2)
C16	C17	1.511(2)	C7	C8	1.525(2)
C10	C9	1.522(2)	C21	C24	1.506(3)
C10	C11	1.541(2)	C21	C22	1.338(3)
C17	C18	1.525(2)	C23	C22	1.480(4)
C17	C19	1.535(2)			

Table 5 Bond Angles for **4**.

Atom	Atom	Atom	Angle/°	Atom	Atom	Atom	Angle/°
C16	O6	C6	115.55(11)	O9	C20	C21	123.56(15)
C14	O4	C10	111.27(11)	O8	C20	C21	113.74(14)
C1	O2	C5	111.59(11)	O6	C6	C5	111.22(12)
C20	O8	C15	117.30(11)	O6	C6	C7	105.08(11)
O6	C16	C17	112.36(12)	C7	C6	C5	114.61(12)
O7	C16	O6	122.73(13)	O8	C15	C4	112.47(11)
O7	C16	C17	124.89(13)	O8	C15	C14	106.28(11)
O4	C10	C9	110.81(12)	C14	C15	C4	109.49(11)
O4	C10	C11	105.74(12)	O2	C5	C4	106.92(11)
C9	C10	C11	112.21(13)	O2	C5	C6	110.81(11)
C16	C17	C18	110.75(12)	C6	C5	C4	113.69(12)
C16	C17	C19	109.52(13)	C3	C2	C4	131.21(15)
C18	C17	C19	111.71(14)	C3	C2	C1	120.98(15)
C11	C12	C14	103.51(12)	C1	C2	C4	107.56(13)
C13	C12	C14	116.78(13)	O1	C1	O2	121.81(15)
C13	C12	C11	114.76(13)	O1	C1	C2	128.20(15)
C15	C4	C5	112.15(11)	O2	C1	C2	109.99(12)
C2	C4	C15	118.40(12)	O5	C7	C9	103.58(12)
C2	C4	C5	102.86(11)	O5	C7	C6	108.95(12)
O4	C14	C12	103.17(11)	O5	C7	C8	111.31(14)
O4	C14	C15	106.98(11)	C9	C7	C6	112.51(12)
O3	C14	O4	108.97(11)	C8	C7	C9	111.22(14)
O3	C14	C12	115.01(12)	C8	C7	C6	109.17(13)
O3	C14	C15	105.54(12)	C20	C21	C24	112.71(17)
C12	C14	C15	116.76(12)	C22	C21	C20	125.43(19)
C10	C9	C7	118.30(13)	C22	C21	C24	121.83(18)
C12	C11	C10	103.99(12)	C21	C22	C23	133.5(2)
O9	C20	O8	122.69(14)				

Table 6 Torsion Angles for **4**.

A	B	C	D	Angle/°
O6	C16	C17	C18	54.46(17)
O6	C16	C17	C19	-69.17(16)
O6	C6	C5	O2	0.69(16)
O6	C6	C5	C4	121.13(12)
O6	C6	C7	O5	56.25(14)
O6	C6	C7	C9	170.52(12)

O6	C6	C7	C8	-65.49(15)
O4	C10	C9	C7	-46.88(18)
O4	C10	C11	C12	12.22(15)
O4	C14	C15	O8	166.99(10)
O4	C14	C15	C4	45.27(15)
O7	C16	C17	C18	-124.10(16)
O7	C16	C17	C19	112.26(17)
O3	C14	C15	O8	51.04(14)
O3	C14	C15	C4	-70.69(14)
O9	C20	C21	C24	-5.1(3)
O9	C20	C21	C22	176.9(2)
O8	C20	C21	C24	173.7(2)
O8	C20	C21	C22	-4.4(3)
C16	O6	C6	C5	-75.82(15)
C16	O6	C6	C7	159.62(12)
C10	O4	C14	O3	-151.58(11)
C10	O4	C14	C12	-28.93(14)
C10	O4	C14	C15	94.77(13)
C10	C9	C7	O5	177.88(13)
C10	C9	C7	C6	60.39(18)
C10	C9	C7	C8	-62.45(18)
C12	C14	C15	O8	-78.13(15)
C12	C14	C15	C4	160.15(12)
C4	C2	C1	O1	173.94(15)
C4	C2	C1	O2	-5.01(17)
C14	O4	C10	C9	-111.08(13)
C14	O4	C10	C11	10.73(15)
C14	C12	C11	C10	-28.77(15)
C9	C10	C11	C12	133.13(13)
C11	C10	C9	C7	-164.82(13)
C11	C12	C14	O4	35.21(14)
C11	C12	C14	O3	153.73(12)
C11	C12	C14	C15	-81.78(15)
C20	O8	C15	C4	-90.44(15)
C20	O8	C15	C14	149.76(12)
C20	C21	C22	C23	-1.5(6)
C13	C12	C14	O4	162.35(13)
C13	C12	C14	O3	-79.13(17)
C13	C12	C14	C15	45.36(19)
C13	C12	C11	C10	-157.16(13)

C6	O6	C16	O7	7.4(2)
C6	O6	C16	C17	-171.19(12)
C15	O8	C20	O9	3.7(2)
C15	O8	C20	C21	-175.05(14)
C15	C4	C5	O2	-138.60(11)
C15	C4	C5	C6	98.80(14)
C15	C4	C2	C3	-52.5(2)
C15	C4	C2	C1	133.45(13)
C5	O2	C1	O1	178.97(14)
C5	O2	C1	C2	-2.00(16)
C5	C4	C15	O8	145.62(12)
C5	C4	C15	C14	-96.46(14)
C5	C4	C2	C3	-176.75(17)
C5	C4	C2	C1	9.19(15)
C5	C6	C7	O5	-66.13(15)
C5	C6	C7	C9	48.13(17)
C5	C6	C7	C8	172.12(13)
C2	C4	C15	O8	26.07(17)
C2	C4	C15	C14	143.99(13)
C2	C4	C5	O2	-10.31(14)
C2	C4	C5	C6	-132.91(12)
C3	C2	C1	O1	-0.9(3)
C3	C2	C1	O2	-179.81(14)
C1	O2	C5	C4	7.98(15)
C1	O2	C5	C6	132.37(13)
C7	C6	C5	O2	119.69(13)
C7	C6	C5	C4	-119.87(13)
C24	C21	C22	C23	-179.4(4)

Table 7 Hydrogen Atom Coordinates (Å×10^4) and Isotropic Displacement Parameters (Å^2×10^3) for **4**

**Atom**	***x***	***y***	***z***	**U(eq)**
H5	10815	8028	2976	41
H3	10935	6608	-634	25
H10	13292	7844	818	22
H17	5496	8890	2013	22
H12	13340	6395	-313	21
H4	8950	6536	728	16
H9A	13503	7464	1969	24
H9B	12436	6619	1894	24

H11A	14834	6680	594	23
H11B	13982	5996	1099	23
H13A	13311	4649	350	36
H13B	14494	4989	-192	36
H13C	12868	4796	-436	36
H6	9725	7748	1261	18
H18A	8021	9669	2657	38
H18B	6484	10164	2591	38
H18C	7438	9977	1920	38
H15	11294	5396	1141	16
H5A	10365	6181	1992	17
H3A	6261	4615	907	32
H3B	7522	4737	319	32
H19A	5555	7767	2912	41
H19B	5319	8760	3229	41
H19C	6879	8289	3265	41
H24A	11037	2194	889	93
H24B	10142	1700	293	93
H24C	9310	2137	934	93
H8A	10793	9231	2108	45
H8B	11525	8975	1389	45
H8C	12502	9064	2063	45
H23A	8664	3882	-1181	125
H23B	10373	3864	-1313	125
H23C	9716	4497	-723	125
H22	9678	2561	-658	70

S22 X-ray data of (2*S*, 5*R*, 2″*R*)-ineupatolide (**10**) and (2*S*, 5*R*, 2″*S*)-ineupatolide (**11**)

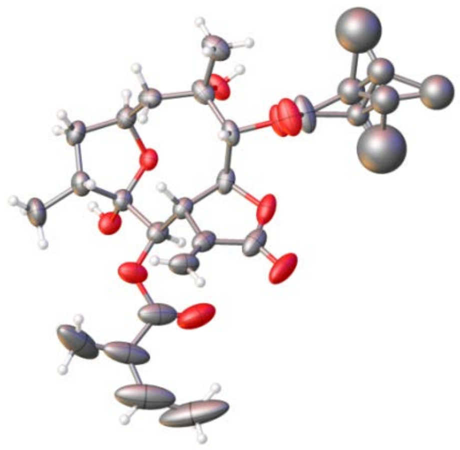

Table 1 Crystal data and structure refinement for **10** and **11**

Identification code	exp_3104
Empirical formula	$C_{25.487355}H_{27}O_{10.25}$
Formula weight	497.32
Temperature / K	103.1
Crystal system	trigonal
Space group	$P3_221$
a / Å, b / Å, c / Å	18.3947(4), 18.3947(4), 28.7027(5)
α/°, β/°, γ/°	90.00, 90.00, 120.00
Volume / $Å^3$	8410.8(3)
Z	12
ρ_{calc} / mg mm^{-3}	1.178
μ / mm^{-1}	0.774
F(000)	3143
Crystal size / mm^3	0.50 × 0.20 × 0.20
2Θ range for data collection	6.34 to 142.2°
Index ranges	$-16 \le h \le 21, -22 \le k \le 22, -35 \le l \le 34$
Reflections collected	56790
Independent reflections	10785[R(int) = 0.0359 (inf-0.9Å)]
Data/restraints/parameters	10785/0/658
Goodness-of-fit on F^2	1.027
Final R indexes [I>2σ (I) i.e. F_o>4σ (F_o)]	R_1 = 0.0668, wR_2 = 0.1781
Final R indexes [all data]	R_1 = 0.0737, wR_2 = 0.1857
Largest diff. peak/hole / e $Å^{-3}$	0.857/-0.560
Flack Parameters	0.0(2)
Completeness	0.995

Table 2 Fractional Atomic Coordinates (×10^4) and Equivalent Isotropic Displacement Parameters ($Å^2$×10^3) for **10** and **11**. U_{eq} is defined as 1/3 of of the trace of the orthogonalised U_{IJ} tensor.

**Atom**	***x***	***y***	***z***	**U(eq)**
O4	6469.9(14)	2312.6(13)	107.8(7)	34.1(4)
O3	6726.9(14)	1272.4(14)	-158.8(8)	42.8(5)
O9	8284.7(16)	2099.1(17)	294.7(10)	52.6(6)
O1	10087.5(19)	5043.3(19)	219.8(12)	76.1(10)
O5	6180.2(18)	3720.3(16)	101.1(8)	48.3(6)
C28	8982(2)	3782(2)	572.4(12)	43.8(8)
O6	7717.3(19)	5107.9(15)	281.4(8)	52.2(7)
C32	7908(2)	3923.3(19)	200.8(10)	40.2(7)

O7	8420(2)	5848.4(17)	910.7(9)	69.4(9)
C43	7817(2)	2497(2)	157.3(11)	38.0(7)
C48	6188(2)	2963(2)	786.7(10)	38.0(7)
C51	5871(2)	2200(2)	469.2(10)	36.6(6)
C57	7508(2)	4314.9(19)	506.7(10)	39.0(7)
O2	8740.0(18)	4592.5(15)	51.9(8)	54.1(7)
C64	5727(2)	1409(2)	724.0(11)	42.5(7)
C71	6884(2)	1853.3(19)	201.7(10)	35.4(6)
C72	8176(4)	5817(2)	517.9(14)	69.5(14)
C74	6544(2)	3830(2)	557.0(11)	42.3(8)
C76	6573(2)	1452(2)	685.5(11)	41.8(7)
C79	8060(2)	3281.1(19)	462.7(10)	35.4(6)
C91	6535(3)	613(2)	761.1(15)	59.2(10)
C104	9354(3)	4527(2)	274.3(13)	55(1)
C107	9418(2)	3654(3)	894.4(15)	54(1)
C117	6294(3)	4367(3)	852.2(13)	55.4(10)
O18	8622.3(14)	6561.7(14)	2515.3(7)	37.0(5)
O11	9462.9(14)	4877.1(16)	2250.5(7)	41.3(5)
O13	7476.6(14)	4921.6(15)	2823.7(7)	39.5(5)
O16	10481.4(14)	7468.0(17)	1802.8(7)	46.1(6)
O12	7905.8(15)	3831.2(15)	2327.4(7)	39.6(5)
O14	7831(2)	3299.9(18)	1615.0(8)	57.1(7)
O10	10375.1(17)	4750(2)	1791.4(9)	57.5(7)
C30	9368.1(19)	5999(2)	1881.7(9)	35.8(7)
O17	9697.5(17)	7954.8(16)	2513.9(8)	49.8(6)
C44	8812(2)	7388(2)	1840.3(11)	44.5(8)
C46	9265(2)	7220(2)	2241.4(10)	39.7(7)
O15	11478.9(17)	7371(2)	2199.4(9)	58.3(7)
C49	9976(2)	5092(2)	1871.4(11)	44.7(8)
C52	7359(2)	5530(2)	2096.9(10)	36.8(7)
C54	8047.4(19)	4620(2)	2130.2(9)	33.9(6)
C56	7800(2)	6364(2)	2360.7(11)	39.5(7)
C60	11895(3)	8275(3)	1545.0(14)	59.5(10)
C63	7949(2)	7104(2)	2053.6(12)	45.6(8)
C66	7351.3(19)	4770(2)	2331.1(10)	36.1(7)
C68	8956.9(19)	5286(2)	2244.5(9)	34.3(6)
C73	9922(2)	5754(2)	1611.7(11)	44.3(8)
C78	7830(2)	3230(2)	2030.9(12)	48.3(8)
C88	9843(2)	6870(2)	2115(1)	40.6(7)
C89	11281(2)	7666(2)	1884.8(12)	46.8(8)

C92	6500(2)	3985(2)	2256.7(12)	44.6(8)
C95	10285(2)	6019(3)	1198.1(12)	57.3(10)
C101	9256(3)	8299(3)	1665.6(15)	61.2(10)
O20	12590(2)	2590(2)	0	53.1(9)
O19	4198(2)	3006(2)	143.5(9)	66.0(8)
O8	9247(2)	2866(3)	-258.4(16)	92.2(13)
C3	9015(3)	2349(4)	37(2)	75.7(16)
C5	9427(4)	1868(5)	221(3)	101(3)
C0AA	10192(5)	2057(7)	53(3)	144(4)
C	8987(4)	1217(4)	593(4)	132(4)
C1AA	10734(6)	2713(10)	-303(3)	203(7)
O21	5832(6)	5013(4)	-158.5(13)	192(4)
C9AA	11726(6)	8418(6)	1141(3)	70.0(17)
C7AA	10967(6)	8161(6)	881(3)	71.6(17)
C8AA	12826(6)	8595(7)	1705(3)	76.1(19)
C6A	12462(7)	8010(8)	1332(4)	76.1(19)
C2A	11970(7)	9046(7)	1470(4)	70.0(17)
C2AB	11552(7)	9448(7)	1690(4)	71.6(17)
C1	7892(11)	2534(7)	2278(3)	46(4)
C4AA	6339(7)	1582(6)	2133(3)	134(4)
C2	8150(8)	6541(6)	268(3)	68(4)
C2AA	7085(7)	1641(6)	2096(3)	77(4)
C4	7636(9)	7036(9)	626(5)	183(7)
C3AA	7713(10)	1839(10)	1994(5)	116(7)
C6	8866(8)	7331(7)	413(4)	74(4)
C7	8665(9)	2485(9)	2224(4)	156(6)
C8	9142(9)	8135(9)	133(5)	107(6)
C9	9197(11)	6863(11)	-6(6)	212(9)
C11	7811(12)	1183(12)	2151(7)	104(8)
C5AA	8428(9)	7319(7)	478(4)	65(5)
C6AA	8531(8)	6582(5)	179(3)	49(4)
C15	7560(17)	2389(10)	2308(4)	71(5)

Table 3 Anisotropic Displacement Parameters ($Å^2×10^3$) for **10** and **11**. The Anisotropic displacement factor exponent takes the form: $-2\pi^2[h^2a^{*2}U_{11}+...+2hka×b×U_{12}]$

Atom	U_{11}	U_{22}	U_{33}	U_{23}	U_{13}	U_{12}
O4	40.2(11)	33.8(10)	23.6(9)	1.3(8)	0.6(8)	15.1(9)
O3	39.3(12)	37.1(12)	41.0(11)	-13.5(9)	-1.3(9)	10.9(10)
O9	42.3(13)	53.3(15)	65.5(16)	-29.0(12)	-19.0(12)	26.3(12)
O1	53.7(17)	51.9(16)	76.1(19)	-23.0(15)	25.7(15)	-8.6(14)

O5	71.2(17)	48.5(14)	36.1(12)	-11.8(10)	-24.0(11)	38.0(13)
C28	36.9(17)	40.9(18)	43.6(18)	-16.0(14)	2.4(14)	11.9(14)
O6	88.0(19)	36.6(12)	28.6(10)	-3.8(9)	-20.9(12)	28.7(13)
C32	48.3(18)	32.9(15)	22.7(13)	-2.2(11)	-2.2(12)	7.8(14)
O7	112(3)	44.5(14)	40.4(14)	-11.6(11)	-35.7(15)	30.3(16)
C43	39.2(16)	37.0(16)	31.3(14)	-9.8(12)	-1.3(12)	14.1(13)
C48	42.7(17)	49.8(18)	25.7(13)	-4.6(13)	-1.9(12)	26.2(15)
C51	36.7(16)	40.3(17)	27.3(13)	-1.0(12)	-2.3(12)	15.2(13)
C57	61(2)	30.1(15)	24.0(13)	-0.7(11)	-9.7(13)	21.9(15)
O2	62.7(17)	38.8(13)	32.7(11)	1.9(10)	10.9(11)	4.4(12)
C64	44.4(18)	38.7(17)	31.0(14)	2.9(13)	3.0(13)	10.7(14)
C71	38.5(16)	30.7(14)	31.4(14)	-5.8(11)	-3.6(12)	13.1(13)
C72	117(4)	36.6(19)	44(2)	-10.1(16)	-29(2)	30(2)
C74	63(2)	44.4(18)	26.3(14)	-9.4(13)	-12.7(14)	31.9(17)
C76	49.2(19)	36.7(16)	34.7(15)	0.6(12)	-6.0(14)	17.8(14)
C79	35.8(16)	35.1(15)	26.5(13)	-6.0(11)	-0.5(11)	11.1(13)
C91	75(3)	40.0(19)	60(2)	11.6(17)	1(2)	26.9(19)
C104	50(2)	43.6(19)	42.6(18)	-15.4(15)	10.5(16)	2.4(17)
C107	33.6(17)	55(2)	66(2)	-26.4(19)	-12.2(16)	17.3(16)
C117	75(3)	63(2)	43.1(18)	-17.8(17)	-18.6(18)	46(2)
O18	38.3(11)	46.9(12)	25.5(9)	-0.3(9)	-2.7(8)	21.1(10)
O11	41.9(12)	62.5(15)	29(1)	-3.5(10)	-2.5(9)	33.3(12)
O13	42.6(12)	51.1(13)	25.4(10)	1.4(9)	3.8(8)	23.8(11)
O16	36.8(12)	56.7(15)	29.7(10)	4.3(10)	-1.3(9)	12.0(11)
O12	49.9(13)	48.2(12)	26.5(10)	4.3(9)	-0.7(9)	29.0(11)
O14	85(2)	59.9(16)	29.5(11)	-5.0(11)	-5.7(12)	38.4(15)
O10	44.0(14)	86(2)	53.6(15)	-21.7(14)	-1.9(11)	40.7(14)
C30	30.6(14)	50.5(18)	21.6(12)	-2.2(12)	-1.2(11)	16.7(13)
O17	50.8(14)	47.9(14)	36.2(12)	-8.3(10)	-3.7(10)	13.8(11)
C44	57(2)	43.1(18)	29.4(15)	-2.0(13)	-7.0(14)	21.7(16)
C46	41.4(17)	43.1(17)	26.3(14)	0.5(12)	-2.0(12)	15.0(14)
O15	44.1(14)	75.4(19)	49.2(15)	9.9(13)	1.7(12)	25.3(13)
C49	35.8(16)	61(2)	33.8(15)	-16.7(15)	-5.2(12)	22.0(16)
C52	32.5(15)	50.3(18)	29.6(14)	-1.0(13)	-4.3(12)	22.2(14)
C54	38.2(15)	43.6(16)	18.9(12)	3.6(11)	0.5(11)	19.7(13)
C56	38.7(16)	53.0(19)	32.6(15)	-0.7(14)	-2.2(12)	27.3(15)
C60	48(2)	60(2)	48(2)	5.6(18)	1.3(17)	10.8(19)
C63	58(2)	54(2)	34.6(16)	-3.7(14)	-10.5(15)	35.0(18)
C66	35.4(15)	49.0(18)	24.5(13)	1.8(12)	-0.4(11)	21.5(14)
C68	38.0(16)	49.3(18)	21.3(12)	-2.6(12)	0.1(11)	26.0(14)

C73	35.1(16)	61(2)	28.5(14)	-9.8(14)	1.4(12)	18.0(15)
C78	58(2)	51(2)	36.8(17)	-1.2(15)	-5.1(15)	27.7(18)
C88	39.7(17)	47.5(18)	24.2(13)	-0.6(12)	-5.0(12)	13.9(15)
C89	40.5(18)	48.1(19)	41.1(17)	-3.4(15)	-1.0(14)	14.1(16)
C92	37.5(17)	51(2)	38.8(16)	1.5(14)	1.0(13)	17.2(15)
C95	47(2)	74(3)	35.8(17)	-9.2(17)	6.6(15)	18.3(19)
C101	82(3)	51(2)	50(2)	3.6(17)	-1(2)	33(2)
O20	69.0(18)	69.0(18)	33.2(16)	-6.2(7)	6.2(7)	43(2)
O19	76.2(19)	104(2)	36.8(13)	-5.8(14)	-1.1(13)	58.8(19)
O8	54(2)	115(3)	96(3)	-26(3)	15.0(19)	34(2)
C3	50(2)	86(4)	94(4)	-59(3)	-24(3)	37(3)
C5	73(3)	115(5)	146(6)	-94(5)	-59(4)	69(4)
C0AA	107(5)	241(11)	140(7)	-131(8)	-78(5)	129(7)
C	84(4)	72(4)	255(11)	-55(6)	-80(6)	51(4)
C1AA	127(7)	450.0(2)	97(5)	-99(9)	-25(5)	191(11)
O21	468(12)	215(6)	62(2)	-76(3)	-116(4)	298(8)
C9AA	64(4)	62(4)	69(4)	11(3)	9(3)	21(3)
C7AA	77(4)	62(4)	71(4)	13(3)	13(3)	31(3)
C8AA	60(4)	79(5)	67(4)	16(3)	4(3)	19(3)
C6A	60(4)	79(5)	67(4)	16(3)	4(3)	19(3)
C2A	64(4)	62(4)	69(4)	11(3)	9(3)	21(3)
C2AB	77(4)	62(4)	71(4)	13(3)	13(3)	31(3)

Table 4 Bond Lengths for **10** and **11**

Atom	Atom	Length/Å	Atom	Atom	Length/Å
O4	C51	1.451(4)	O15	C89	1.200(5)
O4	C71	1.418(4)	C49	C73	1.474(6)
O3	C71	1.410(4)	C52	C56	1.530(5)
O9	C43	1.436(4)	C52	C66	1.545(5)
O9	C3	1.396(6)	C54	C66	1.550(4)
O1	C104	1.211(5)	C54	C68	1.536(4)
O5	C74	1.437(4)	C56	C63	1.527(5)
C28	C79	1.504(4)	C60	C89	1.489(5)
C28	C104	1.463(6)	C60	C9AA	1.261(10)
C28	C107	1.321(6)	C60	C8AA	1.575(10)
O6	C57	1.460(4)	C60	C6A	1.485(13)
O6	C72	1.332(4)	C60	C2A	1.371(12)
C32	C57	1.537(5)	C66	C92	1.524(5)
C32	O2	1.468(4)	C73	C95	1.330(5)
C32	C79	1.539(5)	C78	C1	1.516(9)

O7	C72	1.204(5)	C78	C15	1.583(14)
C43	C71	1.527(4)	O8	C3	1.183(7)
C43	C79	1.550(4)	C3	C5	1.519(9)
C48	C51	1.524(4)	C5	C0AA	1.357(10)
C48	C74	1.539(5)	C5	C	1.503(12)
C51	C64	1.528(5)	C0AA	C1AA	1.513(17)
C57	C74	1.542(5)	C9AA	C7AA	1.438(14)
O2	C104	1.354(6)	C2A	C2AB	1.450(16)
C64	C76	1.522(5)	C1	C2AA	1.654(17)
C71	C76	1.542(4)	C1	C3AA	1.408(17)
C72	C2	1.534(10)	C1	C7	1.48(2)
C72	C6AA	1.559(10)	C1	C15	0.537(15)
C74	C117	1.534(5)	C4AA	C2AA	1.327(15)
C76	C91	1.525(5)	C2	C4	1.91(2)
O18	C46	1.432(4)	C2	C6	1.449(15)
O18	C56	1.439(4)	C2	C9	1.88(2)
O11	C49	1.363(4)	C2	C5AA	1.392(14)
O11	C68	1.461(4)	C2	C6AA	0.715(11)
O13	C66	1.437(3)	C2AA	C3AA	1.063(16)
O16	C88	1.449(4)	C2AA	C11	1.91(2)
O16	C89	1.348(4)	C2AA	C15	1.352(19)
O12	C54	1.455(4)	C4	C5AA	1.35(2)
O12	C78	1.346(4)	C3AA	C7	1.68(2)
O14	C78	1.201(4)	C3AA	C11	1.38(2)
O10	C49	1.203(4)	C3AA	C15	1.48(2)
C30	C68	1.544(4)	C6	C8	1.530(18)
C30	C73	1.517(5)	C6	C9	1.76(2)
C30	C88	1.542(5)	C6	C5AA	0.817(13)
O17	C46	1.412(4)	C6	C6AA	1.371(13)
C44	C46	1.541(5)	C7	C15	1.96(3)
C44	C63	1.529(5)	C8	C5AA	1.728(19)
C44	C101	1.536(5)	C9	C6AA	1.19(2)
C46	C88	1.537(5)	C5AA	C6AA	1.694(16)

Table 5 Bond Angles for **10** and **11**

Atom	Atom	Atom	Angle/°	Atom	Atom	Atom	Angle/°
C71	O4	C51	112.0(2)	O16	C89	C60	113.8(3)
C3	O9	C43	114.2(4)	O15	C89	O16	123.1(3)
C104	C28	C79	107.9(3)	O15	C89	C60	123.1(4)
C107	C28	C79	129.6(3)	O9	C3	C5	107.6(6)

C107	C28	C104	122.3(3)	O8	C3	O9	123.0(5)
C72	O6	C57	118.3(3)	O8	C3	C5	129.3(6)
C57	C32	C79	113.2(2)	C0AA	C5	C3	118.8(9)
O2	C32	C57	108.4(3)	C0AA	C5	C	122.3(7)
O2	C32	C79	105.8(3)	C	C5	C3	118.9(5)
O9	C43	C71	108.0(3)	C5	C0AA	C1AA	129.1(9)
O9	C43	C79	110.6(2)	C60	C9AA	C7AA	135.1(8)
C71	C43	C79	111.3(3)	C60	C2A	C2AB	130.9(9)
C51	C48	C74	117.9(2)	C78	C1	C2AA	106.5(8)
O4	C51	C48	112.5(3)	C3AA	C1	C78	114.5(8)
O4	C51	C64	103.6(3)	C3AA	C1	C2AA	39.6(7)
C48	C51	C64	113.2(2)	C3AA	C1	C7	71.3(10)
O6	C57	C32	105.4(2)	C7	C1	C78	118.9(12)
O6	C57	C74	105.8(3)	C7	C1	C2AA	108.6(9)
C32	C57	C74	117.7(3)	C15	C1	C78	87.1(19)
C104	O2	C32	110.7(3)	C15	C1	C2AA	47.6(17)
C76	C64	C51	103.3(3)	C15	C1	C3AA	87(2)
O4	C71	C43	104.6(2)	C15	C1	C7	151(2)
O4	C71	C76	106.0(3)	C72	C2	C4	113.7(8)
O3	C71	O4	110.2(2)	C72	C2	C9	89.8(9)
O3	C71	C43	104.6(2)	C6	C2	C72	109.3(8)
O3	C71	C76	114.3(3)	C6	C2	C4	77.7(9)
C43	C71	C76	116.8(3)	C6	C2	C9	62.0(9)
O6	C72	C2	109.5(4)	C9	C2	C4	138.5(8)
O6	C72	C6AA	109.9(4)	C5AA	C2	C72	122.5(8)
O7	C72	O6	124.2(4)	C5AA	C2	C4	44.9(8)
O7	C72	C2	124.6(5)	C5AA	C2	C6	33.3(6)
O7	C72	C6AA	124.3(5)	C5AA	C2	C9	93.8(10)
C2	C72	C6AA	26.7(4)	C6AA	C2	C72	78.6(12)
O5	C74	C48	108.5(3)	C6AA	C2	C4	147.0(14)
O5	C74	C57	108.4(3)	C6AA	C2	C6	69.3(13)
O5	C74	C117	109.0(3)	C6AA	C2	C9	11.6(11)
C48	C74	C57	114.2(3)	C6AA	C2	C5AA	102.2(14)
C117	C74	C48	108.7(3)	C1	C2AA	C11	86.2(9)
C117	C74	C57	107.9(3)	C4AA	C2AA	C1	115.6(10)
C64	C76	C71	102.4(3)	C4AA	C2AA	C11	151.9(11)
C64	C76	C91	114.2(3)	C4AA	C2AA	C15	99.3(13)
C91	C76	C71	115.5(3)	C3AA	C2AA	C1	57.6(11)
C28	C79	C32	102.4(3)	C3AA	C2AA	C4AA	162.1(14)
C28	C79	C43	113.6(3)	C3AA	C2AA	C11	45.2(10)

C32	C79	C43	111.0(2)	C3AA	C2AA	C15	74.7(15)
O1	C104	C28	128.8(5)	C15	C2AA	C1	17.0(8)
O1	C104	O2	121.4(4)	C15	C2AA	C11	100.0(12)
O2	C104	C28	109.8(3)	C5AA	C4	C2	46.9(7)
C46	O18	C56	111.3(2)	C1	C3AA	C7	56.2(10)
C49	O11	C68	111.7(3)	C1	C3AA	C15	21.2(6)
C89	O16	C88	117.2(3)	C2AA	C3AA	C1	82.8(14)
C78	O12	C54	117.8(2)	C2AA	C3AA	C7	134.7(14)
C73	C30	C68	102.1(3)	C2AA	C3AA	C11	101.8(15)
C73	C30	C88	114.5(3)	C2AA	C3AA	C15	61.6(14)
C88	C30	C68	111.8(2)	C11	C3AA	C1	122.1(13)
C63	C44	C46	101.9(3)	C11	C3AA	C7	86.8(12)
C63	C44	C101	113.5(3)	C11	C3AA	C15	123.4(14)
C101	C44	C46	114.6(3)	C15	C3AA	C7	76.4(13)
O18	C46	C44	106.2(3)	C2	C6	C8	121.0(10)
O18	C46	C88	103.1(3)	C2	C6	C9	71.2(9)
O17	C46	O18	109.7(2)	C8	C6	C9	94.9(10)
O17	C46	C44	108.9(3)	C5AA	C6	C2	69.5(13)
O17	C46	C88	110.8(3)	C5AA	C6	C8	89.5(14)
C88	C46	C44	117.7(3)	C5AA	C6	C9	136.4(15)
O11	C49	C73	108.6(3)	C5AA	C6	C6AA	98.3(14)
O10	C49	O11	121.1(4)	C6AA	C6	C2	29.2(5)
O10	C49	C73	130.3(3)	C6AA	C6	C8	118.7(10)
C56	C52	C66	116.9(2)	C6AA	C6	C9	42.6(8)
O12	C54	C66	106.3(2)	C1	C7	C3AA	52.4(8)
O12	C54	C68	107.0(2)	C1	C7	C15	7.6(6)
C68	C54	C66	116.5(3)	C3AA	C7	C15	47.2(7)
O18	C56	C52	111.7(3)	C6	C8	C5AA	28.2(5)
O18	C56	C63	104.8(3)	C6	C9	C2	46.8(7)
C63	C56	C52	112.1(3)	C6AA	C9	C2	6.9(6)
C89	C60	C8AA	111.4(5)	C6AA	C9	C6	51.2(9)
C9AA	C60	C89	125.8(5)	C3AA	C11	C2AA	33.0(8)
C9AA	C60	C8AA	121.7(6)	C2	C5AA	C8	112.0(10)
C9AA	C60	C6A	88.7(7)	C2	C5AA	C6AA	24.3(5)
C9AA	C60	C2A	63.2(7)	C4	C5AA	C2	88.2(11)
C6A	C60	C89	114.5(6)	C4	C5AA	C8	136.3(12)
C6A	C60	C8AA	55.4(6)	C4	C5AA	C6AA	112.5(10)
C2A	C60	C89	121.8(6)	C6	C5AA	C2	77.2(14)
C2A	C60	C8AA	97.6(7)	C6	C5AA	C4	160.7(17)
C2A	C60	C6A	123.5(7)	C6	C5AA	C8	62.3(12)

C56	C63	C44	103.3(3)	C6	C5AA	C6AA	53.2(11)
O13	C66	C52	108.9(3)	C6AA	C5AA	C8	93.7(9)
O13	C66	C54	109.5(2)	C72	C6AA	C5AA	104.0(7)
O13	C66	C92	108.1(2)	C2	C6AA	C72	74.7(11)
C52	C66	C54	111.4(2)	C2	C6AA	C6	81.5(13)
C92	C66	C52	109.7(3)	C2	C6AA	C9	161.5(17)
C92	C66	C54	109.1(3)	C2	C6AA	C5AA	53.4(11)
O11	C68	C30	106.1(2)	C6	C6AA	C72	112.1(7)
O11	C68	C54	108.2(3)	C6	C6AA	C5AA	28.5(6)
C54	C68	C30	114.1(2)	C9	C6AA	C72	123.1(12)
C49	C73	C30	108.7(3)	C9	C6AA	C6	86.2(11)
C95	C73	C30	129.5(4)	C9	C6AA	C5AA	112.4(11)
C95	C73	C49	121.7(4)	C78	C15	C7	92.6(13)
O12	C78	C1	112.2(4)	C1	C15	C78	73.1(19)
O12	C78	C15	109.8(5)	C1	C15	C2AA	115(2)
O14	C78	O12	123.2(3)	C1	C15	C3AA	72(2)
O14	C78	C1	123.9(5)	C1	C15	C7	21.3(16)
O14	C78	C15	125.9(5)	C2AA	C15	C78	119.8(9)
C1	C78	C15	19.8(5)	C2AA	C15	C3AA	43.7(7)
O16	C88	C30	110.1(2)	C2AA	C15	C7	98.6(11)
O16	C88	C46	107.2(3)	C3AA	C15	C78	106.7(12)
C46	C88	C30	112.8(3)	C3AA	C15	C7	56.4(11)

Table 6 Torsion Angles for **10** and **11**

**A**	**B**	**C**	**D**	**Angle/˚**
O4	C51	C64	C76	32.5(3)
O4	C71	C76	C64	24.5(3)
O4	C71	C76	C91	149.2(3)
O3	C71	C76	C64	-97.0(3)
O3	C71	C76	C91	27.7(4)
O9	C43	C71	O4	174.2(2)
O9	C43	C71	O3	-70.0(3)
O9	C43	C71	C76	57.4(3)
O9	C43	C79	C28	39.1(4)
O9	C43	C79	C32	153.8(3)
O9	C3	C5	C0AA	-174.2(4)
O9	C3	C5	C	4.1(6)
O6	C57	C74	O5	58.6(3)
O6	C57	C74	C48	179.6(2)
O6	C57	C74	C117	-59.4(3)

O6	C72	C2	C4	116.5(7)
O6	C72	C2	C6	-158.9(9)
O6	C72	C2	C9	-98.8(7)
O6	C72	C2	C5AA	166.9(12)
O6	C72	C2	C6AA	-95.8(11)
O6	C72	C6AA	C2	94.0(11)
O6	C72	C6AA	C6	167.9(9)
O6	C72	C6AA	C9	-91.7(12)
O6	C72	C6AA	C5AA	139.1(7)
C32	C57	C74	O5	-58.8(3)
C32	C57	C74	C48	62.2(3)
C32	C57	C74	C117	-176.8(3)
C32	O2	C104	O1	-176.2(3)
C32	O2	C104	C28	3.0(4)
O7	C72	C2	C4	-49.1(12)
O7	C72	C2	C6	35.5(14)
O7	C72	C2	C9	95.6(9)
O7	C72	C2	C5AA	1.3(17)
O7	C72	C2	C6AA	98.6(12)
O7	C72	C6AA	C2	-100.1(12)
O7	C72	C6AA	C6	-26.2(14)
O7	C72	C6AA	C9	74.2(14)
O7	C72	C6AA	C5AA	-54.9(11)
C43	O9	C3	O8	2.6(6)
C43	O9	C3	C5	-179.5(3)
C43	C71	C76	C64	140.5(3)
C43	C71	C76	C91	-94.8(4)
C48	C51	C64	C76	-89.6(3)
C51	O4	C71	O3	119.9(3)
C51	O4	C71	C43	-128.2(2)
C51	O4	C71	C76	-4.2(3)
C51	C48	C74	O5	25.0(4)
C51	C48	C74	C57	-96.1(3)
C51	C48	C74	C117	143.4(3)
C51	C64	C76	C71	-34.6(3)
C51	C64	C76	C91	-160.2(3)
C57	O6	C72	O7	-1.4(8)
C57	O6	C72	C2	-167.0(6)
C57	O6	C72	C6AA	164.6(6)
C57	C32	O2	C104	108.2(3)

C57	C32	C79	C28	-100.7(3)
C57	C32	C79	C43	137.7(3)
O2	C32	C57	O6	40.6(3)
O2	C32	C57	C74	158.2(3)
O2	C32	C79	C28	17.9(3)
O2	C32	C79	C43	-103.7(3)
C71	O4	C51	C48	104.8(3)
C71	O4	C51	C64	-17.8(3)
C71	C43	C79	C28	159.2(3)
C71	C43	C79	C32	-86.1(3)
C72	O6	C57	C32	-118.6(4)
C72	O6	C57	C74	116.1(4)
C72	C2	C4	C5AA	113.0(10)
C72	C2	C6	C8	163.4(11)
C72	C2	C6	C9	79.1(10)
C72	C2	C6	C5AA	-120.2(13)
C72	C2	C6	C6AA	69.2(12)
C72	C2	C9	C6	-112.1(8)
C72	C2	C9	C6AA	15(5)
C72	C2	C5AA	C4	-92.0(14)
C72	C2	C5AA	C6	75.2(16)
C72	C2	C5AA	C8	127.9(12)
C72	C2	C5AA	C6AA	84.2(16)
C72	C2	C6AA	C6	-115.9(6)
C72	C2	C6AA	C9	-165(5)
C72	C2	C6AA	C5AA	-121.1(8)
C74	C48	C51	O4	49.3(4)
C74	C48	C51	C64	166.4(3)
C79	C28	C104	O1	-171.7(4)
C79	C28	C104	O2	9.2(4)
C79	C32	C57	O6	157.7(2)
C79	C32	C57	C74	-84.7(3)
C79	C32	O2	C104	-13.6(3)
C79	C43	C71	O4	52.6(3)
C79	C43	C71	O3	168.4(3)
C79	C43	C71	C76	-64.2(4)
C104	C28	C79	C32	-16.5(3)
C104	C28	C79	C43	103.3(3)
C107	C28	C79	C32	158.9(4)
C107	C28	C79	C43	-81.3(4)

C107	C28	C104	O1	12.4(6)
C107	C28	C104	O2	-166.6(3)
O18	C46	C88	O16	175.3(2)
O18	C46	C88	C30	54.1(3)
O18	C56	C63	C44	31.5(3)
O11	C49	C73	C30	4.3(4)
O11	C49	C73	C95	-172.4(3)
O12	C54	C66	O13	67.1(3)
O12	C54	C66	C52	-172.3(2)
O12	C54	C66	C92	-51.1(3)
O12	C54	C68	O11	32.3(3)
O12	C54	C68	C30	150.2(2)
O12	C78	C1	C2AA	130.9(6)
O12	C78	C1	C3AA	172.4(11)
O12	C78	C1	C7	-106.2(8)
O12	C78	C1	C15	86.8(18)
O12	C78	C15	C1	-100.8(18)
O12	C78	C15	C2AA	149.3(15)
O12	C78	C15	C3AA	-164.9(11)
O12	C78	C15	C7	-109.3(6)
O14	C78	C1	C2AA	-58.8(12)
O14	C78	C1	C3AA	-17.3(18)
O14	C78	C1	C7	64.1(12)
O14	C78	C1	C15	-102.9(18)
O14	C78	C15	C1	91.0(19)
O14	C78	C15	C2AA	-19(3)
O14	C78	C15	C3AA	27(2)
O14	C78	C15	C7	82.5(11)
O10	C49	C73	C30	-176.5(3)
O10	C49	C73	C95	6.8(6)
O17	C46	C88	O16	-67.4(3)
O17	C46	C88	C30	171.4(2)
C44	C46	C88	O16	58.9(3)
C44	C46	C88	C30	-62.4(4)
C46	O18	C56	C52	106.2(3)
C46	O18	C56	C63	-15.4(3)
C46	C44	C63	C56	-34.7(3)
C49	O11	C68	C30	-15.4(3)
C49	O11	C68	C54	107.5(3)
C52	C56	C63	C44	-89.8(3)

C54	O12	C78	O14	-3.9(5)
C54	O12	C78	C1	166.5(8)
C54	O12	C78	C15	-172.5(10)
C56	O18	C46	O17	110.6(3)
C56	O18	C46	C44	-7.0(3)
C56	O18	C46	C88	-131.4(2)
C56	C52	C66	O13	22.1(4)
C56	C52	C66	C54	-98.8(3)
C56	C52	C66	C92	140.3(3)
C63	C44	C46	O18	26.2(3)
C63	C44	C46	O17	-91.9(3)
C63	C44	C46	C88	140.9(3)
C66	C52	C56	O18	49.3(4)
C66	C52	C56	C63	166.6(3)
C66	C54	C68	O11	151.1(2)
C66	C54	C68	C30	-91.1(3)
C68	O11	C49	O10	-172.2(3)
C68	O11	C49	C73	7.1(3)
C68	C30	C73	C49	-12.8(3)
C68	C30	C73	C95	163.6(4)
C68	C30	C88	O16	156.8(3)
C68	C30	C88	C46	-83.6(3)
C68	C54	C66	O13	-52.0(3)
C68	C54	C66	C52	68.5(3)
C68	C54	C66	C92	-170.2(2)
C73	C30	C68	O11	16.5(3)
C73	C30	C68	C54	-102.6(3)
C73	C30	C88	O16	41.3(4)
C73	C30	C88	C46	160.9(3)
C78	O12	C54	C66	125.7(3)
C78	O12	C54	C68	-109.2(3)
C78	C1	C2AA	C4AA	-52.3(12)
C78	C1	C2AA	C3AA	108.7(13)
C78	C1	C2AA	C11	146.1(9)
C78	C1	C2AA	C15	-70(2)
C78	C1	C3AA	C2AA	-86.5(15)
C78	C1	C3AA	C7	114.0(14)
C78	C1	C3AA	C11	174.2(15)
C78	C1	C3AA	C15	-86(2)
C78	C1	C7	C3AA	-108.2(10)

C78	C1	C7	C15	-152(5)
C78	C1	C15	C2AA	115.5(14)
C78	C1	C15	C3AA	114.7(8)
C78	C1	C15	C7	156(4)
C88	O16	C89	O15	0.1(5)
C88	O16	C89	C60	179.3(3)
C88	C30	C68	O11	-106.4(3)
C88	C30	C68	C54	134.6(3)
C88	C30	C73	C49	108.2(3)
C88	C30	C73	C95	-75.4(4)
C89	O16	C88	C30	-102.0(3)
C89	O16	C88	C46	135.0(3)
C89	C60	C9AA	C7AA	6.8(16)
C89	C60	C2A	C2AB	3.0(15)
C101	C44	C46	O18	149.2(3)
C101	C44	C46	O17	31.1(4)
C101	C44	C46	C88	-96.0(4)
C101	C44	C63	C56	-158.5(3)
O8	C3	C5	C0AA	3.5(8)
O8	C3	C5	C	-178.2(6)
C3	O9	C43	C71	143.4(3)
C3	O9	C43	C79	-94.5(3)
C3	C5	C0AA	C1AA	1.0(10)
C	C5	C0AA	C1AA	-177.2(7)
C9AA	C60	C89	O16	-21.5(8)
C9AA	C60	C89	O15	157.6(7)
C9AA	C60	C2A	C2AB	120.3(14)
C8AA	C60	C89	O16	170.6(5)
C8AA	C60	C89	O15	-10.2(7)
C8AA	C60	C9AA	C7AA	173.5(11)
C8AA	C60	C2A	C2AB	-117.9(12)
C6A	C60	C89	O16	-128.9(6)
C6A	C60	C89	O15	50.3(7)
C6A	C60	C9AA	C7AA	126.4(13)
C6A	C60	C2A	C2AB	-171.1(11)
C2A	C60	C89	O16	56.5(7)
C2A	C60	C89	O15	-124.3(7)
C2A	C60	C9AA	C7AA	-104.5(14)
C1	C78	C15	C2AA	-110(3)
C1	C78	C15	C3AA	-64(2)

C1	C78	C15	C7	-8.6(15)
C1	C2AA	C3AA	C7	24.1(14)
C1	C2AA	C3AA	C11	121.4(14)
C1	C2AA	C3AA	C15	-0.4(8)
C1	C2AA	C11	C3AA	-46.3(14)
C1	C2AA	C15	C78	84(3)
C1	C2AA	C15	C3AA	1(2)
C1	C2AA	C15	C7	-13.7(19)
C1	C3AA	C7	C15	7.1(8)
C1	C3AA	C11	C2AA	88.8(19)
C1	C3AA	C15	C78	65(2)
C1	C3AA	C15	C2AA	-179(2)
C1	C3AA	C15	C7	-16.6(18)
C1	C7	C15	C78	23(4)
C1	C7	C15	C2AA	144(5)
C1	C7	C15	C3AA	132(5)
C4AA	C2AA	C3AA	C1	73(5)
C4AA	C2AA	C3AA	C7	97(5)
C4AA	C2AA	C3AA	C11	-166(4)
C4AA	C2AA	C3AA	C15	72(4)
C4AA	C2AA	C11	C3AA	171(3)
C4AA	C2AA	C15	C78	-79.6(19)
C4AA	C2AA	C15	C1	-164(2)
C4AA	C2AA	C15	C3AA	-162.8(14)
C4AA	C2AA	C15	C7	-177.6(8)
C2	C72	C6AA	C6	73.9(14)
C2	C72	C6AA	C9	174.3(19)
C2	C72	C6AA	C5AA	45.1(11)
C2	C4	C5AA	C6	-41(5)
C2	C4	C5AA	C8	120.6(16)
C2	C4	C5AA	C6AA	-1.7(6)
C2	C6	C8	C5AA	65.6(15)
C2	C6	C9	C6AA	-7.1(8)
C2	C6	C5AA	C4	42(5)
C2	C6	C5AA	C8	-123.6(9)
C2	C6	C5AA	C6AA	-4.6(8)
C2	C6	C6AA	C72	-69.6(12)
C2	C6	C6AA	C9	166.1(16)
C2	C6	C6AA	C5AA	8.9(16)
C2	C9	C6AA	C72	-162(6)

C2	C9	C6AA	C6	-48(5)
C2	C9	C6AA	C5AA	-37(5)
C2	C5AA	C6AA	C72	-58.3(12)
C2	C5AA	C6AA	C6	-169.1(19)
C2	C5AA	C6AA	C9	166.2(19)
C2AA	C1	C3AA	C7	-159.5(13)
C2AA	C1	C3AA	C11	-99(2)
C2AA	C1	C3AA	C15	1(2)
C2AA	C1	C7	C3AA	13.6(8)
C2AA	C1	C7	C15	-30(4)
C2AA	C1	C15	C78	-115.5(14)
C2AA	C1	C15	C3AA	-0.8(17)
C2AA	C1	C15	C7	40(5)
C2AA	C3AA	C7	C1	-29.2(18)
C2AA	C3AA	C7	C15	-22.0(17)
C2AA	C3AA	C15	C78	-115.9(15)
C2AA	C3AA	C15	C1	179(2)
C2AA	C3AA	C15	C7	162.3(13)
C4	C2	C6	C8	-85.6(13)
C4	C2	C6	C9	-169.9(8)
C4	C2	C6	C5AA	-9.2(11)
C4	C2	C6	C6AA	-179.8(13)
C4	C2	C9	C6	15.0(12)
C4	C2	C9	C6AA	142(5)
C4	C2	C5AA	C6	167.2(16)
C4	C2	C5AA	C8	-140.1(13)
C4	C2	C5AA	C6AA	176.2(14)
C4	C2	C6AA	C72	116(2)
C4	C2	C6AA	C6	0(2)
C4	C2	C6AA	C9	-49(6)
C4	C2	C6AA	C5AA	-4.9(18)
C4	C5AA	C6AA	C72	-54.3(11)
C4	C5AA	C6AA	C2	4.1(15)
C4	C5AA	C6AA	C6	-165.0(18)
C4	C5AA	C6AA	C9	170.3(13)
C3AA	C1	C2AA	C4AA	-161.0(15)
C3AA	C1	C2AA	C11	37.4(11)
C3AA	C1	C2AA	C15	-179(3)
C3AA	C1	C7	C15	-44(4)
C3AA	C1	C15	C78	-114.7(8)

C3AA	C1	C15	C2AA	0.8(17)
C3AA	C1	C15	C7	41(4)
C3AA	C2AA	C15	C78	83.2(19)
C3AA	C2AA	C15	C1	-1(2)
C3AA	C2AA	C15	C7	-14.8(11)
C3AA	C7	C15	C78	-108.4(10)
C3AA	C7	C15	C1	-132(5)
C3AA	C7	C15	C2AA	12.3(9)
C6	C2	C4	C5AA	7.1(9)
C6	C2	C9	C6AA	127(5)
C6	C2	C5AA	C4	-167.2(16)
C6	C2	C5AA	C8	52.7(12)
C6	C2	C5AA	C6AA	9.0(16)
C6	C2	C6AA	C72	115.9(6)
C6	C2	C6AA	C9	-49(5)
C6	C2	C6AA	C5AA	-5.3(9)
C6	C8	C5AA	C2	-61.2(14)
C6	C8	C5AA	C4	-173(2)
C6	C8	C5AA	C6AA	-44.6(11)
C6	C9	C6AA	C72	-114.1(11)
C6	C9	C6AA	C2	48(5)
C6	C9	C6AA	C5AA	11.5(7)
C6	C5AA	C6AA	C72	110.7(13)
C6	C5AA	C6AA	C2	169.1(19)
C6	C5AA	C6AA	C9	-24.7(16)
C7	C1	C2AA	C4AA	178.5(10)
C7	C1	C2AA	C3AA	-20.5(13)
C7	C1	C2AA	C11	16.9(10)
C7	C1	C2AA	C15	161(3)
C7	C1	C3AA	C2AA	159.5(13)
C7	C1	C3AA	C11	60.1(17)
C7	C1	C3AA	C15	160(2)
C7	C1	C15	C78	-156(4)
C7	C1	C15	C2AA	-40(5)
C7	C1	C15	C3AA	-41(4)
C7	C3AA	C11	C2AA	135.0(15)
C7	C3AA	C15	C78	81.8(14)
C7	C3AA	C15	C1	16.6(18)
C7	C3AA	C15	C2AA	-162.3(13)
C8	C6	C9	C2	-121.1(10)

C8	C6	C9	C6AA	-128.2(12)
C8	C6	C5AA	C2	123.6(9)
C8	C6	C5AA	C4	165(5)
C8	C6	C5AA	C6AA	119.0(10)
C8	C6	C6AA	C72	-172.5(11)
C8	C6	C6AA	C2	-102.9(16)
C8	C6	C6AA	C9	63.2(15)
C8	C6	C6AA	C5AA	-94.1(17)
C8	C5AA	C6AA	C72	161.6(8)
C8	C5AA	C6AA	C2	-140.1(14)
C8	C5AA	C6AA	C6	50.9(13)
C8	C5AA	C6AA	C9	26.2(13)
C9	C2	C4	C5AA	-6.4(14)
C9	C2	C6	C8	84.3(13)
C9	C2	C6	C5AA	160.7(14)
C9	C2	C6	C6AA	-9.9(12)
C9	C2	C5AA	C4	175.8(9)
C9	C2	C5AA	C6	-17.0(12)
C9	C2	C5AA	C8	35.7(11)
C9	C2	C5AA	C6AA	-8.0(11)
C9	C2	C6AA	C72	165(5)
C9	C2	C6AA	C6	49(5)
C9	C2	C6AA	C5AA	44(5)
C9	C6	C8	C5AA	136.5(15)
C9	C6	C5AA	C2	26.9(19)
C9	C6	C5AA	C4	69(5)
C9	C6	C5AA	C8	-96.7(19)
C9	C6	C5AA	C6AA	22.3(14)
C9	C6	C6AA	C72	124.4(13)
C9	C6	C6AA	C2	-166.1(16)
C9	C6	C6AA	C5AA	-157.2(15)
C11	C2AA	C3AA	C1	-121.4(14)
C11	C2AA	C3AA	C7	-97(2)
C11	C2AA	C3AA	C15	-121.7(13)
C11	C2AA	C15	C78	121.0(17)
C11	C2AA	C15	C1	37(2)
C11	C2AA	C15	C3AA	37.8(11)
C11	C2AA	C15	C7	23.0(10)
C11	C3AA	C7	C1	-132.6(13)
C11	C3AA	C7	C15	-125.5(14)

C11	C3AA	C15	C78	158.7(16)
C11	C3AA	C15	C1	93(2)
C11	C3AA	C15	C2AA	-85.5(19)
C11	C3AA	C15	C7	76.8(18)
C5AA	C2	C6	C8	-76.4(16)
C5AA	C2	C6	C9	-160.7(14)
C5AA	C2	C6	C6AA	-170.6(17)
C5AA	C2	C9	C6	10.5(7)
C5AA	C2	C9	C6AA	137(5)
C5AA	C2	C6AA	C72	121.1(8)
C5AA	C2	C6AA	C6	5.3(9)
C5AA	C2	C6AA	C9	-44(5)
C5AA	C6	C9	C2	-26.6(19)
C5AA	C6	C9	C6AA	-34(2)
C5AA	C6	C6AA	C72	-78.4(15)
C5AA	C6	C6AA	C2	-8.9(16)
C5AA	C6	C6AA	C9	157.2(15)
C6AA	C72	C2	C4	-147.8(15)
C6AA	C72	C2	C6	-63.1(13)
C6AA	C72	C2	C9	-3.0(10)
C6AA	C72	C2	C5AA	-97.4(17)
C6AA	C2	C4	C5AA	7(2)
C6AA	C2	C6	C8	94.2(16)
C6AA	C2	C6	C9	9.9(12)
C6AA	C2	C6	C5AA	170.6(17)
C6AA	C2	C9	C6	-127(5)
C6AA	C2	C5AA	C4	-176.2(14)
C6AA	C2	C5AA	C6	-9.0(16)
C6AA	C2	C5AA	C8	43.7(16)
C6AA	C6	C8	C5AA	99.2(17)
C6AA	C6	C9	C2	7.1(8)
C6AA	C6	C5AA	C2	4.6(8)
C6AA	C6	C5AA	C4	46(5)
C6AA	C6	C5AA	C8	-119.0(10)
C15	C78	C1	C2AA	44.0(17)
C15	C78	C1	C3AA	86(2)
C15	C78	C1	C7	167(2)
C15	C1	C2AA	C4AA	18(2)
C15	C1	C2AA	C3AA	179(3)
C15	C1	C2AA	C11	-144(2)

C15	C1	C3AA	C2AA	-1(2)
C15	C1	C3AA	C7	-160(2)
C15	C1	C3AA	C11	-100(2)
C15	C1	C7	C3AA	44(4)
C15	C2AA	C3AA	C1	0.4(8)
C15	C2AA	C3AA	C7	24.5(18)
C15	C2AA	C3AA	C11	121.7(13)
C15	C2AA	C11	C3AA	-56.4(16)
C15	C3AA	C7	C1	-7.1(8)
C15	C3AA	C11	C2AA	63.6(19)

Table 7 Hydrogen Atom Coordinates (Å×10^4) and Isotropic Displacement Parameters (Å^2×10^3) for **10** and **11**

**Atom**	***x***	***y***	***z***	**U(eq)**
H3	6225	923	-165	64
H5	6133	4129	36	72
H32	7555	3658	-73	48
H43	7943	2672	-169	46
H48A	6622	2977	985	46
H48B	5728	2878	988	46
H51	5344	2091	324	44
H57	7764	4430	817	47
H64A	5577	1416	1047	51
H64B	5288	908	575	51
H76	6948	1847	921	50
H79	7734	3113	752	42
H91A	6368	430	1076	89
H91B	7079	677	705	89
H91C	6135	203	550	89
H10A	9983	4045	938	65
H10B	9161	3173	1078	65
H11A	6488	4900	703	83
H11B	6544	4452	1155	83
H11C	5694	4083	882	83
H13	7692	5426	2874	59
H30	8935	5978	1674	43
H17	10112	8305	2372	75
H44	8746	7017	1579	53
H52A	7625	5615	1794	44
H52B	6781	5387	2043	44

H54	7983	4561	1791	41
H56	7459	6334	2631	47
H63A	7953	7548	2237	55
H63B	7522	6930	1814	55
H68	8977	5527	2552	41
H88	10114	6827	2399	49
H92A	6495	3516	2406	67
H92B	6401	3876	1929	67
H92C	6068	4069	2388	67
H95A	10583	5785	1065	69
H95B	10242	6438	1041	69
H10C	9260	8660	1908	92
H10D	8963	8341	1399	92
H10E	9823	8466	1580	92
H0A	10413	1739	173	173
HA	8905	1476	863	198
HB	9321	970	675	198
HC	8452	789	478	198
H1AA	10617	2464	-608	304
H1AB	11315	2926	-229	304
H1AC	10614	3164	-297	304
H9A	12205	8774	972	84
H7AA	10986	7896	597	107
H7AB	10916	8643	811	107
H7AC	10493	7771	1063	107
H8AA	12900	8802	2018	114
H8AB	13205	9038	1501	114
H8AC	12940	8140	1692	114
H6AA	12657	7779	1569	114
H6AB	12933	8486	1192	114
H6AC	12161	7592	1098	114
H2A	12353	9364	1240	84
H2AA	11227	9542	1462	107
H2AB	11964	9975	1821	107
H2AC	11189	9092	1933	107

S23 X-ray data of incaspitolide (**17)**

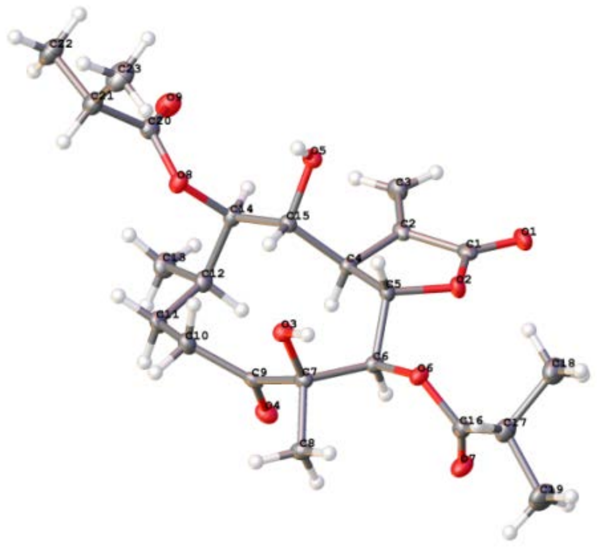

Table 1 Crystal data and structure refinement for **17**	
Identification code	exp_2705
Empirical formula	$C_{23}H_{34}O_9$
Formula weight	454.50
Temperature / K	101.0
Crystal system	monoclinic
Space group	$P2_1$
a / Å, b / Å, c / Å	13.5135(4), 9.5039(2), 18.8280(6)
α/°, β/°, γ/°	90.00, 105.051(3), 90.00
Volume / $Å^3$	2335.13(11)
Z	4
ρ_{calc} / mg mm^{-3}	1.293
μ / mm^{-1}	0.827
F(000)	976
Crystal size / mm^3	0.55 × 0.40 × 0.36
2Θ range for data collection	7.24 to 142.32°
Index ranges	$-16 \leq h \leq 15$, $-11 \leq k \leq 11$, $-16 \leq l \leq 23$
Reflections collected	16917
Independent reflections	8859[R(int) = 0.0244 (inf-0.9Å)]
Data/restraints/parameters	8859/1/593
Goodness-of-fit on F^2	1.024
Final R indexes [I>2σ (I) i.e. F_o>4σ (F_o)]	R_1 = 0.0390, wR_2 = 0.1006
Final R indexes [all data]	R_1 = 0.0397, wR_2 = 0.1012
Largest diff. peak/hole / e $Å^{-3}$	0.759/-0.445
Flack Parameters	0.00(11)
Completeness	0.988

Table 2 Fractional Atomic Coordinates (×10^4) and Equivalent Isotropic Displacement Parameters (Å^2×10^3) for **17**. U_{eq} is defined as 1/3 of of the trace of the orthogonalised U_{IJ} tensor.

**Atom**	***x***	***y***	***z***	**U(eq)**
O3	9387.3(10)	226.2(14)	4251.1(7)	15.6(3)
O7	10993.7(11)	2018.2(17)	2507.5(7)	23.6(3)
O5	8648.2(10)	3946.5(14)	5219.6(7)	16.2(3)
O4	7788.5(10)	1596.7(16)	2607.9(7)	19.5(3)
O1	10613.3(11)	6631.8(16)	3957.4(8)	22.7(3)
O6	10903.6(10)	1638.1(15)	3670.5(7)	16.0(3)
O2	10518.7(10)	4296.3(14)	3967.2(7)	17.4(3)
O9	5806.2(12)	4558.4(16)	5129.0(8)	24.9(3)
O8	6693.7(10)	2718.7(14)	4855.5(7)	16.7(3)
C12	6441.9(14)	3023(2)	3544.5(11)	17.1(4)
C15	8211.7(13)	3314(2)	4517.8(10)	13.5(3)
C3	8506.1(15)	6581(2)	4137.7(11)	20.1(4)
C9	8059.9(15)	1028.1(19)	3201.5(10)	16.1(4)
C20	6057.9(15)	3348(2)	5208.9(11)	18.1(4)
C1	10118.9(15)	5581(2)	3980.5(10)	17.5(4)
C5	9805.2(14)	3199(2)	4050.5(10)	14.2(4)
C16	11399.3(15)	1680(2)	3129.2(10)	17.6(4)
C4	8753.9(14)	3925(2)	3960.4(10)	13.6(4)
C7	9211.7(14)	729(2)	3517.6(10)	14.6(4)
C11	6320.2(14)	1421(2)	3446.4(11)	19.8(4)
C6	9846.7(14)	2069(2)	3486(1)	14.4(4)
C2	9038.7(14)	5468(2)	4035.3(10)	14.7(4)
C14	7046.6(14)	3521(2)	4314(1)	15.5(4)
C13	5374.4(15)	3694(2)	3349.2(12)	23.2(4)
C8	9469.8(15)	-419(2)	3012.8(10)	18.0(4)
C18	13059.4(17)	2394(3)	3967.5(13)	31.3(5)
C23	6667.2(19)	1878(3)	6333.0(13)	33.2(5)
C10	7296.5(15)	532(2)	3613.6(11)	19.1(4)
C22	4869.1(17)	2894(3)	6007.6(13)	29.6(5)
C17	12501.4(15)	1241(2)	3442.1(11)	22.3(4)
C19	13017.1(17)	934(3)	2825.3(14)	33.0(5)
C21	5734.6(16)	2300(2)	5711.5(11)	21.6(4)
O11	11063.3(10)	2713.3(15)	932.6(7)	18.4(3)
O17	6716.9(10)	4731.1(14)	83.2(7)	17.2(3)
O12	10034.8(10)	6925.2(15)	763.1(7)	18.4(3)

O10	11049.3(12)	377.1(16)	984.6(8)	24.5(3)
O13	9489.6(11)	5532.0(16)	2357.0(7)	20.5(3)
O14	8320.3(10)	3258.3(15)	-251.2(7)	17.0(3)
O15	11840.9(10)	5272.6(16)	1265.5(8)	21.6(3)
O16	12831.0(12)	4388(2)	2328.3(9)	35.5(4)
O18	5505.1(11)	3040.5(17)	-187.6(8)	24.8(3)
C32	9396.8(15)	6141.9(19)	1780.1(10)	16.2(4)
C43	5803.9(15)	4189(2)	-290.9(10)	17.9(4)
C24	10599.5(15)	1480(2)	961(1)	17.8(4)
C28	10360.2(14)	3889(2)	900.4(10)	15.4(4)
C26	8872.7(16)	637(2)	913.3(11)	23.3(4)
C37	7400.1(14)	3875(2)	643.9(10)	16.4(4)
C35	7349.7(14)	4428(2)	1401.8(10)	18.2(4)
C38	8430.5(14)	3930(2)	445.7(10)	14.9(4)
C34	7450.8(16)	6026(2)	1511.6(11)	21.5(4)
C30	10352.9(15)	6358(2)	1485.7(10)	16.8(4)
C31	11043.4(16)	7391(2)	2018.7(11)	21.4(4)
C45	4159.8(17)	4670(3)	-1246.7(12)	28.0(5)
C27	9350.6(14)	3275(2)	1010.7(10)	13.7(3)
C36	6333.8(16)	3976(3)	1549.4(12)	24.8(4)
C25	9508.5(15)	1707(2)	951.2(10)	16.7(4)
C29	10912.1(14)	4947(2)	1478.6(10)	15.2(4)
C39	12747.9(16)	4864(3)	1737.9(14)	32.1(5)
C44	5212.4(15)	5255(2)	-839.6(11)	21.9(4)
C33	8383.4(15)	6773(2)	1361.3(10)	18.5(4)
C42	14133(3)	6509(4)	1694.4(16)	54.2(8)
C46	5833.2(19)	5735(3)	-1360.9(14)	36.2(6)
C41	13438(2)	4903(4)	622.1(15)	43.1(6)
C40	13642.2(19)	5123(4)	1415.9(16)	46.5(7)

Table 3 Anisotropic Displacement Parameters ($Å^2×10^3$) for **17**. The Anisotropic displacement factor exponent takes the form: $-2\pi^2[h^2a^{*2}U_{11}+...+2hka×b×U_{12}]$

**Atom**	**U_{11}**	**U_{22}**	**U_{33}**	**U_{23}**	**U_{13}**	**U_{12}**
O3	15.5(6)	17.4(7)	13.5(6)	2.6(5)	2.7(5)	2.1(5)
O7	19.1(7)	37.5(9)	14.8(7)	1.2(6)	5.6(5)	-1.3(6)
O5	17.7(6)	16.3(6)	13.5(6)	1.4(5)	1.8(5)	-1.0(5)
O4	19.0(6)	20.4(7)	15.6(6)	-0.2(5)	-1.6(5)	0.3(6)
O1	26.7(7)	20.0(7)	21.2(7)	-2.8(6)	5.9(6)	-10.8(6)
O6	12.8(6)	21.4(7)	14.1(6)	1.4(5)	4.0(5)	1.5(5)
O2	13.3(6)	17.1(7)	21.2(7)	-1.8(5)	3.5(5)	-4.5(5)

O9	28.1(7)	22.0(8)	27.0(7)	3.6(6)	11.4(6)	6.0(6)
O8	16.5(6)	16.4(6)	18.3(6)	3.9(5)	6.3(5)	1.5(5)
C12	12.3(8)	20.8(10)	17.1(9)	2.6(7)	2.0(7)	-0.3(7)
C15	13.6(8)	12.2(8)	13.6(8)	0.2(7)	1.9(7)	-0.8(7)
C3	22.6(9)	14.6(9)	23.5(10)	0.2(8)	6.5(8)	-1.3(8)
C9	19.3(9)	12.0(8)	15.6(9)	-4.7(7)	1.9(7)	-2.0(7)
C20	14.7(8)	21(1)	17.6(9)	0.2(8)	2.7(7)	0.4(8)
C1	21.4(10)	19.6(10)	10.4(8)	-1.0(7)	2.1(7)	-3.2(8)
C5	11.8(8)	15.4(9)	14.5(8)	0.9(7)	1.7(6)	-2.2(7)
C16	19.1(9)	15.5(9)	19.5(9)	-1.8(7)	7.4(7)	-3.0(8)
C4	12.6(8)	14.6(9)	13.0(8)	-1.0(7)	2.1(7)	-0.5(7)
C7	17.0(9)	13.9(9)	12.8(8)	0.4(7)	3.5(7)	-0.1(7)
C11	12.4(8)	24.3(10)	21.2(9)	-3.2(8)	1.7(7)	-4.4(8)
C6	12.4(8)	17.5(9)	12.7(8)	0.0(7)	2.3(7)	1.5(7)
C2	17.9(9)	13.8(9)	10.8(8)	2.1(7)	0.5(7)	-2.2(7)
C14	13.2(9)	16.2(9)	17.3(9)	2.9(7)	4.1(7)	1.0(7)
C13	13.5(9)	31.4(12)	22.1(10)	4.1(8)	0.0(8)	2.0(8)
C8	24.0(9)	15.3(9)	15.4(9)	-0.9(7)	6.3(7)	0.7(8)
C18	19.5(10)	46.5(14)	27.2(11)	-1.5(10)	5.1(9)	-5.3(10)
C23	30.6(12)	41.7(14)	28.4(11)	13.8(10)	9.7(9)	7.4(10)
C10	16.2(9)	18.1(9)	22.0(9)	-3.0(8)	3.0(7)	-3.0(7)
C22	27.7(11)	34.8(13)	30.8(12)	3.7(10)	15.5(9)	1.6(10)
C17	16.6(9)	27.7(11)	23.2(10)	2.7(8)	6.0(8)	1.3(8)
C19	21(1)	44.9(14)	36.1(13)	-2.5(11)	12.9(10)	4.1(10)
C21	21.4(10)	22.7(10)	22.4(9)	0.8(8)	8.5(8)	-1.8(8)
O11	17.5(6)	18.9(7)	19.8(7)	-1.4(5)	6.7(5)	2.8(6)
O17	15.9(6)	18.5(7)	16.2(6)	4.9(5)	2.2(5)	3.1(5)
O12	19.7(6)	19.6(7)	14.6(6)	3.3(5)	2.3(5)	-3.7(5)
O10	32.3(8)	18.7(7)	22.6(7)	-2.6(6)	7.3(6)	8.4(6)
O13	23.8(7)	23.7(7)	14.3(6)	-2.1(6)	5.6(5)	-0.7(6)
O14	21.3(7)	16.2(6)	13.0(6)	0.6(5)	3.6(5)	-1.6(5)
O15	17.4(7)	27.9(8)	21.8(7)	-2.2(6)	9.1(6)	-7.8(6)
O16	20.0(7)	60.3(12)	22.7(8)	-7.0(8)	-0.8(6)	3.8(8)
O18	20.3(7)	27.8(8)	24.3(7)	4.4(6)	1.9(6)	-1.5(6)
C32	21.8(9)	12.4(9)	14.1(9)	-5.7(7)	4.4(7)	-2.2(7)
C43	17.4(9)	20.8(10)	15.8(9)	-0.6(8)	4.9(7)	2.9(8)
C24	24.5(10)	17.9(10)	10.2(8)	-2.2(7)	3.2(7)	1.9(8)
C28	15.6(9)	15.8(9)	14.4(9)	1.1(7)	3.1(7)	1.1(7)
C26	23.1(10)	18.8(10)	25.2(10)	3.9(8)	1.4(8)	-1.3(8)
C37	15.6(9)	15.9(9)	17.1(9)	4.4(7)	3.3(7)	0.7(7)

C35	14.9(9)	25(1)	14.7(9)	4.0(8)	3.6(7)	2.8(8)
C38	15.9(9)	13.6(9)	14.6(9)	-0.4(7)	3.0(7)	-0.5(7)
C34	19.4(9)	26.5(11)	19.6(9)	-1.9(8)	7.0(8)	4.2(8)
C30	18.0(9)	16.7(9)	15.0(9)	0.1(7)	2.9(7)	-1.4(7)
C31	23.9(10)	19.1(10)	19.5(9)	-1.5(8)	2.6(8)	-3.5(8)
C45	25.5(11)	30.0(12)	23.8(10)	2.1(9)	-1.8(8)	4.6(9)
C27	13.5(8)	14.8(9)	12.9(8)	-1.9(7)	3.4(7)	-1.9(7)
C36	19.9(10)	36.3(12)	19.6(9)	4.8(9)	7.4(8)	0.9(9)
C25	21.1(9)	16.6(9)	10.7(8)	1.1(7)	1.1(7)	1.7(8)
C29	13.0(8)	18.7(9)	14.5(8)	0.0(7)	4.4(7)	-2.7(7)
C39	14.8(9)	45.5(14)	35.9(13)	-17.6(11)	6.8(9)	-7.0(9)
C44	22.1(10)	23.3(10)	18.4(9)	1.5(8)	1.5(8)	6.5(8)
C33	21.0(9)	16.2(9)	18.5(9)	-1.5(7)	5.6(7)	2.6(8)
C42	72(2)	57.4(19)	39.7(15)	-9.9(14)	25.9(14)	-37.4(17)
C46	30.2(12)	47.0(15)	30.1(12)	17.2(11)	5.5(10)	4.9(11)
C41	39.2(14)	58.6(18)	36.6(13)	-7.1(13)	19.2(11)	-6.8(13)
C40	24.2(12)	76(2)	41.7(15)	-7.7(14)	13.0(11)	-5.6(13)

Table 4 Bond Lengths for **17**.

Atom	Atom	Length/Å	Atom	Atom	Length/Å
O3	C7	1.422(2)	O11	C24	1.337(3)
O7	C16	1.200(2)	O11	C28	1.458(2)
O5	C15	1.432(2)	O17	C43	1.354(2)
O4	C9	1.210(2)	O17	C37	1.457(2)
O1	C1	1.209(2)	O12	C30	1.422(2)
O6	C16	1.358(2)	O10	C24	1.207(3)
O6	C6	1.439(2)	O13	C32	1.209(2)
O2	C1	1.338(2)	O14	C38	1.432(2)
O2	C5	1.456(2)	O15	C29	1.447(2)
O9	C20	1.198(3)	O15	C39	1.370(3)
O8	C20	1.355(2)	O16	C39	1.178(3)
O8	C14	1.449(2)	O18	C43	1.197(3)
C12	C11	1.538(3)	C32	C30	1.546(3)
C12	C14	1.541(3)	C32	C33	1.515(3)
C12	C13	1.532(3)	C43	C44	1.518(3)
C15	C4	1.541(2)	C24	C25	1.486(3)
C15	C14	1.533(2)	C28	C27	1.547(2)
C3	C2	1.321(3)	C28	C29	1.526(3)
C9	C7	1.542(3)	C26	C25	1.321(3)
C9	C10	1.518(3)	C37	C35	1.539(3)

C20	C21	1.515(3)	C37	C38	1.533(3)
C1	C2	1.494(3)	C35	C34	1.535(3)
C5	C4	1.549(2)	C35	C36	1.531(3)
C5	C6	1.522(3)	C38	C27	1.542(2)
C16	C17	1.511(3)	C34	C33	1.535(3)
C4	C2	1.513(3)	C30	C31	1.535(3)
C7	C6	1.545(3)	C30	C29	1.541(3)
C7	C8	1.545(3)	C45	C44	1.534(3)
C11	C10	1.529(3)	C27	C25	1.513(3)
C18	C17	1.537(3)	C39	C40	1.506(3)
C23	C21	1.534(3)	C44	C46	1.518(3)
C22	C21	1.529(3)	C42	C40	1.506(4)
C17	C19	1.529(3)	C41	C40	1.463(4)

Table 5 Bond Angles for **17**.

Atom	Atom	Atom	Angle/˚	Atom	Atom	Atom	Angle/˚
C16	O6	C6	117.55(14)	C24	O11	C28	111.51(14)
C1	O2	C5	111.68(14)	C43	O17	C37	119.00(15)
C20	O8	C14	118.66(15)	C39	O15	C29	116.97(16)
C11	C12	C14	115.53(16)	O13	C32	C30	118.98(17)
C13	C12	C11	108.58(16)	O13	C32	C33	121.77(18)
C13	C12	C14	109.53(16)	C33	C32	C30	119.16(16)
O5	C15	C4	108.38(14)	O17	C43	C44	110.36(17)
O5	C15	C14	109.10(14)	O18	C43	O17	124.25(18)
C14	C15	C4	114.84(15)	O18	C43	C44	125.37(18)
O4	C9	C7	118.20(17)	O11	C24	C25	110.24(17)
O4	C9	C10	121.86(18)	O10	C24	O11	121.75(18)
C10	C9	C7	119.84(16)	O10	C24	C25	128.01(19)
O9	C20	O8	123.92(18)	O11	C28	C27	107.04(15)
O9	C20	C21	126.28(19)	O11	C28	C29	106.43(14)
O8	C20	C21	109.81(17)	C29	C28	C27	115.94(15)
O1	C1	O2	121.58(18)	O17	C37	C35	107.98(15)
O1	C1	C2	128.38(19)	O17	C37	C38	104.43(14)
O2	C1	C2	110.04(16)	C38	C37	C35	118.41(16)
O2	C5	C4	106.46(15)	C34	C35	C37	116.05(16)
O2	C5	C6	106.54(14)	C36	C35	C37	109.28(16)
C6	C5	C4	115.92(15)	C36	C35	C34	107.82(17)
O7	C16	O6	123.41(18)	O14	C38	C37	108.84(15)
O7	C16	C17	127.47(18)	O14	C38	C27	109.01(15)
O6	C16	C17	109.12(16)	C37	C38	C27	116.02(15)

C15	C4	C5	110.01(15)	C35	C34	C33	118.80(17)
C2	C4	C15	117.15(15)	O12	C30	C32	108.67(15)
C2	C4	C5	102.58(14)	O12	C30	C31	111.36(16)
O3	C7	C9	109.51(14)	O12	C30	C29	110.40(15)
O3	C7	C6	111.11(15)	C31	C30	C32	105.86(15)
O3	C7	C8	110.64(15)	C31	C30	C29	110.26(15)
C9	C7	C6	110.40(15)	C29	C30	C32	110.18(15)
C9	C7	C8	105.02(15)	C38	C27	C28	109.64(15)
C6	C7	C8	109.99(15)	C25	C27	C28	102.44(15)
C10	C11	C12	117.56(16)	C25	C27	C38	116.76(15)
O6	C6	C5	104.26(14)	C24	C25	C27	107.51(16)
O6	C6	C7	106.42(15)	C26	C25	C24	121.20(19)
C5	C6	C7	115.66(15)	C26	C25	C27	131.26(19)
C3	C2	C1	122.13(18)	O15	C29	C28	103.97(14)
C3	C2	C4	130.85(18)	O15	C29	C30	106.20(15)
C1	C2	C4	107.01(16)	C28	C29	C30	115.43(15)
O8	C14	C12	107.98(15)	O15	C39	C40	111.6(2)
O8	C14	C15	105.10(14)	O16	C39	O15	124.8(2)
C15	C14	C12	117.08(15)	O16	C39	C40	123.6(2)
C9	C10	C11	112.82(17)	C43	C44	C45	110.75(18)
C16	C17	C18	108.83(18)	C43	C44	C46	111.05(17)
C16	C17	C19	110.73(17)	C46	C44	C45	112.48(18)
C19	C17	C18	112.37(18)	C32	C33	C34	113.27(16)
C20	C21	C23	109.60(17)	C42	C40	C39	109.3(2)
C20	C21	C22	110.90(18)	C41	C40	C39	115.4(2)
C22	C21	C23	111.92(18)	C41	C40	C42	115.5(3)

Table 6 Torsion Angles for **17**.

**A**	**B**	**C**	**D**	**Angle/°**
O3	C7	C6	O6	67.89(17)
O3	C7	C6	C5	-47.4(2)
O7	C16	C17	C18	110.6(2)
O7	C16	C17	C19	-13.4(3)
O5	C15	C4	C5	79.13(18)
O5	C15	C4	C2	-37.4(2)
O5	C15	C14	O8	-65.28(18)
O5	C15	C14	C12	174.92(15)
O4	C9	C7	O3	172.71(16)
O4	C9	C7	C6	50.1(2)
O4	C9	C7	C8	-68.5(2)

O4	C9	C10	C11	-29.7(3)
O1	C1	C2	C3	-6.7(3)
O1	C1	C2	C4	173.89(18)
O6	C16	C17	C18	-69.5(2)
O6	C16	C17	C19	166.48(18)
O2	C1	C2	C3	172.55(17)
O2	C1	C2	C4	-6.88(19)
O2	C5	C4	C15	-140.13(14)
O2	C5	C4	C2	-14.77(17)
O2	C5	C6	O6	58.62(17)
O2	C5	C6	C7	175.08(14)
O9	C20	C21	C23	-113.1(2)
O9	C20	C21	C22	10.9(3)
O8	C20	C21	C23	66.4(2)
O8	C20	C21	C22	-169.52(17)
C12	C11	C10	C9	-53.1(2)
C15	C4	C2	C3	-45.8(3)
C15	C4	C2	C1	133.58(16)
C9	C7	C6	O6	-170.41(14)
C9	C7	C6	C5	74.35(19)
C20	O8	C14	C12	-103.86(18)
C20	O8	C14	C15	130.46(16)
C1	O2	C5	C4	11.66(18)
C1	O2	C5	C6	135.93(15)
C5	O2	C1	O1	176.14(17)
C5	O2	C1	C2	-3.15(19)
C5	C4	C2	C3	-166.35(19)
C5	C4	C2	C1	13.02(18)
C16	O6	C6	C5	-125.92(17)
C16	O6	C6	C7	111.36(17)
C4	C15	C14	O8	172.86(15)
C4	C15	C14	C12	53.1(2)
C4	C5	C6	O6	176.84(15)
C4	C5	C6	C7	-66.7(2)
C7	C9	C10	C11	154.08(16)
C11	C12	C14	O8	-44.3(2)
C11	C12	C14	C15	73.9(2)
C6	O6	C16	O7	-2.1(3)
C6	O6	C16	C17	177.98(16)
C6	C5	C4	C15	101.60(18)

C6	C5	C4	C2	-133.03(16)
C14	O8	C20	O9	-3.9(3)
C14	O8	C20	C21	176.50(15)
C14	C12	C11	C10	-56.5(2)
C14	C15	C4	C5	-158.61(15)
C14	C15	C4	C2	84.8(2)
C13	C12	C11	C10	-179.91(17)
C13	C12	C14	O8	78.63(19)
C13	C12	C14	C15	-163.11(16)
C8	C7	C6	O6	-54.97(18)
C8	C7	C6	C5	-170.21(15)
C10	C9	C7	O3	-10.9(2)
C10	C9	C7	C6	-133.54(17)
C10	C9	C7	C8	107.94(18)
O11	C24	C25	C26	174.32(17)
O11	C24	C25	C27	-7.1(2)
O11	C28	C27	C38	-135.25(15)
O11	C28	C27	C25	-10.62(18)
O11	C28	C29	O15	57.82(18)
O11	C28	C29	C30	173.71(14)
O17	C43	C44	C45	-177.94(16)
O17	C43	C44	C46	56.3(2)
O17	C37	C35	C34	-47.8(2)
O17	C37	C35	C36	74.29(19)
O17	C37	C38	O14	-64.75(18)
O17	C37	C38	C27	171.92(15)
O12	C30	C29	O15	65.37(18)
O12	C30	C29	C28	-49.2(2)
O10	C24	C25	C26	-5.1(3)
O10	C24	C25	C27	173.42(18)
O13	C32	C30	O12	172.39(16)
O13	C32	C30	C31	-67.9(2)
O13	C32	C30	C29	51.3(2)
O13	C32	C33	C34	-26.9(3)
O14	C38	C27	C28	78.42(18)
O14	C38	C27	C25	-37.4(2)
O15	C39	C40	C42	96.2(3)
O15	C39	C40	C41	-35.9(4)
O16	C39	C40	C42	-82.7(4)
O16	C39	C40	C41	145.2(3)

O18	C43	C44	C45	0.6(3)
O18	C43	C44	C46	-125.1(2)
C32	C30	C29	O15	-174.59(14)
C32	C30	C29	C28	70.79(19)
C43	O17	C37	C35	-104.55(18)
C43	O17	C37	C38	128.57(16)
C24	O11	C28	C27	7.04(19)
C24	O11	C28	C29	131.61(15)
C28	O11	C24	O10	179.44(17)
C28	O11	C24	C25	-0.05(19)
C28	C27	C25	C24	10.59(18)
C28	C27	C25	C26	-171.1(2)
C37	O17	C43	O18	0.9(3)
C37	O17	C43	C44	179.45(15)
C37	C35	C34	C33	-54.2(2)
C37	C38	C27	C28	-158.33(16)
C37	C38	C27	C25	85.8(2)
C35	C37	C38	O14	175.14(15)
C35	C37	C38	C27	51.8(2)
C35	C34	C33	C32	-53.8(2)
C38	C37	C35	C34	70.4(2)
C38	C37	C35	C36	-167.44(17)
C38	C27	C25	C24	130.37(16)
C38	C27	C25	C26	-51.3(3)
C30	C32	C33	C34	156.77(16)
C31	C30	C29	O15	-58.11(19)
C31	C30	C29	C28	-172.72(16)
C27	C28	C29	O15	176.72(15)
C27	C28	C29	C30	-67.4(2)
C36	C35	C34	C33	-177.09(17)
C29	O15	C39	O16	-7.2(3)
C29	O15	C39	C40	173.9(2)
C29	C28	C27	C38	106.19(18)
C29	C28	C27	C25	-129.18(16)
C39	O15	C29	C28	-114.27(19)
C39	O15	C29	C30	123.51(18)
C33	C32	C30	O12	-11.1(2)
C33	C32	C30	C31	108.57(19)
C33	C32	C30	C29	-132.23(17)

Table 7 Hydrogen Atom Coordinates (Å×10^4) and Isotropic Displacement Parameters (Å^2×10^3)

for **17**.

**Atom**	***x***	***y***	***z***	**U(eq)**
H3	9988	-15	4403	23
H5	8804	3331	5534	24
H12	6803	3365	3189	20
H15	8352	2302	4561	16
H3A	8802	7470	4178	24
H3B	7835	6475	4169	24
H5A	10032	2797	4545	17
H4	8328	3752	3461	16
H11A	5899	1092	3759	24
H11B	5944	1238	2942	24
H6	9653	2469	2989	17
H14	6899	4519	4368	19
H13A	4995	3373	2873	35
H13B	5440	4699	3343	35
H13C	5020	3429	3709	35
H8A	10170	-707	3201	27
H8B	9373	-49	2525	27
H8C	9027	-1214	2999	27
H18A	13061	3251	3698	47
H18B	13752	2105	4186	47
H18C	12713	2546	4346	47
H23A	7194	1514	6126	50
H23B	6470	1168	6632	50
H23C	6921	2687	6629	50
H10A	7618	565	4138	23
H10B	7115	-439	3483	23
H22A	5101	3732	6286	44
H22B	4670	2209	6319	44
H22C	4292	3116	5604	44
H17	12508	375	3726	27
H19A	12677	159	2535	49
H19B	13724	700	3035	49
H19C	12974	1751	2519	49
H21	5478	1455	5423	26
H12A	10540	7164	628	28
H14A	8333	3854	-564	26
H28	10223	4327	413	18

H26A	9106	-279	892	28
H26B	8192	800	908	28
H37	7151	2902	592	20
H35	7905	3983	1774	22
H38	8592	4922	389	18
H34A	7448	6226	2016	26
H34B	6841	6455	1199	26
H31A	11663	7542	1870	32
H31B	11208	7008	2507	32
H31C	10691	8270	2012	32
H45A	4249	3867	-1534	42
H45B	3779	5382	-1565	42
H45C	3792	4394	-896	42
H27	9301	3497	1508	16
H36A	5775	4375	1182	37
H36B	6307	4300	2027	37
H36C	6282	2968	1531	37
H29	11078	4518	1969	18
H44	5095	6080	-561	26
H33A	8335	6729	839	22
H33B	8368	7757	1495	22
H42A	13686	7265	1476	81
H42B	14772	6599	1565	81
H42C	14254	6547	2220	81
H46A	6487	6078	-1082	54
H46B	5473	6475	-1670	54
H46C	5932	4958	-1661	54
H41A	13191	3962	503	65
H41B	14058	5037	472	65
H41C	12930	5564	370	65
H40	14151	4408	1638	56

S24 ^{1}H and ^{13}C NMR spectroscopic data of **17** (600 and 150 MHz in CD_3OD; δ in ppm, *J* in Hz).

position	**17**	
	δ_H	δ_C
1	1.87 m, 1.75 m	25.3
2	3.85 br d (10.2), 2.22 o	32.9
3		217.6

4		80.4
5	5.43 dd (9.6,2.4)	78.1
6	4.70 dd (9.6,6.6)	80.0
7	3.05 m	41.5
8	4.44 d (10.2)	70.3
9	5.17 d(10.2)	78.5
10	2.22 o	30.0
11		132.7
12		169.6
13	6.35 d (3.0), 5.70 d (2.4)	123.8
14	0.99 d (6.6)	19.9
15	1.26 s	23.3
1′		176.4
2′	2.71 o	34.0
3′	1.28 d (6.6)	18.0
4′	1.27 d (6.6)	17.8
1″		177.2
2″	2.71 o	34.1
3″	1.26 d (7.2)	18.5
4″	1.26 d (7.2)	17.9
